# Genome Sequencing Reveals the Potential of *Achromobacter* sp. HZ01 for Bioremediation

**DOI:** 10.3389/fmicb.2017.01507

**Published:** 2017-08-09

**Authors:** Yue-Hui Hong, Cong-Cong Ye, Qian-Zhi Zhou, Xiao-Ying Wu, Jian-Ping Yuan, Juan Peng, Hailin Deng, Jiang-Hai Wang

**Affiliations:** ^1^Guangdong Provincial Key Laboratory of Marine Resources and Coastal Engineering/South China Sea Bioresource Exploitation and Utilization Collaborative Innovation Center, School of Marine Sciences, Sun Yat-sen University Guangzhou, China; ^2^State Key Laboratory of Conservation and Utilization of Subtropical Agro-Bioresources, College of Natural Resources and Environment, South China Agricultural University Guangzhou, China

**Keywords:** *Achromobacter*, bioremediation, genome, hydrocarbon, petroleum pollution, strain HZ01

## Abstract

Petroleum pollution is a severe environmental issue. Comprehensively revealing the genetic backgrounds of hydrocarbon-degrading microorganisms contributes to developing effective methods for bioremediation of crude oil-polluted environments. Marine bacterium *Achromobacter* sp. HZ01 is capable of degrading hydrocarbons and producing biosurfactants. In this study, the draft genome (5.5 Mbp) of strain HZ01 has been obtained by Illumina sequencing, containing 5,162 predicted genes. Genome annotation shows that “amino acid metabolism” is the most abundant metabolic pathway. Strain HZ01 is not capable of using some common carbohydrates as the sole carbon sources, which is due to that it contains few genes associated with carbohydrate transport and lacks some important enzymes related to glycometabolism. It contains abundant proteins directly related to petroleum hydrocarbon degradation. AlkB hydroxylase and its homologs were not identified. It harbors a complete enzyme system of terminal oxidation pathway for *n*-alkane degradation, which may be initiated by cytochrome P450. The enzymes involved in the catechol pathway are relatively complete for the degradation of aromatic compounds. This bacterium lacks several essential enzymes for methane oxidation, and Baeyer-Villiger monooxygenase involved in the subterminal oxidation pathway and cycloalkane degradation was not identified. These results suggest that strain HZ01 degrades *n*-alkanes via the terminal oxidation pathway, degrades aromatic compounds primarily via the catechol pathway and cannot perform methane oxidation or cycloalkane degradation. Additionally, strain HZ01 possesses abundant genes related to the metabolism of secondary metabolites, including some genes involved in biosurfactant (such as glycolipids and lipopeptides) synthesis. The genome analysis also reveals its genetic basis for nitrogen metabolism, antibiotic resistance, regulatory responses to environmental changes, cell motility, and material transport. The obtained genome data provide us with a better understanding of hydrocarbon-degrading bacteria, which may contribute to the future design of rational strategies for bioremediation of petroleum-polluted marine environments.

## Introduction

Petroleum pollution in marine environments mainly caused by anthropogenic activities is a serious environmental issue due to its negative impacts on human health and ecosystems. For instance, the “Deep Water Horizon” oil spill accident in the Gulf of Mexico was one of the most dramatic pollution events, which had resulted in a serious damage to the marine sites (Xue et al., 2015). The maintenance of ecological balance imperatively requires the development of effective ways to remediate crude oil-polluted environments. Among the proposed remediation techniques for the treatment of marine oil pollution, microbial remediation has been regarded as one of the most reliable strategies for the thorough elimination of petroleum contaminants (Hassanshahian et al., 2012). Revealing the genomic backgrounds of hydrocarbon-degrading bacteria contributes to developing effective methods to reduce oil contamination and mitigate its environmental damage. High-throughput sequencing is providing us with novel knowledge on the underlying mechanisms in microorganisms conducting oil degradation. So far, the genomes of a few hydrocarbon-degrading bacteria have been analyzed in depth. These genomes exhibit various characteristic differences. This is in accord with the inference that the degradation properties of hydrocarbon-degrading bacteria are generally different (Rojo, 2009). For instance, *Alcanivorax borkumensis* SK2 contains a streamlined genome with few energy production-related genes and mobile genetic elements, but with abundant genes related to oil degradation (Schneiker et al., 2006). Compared with strain SK2, *Oleispira antarctica* RB-8 has a larger genome with massive gene-transfer events (Kube et al., 2013). In view of the characteristic differences among diverse oil-degrading bacteria and the complexity of petroleum degradation, genome-wide elucidation of the entire degradation mechanisms of petroleum hydrocarbons is still in a relatively early stage.

Some strains belonging to the genus *Achromobacter* are regarded as representative bacteria with bioremediation potential, as diverse bioremediation properties of this bacterial population have been reported, e.g., biphenyl catabolism (Furukawa et al., 1989), arsenite oxidation (Cai et al., 2009), haloaromatic acid degradation (Jencova et al., 2008), detoxification of chromium-containing slag (Chai et al., 2010), and hydrocarbon degradation (Deng et al., 2014). Many genome sequences of *Achromobacter* spp. are already known, and some characteristics of *Achromobacter* strains have been deeply analyzed at the genomic level. For instance, the pathogenic mechanisms of opportunistic pathogen *Achromobacter xylosoxidans* NH44784-1996 has been revealed using complete genome sequencing (Jakobsen et al., 2013). However, genome-wide researches on elucidating (i) the pathways of hydrocarbon degradation, (ii) the biosynthesis of secondary metabolites, and (iii) the genetic basis for environment adaptation in hydrocarbon-degrading *Achromobacter* spp. are currently lacking.

*Achromobacter* sp. HZ01, isolated from the crude oil-polluted seawater in the South China Sea, is capable of degrading petroleum hydrocarbons and producing biosurfactants (Deng et al., 2014, 2016), exhibiting a good potential for various applications. A *de novo* transcriptome of strain HZ01 regarding hydrocarbon degradation was previously reported, and some functional genes and pathways were analyzed (Hong et al., 2016). However, RNA-seq-based transcriptomics cannot cover all genes and pathways because it focuses on the expressed genes under specific conditions. To reveal the genetic background of strain HZ01 more comprehensively, it is necessary to carry out whole genome sequencing.

In this study, we report the draft genome of strain HZ01. The genome analysis contributed to better understanding its genetic basis for petroleum degradation, production of secondary metabolites, antibiotic resistance, and some other important physiological functions. The present work may also provide a basis for developing a cost-effective and eco-friendly method to remediate crude oil-contaminated marine environments.

## Materials and Methods

### Strain and Carbon Source Utilization

Strain HZ01 was isolated from crude oil-polluted seawater at the Daya Bay, South China Sea, as previously described (Deng et al., 2014). It was deposited in the China Center for Type Culture Collection with the preservation number of “CCTCC AB 2013198.”

Medium A (pH 7.5) contained (g/L): NH_4_NO_3_, 2.5; Na_2_HPO_4_⋅12H_2_O, 2; KH_2_PO_4_, 1; MgSO_4_⋅7H_2_O, 0.2; NaCl, 10; and trace element solution (1 mL/L). Medium A was supplemented with single carbon source (glucose, D-fructose, D-galactose, lactose, sucrose, D-mannose, D-maltose, mannitol, pyruvic acid, glycerol, and citric acid, respectively). The trace element solution was composed of (mg/L): CaCl_2_, 20; CuSO_4_, 0.5; MnSO_4_⋅H_2_O, 0.5; FeCl_3_, 30; and ZnSO_4_⋅7H_2_O, 10. Strain HZ01 was incubated in the Luria–Bertani (LB) medium at 28°C and 150 rpm for 16 h. Bacterial cells in the logarithmic phase were collected by centrifugation (2,600 *g*) at room temperature for 2 min. Then, the cells were resuspended and inoculated into medium A with an inoculum dose of 10% (v/v), followed by incubation at 28°C and 150 rpm. At indicated time points, the optical densities (OD_600_) of the culture broths were measured using a spectrophotometer. The medium without bacteria inoculation at each time point served as a negative control. The obtained OD_600_ values were used for plotting growth curves, to determine whether strain HZ01 was capable of utilizing an indicated compound as the sole carbon and energy source. The experiments were performed in triplicate with two repetitions.

### Emulsification Activity of the Culture Broth

Strain HZ01 was incubated in the LB medium at 28°C and 150 rpm for 16 h, followed by centrifugation (2,600 *g*) at room temperature for 2 min. The cell pellets were resuspended and inoculated into medium A with an inoculum dose of 10% (v/v). Citric acid (40 g/L) was used as the sole source of carbon and energy. The medium without bacteria inoculation served as a negative control. After incubation at 28°C and 150 rpm for 3 days, the culture broth was subjected to centrifugation (12,000 *g*) at room temperature for 2 min. Three milliliters of the resulting supernatants were mixed with an equal volume of soybean oil, coconut oil, olive oil, diesel oil, kerosene, and hexane, respectively. Three milliliters of sodium dodecyl sulfate (SDS; 0.5 g/L) were also mixed with those compounds, respectively, serving as positive controls. After being vortexed for 2 min, the mixtures were kept to settle at room temperature for 24 h, followed by the observation of emulsification layers. The experiments were performed in duplicate with two repetitions.

### Genome Sequencing

Strain HZ01 was incubated in the LB medium at 28°C and 150 rpm for 16 h before DNA extraction. The genomic DNA was extracted using the phenol-chloroform-isoamyl alcohol method (Sambrook and Russell, 2001). After quality verification of the isolated DNA, genome sequencing libraries with insert sizes of 500 and 800 bp were constructed, respectively. The Illumina HiSeq 2500 sequencing platform was employed to perform the genome sequencing using the paired-end (PE250) sequencing strategy.

### Sequence Assembly and Annotation

High-quality reads were obtained after quality control and elimination of adaptor sequences and low-quality reads, followed by sequence assembly into scaffolds using the Newbler v2.9 assembly tool (Roche Diagnostics). The CheckM (Parks et al., 2015) was employed to estimate the completeness of the draft genome. Protein-coding genes were predicted by the Genemark (Besemer et al., 2001). Gene annotation was performed by similarity searches (*E*-value ≤ 10^-5^) against the non-redundant database (NCBI-nr^1^), the Kyoto Encyclopedia of Genes and Genomes (KEGG^2^), and the evolutionary genealogy of genes: Non-supervised Orthologous Groups (eggNOG) (Jensen et al., 2008). Additionally, pathway-based functional annotation of the predicted genes was conducted using the KEGG Automatic Annotation Server (KAAS) (Moriya et al., 2007).

### Genome Comparisons and Gene Cluster Prediction

The general genome features of strain HZ01 and some other strains were compared. The genome synteny was analyzed by using the MUMmer (Kurtz et al., 2004) with the genomes of *Achromobacter xylosoxidans* A8 and *Achromobacter xylosoxidans* NH44784-1996 as a reference, respectively. Two-tailed Fisher exact test was employed to evaluate the differences in gene abundance of COG categories between the genome of strain HZ01 and other genomes in the Integrated Microbial Genomes (IMG) database (Markowitz et al., 2014).

The analysis of core and pan genome was carried out as previously described (Xiao et al., 2016; Ibrahim et al., 2017). The genome data of eight strains (including all *Achromobacter* species) were from the NCBI database. The core genome contains genes (core genes) present in the genomes of all indicated organisms. The pan genome is composed of core genes and a dispensable genome including genes unique to a genome and genes contained in two or more species (Medini et al., 2005). Single-copy genes were screened out according to the core and pan genome analysis. Protein sequence alignment was performed using the MUSCLE v3.8.31 (Edgar, 2004). The Treebest v1.9.2 (Vilella et al., 2009) was employed to construct a phylogenetic tree based on the core genes using the neighbor-joining method. Additionally, a phylogenetic tree based on the 16S rRNA genes was constructed using the neighbor-joining method. The sequences of 16S rRNA genes were from the NCBI database.

The antibiotics and secondary metabolite analysis shell (antiSMASH) (Weber et al., 2015) was employed to predict the gene clusters related to secondary metabolite biosynthesis.

### Analysis of Drug Resistance

To identify the antibiotic resistance genes in strain HZ01, homology alignment was performed using blastp (*E*-value < 10^-5^; percent identity ≥ 40%) against the Antibiotic Resistance Genes Database (ARDB) (Liu and Pop, 2009). The best hit was selected as the annotation of a query sequence.

The antibiotic resistance profile of strain HZ01 was determined by the Kirby-Bauer disk diffusion method (Cockerill et al., 2013) using *Escherichia coli* ATCC 25922 as a quality control. Briefly, bacterial cells were incubated in the LB medium for 16 h, followed by centrifugation (2,600 *g*) at room temperature for 2 min. The cell pellets were resuspended using sterile normal saline and adjusted to 0.5 McFarland standards. Then, the cells were spread onto the Mueller Hinton Agar (Guangdong Huankai Microbial Sci. & Tech. Co., Ltd., Guangzhou, China) plates using sterile cotton swabs. After being dried at room temperature for 5 min, the plates were placed with antibiotic disks (Hangzhou Microbial Reagent Co., Ltd., Hangzhou, China), followed by incubation at 35°C for 18 h. The inhibition zones were then measured, and the results were interpreted according to the standards of Clinical and Laboratory Standards Institute (CLSI) (Cockerill et al., 2013). The experiments were performed in triplicate with two repetitions.

### Data Deposition

The raw reads generated from genome sequencing were deposited in the Sequence Read Archive (SRA) under accession number SRP073408. The Whole Genome Shotgun project has been deposited at DDBJ/ENA/GenBank under accession number LWKV00000000. The version described in this paper is version LWKV01000000.

## Results

### General Features of the Draft Genome

**FIGURE 1:**
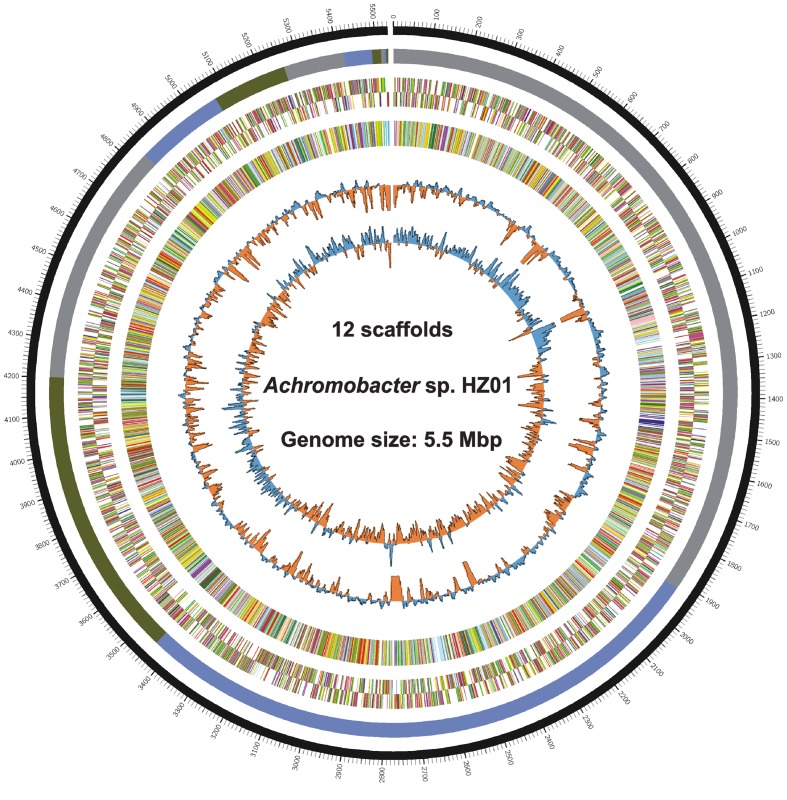
Circular chromosome of *Achromobacter* sp. HZ01. The circular genome map from the outside inward: ring 1, genomic position; ring 2, assembled scaffolds in order of size; rings 3 and 4, predicted genes on the forward (ring 3) and the reverse (ring 4) strands; ring 5, genes assigned to COG categories; ring 6, GC content; ring 7, GC skew. The circular map was generated by Circos v0.65 (Krzywinski et al., 2009).

Genome sequencing generated 1.87 and 1.34 M pairs of clean reads for libraries A and B, respectively, providing a 140 × coverage of the genome. The quality control information of the Illumina sequencing was shown in Supplementary Table S1 and Figure S1. After sequence assembly, the draft genome (5,532,918 bp; with a GC content of 68.1%) of strain HZ01 was obtained, containing 12 scaffolds (**Table 1** and **Figure 1**). The genome contained 5,162 predicted genes with an average length of 990 bp, including 4 rRNA and 54 tRNA genes.

**Table 1:** General features of the *Achromobacter* sp. HZ01 genome.

Feature	Count/Value
Genome size (bp)	5,532,918
Completeness (%)	99.5
GC content (%)	68.1
Scaffolds	12
Length of the longest scaffold (bp)	1,918,965
Genes	5,162
Gene length (bp)	5,108,407
Gene average length (bp)	990
Genes (RNA)^a^	62
Pseudogenes^a^	31
CDS^a^	5,078
rRNAs (5S, 16S, 23S)^a^	4 (2, 1, 1)
tRNAs^a^	54


*^a^Annotation is added by the NCBI Prokaryotic Genome Annotation Pipeline.*

A total of 5,081 genes were annotated in the NCBI-nr database (Supplementary Table S2). Annotation using the KAAS revealed that more genes were enriched in “amino acid metabolism” (Supplementary Figure S2). More specifically, “ABC transporters” was the most abundant subcategories among the annotated metabolic pathways, followed by “biosynthesis of amino acids,” “two-component system,” and “carbon metabolism” (Supplementary Figure S3). Annotation in the eggNOG database showed that most genes were assigned to “amino acid transport and metabolism,” “general function prediction only,” “transcription,” “function unknown,” and “inorganic ion transport and metabolism” (Supplementary Table S3).

### Carbon Source Utilization

**FIGURE 2:**
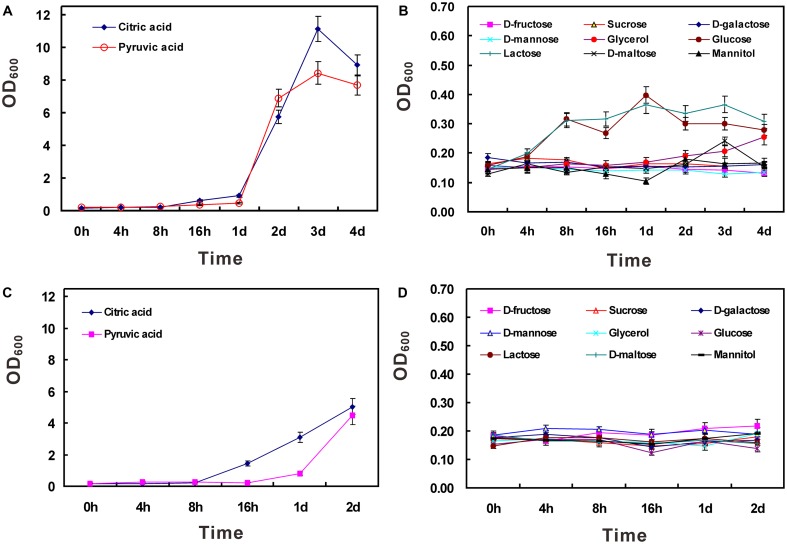
Utilization of carbon sources in strain HZ01. The incubation was performed using medium A supplemented with single carbon source at the concentration of 40 **(A,B)** and 20 g/L **(C,D)**, respectively. Data are presented as mean ± standard deviation (SD) involving triplicate assays. OD_600_, optical density at 600 nm; h, hour; d, day.

It has been demonstrated that strain HZ01 is capable of utilizing NH_4_NO_3_ as a nitrogen source and using hydrophobic *n*-alkanes, anthracene, phenanthrene, and pyrene as the carbon source, respectively (Deng et al., 2014). In this study, NH_4_NO_3_ was employed as the sole nitrogen source to investigate the utilization of water-soluble carbon sources. The results showed that active growth was obtained using citric acid or pyruvic acid as the sole carbon source, respectively (**Figures 2A,C**). When the concentration of carbon sources was set at 40 g/L, the OD_600_ values (after the time point of 4 h) of the glucose- or lactose-treated group were a little higher than those of other groups, except the citric acid- or pyruvic acid-treated group (**Figure 2B**). However, when the concentration was set at 20 g/L, none of the carbon sources, other than citric acid and pyruvic acid, could be efficiently utilized (**Figures 2C,D**). The inefficient utilization of lactose in strain HZ01 is consistent with the property of non-lactose fermenting strains of *Achromobacter xylosoxidans* (De Baets et al., 2007). The limited carbon source range was similar to the inability of hydrocarbon-degrading *Alcanivorax borkumensis* SK2 to use common carbohydrates for growth (Schneiker et al., 2006), while *Celeribacter indicus* P73^T^ is capable of utilizing many sugars (Cao et al., 2015).

A total of 430 genes in strain HZ01 were assigned to the “carbohydrate metabolism” pathway by KAAS annotation (Supplementary Table S4). Specifically, abundant genes were enriched in “pyruvate metabolism,” which might explain the bacterial efficient utilization of pyruvic acid (**Figures 2A,C**). An intact citrate cycle (TCA cycle; Supplementary Table S4 and Figure S4) pathway was identified in strain HZ01, which was consistent with its capability to efficiently utilize citric acid (**Figures 2A,C**). Although most enzymes involved in the “glycolysis/gluconeogenesis” pathway were identified in strain HZ01, genes coding for hexokinase and its homologs were not included in the genome. The hexokinase is a key enzyme of glycolysis and catalyzes the irreversible phosphorylation of glucose, mannose, and fructose. In addition to the lack of hexokinase, fructokinase was also not identified. Thus, fructose cannot be converted to fructose-6-phosphate in strain HZ01. Besides, the gene coding for 6-phosphofructokinase-1, another key enzyme that converted fructose-6-phosphate to 1,6-fructose-biphosphate, was not identified in the genome (Supplementary Figure S5). This feature was similar to that of hydrocarbon-degrading *Polymorphum gilvum* SL003B-26A1^T^ (Nie et al., 2012). In strain SL003B-26A1^T^, the lack of 6-phosphofructokinase-1 was complemented by the pentose phosphate (PP) pathway to generate glyceraldehyde-3-phosphate that subsequently involved in the downstream steps of glycolysis. A total of 26 genes in strain HZ01 were annotated as the enzymes involved in the PP pathway (Supplementary Table S4), however, several important enzymes, such as glucose-6-phosphate dehydrogenase and 6-phosphogluconolactonase, were not present in this bacterium (Supplementary Figure S6). Thus, strain HZ01 cannot convert glucose to glyceraldehyde-3-phosphate through the intermediate product 1,6-fructose-biphosphate or via the PP pathway. Several oil-degrading microorganisms, such as *Geobacillus thermodenitrificans* NG80-2 and *Desulfatibacillum alkenivorans* AK-01, harbor a comparatively complete glycolysis pathway through the 1,6-fructose-biphosphate (Feng et al., 2007; Callaghan et al., 2012). Hydrocarbon-degrading *Celeribacter indicus* P73^T^ contains all the enzymes (except glucose-6-phosphatase) needed in glycolysis/gluconeogenesis, as well as all the enzymes required for the PP pathway and the TCA cycle (Cao et al., 2015). These evidences indicate the differences of carbohydrate metabolism among oil-degrading bacteria. Additionally, strain HZ01 lacks lactose permease and β-galactosidase for the metabolism of lactose. Galactose cannot be processed by glycolysis in this strain partially due to its lack of two essential enzymes (galactokinase and galactose-1-phosphate uridylyltransferase), which convert the galactose to glucose-1-phosphate. The absence of beta-fructofuranosidase and mannitol dehydrogenase might explain the inefficient utilization of sucrose and mannitol, respectively.

Genome annotation suggests that carbohydrate transporters in strain HZ01 are relatively rare (Supplementary Table S5), which may be another reason explaining its inability to utilize several common carbohydrates as the sole carbon source for growth (**Figure 2**). For instance, strain HZ01 lacks FrcB, FrcC, and FrcA for fructose transport. The glucose/arabinose transport system, consisting of GlcS, GlcU, GlcT, and GlcV, are not present in the genome. Among the proteins SmoE, SmoF, SmoG, and SmoK for sorbitol/mannitol transport, SmoG is not contained in strain HZ01. The sucrose-specific IIA component for sucrose uptake is absent. The LacE, LacF, LacG, and LacK for lactose/L-arabinose transport were also not identified. Among the proteins MalE, MalF, MalG, and MalK for maltose/maltodextrin transport, only MalK was identified (Supplementary Table S5).

In summary, lacking some important enzymes and carbohydrate transporters in strain HZ01 is in accord with its inability to efficiently utilize several common carbohydrates for growth, such as glucose, fructose, mannose, galactose, lactose, sucrose, and mannitol (**Figure 2**). The inefficient utilization of these compounds is consistent with the low carbohydrate availability in the marine environment where strain HZ01 was isolated (Nie et al., 2012; Deng et al., 2014). This genome feature may result from the long-term evolution for adaptation to its environment. Although strain HZ01 is not capable of utilizing some common carbohydrates, it can use hydrocarbons as carbon sources (Deng et al., 2014).

### Genetic Basis for Petroleum Degradation

#### Emulsification of Biosurfactants

Biosurfactants, which are mainly derived from the secondary metabolites of microorganisms (Nie et al., 2012), contain hydrophilic and hydrophobic groups in their structure. They harbor surface activities and emulsification activities that increase the bioavailability and degradation rates of hydrophobic substrates, including hydrocarbons (Desai and Banat, 1997). They are a structurally diverse class of compounds, with glycolipids and lipopeptides reported as two primary isolated families. So far, researches regarding the effects of biosurfactants on the bioremediation properties of *Achromobacter* strains are scarce. It has been demonstrated that surfactants SDS, Tween 60 and Tween 80 (derived from chemical processes) can enhance the detoxification of chromium-containing slag by *Achromobacter* sp. CH-1 (Chai et al., 2009).

**FIGURE 3:**
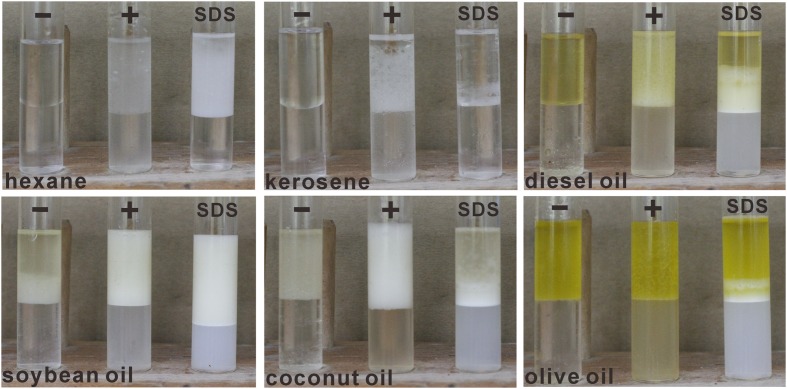
Emulsification activity of the fermentation broth using citric acid as the sole source of carbon and energy. The cell-free supernatant of the fermentation broth was used in the experiments. SDS at the concentration of 0.5 g/L was used as a positive control. -, fermentation medium without bacteria inoculation incubated for 3 days; +, incubation for 3 days with bacteria inoculation.

Strain HZ01 is capable of producing biosurfactants that efficiently emulsify diverse hydrophobic compounds, exhibiting the potential for bioremediation (Deng et al., 2016). It has been demonstrated that citric acid is related to biosurfactant production (Pirog et al., 2008). In this work, the emulsification activities of the culture broth using citric acid as the sole carbon source were investigated. Although the fermentation broth was not concentrated, stable emulsions were formed using soybean oil, coconut oil, diesel oil, kerosene, and hexane as emulsion substrates, respectively (**Figure 3**), indicating that biosurfactants might exist in the fermentation broth. Further studies are needed to confirm the biosurfactant production in strain HZ01 using citric acid as the sole carbon source. Diesel oil and kerosene, both derived from petroleum, contain diverse hydrocarbons. The emulsification of diesel oil, kerosene, and hexane suggests that the fermentation products produced by strain HZ01 may promote its utilization of hydrocarbons.

Genome annotation revealed that a total of 197 genes were assigned to “secondary metabolites biosynthesis, transport, and catabolism” (Supplementary Table S6). The genome harbors candidate genes associated with biosurfactant production, such as 3-oxoacyl-ACP reductase, acyltransferase, phosphomannomutase, and glycosyltransferase (Supplementary Table S7), which are essential for glycolipid synthesis (Maier and Soberon-Chavez, 2000). Eleven genes were annotated as LuxR family transcriptional regulators that play an important role in the regulation of glycolipid synthesis (Satpute et al., 2010). Genes coding for cell envelope biogenesis protein OmpA (gene_2469) and outer membrane protein OmpAb (gene_3528) were identified. OmpA is an active constituent of alasan (a bioemulsifier) and has been demonstrated to be involved in the degradation of hydrophobic compounds, including hydrocarbons (Walzer et al., 2006). OmpH (gene_1336), which is possibly related to bioemulsifier production (Schneiker et al., 2006), is also present in strain HZ01. Generally, lipopeptides are synthesized through the ribosome-independent pathway using non-ribosomal peptide synthetases (NRPSs) (Roongsawang et al., 2010). One gene (gene_1007) was assigned to “non-ribosomal peptide structures.” Additionally, thioesterases and peptide synthetases, both related to lipopeptide biosynthesis (Yao et al., 2003; Moyne et al., 2004), were also identified (Supplementary Table S7). Strain HZ01 was previously reported to produce a cyclic lipopeptide containing the peptide chain of Gly-Gly-Leu-Met-Leu-Leu linked to a C_21_ fatty acid chain (Deng et al., 2016). We infer that the biosynthetic pathway of this biosurfactant includes three major processes (Supplementary Figure S7), i.e., (a) a 3-hydroxy-heneicosanoic acid is derived from specific carbon sources via fatty acid biosynthesis; (b) the hexapeptide is obtained by a series of enzymatic condensations from the N-terminal of Leu to the C-terminal of Gly; and (c) the 3-hydroxy-heneicosanoic acid may undergo an enzymatic condensation process, being incorporated at the C- and N-terminals of the hexapeptide to produce a cyclic lipopeptide. Further studies are needed to clarify this biosynthetic pathway.

In summary, the identified genes provide a genetic basis for biosurfactant production in strain HZ01, which may facilitate its uptake and degradation of petroleum hydrocarbons.

#### Degradation Pathways of *n*-Alkanes

Petroleum is a complex mixture primarily containing saturated hydrocarbons, PAHs and asphaltenes, of which alkanes are its major constituents (Rojo, 2009). Strain HZ01 is capable of degrading C_12_–C_27_ *n*-alkanes and PAHs (Deng et al., 2014). Its genome contains numerous genes for hydrocarbon degradation. Methyl-accepting chemotaxis proteins (MCP; gene_441, gene_1384, gene_1386, gene_1387, and gene_2249), related to alkane chemotaxis (Smits et al., 2003), may be beneficial for strain HZ01 to seek hydrocarbons as substrates. Cytochrome *o* ubiquinol oxidase (Cyo; gene_2136, gene_2137, gene_2138, gene_2181, gene_2182, gene_2183, and gene_2184) exhibits a negative regulation over the expression of alkane degradation genes, and there is a marked correlation between the Cyo levels and the repression extents of alkane degradation pathway (Dinamarca et al., 2003). Transcriptome sequencing showed that the transcriptional level of Cyo in strain HZ01 was not affected during petroleum degradation (Hong et al., 2016). The Cyo is not reported in hydrocarbon-degrading *Alcanivorax borkumensis* SK2 (Schneiker et al., 2006), *Oleispira antarctica* RB-8 (Kube et al., 2013), *Geobacillus thermodenitrificans* NG80-2 (Feng et al., 2007), *Polymorphum gilvum* SL003B-26A1^T^ (Nie et al., 2012), or *Desulfatibacillum alkenivorans* AK-01 (Callaghan et al., 2012).

*n*-Alkanes are usually subjected to degradation via the terminal or subterminal oxidation pathway (Rojo, 2009). In the terminal oxidation pathway, alkane degradation is initiated by oxygenation of a terminal methyl group to generate a primary alcohol, which is converted into its corresponding aldehyde and is further oxidized to a fatty acid. Subsequently, the fatty acid combines with CoA and is subjected to β-oxidation to produce acetyl-CoA. The acetyl-CoA is further processed by the TCA cycle to finally generate H_2_O, CO_2_, and energy. Diverse enzymes involved in this process were identified in strain HZ01, such as oxygenases, hydroxylases, alcohol dehydrogenases, aldehyde dehydrogenases, and acyl-CoA synthetases (Supplementary Table S8). Representative alkane hydroxylase AlkB and its homologs that initiated the oxidation of medium-chain alkanes were not identified in the genome, which was consistent with the genome features of oil-degrading *Geobacillus thermodenitrificans* NG80-2 (Feng et al., 2007) and *Polymorphum gilvum* SL003B-26A1^T^ (Nie et al., 2012). The lack of AlkB and its homologs in hydrocarbon-degrading marine bacteria is not uncommon (Venter et al., 2004). Some bacteria use the enzymes of cytochrome P450 family to perform similar functions as that of AlkB and its homologs (Rojo, 2009). One gene coding for cytochrome P450 (gene_1029) in strain HZ01 was identified. Its sequence showed an identity of 60% to the sequence of cytochrome P450 monooxygenase in *Pseudomonas* sp. Lz4W (GenBank accession no. EMI05095.1), suggesting that the cytochrome P450 of strain HZ01 may have similar functions as that of strain Lz4W. RNA-seq demonstrated that two transcripts of cytochrome P450 were up-regulated after petroleum treatment (Hong et al., 2016). Additionally, ferredoxin and ferredoxin reductase, required for the electron transfer from NAD(P)H to cytochrome P450, were also present in the genome (Supplementary Table S8), contributing to the alkane oxidation. Thus, the cytochrome P450 may play an important role in the initial oxidation of *n*-alkanes in strain HZ01. Comparative analysis revealed that, although strains NG80-2 and SL003B-26A1^T^ lacked AlkB and its homologs, long-chain alkane monooxygenase LadA was included in their genomes for alkane degradation (Feng et al., 2007; Nie et al., 2012). LadA belongs to the bacterial luciferase family and plays an important role in the initial oxidation of C_15_–C_36_ *n*-alkanes (Feng et al., 2007). On the contrary, *Alcanivorax borkumensis* SK2 and *Oleispira antarctica* RB-8 contained AlkB and its homologs, as well as cytochrome P450, whereas LadA was not identified (Schneiker et al., 2006; Kube et al., 2013). LadA was also not identified in strain HZ01 by genome sequencing. Nevertheless, RNA-seq showed that strain HZ01 contained one transcript coding for luciferase-like monooxygenase family protein 1 (Hong et al., 2016), which might have the functions similar to those of LadA.

Fatty acids are the major intermediate products of alkane degradation and are processed by β-oxidation to generate acetyl-CoA (Rojo, 2009). Besides acyl-CoA synthetase, which catalyzes the conjugation of fatty acid to CoA, fatty acid hydroxylase (gene_696, gene_1843, and gene_2856) is also contained in strain HZ01. The fatty acid hydroxylase catalyzes the hydroxylation of fatty acids, generating hydroxy fatty acids. Subsequently, the hydroxy fatty acids are converted into dicarboxylic acids, followed by β-oxidation (Coon, 2005). A total of 40 genes in strain HZ01 were assigned to the “fatty acid degradation” pathway, including the genes related to β-oxidation (Supplementary Table S9 and Figure S8). RNA-seq demonstrated that the genes involved in β-oxidation were activated after petroleum treatment (Hong et al., 2016). In addition, an intact TCA cycle pathway for processing the resulting acetyl-CoA is present in strain HZ01 (Supplementary Figure S4).

**FIGURE 4:**
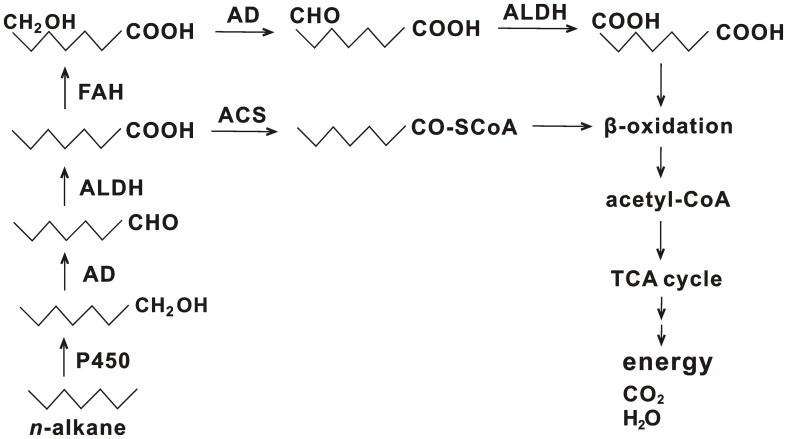
Terminal oxidation pathway for *n*-alkane degradation in *Achromobacter* sp. HZ01. P450, cytochrome P450; AD, alcohol dehydrogenase; ALDH, aldehyde dehydrogenase; ACS, acyl-CoA synthetase; FAH, fatty acid hydroxylase; TCA cycle, citrate cycle.

A total of 35 genes were assigned to the “methane metabolism” pathway (Supplementary Table S10). For instance, strain HZ01 contained *fdoG*, *fdfH*, *fdoH*, and *fdoI* coding for formate dehydrogenase subunits. However, some enzymes essential for methane oxidation were not identified, including methane monooxygenase, methanol dehydrogenase, and formaldehyde dehydrogenase. Strain HZ01 contains esterases, but the Baeyer-Villiger monooxygenase, an essential enzyme involved in the cycloalkane degradation and the subterminal oxidation of *n*-alkanes, was not included in the genome. The identified genes suggest that (i) subterminal oxidation pathway is not present in strain HZ01; (ii) this bacterium cannot perform methane oxidation or cycloalkane degradation; and that (iii) the degradation of *n*-alkanes in strain HZ01 is performed via the terminal oxidation pathway (**Figure 4**). The results of genome sequencing further verify the pathway for *n*-alkane degradation predicted by RNA-seq (Hong et al., 2016). The metabolic reaction initiated by fatty acid hydroxylase (**Figure 4**) was not included in the previous predicted pathway. Besides, RNA-seq showed that the following steps of terminal oxidation pathway were activated after strain HZ01 was treated with petroleum for 16 h: (i) the initial oxidation of *n*-alkanes; (ii) reactions catalyzed by dehydrogenases and acyl-CoA synthases; and (iii) fatty acid β-oxidation (Hong et al., 2016).

#### Degradation Pathways of Aromatic Compounds

In the previous RNA-seq, some transcripts related to the degradation of aromatic compounds were identified in strain HZ01 (Hong et al., 2016). Nevertheless, the potential pathways for degradation of aromatic compounds in this bacterium have not been revealed so far.

Genome sequencing of this study showed that abundant genes related to the degradation of aromatic compounds were contained in strain HZ01. For instance, a total of 28 genes were assigned to “degradation of aromatic compounds” (Supplementary Table S11). During the degradation of aromatic compounds, reactive dihydroxylated intermediates, such as catechol and protocatechuate, will be produced. These intermediates are further degraded via the intradiol (*ortho*) or extradiol (*meta*) ring cleavage (Strachan et al., 1998). The resulting products of the *ortho*-pathway (acetyl-CoA and succinyl-CoA) and the *meta*-pathway (pyruvate and acetaldehyde) are further processed by the TCA cycle (Pessione et al., 1999).

**FIGURE 5:**
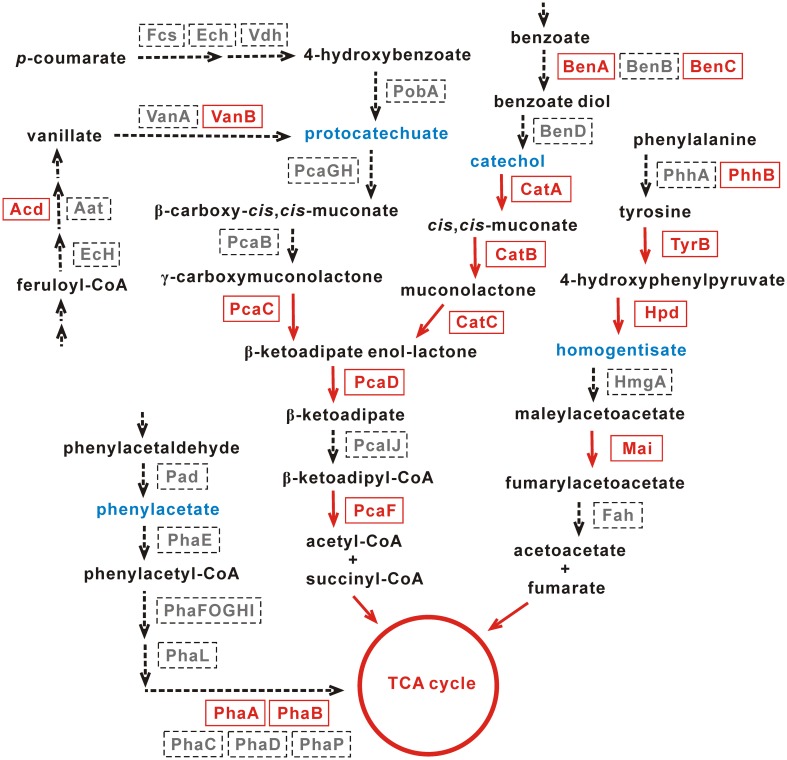
Pathways for the degradation of aromatic compounds in *Achromobacter* sp. HZ01. The enzymes present and absent in strain HZ01 are shown in red fonts and gray fonts, respectively. The red arrows indicate that strain HZ01 contains corresponding metabolic steps. The dotted arrows show the lacking steps in strain HZ01. Blue fonts represent the key intermediate products. Not all the upper-stream steps are shown in the figure.

The homogentisate pathway, the protocatechuate and catechol branches of the β-ketoadipate pathway, and the phenylacetate pathway are four common pathways for the degradation of aromatic compounds (Jimenez et al., 2002). The genome sequencing of this work showed that only PhaA and PhaB, both belonging to the enoyl-CoA hydratase/isomerase family proteins, were identified in the phenylacetate pathway (**Figure 5**).

Protocatechuate and catechol are representative intermediates of the protocatechuate and catechol branches of the β-ketoadipate pathway, respectively, and these two intermediates are generally produced during the degradation of aromatic compounds. For instance, the catechol intermediate may be generated from benzoate, benzamide, (chloro)-biphenyl, benzonitrile, benzaldehyde, salicylate, and mandelate (Chain et al., 2006). Both the protocatechuate and catechol branches will produce β-ketoadipate enol-lactone, which is sequentially processed by PcaD (3-oxoadipate enol-lactonase; gene_4113), PcaIJ (not identified) and PcaF (beta-ketoadipyl CoA thiolase; gene_443). Then, acetyl-CoA and succinyl-CoA are generated and further metabolized by TCA cycle (Jimenez et al., 2002). PcaC (4-carboxymuconolactone decarboxylase; gene_4173) catalyzed the production of β-ketoadipate enol-lactone from γ-carboxymuconolactone and was identified in the protocatechuate branch (**Figure 5**). Nevertheless, PcaGH and PcaB were not identified. In the upper-stream of the protocatechuate branch, Acd (acyl-CoA dehydrogenase) and VanB (vanillate monooxygenase ferredoxin subunit) are present in strain HZ01. Acd and VanB play important roles in the production of vanillate from feruloyl-CoA and protocatechuate from vanillate, respectively.

So far, some *Achromobacter* strains capable of degrading aromatic compounds have been reported. Catechol dioxygenases play an important role in the degradation of aromatic compounds (Moon et al., 1997). Similar to strain HZ01, *Achromobacter* sp. DMS1 and *Achromobacter xylosoxidans* DN002 contain catechol 1,2-dioxygenase (Supplementary Table S12). *Achromobacter xylosoxidans* KF701, strain DN002 and *Achromobacter xylosoxidans* T7 contain catechol 2,3-dioxygenase, which is not found in strain HZ01. The catechol branch of the β-ketoadipate pathway is comparatively intact than the protocatechuate branch in strain HZ01. CatA (catechol 1,2-dioxygenase; gene_376) performs the intradiol cleavage of catechol and some of its derivatives. This enzyme also catalyzes the extradiol cleavage of *meta*-substituted substrates, such as 3-methoxycatechol and 3-methylcatechol (Strachan et al., 1998). Within the catechol branch, CatA transforms catechol into *cis*,*cis*-muconate, which is processed by CatB (muconate cycloisomerase; gene_772) to generate muconolactone (**Figure 5**). Then, CatC (muconolactone D-isomerase; gene_4111) catalyzes muconolactone to produce β-ketoadipate enol-lactone. In the upper-stream of catechol, BenA (benzoate/toluate 1,2-dioxygenase subunit alpha; gene_3355 and gene_3376) and BenC (benzoate/toluate 1,2-dioxygenase reductase component; gene_2862) were identified, but BenB and BenD were not present. BenABC can transform benzoate into benzoate diol, which is further metabolized by BenD to generate catechol (**Figure 5**).

Regarding the homogentisate pathway, only Mai (maleylacetoacetate isomerase; gene_2657 and gene_5130) was identified in the downstream of homogentisate. This enzyme catalyzes maleylacetoacetate to generate fumarylacetoacetate (**Figure 5**). In the upper-stream of homogentisate, PhhB (pterin-4-alpha-carbinolamine dehydratase; gene_3287), TyrB (aromatic amino acid aminotransferase; gene_533) and Hpd (4-hydroxyphenylpyruvate dioxygenase; gene_3903) were identified, but PhhA was not found. PhhA and PhhB play an important role in the transformation of phenylalanine into tyrosine (Jimenez et al., 2002). Then, the tyrosine is catalyzed by TyrB to generate 4-hydroxyphenylpyruvate, which is transformed into homogentisate by Hpd (**Figure 5**).

Additionally, the genes coding for benzoate/toluate 1,2-dioxygenase subunit alpha (gene_3355 and gene_3376) and benzoate/toluate 1,2-dioxygenase reductase component (gene_2862) were identified in strain HZ01. RNA-seq showed that the expression levels of benzoate 1,2-dioxygenase subunit alpha and benzoate 1,2-dioxygenase large subunit were not affected by petroleum treatment (Hong et al., 2016). The benzoate 1,2-dioxygenase system performs its functions via catalyzing the double hydroxylation of benzoates with the incorporation of two oxygen atoms (Yamaguchi and Fujisawa, 1980). Salicylate hydroxylase (gene_2661), a key enzyme in naphthalene catabolism, catalyzes the decarboxylation and hydroxylation of salicylate, generating catechol, H_2_O, and CO_2_ (You et al., 1991). Glutathione *S*-transferase (GST), related to PAHs degradation (Xia and Min, 2003), were also identified in strain HZ01 (Supplementary Table S8). Laccases (gene_3832), a family of blue multicopper oxidases, play an important role in the oxidation of numerous aromatic compounds (Fang et al., 2011). In addition, 150 genes in strain HZ01 were assigned to “xenobiotics biodegradation and metabolism” using KAAS annotation (Supplementary Table S13), such as genes related to the degradation of fluorobenzoate, toluene, xylene, nitrotoluene, and dioxin. Several genes encoding GntR family transcriptional regulators and MarR family transcriptional regulators related to aromatic compound degradation were also identified.

Strain HZ01 has been reported to degrade anthracene, phenanthrene, and pyrene (Deng et al., 2014). The genome sequencing in this study further suggests that strain HZ01 has the potential for bioremediation of aromatic-compounds-contaminated environments. Since the enzymes involved in the catechol pathway are comparatively complete, this pathway may play a major role in the degradation of aromatic compounds in strain HZ01. Further studies are needed to elucidate the aromatic compounds degradation in this bacterium.

Unsaturated fatty acids are related to the fluidity of cell membrane and contribute to the adaptation of bacteria to low-temperature environments and the low solubility of substrates (including hydrocarbons). A total of 12 genes in the genome of strain HZ01 were assigned to “biosynthesis of unsaturated fatty acids.” For instance, gene_4447 encodes fatty acid desaturase, which is existent in almost all organisms and is a key enzyme in the biosynthesis of fatty acids (Alonso et al., 2003). These genes play important roles in the fatty acid synthesis in strain HZ01 and may enhance its capability for hydrocarbon degradation. Other candidate genes associated with the degradation of petroleum components were shown in Supplementary Table S8, mainly including the genes coding for dehydrogenases, rubredoxins, oxidoreductases, monooxygenases, and dioxygenases. In summary, the identified genes and pathways provide a genetic basis for petroleum degradation in strain HZ01.

**FIGURE 6:**
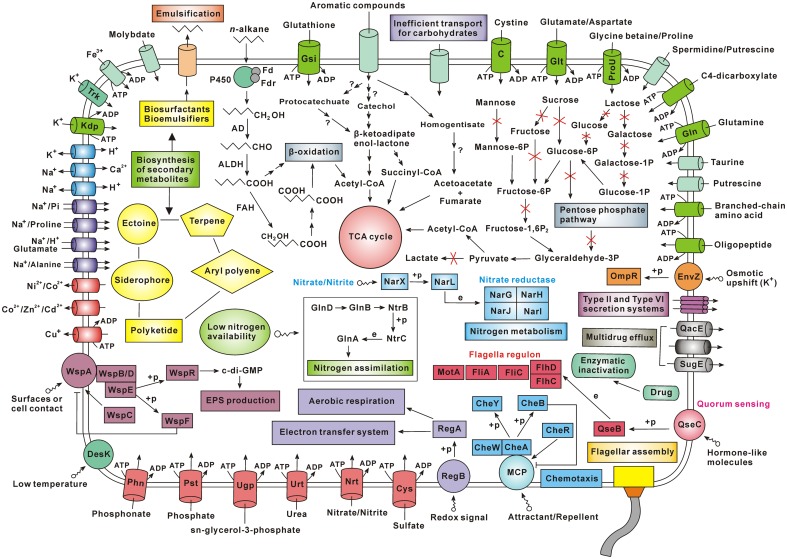
Schematic overview of the metabolism and material transport in *Achromobacter* sp. HZ01. Primary information: (i) the *n*-alkane degradation in strain HZ01 is performed via the terminal oxidation pathway, and the catechol pathway may play a major role in the degradation of aromatic compounds; (ii) the inefficient carbohydrate transport and the lack of some key enzymes account for the bacterial inability to utilize several common carbohydrates for growth; (iii) strain HZ01 harbors the genes related to biosynthesis of secondary metabolites; (iv) the membrane transporters are essential for nutrient uptake and substance export; (v) strain HZ01 contains important two-component systems for the responses to environmental changes; (vi) the chemotaxis and flagellar assembly are beneficial for pursuing nutrients and avoiding environmental damages; (vii) besides through the enzymatic inactivation of antibiotics, strain HZ01 contains efflux pumps for elimination of antibiotics. Not all the metabolic pathways and transporters are shown in the figure. Import or export of solutes is indicated by the direction of the arrow through the transporter. The arrow with a red X shape indicates that the metabolic step is absent. The arrow with a question mark indicates that it is unsure whether the corresponding step can be completed. Abbreviations: Fd, ferredoxin; Fdr, ferredoxin reductase; P450, cytochrome P450; AD, alcohol dehydrogenase; ALDH, aldehyde dehydrogenase; FAH, fatty acid hydroxylase; +p, phosphorylation; e, expression; Trk, potassium transporter Trk; Kdp, Kdp system for potassium transport; EPS, extracellular polysaccharide; Phn, phosphonate transport system; Pst, phosphate transport system; Ugp, sn-glycerol-3-phosphate transport system; Urt, urea transport system; Nrt, nitrate/nitrite transport system; Cys, sulfate transport system; MCP, methyl-accepting chemotaxis protein; Gln, glutamine transport system; ProU, glycine betaine/proline transport system; Glt, glutamate/aspartate transport system; C, cystine transport system; Gsi, glutathione transport system.

Besides, the analysis contents of this study also include (i) genomic comparison, (ii) amino acid metabolism, (iii) nitrogen metabolism, (iv) biosynthesis of other secondary metabolites, (v) antibiotic resistance, (vi) two-component systems, (vii) cell motility, and (viii) membrane transport (Supplementary Results and Discussion). According to the genes identified by genome sequencing, the material transport and metabolic pathways in *Achromobacter* sp. HZ01 were depicted in **Figure 6**.

## Discussion

It has been demonstrated that *Achromobacter* sp. HZ01 is capable of degrading petroleum hydrocarbons and adapting to a wide range of salinity (Deng et al., 2014). The genome analysis of this study verifies its genetic basis for these properties (see the results above and Supplementary Results), which further indicates that strain HZ01 has a good potential for bioremediation of crude oil-polluted marine environments. *Achromobacter* strains are representative bacteria with bioremediation potential (Supplementary Table S12), and some of their degradation properties have been analyzed (Furukawa et al., 1989; Cai et al., 2009). Among the reported *Achromobacter* strains, *Achromobacter xylosoxidans* XL and *Achromobacter* sp. 4(2010) have some similarities with strain HZ01 in alkane degradation. Strain XL is capable of degrading C_12_–C_23_ and C_27_–C_43_ alkanes (Si et al., 2011), and strain 4(2010) can degrade diesel oil (Kaczorek et al., 2013), an oil mixture containing diverse hydrocarbons. However, their pathways for alkane degradation have not been determined. To our knowledge, genome-wide researches regarding the bioremediation properties of *Achromobacter* strains are very rare (Supplementary Table S12). Our work is the first genome analysis systematically elucidating the pathways of hydrocarbon degradation in bacterial strains belonging to the genus *Achromobacter*. The obtained data expand our insights into the bioremediation properties of *Achromobacter* strains. It also provides valuable genome information for developing a cost-effective and eco-friendly method for bioremediation. For instance, the genome sequencing indicates that strain HZ01 lacks long-chain alkane monooxygenase LadA, which is related to the degradation of C_15_–C_36_ alkanes (Feng et al., 2007). Although strain HZ01 is capable of degrading C_12_–C_27_ *n*-alkanes (Deng et al., 2014), cloning the LadA into this bacterium may enhance its capability for *n*-alkane degradation. In other words, based on comprehensively understanding the genetic background of strain HZ01, gene engineering technique may be employed to improve its hereditary features, which will undoubtedly enhance its possibility for practical applications in bioremediation.

In summary, the genome sequencing and genome-based functional analysis of *Achromobacter* sp. HZ01 provide us with deep insights into its genetic basis for (i) major metabolisms, (ii) petroleum degradation, (iii) biosynthesis of secondary metabolites, (iv) antibiotic resistance, (v) bacterial responses to environmental changes, (vi) cell motility, and (vii) material transport and secretion. The obtained genome data contribute to developing rational strategies for bioremediation of petroleum-polluted marine environments. The results also provide a valuable reference for the genomics, transcriptomics and proteomics of other microorganisms.

## Author Contributions

Y-HH, J-HW, and HD designed the experiments. Y-HH, C-CY, and Q-ZZ performed the experiments. Y-HH, J-HW, HD, X-YW, J-PY, and JP interpreted the experimental data. Y-HH and J-HW wrote the manuscript. Y-HH, J-HW, and HD revised the manuscript.

## Conflict of Interest Statement

The authors declare that the research was conducted in the absence of any commercial or financial relationships that could be construed as a potential conflict of interest.

## Footnotes

1 http://www.ncbi.nlm.nih.gov/

2 http://www.genome.jp/kegg/
