# Supplementary material for: Genome Sequencing Reveals the Potential of Achromobacter sp. HZ01 for Bioremediation
Source: Front Microbiol. 2017 Aug 9;8:1507. doi: 10.3389/fmicb.2017.01507 (PMC5552670; doi:10.3389/fmicb.2017.01507)
Supplement: Supplementary file 1 [file Table_1.DOC]

***Supplementary Tables***

**Genome Sequencing Reveals the Potential of *Achromobacter* sp. HZ01 for Bioremediation**

**Yue-Hui Hong^1^,** **Cong-Cong Ye^1^, Qian-Zhi Zhou^1^, Xiao-Ying Wu^2^, Jian-Ping Yuan^1^, Juan Peng^1^, Hailin Deng^1^,*, Jiang-Hai Wang^1^,***

^1^ Guangdong Provincial Key Laboratory of Marine Resources and Coastal Engineering/South China Sea Bioresource Exploitation and Utilization Collaborative Innovation Center, School of Marine Sciences, Sun Yat-Sen University, Guangzhou, People’s Republic of China

^2^ State Key Laboratory of Conservation and Utilization of Subtropical Agro-Bioresources, College of Natural Resources and Environment, South China Agricultural University, Guangzhou, People’s Republic of China

***Correspondence:**

Jiang-Hai Wang

wangjhai@mail.sysu.edu.cn

Hailin Deng

denghlin3@mail.sysu.edu.cn

**Table S1 Reads generation of genome sequencing and quality control.**

| Library | Insert length | Raw reads | |  | Quality reads | | Removal rate (%) |
| --- | --- | --- | --- | --- | --- | --- | --- |
|  |  | Total bases (Mbp) | Read pairs (M)^a^ |  | Total bases (Mbp) | Read pairs (M)^a^ |  |
|  |  |  |  |  |  |  |  |
| A | 500 bp | 936 | 1.87 |  | 908 | 1.87 | 3.0 |
| B | 800 bp | 670 | 1.34 |  | 642 | 1.34 | 4.2 |

^a^Two decimal places of the numbers were shown.

**Table S2** **Summary of gene annotation.**

| Database | Annotated genes |
| --- | --- |
| NCBI-nr | 5,081 (98.4%) |
| KEGG | 4,929 (95.5%) |
| eggNOG | 4,927 (95.4%) |

The number predicted genes amounted to 5,162. The annotated gene proportion were shown in the parentheses.

**Table S3** **Gene abundances and comparison of COG categories.**

| COG category | Annotated genes in strain HZ01 in each COG category | Total annotated genes in strain HZ01 | Annotated genes in database in each COG category | Total annotated genes in database | Odds ratio | P-value | P.adjust |
| --- | --- | --- | --- | --- | --- | --- | --- |
| [E] Amino acid transport and metabolism | 661 | 4962 | 5499902 | 59858600 | 0.371444516 | 1.75E-21 | 3.68E-20 |
| [R] General function prediction only | 562 | 4962 | 6973258 | 59858600 | -0.02815975 | 0.492765085 | 0.517403339 |
| [K] Transcription | 484 | 4962 | 4621252 | 59858600 | 0.233840193 | 2.09E-07 | 6.27E-07 |
| [S] Function unknown | 469 | 4962 | 5346904 | 59858600 | 0.056506017 | 0.204255735 | 0.238298358 |
| [P] Inorganic ion transport and metabolism | 358 | 4962 | 3351134 | 59858600 | 0.25365514 | 1.92E-06 | 4.03E-06 |
| [C] Energy production and conversion | 328 | 4962 | 3612896 | 59858600 | 0.090924892 | 0.0893352 | 0.11725245 |
| [I] Lipid transport and metabolism | 275 | 4962 | 2257429 | 59858600 | 0.384965493 | 8.64E-10 | 3.02E-09 |
| [G] Carbohydrate transport and metabolism | 274 | 4962 | 4384381 | 59858600 | -0.2824994 | 4.51E-07 | 1.18E-06 |
| [M] Cell wall/membrane/envelope biogenesis | 255 | 4962 | 3221559 | 59858600 | -0.04618091 | 0.469345186 | 0.517403339 |
| [T] Signal transduction mechanisms | 204 | 4962 | 2486487 | 59858600 | -0.01032993 | 0.915003502 | 0.915003502 |
| [Q] Secondary metabolites biosynthesis, transport, and catabolism | 197 | 4962 | 1427255 | 59858600 | 0.50987166 | 2.13E-11 | 8.95E-11 |
| [J] Translation, ribosomal structure and biogenesis | 174 | 4962 | 3523635 | 59858600 | -0.51801688 | 2.42E-14 | 1.69E-13 |
| [H] Coenzyme transport and metabolism | 160 | 4962 | 2559457 | 59858600 | -0.28220036 | 0.000170395 | 0.0003253 |
| [O] Post-translational modification, protein turnover, and chaperones | 144 | 4962 | 2049939 | 59858600 | -0.16557579 | 0.042325061 | 0.063487592 |
| [L] Replication, recombination and repair | 120 | 4962 | 2943639 | 59858600 | -0.70973388 | 3.72E-19 | 3.91E-18 |
| [U] Intracellular trafficking, secretion, and vesicular transport | 91 | 4962 | 1357204 | 59858600 | -0.21214624 | 0.040156297 | 0.063487592 |
| [N] Cell motility | 70 | 4962 | 1040342 | 59858600 | -0.20863331 | 0.081865136 | 0.11461119 |
| [F] Nucleotide transport and metabolism | 65 | 4962 | 1630702 | 59858600 | -0.73220238 | 1.95E-11 | 8.95E-11 |
| [V] Defense mechanisms | 41 | 4962 | 978100 | 59858600 | -0.68186362 | 1.29E-06 | 3.01E-06 |
| [D] Cell cycle control, cell division, chromosome partitioning | 27 | 4962 | 578110 | 59858600 | -0.57375107 | 0.001354909 | 0.002371091 |
| [B] Chromatin structure and dynamics | 3 | 4962 | 15015 | 59858600 | 0.879738816 | 0.130346402 | 0.161016144 |

**Table S4** **Genes assigned to the “carbohydrate metabolism” pathway.**

| Pathway | Predicted gene | Entry/Gene name | | Enzyme/Protein |
| --- | --- | --- | --- | --- |
| 00010 Glycolysis / Gluconeogenesis [PATH:ko00010] | | | | |
|  | scaffold00001_gene_136 | K01810 GPI, pgi | | glucose-6-phosphate isomerase [EC:5.3.1.9] |
|  | scaffold00002_gene_2380 | K01810 GPI, pgi | | glucose-6-phosphate isomerase [EC:5.3.1.9] |
|  | scaffold00005_gene_4632 | K03841 FBP, fbp | | fructose-1,6-bisphosphatase I [EC:3.1.3.11] |
|  | scaffold00002_gene_1952 | K01624 FBA, fbaA | | fructose-bisphosphate aldolase, class II [EC:4.1.2.13] |
|  | scaffold00003_gene_3552 | K01803 TPI, tpiA | | triosephosphate isomerase (TIM) [EC:5.3.1.1] |
|  | scaffold00002_gene_1999 | K00134 GAPDH, gapA | | glyceraldehyde 3-phosphate dehydrogenase [EC:1.2.1.12] |
|  | scaffold00002_gene_1998 | K00927 PGK, pgk | | phosphoglycerate kinase [EC:2.7.2.3] |
|  | scaffold00004_gene_4211 | K01834 PGAM, gpmA | | 2,3-bisphosphoglycerate-dependent phosphoglycerate mutase [EC:5.4.2.11] |
|  | scaffold00002_gene_1881 | K15634 gpmB | | probable phosphoglycerate mutase [EC:5.4.2.12] |
|  | scaffold00003_gene_3784 | K01689 ENO, eno | | enolase [EC:4.2.1.11] |
|  | scaffold00003_gene_3295 | K00873 PK, pyk | | pyruvate kinase [EC:2.7.1.40] |
|  | scaffold00001_gene_1422 | K00163 aceE | | pyruvate dehydrogenase E1 component [EC:1.2.4.1] |
|  | scaffold00003_gene_3813 | K00163 aceE | | pyruvate dehydrogenase E1 component [EC:1.2.4.1] |
|  | scaffold00001_gene_1421 | K00627 DLAT, aceF, pdhC | | pyruvate dehydrogenase E2 component (dihydrolipoamide acetyltransferase) [EC:2.3.1.12] |
|  | scaffold00002_gene_2696 | K00627 DLAT, aceF, pdhC | | pyruvate dehydrogenase E2 component (dihydrolipoamide acetyltransferase) [EC:2.3.1.12] |
|  | scaffold00001_gene_893 | K00382 DLD, lpd, pdhD | | dihydrolipoamide dehydrogenase [EC:1.8.1.4] |
|  | scaffold00001_gene_1420 | K00382 DLD, lpd, pdhD | | dihydrolipoamide dehydrogenase [EC:1.8.1.4] |
|  | scaffold00001_gene_1471 | K00382 DLD, lpd, pdhD | | dihydrolipoamide dehydrogenase [EC:1.8.1.4] |
|  | scaffold00002_gene_2697 | K00382 DLD, lpd, pdhD | | dihydrolipoamide dehydrogenase [EC:1.8.1.4] |
|  | scaffold00003_gene_3822 | K00382 DLD, lpd, pdhD | | dihydrolipoamide dehydrogenase [EC:1.8.1.4] |
|  | scaffold00002_gene_2882 | K00121 frmA, ADH5, adhC | | S-(hydroxymethyl)glutathione dehydrogenase / alcohol dehydrogenase [EC:1.1.1.284 1.1.1.1] |
|  | scaffold00007_gene_4951 | K00121 frmA, ADH5, adhC | | S-(hydroxymethyl)glutathione dehydrogenase / alcohol dehydrogenase [EC:1.1.1.284 1.1.1.1] |
|  | scaffold00001_gene_699 | K04072 adhE | | acetaldehyde dehydrogenase / alcohol dehydrogenase [EC:1.2.1.10 1.1.1.1] |
|  | scaffold00002_gene_1903 | K13953 adhP | | alcohol dehydrogenase, propanol-preferring [EC:1.1.1.1] |
|  | scaffold00001_gene_377 | K00128 E1.2.1.3 | | aldehyde dehydrogenase (NAD+) [EC:1.2.1.3] |
|  | scaffold00002_gene_2864 | K00128 E1.2.1.3 | | aldehyde dehydrogenase (NAD+) [EC:1.2.1.3] |
|  | scaffold00003_gene_3561 | K00128 E1.2.1.3 | | aldehyde dehydrogenase (NAD+) [EC:1.2.1.3] |
|  | scaffold00006_gene_4875 | K00128 E1.2.1.3 | | aldehyde dehydrogenase (NAD+) [EC:1.2.1.3] |
|  | scaffold00002_gene_2302 | K00149 ALDH9A1 | | aldehyde dehydrogenase family 9 member A1 [EC:1.2.1.47 1.2.1.3] |
|  | scaffold00002_gene_2849 | K00138 aldB | | aldehyde dehydrogenase [EC:1.2.1.-] |
|  | scaffold00001_gene_1650 | K01895 ACSS, acs | | acetyl-CoA synthetase [EC:6.2.1.1] |
|  | scaffold00003_gene_3762 | K01895 ACSS, acs | | acetyl-CoA synthetase [EC:6.2.1.1] |
|  | scaffold00003_gene_3789 | K01895 ACSS, acs | | acetyl-CoA synthetase [EC:6.2.1.1] |
|  | scaffold00001_gene_44 | K01785 galM, GALM | | aldose 1-epimerase [EC:5.1.3.3] |
|  | scaffold00001_gene_135 | K15778 pmm-pgm | | phosphomannomutase / phosphoglucomutase [EC:5.4.2.8 5.4.2.2] |
|  | scaffold00002_gene_2379 | K15778 pmm-pgm | | phosphomannomutase / phosphoglucomutase [EC:5.4.2.8 5.4.2.2] |
|  | scaffold00001_gene_1494 | K18978 gapN | | glyceraldehyde-3-phosphate dehydrogenase [NAD(P)+] [EC:1.2.1.90] |
|  | scaffold00001_gene_1649 | K01596 E4.1.1.32, pckA, PEPCK | | phosphoenolpyruvate carboxykinase (GTP) [EC:4.1.1.32] |
| 00020 Citrate cycle (TCA cycle) [PATH:ko00020] | | | | |
|  | scaffold00003_gene_3809 | | K01647 CS, gltA | citrate synthase [EC:2.3.3.1] |
|  | scaffold00001_gene_176 | | K01681 ACO, acnA | aconitate hydratase [EC:4.2.1.3] |
|  | scaffold00002_gene_2484 | | K01681 ACO, acnA | aconitate hydratase [EC:4.2.1.3] |
|  | scaffold00001_gene_189 | | K01682 acnB | aconitate hydratase 2 / 2-methylisocitrate dehydratase [EC:4.2.1.3 4.2.1.99] |
|  | scaffold00001_gene_1185 | | K00031 IDH1, IDH2, icd | isocitrate dehydrogenase [EC:1.1.1.42] |
|  | scaffold00003_gene_3482 | | K00031 IDH1, IDH2, icd | isocitrate dehydrogenase [EC:1.1.1.42] |
|  | scaffold00003_gene_3820 | | K00164 OGDH, sucA | 2-oxoglutarate dehydrogenase E1 component [EC:1.2.4.2] |
|  | scaffold00003_gene_3821 | | K00658 DLST, sucB | 2-oxoglutarate dehydrogenase E2 component (dihydrolipoamide succinyltransferase) [EC:2.3.1.61] |
|  | scaffold00001_gene_893 | | K00382 DLD, lpd, pdhD | dihydrolipoamide dehydrogenase [EC:1.8.1.4] |
|  | scaffold00001_gene_1420 | | K00382 DLD, lpd, pdhD | dihydrolipoamide dehydrogenase [EC:1.8.1.4] |
|  | scaffold00001_gene_1471 | | K00382 DLD, lpd, pdhD | dihydrolipoamide dehydrogenase [EC:1.8.1.4] |
|  | scaffold00002_gene_2697 | | K00382 DLD, lpd, pdhD | dihydrolipoamide dehydrogenase [EC:1.8.1.4] |
|  | scaffold00003_gene_3822 | | K00382 DLD, lpd, pdhD | dihydrolipoamide dehydrogenase [EC:1.8.1.4] |
|  | scaffold00005_gene_4614 | | K01902 sucD | succinyl-CoA synthetase alpha subunit [EC:6.2.1.5] |
|  | scaffold00005_gene_4613 | | K01903 sucC | succinyl-CoA synthetase beta subunit [EC:6.2.1.5] |
|  | scaffold00002_gene_1807 | | K18118 aarC, cat1 | succinyl-CoA:acetate CoA-transferase [EC:2.8.3.18] |
|  | scaffold00003_gene_3806 | | K00239 sdhA, frdA | succinate dehydrogenase / fumarate reductase, flavoprotein subunit [EC:1.3.5.1 1.3.5.4] |
|  | scaffold00003_gene_3807 | | K00240 sdhB, frdB | succinate dehydrogenase / fumarate reductase, iron-sulfur subunit [EC:1.3.5.1 1.3.5.4] |
|  | scaffold00003_gene_3804 | | K00241 sdhC, frdC | succinate dehydrogenase / fumarate reductase, cytochrome b subunit |
|  | scaffold00003_gene_3805 | | K00242 sdhD, frdD | succinate dehydrogenase / fumarate reductase, membrane anchor subunit |
|  | scaffold00003_gene_3306 | | K01676 E4.2.1.2A, fumA, fumB | fumarate hydratase, class I [EC:4.2.1.2] |
|  | scaffold00003_gene_3333 | | K01679 E4.2.1.2B, fumC | fumarate hydratase, class II [EC:4.2.1.2] |
|  | scaffold00003_gene_3802 | | K00024 mdh | malate dehydrogenase [EC:1.1.1.37] |
|  | scaffold00001_gene_1649 | | K01596 E4.1.1.32, pckA, PEPCK | phosphoenolpyruvate carboxykinase (GTP) [EC:4.1.1.32] |
|  | scaffold00001_gene_1422 | | K00163 aceE | pyruvate dehydrogenase E1 component [EC:1.2.4.1] |
|  | scaffold00003_gene_3813 | | K00163 aceE | pyruvate dehydrogenase E1 component [EC:1.2.4.1] |
|  | scaffold00001_gene_1421 | | K00627 DLAT, aceF, pdhC | pyruvate dehydrogenase E2 component (dihydrolipoamide acetyltransferase) [EC:2.3.1.12] |
|  | scaffold00002_gene_2696 | | K00627 DLAT, aceF, pdhC | pyruvate dehydrogenase E2 component (dihydrolipoamide acetyltransferase) [EC:2.3.1.12] |
| 00030 Pentose phosphate pathway [PATH:ko00030] | | | | |
|  | scaffold00001_gene_136 | | K01810 GPI, pgi | glucose-6-phosphate isomerase [EC:5.3.1.9] |
|  | scaffold00002_gene_2380 | | K01810 GPI, pgi | glucose-6-phosphate isomerase [EC:5.3.1.9] |
|  | scaffold00002_gene_2842 | | K01783 rpe, RPE | ribulose-phosphate 3-epimerase [EC:5.1.3.1] |
|  | scaffold00001_gene_1540 | | K00615 E2.2.1.1, tktA, tktB | transketolase [EC:2.2.1.1] |
|  | scaffold00001_gene_1541 | | K00615 E2.2.1.1, tktA, tktB | transketolase [EC:2.2.1.1] |
|  | scaffold00002_gene_1818 | | K00615 E2.2.1.1, tktA, tktB | transketolase [EC:2.2.1.1] |
|  | scaffold00002_gene_1819 | | K00615 E2.2.1.1, tktA, tktB | transketolase [EC:2.2.1.1] |
|  | scaffold00002_gene_2000 | | K00615 E2.2.1.1, tktA, tktB | transketolase [EC:2.2.1.1] |
|  | scaffold00002_gene_1886 | | K00616 E2.2.1.2, talA, talB | transaldolase [EC:2.2.1.2] |
|  | scaffold00005_gene_4720 | | K01807 rpiA | ribose 5-phosphate isomerase A [EC:5.3.1.6] |
|  | scaffold00001_gene_385 | | K13831 hps-phi | 3-hexulose-6-phosphate synthase / 6-phospho-3-hexuloisomerase [EC:4.1.2.43 5.3.1.27] |
|  | scaffold00001_gene_1164 | | K13831 hps-phi | 3-hexulose-6-phosphate synthase / 6-phospho-3-hexuloisomerase [EC:4.1.2.43 5.3.1.27] |
|  | scaffold00001_gene_135 | | K15778 pmm-pgm | phosphomannomutase / phosphoglucomutase [EC:5.4.2.8 5.4.2.2] |
|  | scaffold00002_gene_2379 | | K15778 pmm-pgm | phosphomannomutase / phosphoglucomutase [EC:5.4.2.8 5.4.2.2] |
|  | scaffold00002_gene_1969 | | K05774 phnN | ribose 1,5-bisphosphokinase [EC:2.7.4.23] |
|  | scaffold00002_gene_2360 | | K00948 PRPS, prsA | ribose-phosphate pyrophosphokinase [EC:2.7.6.1] |
|  | scaffold00003_gene_3361 | | K01625 eda | 2-dehydro-3-deoxyphosphogluconate aldolase / (4S)-4-hydroxy-2-oxoglutarate aldolase [EC:4.1.2.14 4.1.3.42] |
|  | scaffold00001_gene_1494 | | K18978 gapN | glyceraldehyde-3-phosphate dehydrogenase [NAD(P)+] [EC:1.2.1.90] |
|  | scaffold00001_gene_838 | | K00034 gdh | glucose 1-dehydrogenase [EC:1.1.1.47] |
|  | scaffold00001_gene_11 | | K01053 E3.1.1.17, gnl, RGN | gluconolactonase [EC:3.1.1.17] |
|  | scaffold00001_gene_708 | | K06151 E1.1.99.3A | gluconate 2-dehydrogenase alpha chain [EC:1.1.99.3] |
|  | scaffold00001_gene_707 | | K06152 E1.1.99.3G | gluconate 2-dehydrogenase gamma chain [EC:1.1.99.3] |
|  | scaffold00001_gene_1745 | | K00090 E1.1.1.215 | gluconate 2-dehydrogenase [EC:1.1.1.215] |
|  | scaffold00003_gene_3362 | | K00874 kdgK | 2-dehydro-3-deoxygluconokinase [EC:2.7.1.45] |
|  | scaffold00002_gene_1952 | | K01624 FBA, fbaA | fructose-bisphosphate aldolase, class II [EC:4.1.2.13] |
|  | scaffold00005_gene_4632 | | K03841 FBP, fbp | fructose-1,6-bisphosphatase I [EC:3.1.3.11] |
| 00040 Pentose and glucuronate interconversions [PATH:ko00040] | | | | |
|  | scaffold00002_gene_2917 | | K01728 pel | pectate lyase [EC:4.2.2.2] |
|  | scaffold00002_gene_2253 | | K00065 kduD | 2-deoxy-D-gluconate 3-dehydrogenase [EC:1.1.1.125] |
|  | scaffold00002_gene_2954 | | K00065 kduD | 2-deoxy-D-gluconate 3-dehydrogenase [EC:1.1.1.125] |
|  | scaffold00004_gene_4487 | | K00065 kduD | 2-deoxy-D-gluconate 3-dehydrogenase [EC:1.1.1.125] |
|  | scaffold00002_gene_2310 | | K01685 uxaA | altronate hydrolase [EC:4.2.1.7] |
|  | scaffold00002_gene_2309 | | K08323 rspA, manD | mannonate dehydratase [EC:4.2.1.8] |
|  | scaffold00001_gene_1018 | | K08322 rspB | L-gulonate 5-dehydrogenase [EC:1.1.1.380] |
|  | scaffold00002_gene_2308 | | K00040 uxuB | fructuronate reductase [EC:1.1.1.57] |
|  | scaffold00002_gene_2959 | | K00012 UGDH, ugd | UDPglucose 6-dehydrogenase [EC:1.1.1.22] |
|  | scaffold00003_gene_3425 | | K00963 UGP2, galU, galF | UTP--glucose-1-phosphate uridylyltransferase [EC:2.7.7.9] |
|  | scaffold00006_gene_4838 | | K00963 UGP2, galU, galF | UTP--glucose-1-phosphate uridylyltransferase [EC:2.7.7.9] |
|  | scaffold00002_gene_2950 | | K13247 CRYL1 | L-gulonate 3-dehydrogenase [EC:1.1.1.45] |
|  | scaffold00002_gene_2842 | | K01783 rpe, RPE | ribulose-phosphate 3-epimerase [EC:5.1.3.1] |
|  | scaffold00002_gene_2316 | | K00008 SORD, gutB | L-iditol 2-dehydrogenase [EC:1.1.1.14] |
|  | scaffold00001_gene_377 | | K00128 E1.2.1.3 | aldehyde dehydrogenase (NAD+) [EC:1.2.1.3] |
|  | scaffold00002_gene_2864 | | K00128 E1.2.1.3 | aldehyde dehydrogenase (NAD+) [EC:1.2.1.3] |
|  | scaffold00003_gene_3561 | | K00128 E1.2.1.3 | aldehyde dehydrogenase (NAD+) [EC:1.2.1.3] |
|  | scaffold00006_gene_4875 | | K00128 E1.2.1.3 | aldehyde dehydrogenase (NAD+) [EC:1.2.1.3] |
| 00051 Fructose and mannose metabolism [PATH:ko00051] | | | | |
|  | scaffold00001_gene_130 | | K16011 algA, xanB, rfbA | mannose-1-phosphate guanylyltransferase / mannose-6-phosphate isomerase [EC:2.7.7.13 5.3.1.8] |
|  | scaffold00001_gene_135 | | K15778 pmm-pgm | phosphomannomutase / phosphoglucomutase [EC:5.4.2.8 5.4.2.2] |
|  | scaffold00002_gene_2379 | | K15778 pmm-pgm | phosphomannomutase / phosphoglucomutase [EC:5.4.2.8 5.4.2.2] |
|  | scaffold00001_gene_128 | | K01711 gmd, GMDS | GDPmannose 4,6-dehydratase [EC:4.2.1.47] |
|  | scaffold00001_gene_127 | | K02377 TSTA3, fcl | GDP-L-fucose synthase [EC:1.1.1.271] |
|  | scaffold00001_gene_1575 | | K01628 fucA | L-fuculose-phosphate aldolase [EC:4.1.2.17] |
|  | scaffold00001_gene_939 | | K18334 fucD | L-fuconate dehydratase [EC:4.2.1.68] |
|  | scaffold00002_gene_2312 | | K18335 K18335 | 2-keto-3-deoxy-L-fuconate dehydrogenase [EC:1.1.1.-] |
|  | scaffold00005_gene_4632 | | K03841 FBP, fbp | fructose-1,6-bisphosphatase I [EC:3.1.3.11] |
|  | scaffold00002_gene_1823 | | K00068 srlD | sorbitol-6-phosphate 2-dehydrogenase [EC:1.1.1.140] |
|  | scaffold00003_gene_3322 | | K00846 KHK | ketohexokinase [EC:2.7.1.3] |
|  | scaffold00002_gene_2316 | | K00008 SORD, gutB | L-iditol 2-dehydrogenase [EC:1.1.1.14] |
|  | scaffold00002_gene_1952 | | K01624 FBA, fbaA | fructose-bisphosphate aldolase, class II [EC:4.1.2.13] |
|  | scaffold00003_gene_3552 | | K01803 TPI, tpiA | triosephosphate isomerase (TIM) [EC:5.3.1.1] |
| 00052 Galactose metabolism [PATH:ko00052] | | | | |
|  | scaffold00001_gene_44 | | K01785 galM, GALM | aldose 1-epimerase [EC:5.1.3.3] |
|  | scaffold00001_gene_137 | | K01784 galE, GALE | UDP-glucose 4-epimerase [EC:5.1.3.2] |
|  | scaffold00004_gene_4420 | | K01784 galE, GALE | UDP-glucose 4-epimerase [EC:5.1.3.2] |
|  | scaffold00003_gene_3425 | | K00963 UGP2, galU, galF | UTP--glucose-1-phosphate uridylyltransferase [EC:2.7.7.9] |
|  | scaffold00006_gene_4838 | | K00963 UGP2, galU, galF | UTP--glucose-1-phosphate uridylyltransferase [EC:2.7.7.9] |
|  | scaffold00001_gene_135 | | K15778 pmm-pgm | phosphomannomutase / phosphoglucomutase [EC:5.4.2.8 5.4.2.2] |
|  | scaffold00002_gene_2379 | | K15778 pmm-pgm | phosphomannomutase / phosphoglucomutase [EC:5.4.2.8 5.4.2.2] |
|  | scaffold00002_gene_2545 | | K01684 dgoD | galactonate dehydratase [EC:4.2.1.6] |
|  | scaffold00002_gene_2543 | | K00883 dgoK | 2-dehydro-3-deoxygalactonokinase [EC:2.7.1.58] |
|  | scaffold00002_gene_2544 | | K01631 dgoA | 2-dehydro-3-deoxyphosphogalactonate aldolase [EC:4.1.2.21] |
| 00053 Ascorbate and aldarate metabolism [PATH:ko00053] | | | | |
|  | scaffold00002_gene_2959 | | K00012 UGDH, ugd | UDPglucose 6-dehydrogenase [EC:1.1.1.22] |
|  | scaffold00001_gene_11 | | K01053 E3.1.1.17, gnl, RGN | gluconolactonase [EC:3.1.1.17] |
|  | scaffold00002_gene_3208 | | K18649 IMPL2 | inositol-phosphate phosphatase / L-galactose 1-phosphate phosphatase / histidinol-phosphatase [EC:3.1.3.25 3.1.3.93 3.1.3.15] |
|  | scaffold00001_gene_1768 | | K02821 PTS-Ula-EIIA, ulaC, sgaA | PTS system, ascorbate-specific IIA component [EC:2.7.1.69] |
|  | scaffold00001_gene_377 | | K00128 E1.2.1.3 | aldehyde dehydrogenase (NAD+) [EC:1.2.1.3] |
|  | scaffold00002_gene_2864 | | K00128 E1.2.1.3 | aldehyde dehydrogenase (NAD+) [EC:1.2.1.3] |
|  | scaffold00003_gene_3561 | | K00128 E1.2.1.3 | aldehyde dehydrogenase (NAD+) [EC:1.2.1.3] |
|  | scaffold00006_gene_4875 | | K00128 E1.2.1.3 | aldehyde dehydrogenase (NAD+) [EC:1.2.1.3] |
|  | scaffold00002_gene_2302 | | K00149 ALDH9A1 | aldehyde dehydrogenase family 9 member A1 [EC:1.2.1.47 1.2.1.3] |
|  | scaffold00001_gene_426 | | K01630 garL | 2-dehydro-3-deoxyglucarate aldolase [EC:4.1.2.20] |
|  | scaffold00001_gene_1183 | | K01707 kdgD | 5-dehydro-4-deoxyglucarate dehydratase [EC:4.2.1.41] |
|  | scaffold00003_gene_3712 | | K01708 garD | galactarate dehydratase [EC:4.2.1.42] |
| 00500 Starch and sucrose metabolism [PATH:ko00500] | | | | |
|  | scaffold00002_gene_2164 | | K01838 pgmB | beta-phosphoglucomutase [EC:5.4.2.6] |
|  | scaffold00001_gene_1071 | | K05343 treS | maltose alpha-D-glucosyltransferase/ alpha-amylase [EC:5.4.99.16 3.2.1.1] |
|  | scaffold00001_gene_1072 | | K16147 glgE | starch synthase (maltosyl-transferring) [EC:2.4.99.16] |
|  | scaffold00001_gene_1069 | | K02438 treX, glgX | glycogen operon protein [EC:3.2.1.-] |
|  | scaffold00001_gene_1066 | | K06044 treY, glgY | (1->4)-alpha-D-glucan 1-alpha-D-glucosylmutase [EC:5.4.99.15] |
|  | scaffold00001_gene_1068 | | K01236 treZ, glgZ | maltooligosyltrehalose trehalohydrolase [EC:3.2.1.141] |
|  | scaffold00002_gene_2959 | | K00012 UGDH, ugd | UDPglucose 6-dehydrogenase [EC:1.1.1.22] |
|  | scaffold00006_gene_4912 | | K08678 UXS1, uxs | UDP-glucuronate decarboxylase [EC:4.1.1.35] |
|  | scaffold00003_gene_3425 | | K00963 UGP2, galU, galF | UTP--glucose-1-phosphate uridylyltransferase [EC:2.7.7.9] |
|  | scaffold00006_gene_4838 | | K00963 UGP2, galU, galF | UTP--glucose-1-phosphate uridylyltransferase [EC:2.7.7.9] |
|  | scaffold00001_gene_135 | | K15778 pmm-pgm | phosphomannomutase / phosphoglucomutase [EC:5.4.2.8 5.4.2.2] |
|  | scaffold00002_gene_2379 | | K15778 pmm-pgm | phosphomannomutase / phosphoglucomutase [EC:5.4.2.8 5.4.2.2] |
|  | scaffold00001_gene_136 | | K01810 GPI, pgi | glucose-6-phosphate isomerase [EC:5.3.1.9] |
|  | scaffold00002_gene_2380 | | K01810 GPI, pgi | glucose-6-phosphate isomerase [EC:5.3.1.9] |
|  | scaffold00001_gene_1073 | | K00703 E2.4.1.21, glgA | starch synthase [EC:2.4.1.21] |
|  | scaffold00001_gene_1070 | | K00700 glgB | 1,4-alpha-glucan branching enzyme [EC:2.4.1.18] |
|  | scaffold00001_gene_1067 | | K00705 malQ | 4-alpha-glucanotransferase [EC:2.4.1.25] |
| 00520 Amino sugar and nucleotide sugar metabolism [PATH:ko00520] | | | | |
|  | scaffold00001_gene_239 | | K01207 nagZ | beta-N-acetylhexosaminidase [EC:3.2.1.52] |
|  | scaffold00003_gene_3290 | | K07102 amgK | anomeric MurNAc/GlcNAc kinase [EC:2.7.1.-] |
|  | scaffold00003_gene_3289 | | K00992 murU | MurNAc alpha-1-phosphate uridylyltransferase [EC:2.7.7.-] |
|  | scaffold00002_gene_2961 | | K04042 glmU | bifunctional UDP-N-acetylglucosamine pyrophosphorylase / Glucosamine-1-phosphate N-acetyltransferase [EC:2.7.7.23 2.3.1.157] |
|  | scaffold00001_gene_978 | | K01791 wecB | UDP-N-acetylglucosamine 2-epimerase (non-hydrolysing) [EC:5.1.3.14] |
|  | scaffold00003_gene_3859 | | K01791 wecB | UDP-N-acetylglucosamine 2-epimerase (non-hydrolysing) [EC:5.1.3.14] |
|  | scaffold00001_gene_979 | | K02472 wecC | UDP-N-acetyl-D-mannosaminuronic acid dehydrogenase [EC:1.1.1.336] |
|  | scaffold00002_gene_2387 | | K02473 E5.1.3.7, wbpP | UDP-N-acetylglucosamine 4-epimerase [EC:5.1.3.7] |
|  | scaffold00002_gene_2388 | | K02474 wbpO | UDP-N-acetyl-D-galactosamine dehydrogenase [EC:1.1.1.-] |
|  | scaffold00001_gene_618 | | K00790 murA | UDP-N-acetylglucosamine 1-carboxyvinyltransferase [EC:2.5.1.7] |
|  | scaffold00002_gene_2998 | | K00790 murA | UDP-N-acetylglucosamine 1-carboxyvinyltransferase [EC:2.5.1.7] |
|  | scaffold00003_gene_3458 | | K00075 murB | UDP-N-acetylmuramate dehydrogenase [EC:1.3.1.98] |
|  | scaffold00002_gene_1868 | | K03431 glmM | phosphoglucosamine mutase [EC:5.4.2.10] |
|  | scaffold00002_gene_2956 | | K00820 glmS, GFPT | glucosamine--fructose-6-phosphate aminotransferase (isomerizing) [EC:2.6.1.16] |
|  | scaffold00006_gene_4912 | | K08678 UXS1, uxs | UDP-glucuronate decarboxylase [EC:4.1.1.35] |
|  | scaffold00001_gene_136 | | K01810 GPI, pgi | glucose-6-phosphate isomerase [EC:5.3.1.9] |
|  | scaffold00002_gene_2380 | | K01810 GPI, pgi | glucose-6-phosphate isomerase [EC:5.3.1.9] |
|  | scaffold00001_gene_135 | | K15778 pmm-pgm | phosphomannomutase / phosphoglucomutase [EC:5.4.2.8 5.4.2.2] |
|  | scaffold00002_gene_2379 | | K15778 pmm-pgm | phosphomannomutase / phosphoglucomutase [EC:5.4.2.8 5.4.2.2] |
|  | scaffold00003_gene_3425 | | K00963 UGP2, galU, galF | UTP--glucose-1-phosphate uridylyltransferase [EC:2.7.7.9] |
|  | scaffold00006_gene_4838 | | K00963 UGP2, galU, galF | UTP--glucose-1-phosphate uridylyltransferase [EC:2.7.7.9] |
|  | scaffold00002_gene_2959 | | K00012 UGDH, ugd | UDPglucose 6-dehydrogenase [EC:1.1.1.22] |
|  | scaffold00001_gene_137 | | K01784 galE, GALE | UDP-glucose 4-epimerase [EC:5.1.3.2] |
|  | scaffold00004_gene_4420 | | K01784 galE, GALE | UDP-glucose 4-epimerase [EC:5.1.3.2] |
|  | scaffold00001_gene_130 | | K16011 algA, xanB, rfbA | mannose-1-phosphate guanylyltransferase / mannose-6-phosphate isomerase [EC:2.7.7.13 5.3.1.8] |
|  | scaffold00001_gene_128 | | K01711 gmd, GMDS | GDPmannose 4,6-dehydratase [EC:4.2.1.47] |
|  | scaffold00001_gene_127 | | K02377 TSTA3, fcl | GDP-L-fucose synthase [EC:1.1.1.271] |
|  | scaffold00007_gene_4999 | | K00523 ascD, ddhD, rfbI | CDP-4-dehydro-6-deoxyglucose reductase, E3 [EC:1.17.1.1] |
| 00620 Pyruvate metabolism [PATH:ko00620] | | | | |
|  | scaffold00001_gene_1650 | | K01895 ACSS, acs | acetyl-CoA synthetase [EC:6.2.1.1] |
|  | scaffold00003_gene_3762 | | K01895 ACSS, acs | acetyl-CoA synthetase [EC:6.2.1.1] |
|  | scaffold00003_gene_3789 | | K01895 ACSS, acs | acetyl-CoA synthetase [EC:6.2.1.1] |
|  | scaffold00001_gene_1422 | | K00163 aceE | pyruvate dehydrogenase E1 component [EC:1.2.4.1] |
|  | scaffold00003_gene_3813 | | K00163 aceE | pyruvate dehydrogenase E1 component [EC:1.2.4.1] |
|  | scaffold00001_gene_1421 | | K00627 DLAT, aceF, pdhC | pyruvate dehydrogenase E2 component (dihydrolipoamide acetyltransferase) [EC:2.3.1.12] |
|  | scaffold00002_gene_2696 | | K00627 DLAT, aceF, pdhC | pyruvate dehydrogenase E2 component (dihydrolipoamide acetyltransferase) [EC:2.3.1.12] |
|  | scaffold00001_gene_893 | | K00382 DLD, lpd, pdhD | dihydrolipoamide dehydrogenase [EC:1.8.1.4] |
|  | scaffold00001_gene_1420 | | K00382 DLD, lpd, pdhD | dihydrolipoamide dehydrogenase [EC:1.8.1.4] |
|  | scaffold00001_gene_1471 | | K00382 DLD, lpd, pdhD | dihydrolipoamide dehydrogenase [EC:1.8.1.4] |
|  | scaffold00002_gene_2697 | | K00382 DLD, lpd, pdhD | dihydrolipoamide dehydrogenase [EC:1.8.1.4] |
|  | scaffold00003_gene_3822 | | K00382 DLD, lpd, pdhD | dihydrolipoamide dehydrogenase [EC:1.8.1.4] |
|  | scaffold00001_gene_699 | | K04072 adhE | acetaldehyde dehydrogenase / alcohol dehydrogenase [EC:1.2.1.10 1.1.1.1] |
|  | scaffold00001_gene_297 | | K04073 mhpF | acetaldehyde dehydrogenase [EC:1.2.1.10] |
|  | scaffold00002_gene_1996 | | K00925 ackA | acetate kinase [EC:2.7.2.1] |
|  | scaffold00002_gene_1995 | | K00625 E2.3.1.8, pta | phosphate acetyltransferase [EC:2.3.1.8] |
|  | scaffold00003_gene_3295 | | K00873 PK, pyk | pyruvate kinase [EC:2.7.1.40] |
|  | scaffold00001_gene_78 | | K01962 accA | acetyl-CoA carboxylase carboxyl transferase subunit alpha [EC:6.4.1.2] |
|  | scaffold00004_gene_4031 | | K02160 accB, bccP | acetyl-CoA carboxylase biotin carboxyl carrier protein |
|  | scaffold00004_gene_4030 | | K01961 accC | acetyl-CoA carboxylase, biotin carboxylase subunit [EC:6.4.1.2 6.3.4.14] |
|  | scaffold00003_gene_3709 | | K01963 accD | acetyl-CoA carboxylase carboxyl transferase subunit beta [EC:6.4.1.2] |
|  | scaffold00002_gene_3152 | | K01512 acyP | acylphosphatase [EC:3.6.1.7] |
|  | scaffold00001_gene_377 | | K00128 E1.2.1.3 | aldehyde dehydrogenase (NAD+) [EC:1.2.1.3] |
|  | scaffold00002_gene_2864 | | K00128 E1.2.1.3 | aldehyde dehydrogenase (NAD+) [EC:1.2.1.3] |
|  | scaffold00003_gene_3561 | | K00128 E1.2.1.3 | aldehyde dehydrogenase (NAD+) [EC:1.2.1.3] |
|  | scaffold00006_gene_4875 | | K00128 E1.2.1.3 | aldehyde dehydrogenase (NAD+) [EC:1.2.1.3] |
|  | scaffold00002_gene_2302 | | K00149 ALDH9A1 | aldehyde dehydrogenase family 9 member A1 [EC:1.2.1.47 1.2.1.3] |
|  | scaffold00002_gene_2849 | | K00138 aldB | aldehyde dehydrogenase [EC:1.2.1.-] |
|  | scaffold00002_gene_1807 | | K18118 aarC, cat1 | succinyl-CoA:acetate CoA-transferase [EC:2.8.3.18] |
|  | scaffold00001_gene_1027 | | K00101 E1.1.2.3, lldD | L-lactate dehydrogenase (cytochrome) [EC:1.1.2.3] |
|  | scaffold00002_gene_2637 | | K00101 E1.1.2.3, lldD | L-lactate dehydrogenase (cytochrome) [EC:1.1.2.3] |
|  | scaffold00002_gene_3150 | | K00101 E1.1.2.3, lldD | L-lactate dehydrogenase (cytochrome) [EC:1.1.2.3] |
|  | scaffold00001_gene_1074 | | K00156 poxB | pyruvate dehydrogenase (quinone) [EC:1.2.5.1] |
|  | scaffold00005_gene_4638 | | K00102 dld, LDHD | D-lactate dehydrogenase (cytochrome) [EC:1.1.2.4] |
|  | scaffold00003_gene_3299 | | K01759 GLO1, gloA | lactoylglutathione lyase [EC:4.4.1.5] |
|  | scaffold00004_gene_3964 | | K01069 E3.1.2.6, gloB | hydroxyacylglutathione hydrolase [EC:3.1.2.6] |
|  | scaffold00004_gene_3917 | | K12972 ghrA | glyoxylate/hydroxypyruvate reductase A [EC:1.1.1.79 1.1.1.81] |
|  | scaffold00002_gene_2004 | | K00029 E1.1.1.40, maeB | malate dehydrogenase (oxaloacetate-decarboxylating)(NADP+) [EC:1.1.1.40] |
|  | scaffold00002_gene_2144 | | K00029 E1.1.1.40, maeB | malate dehydrogenase (oxaloacetate-decarboxylating)(NADP+) [EC:1.1.1.40] |
|  | scaffold00003_gene_3812 | | K00029 E1.1.1.40, maeB | malate dehydrogenase (oxaloacetate-decarboxylating)(NADP+) [EC:1.1.1.40] |
|  | scaffold00003_gene_3802 | | K00024 mdh | malate dehydrogenase [EC:1.1.1.37] |
|  | scaffold00003_gene_3306 | | K01676 E4.2.1.2A, fumA, fumB | fumarate hydratase, class I [EC:4.2.1.2] |
|  | scaffold00003_gene_3333 | | K01679 E4.2.1.2B, fumC | fumarate hydratase, class II [EC:4.2.1.2] |
|  | scaffold00002_gene_2706 | | K01595 ppc | phosphoenolpyruvate carboxylase [EC:4.1.1.31] |
|  | scaffold00001_gene_1649 | | K01596 E4.1.1.32, pckA, PEPCK | phosphoenolpyruvate carboxykinase (GTP) [EC:4.1.1.32] |
|  | scaffold00001_gene_1256 | | K01007 pps, ppsA | pyruvate, water dikinase [EC:2.7.9.2] |
|  | scaffold00001_gene_1328 | | K01007 pps, ppsA | pyruvate, water dikinase [EC:2.7.9.2] |
|  | scaffold00004_gene_4460 | | K01638 aceB, glcB | malate synthase [EC:2.3.3.9] |
|  | scaffold00001_gene_199 | | K00626 E2.3.1.9, atoB | acetyl-CoA C-acetyltransferase [EC:2.3.1.9] |
|  | scaffold00002_gene_2940 | | K00626 E2.3.1.9, atoB | acetyl-CoA C-acetyltransferase [EC:2.3.1.9] |
|  | scaffold00002_gene_3076 | | K00626 E2.3.1.9, atoB | acetyl-CoA C-acetyltransferase [EC:2.3.1.9] |
|  | scaffold00006_gene_4805 | | K00626 E2.3.1.9, atoB | acetyl-CoA C-acetyltransferase [EC:2.3.1.9] |
|  | scaffold00002_gene_3052 | | K01649 leuA | 2-isopropylmalate synthase [EC:2.3.3.13] |
|  | scaffold00001_gene_23 | | K01655 LYS21, LYS20 | homocitrate synthase [EC:2.3.3.14] |
| 00630 Glyoxylate and dicarboxylate metabolism [PATH:ko00630] | | | | |
|  | scaffold00001_gene_245 | | K01637 E4.1.3.1, aceA | isocitrate lyase [EC:4.1.3.1] |
|  | scaffold00004_gene_4460 | | K01638 aceB, glcB | malate synthase [EC:2.3.3.9] |
|  | scaffold00003_gene_3802 | | K00024 mdh | malate dehydrogenase [EC:1.1.1.37] |
|  | scaffold00003_gene_3809 | | K01647 CS, gltA | citrate synthase [EC:2.3.3.1] |
|  | scaffold00001_gene_176 | | K01681 ACO, acnA | aconitate hydratase [EC:4.2.1.3] |
|  | scaffold00002_gene_2484 | | K01681 ACO, acnA | aconitate hydratase [EC:4.2.1.3] |
|  | scaffold00001_gene_189 | | K01682 acnB | aconitate hydratase 2 / 2-methylisocitrate dehydratase [EC:4.2.1.3 4.2.1.99] |
|  | scaffold00001_gene_199 | | K00626 E2.3.1.9, atoB | acetyl-CoA C-acetyltransferase [EC:2.3.1.9] |
|  | scaffold00002_gene_2940 | | K00626 E2.3.1.9, atoB | acetyl-CoA C-acetyltransferase [EC:2.3.1.9] |
|  | scaffold00002_gene_3076 | | K00626 E2.3.1.9, atoB | acetyl-CoA C-acetyltransferase [EC:2.3.1.9] |
|  | scaffold00006_gene_4805 | | K00626 E2.3.1.9, atoB | acetyl-CoA C-acetyltransferase [EC:2.3.1.9] |
|  | scaffold00003_gene_3834 | | K00023 E1.1.1.36, phbB | acetoacetyl-CoA reductase [EC:1.1.1.36] |
|  | scaffold00001_gene_683 | | K11517 HAO | (S)-2-hydroxy-acid oxidase [EC:1.1.3.15] |
|  | scaffold00005_gene_4639 | | K00104 glcD | glycolate oxidase [EC:1.1.3.15] |
|  | scaffold00005_gene_4640 | | K11472 glcE | glycolate oxidase FAD binding subunit |
|  | scaffold00005_gene_4641 | | K11473 glcF | glycolate oxidase iron-sulfur subunit |
|  | scaffold00001_gene_1243 | | K03781 katE, CAT, catB, srpA | catalase [EC:1.11.1.6] |
|  | scaffold00002_gene_3167 | | K03781 katE, CAT, catB, srpA | catalase [EC:1.11.1.6] |
|  | scaffold00002_gene_2843 | | K01091 gph | phosphoglycolate phosphatase [EC:3.1.3.18] |
|  | scaffold00003_gene_3717 | | K01091 gph | phosphoglycolate phosphatase [EC:3.1.3.18] |
|  | scaffold00003_gene_3881 | | K01091 gph | phosphoglycolate phosphatase [EC:3.1.3.18] |
|  | scaffold00004_gene_4251 | | K01091 gph | phosphoglycolate phosphatase [EC:3.1.3.18] |
|  | scaffold00001_gene_578 | | K01601 rbcL | ribulose-bisphosphate carboxylase large chain [EC:4.1.1.39] |
|  | scaffold00001_gene_784 | | K00830 AGXT | alanine-glyoxylate transaminase / serine-glyoxylate transaminase / serine-pyruvate transaminase [EC:2.6.1.44 2.6.1.45 2.6.1.51] |
|  | scaffold00001_gene_952 | | K01915 glnA, GLUL | glutamine synthetase [EC:6.3.1.2] |
|  | scaffold00002_gene_3209 | | K01915 glnA, GLUL | glutamine synthetase [EC:6.3.1.2] |
|  | scaffold00002_gene_2501 | | K00600 glyA, SHMT | glycine hydroxymethyltransferase [EC:2.1.2.1] |
|  | scaffold00004_gene_3984 | | K00600 glyA, SHMT | glycine hydroxymethyltransferase [EC:2.1.2.1] |
|  | scaffold00002_gene_2406 | | K00281 GLDC, gcvP | glycine dehydrogenase [EC:1.4.4.2] |
|  | scaffold00002_gene_2408 | | K00605 gcvT, AMT | aminomethyltransferase [EC:2.1.2.10] |
|  | scaffold00001_gene_893 | | K00382 DLD, lpd, pdhD | dihydrolipoamide dehydrogenase [EC:1.8.1.4] |
|  | scaffold00001_gene_1420 | | K00382 DLD, lpd, pdhD | dihydrolipoamide dehydrogenase [EC:1.8.1.4] |
|  | scaffold00001_gene_1471 | | K00382 DLD, lpd, pdhD | dihydrolipoamide dehydrogenase [EC:1.8.1.4] |
|  | scaffold00002_gene_2697 | | K00382 DLD, lpd, pdhD | dihydrolipoamide dehydrogenase [EC:1.8.1.4] |
|  | scaffold00003_gene_3822 | | K00382 DLD, lpd, pdhD | dihydrolipoamide dehydrogenase [EC:1.8.1.4] |
|  | scaffold00002_gene_2407 | | K02437 gcvH, GCSH | glycine cleavage system H protein |
|  | scaffold00004_gene_4148 | | K01816 hyi, gip | hydroxypyruvate isomerase [EC:5.3.1.22] |
|  | scaffold00004_gene_4169 | | K01816 hyi, gip | hydroxypyruvate isomerase [EC:5.3.1.22] |
|  | scaffold00003_gene_3294 | | K00865 glxK | glycerate kinase [EC:2.7.1.31] |
|  | scaffold00001_gene_418 | | K15919 HPR2 | hydroxypyruvate reductase 2 |
|  | scaffold00004_gene_3917 | | K12972 ghrA | glyoxylate/hydroxypyruvate reductase A [EC:1.1.1.79 1.1.1.81] |
|  | scaffold00002_gene_2105 | | K07246 ttuC, dmlA | tartrate dehydrogenase/decarboxylase / D-malate dehydrogenase [EC:1.1.1.93 4.1.1.73 1.1.1.83] |
|  | scaffold00007_gene_4962 | | K07246 ttuC, dmlA | tartrate dehydrogenase/decarboxylase / D-malate dehydrogenase [EC:1.1.1.93 4.1.1.73 1.1.1.83] |
|  | scaffold00003_gene_3361 | | K01625 eda | 2-dehydro-3-deoxyphosphogluconate aldolase / (4S)-4-hydroxy-2-oxoglutarate aldolase [EC:4.1.2.14 4.1.3.42] |
|  | scaffold00002_gene_2067 | | K00123 fdoG, fdfH | formate dehydrogenase major subunit [EC:1.2.1.2] |
|  | scaffold00002_gene_2229 | | K00123 fdoG, fdfH | formate dehydrogenase major subunit [EC:1.2.1.2] |
|  | scaffold00002_gene_2066 | | K00124 fdoH | formate dehydrogenase iron-sulfur subunit |
|  | scaffold00002_gene_2065 | | K00127 fdoI | formate dehydrogenase subunit gamma |
|  | scaffold00002_gene_2232 | | K00127 fdoI | formate dehydrogenase subunit gamma |
|  | scaffold00004_gene_4078 | | K01432 AFMID | arylformamidase [EC:3.5.1.9] |
|  | scaffold00008_gene_5126 | | K07130 kynB | arylformamidase [EC:3.5.1.9] |
|  | scaffold00003_gene_3343 | | K01433 purU | formyltetrahydrofolate deformylase [EC:3.5.1.10] |
|  | scaffold00001_gene_339 | | K01455 E3.5.1.49 | formamidase [EC:3.5.1.49] |
| 00640 Propanoate metabolism [PATH:ko00640] | | | | |
|  | scaffold00001_gene_1650 | | K01895 ACSS, acs | acetyl-CoA synthetase [EC:6.2.1.1] |
|  | scaffold00003_gene_3762 | | K01895 ACSS, acs | acetyl-CoA synthetase [EC:6.2.1.1] |
|  | scaffold00003_gene_3789 | | K01895 ACSS, acs | acetyl-CoA synthetase [EC:6.2.1.1] |
|  | scaffold00003_gene_3759 | | K01908 prpE | propionyl-CoA synthetase [EC:6.2.1.17] |
|  | scaffold00002_gene_1996 | | K00925 ackA | acetate kinase [EC:2.7.2.1] |
|  | scaffold00002_gene_1995 | | K00625 E2.3.1.8, pta | phosphate acetyltransferase [EC:2.3.1.8] |
|  | scaffold00001_gene_1474 | | K00166 BCKDHA, bkdA1 | 2-oxoisovalerate dehydrogenase E1 component alpha subunit [EC:1.2.4.4] |
|  | scaffold00001_gene_1473 | | K00167 BCKDHB, bkdA2 | 2-oxoisovalerate dehydrogenase E1 component beta subunit [EC:1.2.4.4] |
|  | scaffold00001_gene_1472 | | K09699 DBT, bkdB | 2-oxoisovalerate dehydrogenase E2 component (dihydrolipoyl transacylase) [EC:2.3.1.168] |
|  | scaffold00001_gene_893 | | K00382 DLD, lpd, pdhD | dihydrolipoamide dehydrogenase [EC:1.8.1.4] |
|  | scaffold00001_gene_1420 | | K00382 DLD, lpd, pdhD | dihydrolipoamide dehydrogenase [EC:1.8.1.4] |
|  | scaffold00001_gene_1471 | | K00382 DLD, lpd, pdhD | dihydrolipoamide dehydrogenase [EC:1.8.1.4] |
|  | scaffold00002_gene_2697 | | K00382 DLD, lpd, pdhD | dihydrolipoamide dehydrogenase [EC:1.8.1.4] |
|  | scaffold00003_gene_3822 | | K00382 DLD, lpd, pdhD | dihydrolipoamide dehydrogenase [EC:1.8.1.4] |
|  | scaffold00001_gene_947 | | K00249 ACADM, acd | acyl-CoA dehydrogenase [EC:1.3.8.7] |
|  | scaffold00001_gene_1360 | | K00249 ACADM, acd | acyl-CoA dehydrogenase [EC:1.3.8.7] |
|  | scaffold00002_gene_2552 | | K00249 ACADM, acd | acyl-CoA dehydrogenase [EC:1.3.8.7] |
|  | scaffold00004_gene_4064 | | K00249 ACADM, acd | acyl-CoA dehydrogenase [EC:1.3.8.7] |
|  | scaffold00004_gene_4183 | | K00249 ACADM, acd | acyl-CoA dehydrogenase [EC:1.3.8.7] |
|  | scaffold00006_gene_4791 | | K00249 ACADM, acd | acyl-CoA dehydrogenase [EC:1.3.8.7] |
|  | scaffold00001_gene_866 | | K19745 acuI | acrylyl-CoA reductase (NADPH) [EC:1.3.1.-] |
|  | scaffold00001_gene_94 | | K01692 paaF, echA | enoyl-CoA hydratase [EC:4.2.1.17] |
|  | scaffold00001_gene_201 | | K01692 paaF, echA | enoyl-CoA hydratase [EC:4.2.1.17] |
|  | scaffold00001_gene_210 | | K01692 paaF, echA | enoyl-CoA hydratase [EC:4.2.1.17] |
|  | scaffold00002_gene_2782 | | K01692 paaF, echA | enoyl-CoA hydratase [EC:4.2.1.17] |
|  | scaffold00002_gene_2790 | | K01692 paaF, echA | enoyl-CoA hydratase [EC:4.2.1.17] |
|  | scaffold00002_gene_2819 | | K01692 paaF, echA | enoyl-CoA hydratase [EC:4.2.1.17] |
|  | scaffold00004_gene_4239 | | K01692 paaF, echA | enoyl-CoA hydratase [EC:4.2.1.17] |
|  | scaffold00001_gene_113 | | K01782 fadJ | 3-hydroxyacyl-CoA dehydrogenase / enoyl-CoA hydratase / 3-hydroxybutyryl-CoA epimerase [EC:1.1.1.35 4.2.1.17 5.1.2.3] |
|  | scaffold00001_gene_78 | | K01962 accA | acetyl-CoA carboxylase carboxyl transferase subunit alpha [EC:6.4.1.2] |
|  | scaffold00004_gene_4031 | | K02160 accB, bccP | acetyl-CoA carboxylase biotin carboxyl carrier protein |
|  | scaffold00004_gene_4030 | | K01961 accC | acetyl-CoA carboxylase, biotin carboxylase subunit [EC:6.4.1.2 6.3.4.14] |
|  | scaffold00003_gene_3709 | | K01963 accD | acetyl-CoA carboxylase carboxyl transferase subunit beta [EC:6.4.1.2] |
|  | scaffold00001_gene_209 | | K01578 MLYCD | malonyl-CoA decarboxylase [EC:4.1.1.9] |
|  | scaffold00001_gene_142 | | K00823 puuE | 4-aminobutyrate aminotransferase [EC:2.6.1.19] |
|  | scaffold00002_gene_2396 | | K00822 E2.6.1.18 | beta-alanine--pyruvate transaminase [EC:2.6.1.18] |
|  | scaffold00005_gene_4613 | | K01903 sucC | succinyl-CoA synthetase beta subunit [EC:6.2.1.5] |
|  | scaffold00005_gene_4614 | | K01902 sucD | succinyl-CoA synthetase alpha subunit [EC:6.2.1.5] |
|  | scaffold00002_gene_2397 | | K00140 mmsA, iolA, ALDH6A1 | malonate-semialdehyde dehydrogenase (acetylating) / methylmalonate-semialdehyde dehydrogenase [EC:1.2.1.18 1.2.1.27] |
|  | scaffold00003_gene_3754 | | K00140 mmsA, iolA, ALDH6A1 | malonate-semialdehyde dehydrogenase (acetylating) / methylmalonate-semialdehyde dehydrogenase [EC:1.2.1.18 1.2.1.27] |
|  | scaffold00003_gene_3800 | | K01659 prpC | 2-methylcitrate synthase [EC:2.3.3.5] |
|  | scaffold00003_gene_3799 | | K01720 prpD | 2-methylcitrate dehydratase [EC:4.2.1.79] |
|  | scaffold00001_gene_189 | | K01682 acnB | aconitate hydratase 2 / 2-methylisocitrate dehydratase [EC:4.2.1.3 4.2.1.99] |
|  | scaffold00003_gene_3801 | | K03417 prpB | methylisocitrate lyase [EC:4.1.3.30] |
|  | scaffold00001_gene_199 | | K00626 E2.3.1.9, atoB | acetyl-CoA C-acetyltransferase [EC:2.3.1.9] |
|  | scaffold00002_gene_2940 | | K00626 E2.3.1.9, atoB | acetyl-CoA C-acetyltransferase [EC:2.3.1.9] |
|  | scaffold00002_gene_3076 | | K00626 E2.3.1.9, atoB | acetyl-CoA C-acetyltransferase [EC:2.3.1.9] |
|  | scaffold00006_gene_4805 | | K00626 E2.3.1.9, atoB | acetyl-CoA C-acetyltransferase [EC:2.3.1.9] |
|  | scaffold00004_gene_4109 | | K01034 atoD | acetate CoA/acetoacetate CoA-transferase alpha subunit [EC:2.8.3.8 2.8.3.9] |
|  | scaffold00002_gene_1933 | | K01035 atoA | acetate CoA/acetoacetate CoA-transferase beta subunit [EC:2.8.3.8 2.8.3.9] |
|  | scaffold00001_gene_544 | | K01734 mgsA | methylglyoxal synthase [EC:4.2.3.3] |
|  | scaffold00002_gene_1821 | | K00005 gldA | glycerol dehydrogenase [EC:1.1.1.6] |
| 00650 Butanoate metabolism [PATH:ko00650] | | | | |
|  | scaffold00001_gene_199 | | K00626 E2.3.1.9, atoB | acetyl-CoA C-acetyltransferase [EC:2.3.1.9] |
|  | scaffold00002_gene_2940 | | K00626 E2.3.1.9, atoB | acetyl-CoA C-acetyltransferase [EC:2.3.1.9] |
|  | scaffold00002_gene_3076 | | K00626 E2.3.1.9, atoB | acetyl-CoA C-acetyltransferase [EC:2.3.1.9] |
|  | scaffold00006_gene_4805 | | K00626 E2.3.1.9, atoB | acetyl-CoA C-acetyltransferase [EC:2.3.1.9] |
|  | scaffold00002_gene_2784 | | K00074 paaH, hbd, fadB, mmgB | 3-hydroxybutyryl-CoA dehydrogenase [EC:1.1.1.157] |
|  | scaffold00004_gene_4105 | | K00074 paaH, hbd, fadB, mmgB | 3-hydroxybutyryl-CoA dehydrogenase [EC:1.1.1.157] |
|  | scaffold00001_gene_111 | | K07516 fadN | 3-hydroxyacyl-CoA dehydrogenase [EC:1.1.1.35] |
|  | scaffold00001_gene_731 | | K07516 fadN | 3-hydroxyacyl-CoA dehydrogenase [EC:1.1.1.35] |
|  | scaffold00001_gene_113 | | K01782 fadJ | 3-hydroxyacyl-CoA dehydrogenase / enoyl-CoA hydratase / 3-hydroxybutyryl-CoA epimerase [EC:1.1.1.35 4.2.1.17 5.1.2.3] |
|  | scaffold00001_gene_94 | | K01692 paaF, echA | enoyl-CoA hydratase [EC:4.2.1.17] |
|  | scaffold00001_gene_201 | | K01692 paaF, echA | enoyl-CoA hydratase [EC:4.2.1.17] |
|  | scaffold00001_gene_210 | | K01692 paaF, echA | enoyl-CoA hydratase [EC:4.2.1.17] |
|  | scaffold00002_gene_2782 | | K01692 paaF, echA | enoyl-CoA hydratase [EC:4.2.1.17] |
|  | scaffold00002_gene_2790 | | K01692 paaF, echA | enoyl-CoA hydratase [EC:4.2.1.17] |
|  | scaffold00002_gene_2819 | | K01692 paaF, echA | enoyl-CoA hydratase [EC:4.2.1.17] |
|  | scaffold00004_gene_4239 | | K01692 paaF, echA | enoyl-CoA hydratase [EC:4.2.1.17] |
|  | scaffold00001_gene_905 | | K00209 fabV, ter | enoyl-[acyl-carrier protein] reductase / trans-2-enoyl-CoA reductase (NAD+) [EC:1.3.1.9 1.3.1.44] |
|  | scaffold00004_gene_4109 | | K01034 atoD | acetate CoA/acetoacetate CoA-transferase alpha subunit [EC:2.8.3.8 2.8.3.9] |
|  | scaffold00002_gene_1933 | | K01035 atoA | acetate CoA/acetoacetate CoA-transferase beta subunit [EC:2.8.3.8 2.8.3.9] |
|  | scaffold00001_gene_1285 | | K00634 ptb | phosphate butyryltransferase [EC:2.3.1.19] |
|  | scaffold00001_gene_699 | | K04072 adhE | acetaldehyde dehydrogenase / alcohol dehydrogenase [EC:1.2.1.10 1.1.1.1] |
|  | scaffold00001_gene_297 | | K04073 mhpF | acetaldehyde dehydrogenase [EC:1.2.1.10] |
|  | scaffold00003_gene_3833 | | K03821 phbC, phaC | polyhydroxyalkanoate synthase [EC:2.3.1.-] |
|  | scaffold00003_gene_3696 | | K05973 E3.1.1.75, phaZ | poly(3-hydroxybutyrate) depolymerase [EC:3.1.1.75] |
|  | scaffold00003_gene_3834 | | K00023 E1.1.1.36, phbB | acetoacetyl-CoA reductase [EC:1.1.1.36] |
|  | scaffold00001_gene_95 | | K01039 gctA | glutaconate CoA-transferase, subunit A [EC:2.8.3.12] |
|  | scaffold00001_gene_96 | | K01040 gctB | glutaconate CoA-transferase, subunit B [EC:2.8.3.12] |
|  | scaffold00002_gene_2105 | | K07246 ttuC, dmlA | tartrate dehydrogenase/decarboxylase / D-malate dehydrogenase [EC:1.1.1.93 4.1.1.73 1.1.1.83] |
|  | scaffold00007_gene_4962 | | K07246 ttuC, dmlA | tartrate dehydrogenase/decarboxylase / D-malate dehydrogenase [EC:1.1.1.93 4.1.1.73 1.1.1.83] |
|  | scaffold00002_gene_2823 | | K01799 nicE, maiA | maleate isomerase [EC:5.2.1.1] |
|  | scaffold00003_gene_3806 | | K00239 sdhA, frdA | succinate dehydrogenase / fumarate reductase, flavoprotein subunit [EC:1.3.5.1 1.3.5.4] |
|  | scaffold00003_gene_3807 | | K00240 sdhB, frdB | succinate dehydrogenase / fumarate reductase, iron-sulfur subunit [EC:1.3.5.1 1.3.5.4] |
|  | scaffold00003_gene_3804 | | K00241 sdhC, frdC | succinate dehydrogenase / fumarate reductase, cytochrome b subunit |
|  | scaffold00003_gene_3805 | | K00242 sdhD, frdD | succinate dehydrogenase / fumarate reductase, membrane anchor subunit |
|  | scaffold00001_gene_142 | | K00823 puuE | 4-aminobutyrate aminotransferase [EC:2.6.1.19] |
|  | scaffold00001_gene_141 | | K00135 gabD | succinate-semialdehyde dehydrogenase / glutarate-semialdehyde dehydrogenase [EC:1.2.1.16 1.2.1.79 1.2.1.20] |
|  | scaffold00004_gene_4511 | | K00135 gabD | succinate-semialdehyde dehydrogenase / glutarate-semialdehyde dehydrogenase [EC:1.2.1.16 1.2.1.79 1.2.1.20] |
|  | scaffold00002_gene_1807 | | K18118 aarC, cat1 | succinyl-CoA:acetate CoA-transferase [EC:2.8.3.18] |
|  | scaffold00001_gene_601 | | K08318 yihU | 4-hydroxybutyrate dehydrogenase / sulfolactaldehyde 3-reductase [EC:1.1.1.61 1.1.1.373] |
|  | scaffold00006_gene_4894 | | K08318 yihU | 4-hydroxybutyrate dehydrogenase / sulfolactaldehyde 3-reductase [EC:1.1.1.61 1.1.1.373] |
|  | scaffold00002_gene_2598 | | K01640 E4.1.3.4, HMGCL, hmgL | hydroxymethylglutaryl-CoA lyase [EC:4.1.3.4] |
|  | scaffold00004_gene_4257 | | K01640 E4.1.3.4, HMGCL, hmgL | hydroxymethylglutaryl-CoA lyase [EC:4.1.3.4] |
|  | scaffold00003_gene_3769 | | K01028 E2.8.3.5A, scoA | 3-oxoacid CoA-transferase subunit A [EC:2.8.3.5] |
|  | scaffold00003_gene_3770 | | K01029 E2.8.3.5B, scoB | 3-oxoacid CoA-transferase subunit B [EC:2.8.3.5] |
|  | scaffold00003_gene_3530 | | K00019 E1.1.1.30, bdh | 3-hydroxybutyrate dehydrogenase [EC:1.1.1.30] |
|  | scaffold00004_gene_4457 | | K00019 E1.1.1.30, bdh | 3-hydroxybutyrate dehydrogenase [EC:1.1.1.30] |
|  | scaffold00002_gene_1908 | | K01652 E2.2.1.6L, ilvB, ilvG, ilvI | acetolactate synthase I/II/III large subunit [EC:2.2.1.6] |
|  | scaffold00002_gene_1924 | | K01652 E2.2.1.6L, ilvB, ilvG, ilvI | acetolactate synthase I/II/III large subunit [EC:2.2.1.6] |
|  | scaffold00002_gene_2609 | | K01652 E2.2.1.6L, ilvB, ilvG, ilvI | acetolactate synthase I/II/III large subunit [EC:2.2.1.6] |
|  | scaffold00002_gene_3119 | | K01652 E2.2.1.6L, ilvB, ilvG, ilvI | acetolactate synthase I/II/III large subunit [EC:2.2.1.6] |
|  | scaffold00003_gene_3239 | | K01652 E2.2.1.6L, ilvB, ilvG, ilvI | acetolactate synthase I/II/III large subunit [EC:2.2.1.6] |
|  | scaffold00003_gene_3533 | | K01652 E2.2.1.6L, ilvB, ilvG, ilvI | acetolactate synthase I/II/III large subunit [EC:2.2.1.6] |
|  | scaffold00003_gene_3534 | | K01653 E2.2.1.6S, ilvH, ilvN | acetolactate synthase I/III small subunit [EC:2.2.1.6] |
|  | scaffold00001_gene_364 | | K03366 butA, budC | meso-butanediol dehydrogenase / (S,S)-butanediol dehydrogenase / diacetyl reductase [EC:1.1.1.- 1.1.1.76 1.1.1.304] |
| 00660 C5-Branched dibasic acid metabolism [PATH:ko00660] | | | | |
|  | scaffold00002_gene_1908 | | K01652 E2.2.1.6L, ilvB, ilvG, ilvI | acetolactate synthase I/II/III large subunit [EC:2.2.1.6] |
|  | scaffold00002_gene_1924 | | K01652 E2.2.1.6L, ilvB, ilvG, ilvI | acetolactate synthase I/II/III large subunit [EC:2.2.1.6] |
|  | scaffold00002_gene_2609 | | K01652 E2.2.1.6L, ilvB, ilvG, ilvI | acetolactate synthase I/II/III large subunit [EC:2.2.1.6] |
|  | scaffold00002_gene_3119 | | K01652 E2.2.1.6L, ilvB, ilvG, ilvI | acetolactate synthase I/II/III large subunit [EC:2.2.1.6] |
|  | scaffold00003_gene_3239 | | K01652 E2.2.1.6L, ilvB, ilvG, ilvI | acetolactate synthase I/II/III large subunit [EC:2.2.1.6] |
|  | scaffold00003_gene_3533 | | K01652 E2.2.1.6L, ilvB, ilvG, ilvI | acetolactate synthase I/II/III large subunit [EC:2.2.1.6] |
|  | scaffold00003_gene_3534 | | K01653 E2.2.1.6S, ilvH, ilvN | acetolactate synthase I/III small subunit [EC:2.2.1.6] |
|  | scaffold00003_gene_3320 | | K18289 ict-P | itaconate CoA-transferase [EC:2.8.3.- 2.8.3.22] |
|  | scaffold00005_gene_4613 | | K01903 sucC | succinyl-CoA synthetase beta subunit [EC:6.2.1.5] |
|  | scaffold00005_gene_4614 | | K01902 sucD | succinyl-CoA synthetase alpha subunit [EC:6.2.1.5] |
|  | scaffold00003_gene_3319 | | K18291 ich-P | itaconyl-CoA hydratase / mesaconyl-C4 CoA hydratase [EC:4.2.1.56 4.2.1.-] |
|  | scaffold00003_gene_3321 | | K18292 E4.1.3.25 | (S)-citramalyl-CoA lyase [EC:4.1.3.25] |
|  | scaffold00001_gene_1798 | | K01703 leuC | 3-isopropylmalate/(R)-2-methylmalate dehydratase large subunit [EC:4.2.1.33 4.2.1.35] |
|  | scaffold00002_gene_3099 | | K01703 leuC | 3-isopropylmalate/(R)-2-methylmalate dehydratase large subunit [EC:4.2.1.33 4.2.1.35] |
|  | scaffold00001_gene_1797 | | K01704 leuD | 3-isopropylmalate/(R)-2-methylmalate dehydratase small subunit [EC:4.2.1.33 4.2.1.35] |
|  | scaffold00002_gene_3098 | | K01704 leuD | 3-isopropylmalate/(R)-2-methylmalate dehydratase small subunit [EC:4.2.1.33 4.2.1.35] |
|  | scaffold00001_gene_1796 | | K00052 leuB | 3-isopropylmalate dehydrogenase [EC:1.1.1.85] |
| 00562 Inositol phosphate metabolism [PATH:ko00562] | | | | |
|  | scaffold00001_gene_1447 | | K01114 plcC | phospholipase C [EC:3.1.4.3] |
|  | scaffold00003_gene_3686 | | K01114 plcC | phospholipase C [EC:3.1.4.3] |
|  | scaffold00005_gene_4722 | | K01114 plcC | phospholipase C [EC:3.1.4.3] |
|  | scaffold00001_gene_62 | | K01092 E3.1.3.25, IMPA, suhB | myo-inositol-1(or 4)-monophosphatase [EC:3.1.3.25] |
|  | scaffold00003_gene_3248 | | K01092 E3.1.3.25, IMPA, suhB | myo-inositol-1(or 4)-monophosphatase [EC:3.1.3.25] |
|  | scaffold00002_gene_3208 | | K18649 IMPL2 | inositol-phosphate phosphatase / L-galactose 1-phosphate phosphatase / histidinol-phosphatase [EC:3.1.3.25 3.1.3.93 3.1.3.15] |
|  | scaffold00002_gene_2397 | | K00140 mmsA, iolA, ALDH6A1 | malonate-semialdehyde dehydrogenase (acetylating) / methylmalonate-semialdehyde dehydrogenase [EC:1.2.1.18 1.2.1.27] |
|  | scaffold00003_gene_3754 | | K00140 mmsA, iolA, ALDH6A1 | malonate-semialdehyde dehydrogenase (acetylating) / methylmalonate-semialdehyde dehydrogenase [EC:1.2.1.18 1.2.1.27] |
|  | scaffold00003_gene_3552 | | K01803 TPI, tpiA | triosephosphate isomerase (TIM) [EC:5.3.1.1] |

**Table S5** **Carbohydrate** **transporters identified in strain HZ01.**

| Saccharides | Transporter protein |
| --- | --- |
| Maltose/Maltodextrin | MalE, MalF, MalG, **MalK** (K10111, K10112) |
| Galactose oligomer/Maltooligosaccharide | GanO, GanP, GanQ, **MsmX** (K10111, K10112) |
| Raffinose/Stachyose/Melibiose | MsmE, MsmF, MsmG, **MsmK** (K10112) |
| Lactose/L-arabinose | —^a^ |
| Sorbitol/Mannitol | **SomE** (K10227), **SmoF** (K10228), SmoG, **SmoK** (K10111, K10112) |
| α-Glucoside | AglE, AglF, AglG, **AglK** (K10235, K10112) |
| Trehalose/Maltose | — |
| Trehalose | — |
| N-Acetylglucosamine | — |
| Cellobiose | CebE, CebF, CebG, **MsiK** (K10112) |
| Chitobiose | DasA, DasB, DasC, **MsiK** (K10112) |
| Arabinooligosaccharide | AraN, AraP, AraQ, **MsmX** (K10112) |
| Xylobiose | — |
| Multiple sugar | — |
| Glucose/Arabinose | — |
| Glucose/Mannose | GtsA, GtsB, GtsC, **MalK** (K10112) |
| Ribose/Autoinducer 2/D-Xylose | **RbsB** (K10439), **RbsC** (K10440), RbsA, RbsD |
| L-Arabinose | **AraF** (K10537), **AraH** (K10538), **AraG** (K10539) |
| Methyl-galactoside | — |
| D-Xylose | — |
| D-Allose | — |
| Glycerol | **GlpV** (K17321), **GlpP** (K17322), **GlpQ** (K17323), **GlpS** (K17324), **GlpT** (K17325) |
| Rhamnose | — |
| myo-Inositol | — |
| Erythritol | — |
| Xylitol | — |
| Fructose | — |

Among the listed transporters, the identified proteins were highlighted in bold. ^a^No transporter proteins

were identified.

**Table S6 Genes assigned to the COG category “secondary metabolites biosynthesis, transport, and catabolism”.**

| Predicted gene | COG number | Enzyme/Protein |
| --- | --- | --- |
| scaffold00001_gene_277 | COG3509 | PHB depolymerase family esterase |
| scaffold00001_gene_302 | COG1228 | amidohydrolase |
| scaffold00001_gene_312 | COG3805 | hypothetical protein |
| scaffold00001_gene_347 | COG1335 | annotation not avaliable |
| scaffold00001_gene_376 | COG3485 | intradiol ring-cleavage dioxygenase |
| scaffold00001_gene_297 | COG4569 | acetaldehyde dehydrogenase; Catalyzes the conversion of acetaldehyde to acetyl-CoA, using NA [...] |
| scaffold00001_gene_751 | COG4664 | TRAP-type mannitol/chloroaromatic compound transport system, large permease component |
| scaffold00001_gene_300 | COG0179 | 5-carboxymethyl-2-hydroxymuconate Delta-isomerase |
| scaffold00001_gene_393 | COG3653 | N-acyl-D-glutamate deacylase |
| scaffold00001_gene_636 | COG3971 | 2-oxo-hepta-3-ene-1,7-dioic acid hydratase |
| scaffold00001_gene_637 | COG0179 | 5-carboxymethyl-2-hydroxymuconate delta-isomerase |
| scaffold00001_gene_705 | COG0179 | 5-oxopent-3-ene-1,2,5-tricarboxylate decarboxylase |
| scaffold00001_gene_749 | COG4663 | hypothetical protein |
| scaffold00001_gene_750 | COG4665 | hypothetical protein |
| scaffold00001_gene_899 | COG4181 | ABC transporter ATP-binding protein |
| scaffold00001_gene_985 | COG3321 | polyketide synthase |
| scaffold00001_gene_1008 | COG3653 | putative N-acyl-D-amino-acid deacylase |
| scaffold00001_gene_1029 | COG2124 | cytochrome P450 |
| scaffold00001_gene_1122 | COG0179 | fumarylacetoacetate (FAA) hydrolase |
| scaffold00001_gene_1177 | COG1335 | SlsA |
| scaffold00001_gene_1178 | COG1335 | putative isochorismatase |
| scaffold00001_gene_1188 | COG3486 | alcaligin biosynthesis enzyme |
| scaffold00001_gene_1190 | COG4264 | alcaligin biosynthesis protein |
| scaffold00001_gene_1314 | COG2050 | hypothetical protein |
| scaffold00001_gene_1573 | COG3917 | 2-Hydroxychromene-2-carboxylate isomerase |
| scaffold00001_gene_1588 | COG1335 | isochorismatase hydrolase |
| scaffold00001_gene_1662 | COG2132 | dissimilatory nitrite reductase |
| scaffold00001_gene_1707 | COG2050 | hypothetical protein |
| scaffold00001_gene_1750 | COG5517 | 3-phenylpropionate dioxygenase subunit beta |
| scaffold00001_gene_1792 | COG4663 | putative periplasmic solute-binding protein |
| scaffold00001_gene_1804 | COG2931 | putative hemolysin |
| scaffold00002_gene_1842 | COG1335 | putative isochorismatase |
| scaffold00002_gene_1851 | COG0179 | putative hydrolase |
| scaffold00002_gene_1942 | COG3971 | 2-oxo-hepta-3-ene-1,7-dioic acid hydratase |
| scaffold00002_gene_1949 | COG0179 | 4-hydroxyphenylacetate degradation bifunctional isomerase/decarboxylase, HpaG1 subunit |
| scaffold00002_gene_2043 | COG4663 | TRAP transporter DctP family protein |
| scaffold00002_gene_2044 | COG4665 | TRAP transporter DctQ family protein |
| scaffold00002_gene_2045 | COG4664 | TRAP transporter, DctM subunit |
| scaffold00002_gene_2056 | COG0412 | putative hydrolase |
| scaffold00002_gene_2206 | COG0179 | 5-oxopent-3-ene-1,2,5-tricarboxylate decarboxylase |
| scaffold00002_gene_2311 | COG0179 | 2-hydroxyhepta-2,4-diene-1, 7-dioateisomerase / 5-carboxymethyl-2-oxo-hex-3-ene-1, 7-dioated [...] |
| scaffold00002_gene_2339 | COG3135 | putative transport protein |
| scaffold00002_gene_2369 | COG3508 | homogentisate 1,2-dioxygenase |
| scaffold00002_gene_2370 | COG0179 | fumarylacetoacetase |
| scaffold00002_gene_2500 | COG3135 | benzoate transport protein |
| scaffold00002_gene_2653 | COG3435 | putative gentisate 1,2-dioxygenase |
| scaffold00002_gene_2654 | COG0179 | putative fumarylacetoacetate (FAA) hydrolase |
| scaffold00002_gene_2730 | COG2132 | copper resitance protein |
| scaffold00002_gene_2761 | COG0179 | hypothetical protein |
| scaffold00002_gene_2762 | COG3435 | putative dioxygenase oxidoreductase protein |
| scaffold00002_gene_2823 | COG3473 | Asp/Glu/hydantoin racemase |
| scaffold00002_gene_2852 | COG3917 | 2-hydroxychromene-2-carboxylate isomerase family protein |
| scaffold00002_gene_2878 | COG1228 | imidazolonepropionase |
| scaffold00002_gene_2907 | COG0179 | fumarylacetoacetate (FAA) hydrolase |
| scaffold00002_gene_2990 | COG1127 | putative ABC transporter, ATP-binding protein |
| scaffold00002_gene_2991 | COG0767 | hypothetical protein |
| scaffold00002_gene_2992 | COG1463 | mce related protein |
| scaffold00002_gene_2994 | COG2854 | hypothetical protein |
| scaffold00002_gene_3151 | COG5285 | Phytanoyl-CoA dioxygenase |
| scaffold00002_gene_3171 | COG2175 | taurine dioxygenase |
| scaffold00003_gene_3354 | COG5517 | ring hydroxylating beta subunit |
| scaffold00003_gene_3375 | COG5517 | putative dioxygenase, hydroxylase small component |
| scaffold00003_gene_3390 | COG1335 | hypothetical protein |
| scaffold00003_gene_3504 | COG3653 | putative N-acyl-D-amino-acid deacylase |
| scaffold00003_gene_3572 | COG3653 | putative N-acyl-D-amino-acid deacylase |
| scaffold00003_gene_3635 | COG2050 | hypothetical protein |
| scaffold00003_gene_3671 | COG2175 | putative taurine catabolism dioxygenase |
| scaffold00003_gene_3861 | COG2015 | metallo-beta-lactamase family protein |
| scaffold00004_gene_3981 | COG1335 | hypothetical protein |
| scaffold00004_gene_3999 | COG4663 | putative exported solute binding protein |
| scaffold00004_gene_4006 | COG1335 | putative hydrolase |
| scaffold00004_gene_4021 | COG1020 | putative D-alanine-D-alanyl carrier protein ligase |
| scaffold00004_gene_4054 | COG1335 | isochorismatase hydrolase |
| scaffold00004_gene_4057 | COG0412 | dienelactone hydrolase |
| scaffold00004_gene_4111 | COG4829 | methylmuconolactone isomerase |
| scaffold00004_gene_4152 | COG1228 | amidohydrolase |
| scaffold00004_gene_4153 | COG1228 | hypothetical protein |
| scaffold00004_gene_4413 | COG1127 | ABC transporter ATP-binding protein |
| scaffold00004_gene_4414 | COG0767 | putative integral membrane protein |
| scaffold00004_gene_4463 | COG1335 | hypothetical protein |
| scaffold00005_gene_4552 | COG0412 | putative carboxymethylenebutenolidase |
| scaffold00005_gene_4623 | COG0179 | hypothetical protein |
| scaffold00005_gene_4707 | COG3127 | putative inner membrane transport permease |
| scaffold00006_gene_4758 | COG3971 | hydratase |
| scaffold00006_gene_4830 | COG3485 | catechol 1,2-dioxygenase |
| scaffold00006_gene_4889 | COG2050 | phenylacetic acid degradation-related protein |
| scaffold00007_gene_4952 | COG2015 | beta-lactamase domain-containing protein |
| scaffold00007_gene_5078 | COG2931 | putative hemolysin |
| scaffold00008_gene_5114 | COG3460 | phenylacetate-CoA oxygenase subunit PaaB |
| scaffold00008_gene_5120 | COG2050 | phenylacetic acid degradation protein |
| scaffold00010_gene_5160 | COG2931 | putative hemolysin |
| scaffold00010_gene_5161 | COG2931 | putative hemolysin |
| scaffold00012_gene_5162 | COG2931 | VCBS |
| scaffold00002_gene_2481 | COG3284 | Fis family GAF modulated sigma54 specific transcriptional regulator |
| scaffold00002_gene_2848 | COG3284 | Fis family GAF modulated sigma54 specific transcriptional regulator |
| scaffold00001_gene_659 | COG0500 | hypothetical protein |
| scaffold00001_gene_1106 | COG0500 | ribosomal protein L11 methylase-like protein |
| scaffold00001_gene_1648 | COG0500 | hypothetical protein |
| scaffold00002_gene_1829 | COG0500 | thiopurine S-methyltransferase |
| scaffold00002_gene_2222 | COG0500 | hypothetical protein |
| scaffold00002_gene_2710 | COG0500 | UbiE/COQ5 methyltransferase family protein |
| scaffold00002_gene_2743 | COG0500 | hypothetical protein |
| scaffold00004_gene_3963 | COG0500 | hypothetical protein |
| scaffold00004_gene_4219 | COG0500 | hypothetical protein |
| scaffold00004_gene_4424 | COG0500 | hypothetical protein |
| scaffold00002_gene_2176 | COG0123 | Histone deacetylase |
| scaffold00003_gene_3900 | COG0123 | histone deacetylase family protein |
| scaffold00001_gene_920 | COG0146 | 5-oxoprolinase |
| scaffold00002_gene_2125 | COG3191 | peptidase S58, DmpA |
| scaffold00002_gene_3145 | COG0146 | 5-oxoprolinase (ATP-hydrolyzing) |
| scaffold00002_gene_3146 | COG0145 | 5-oxoprolinase (ATP-hydrolyzing) |
| scaffold00003_gene_3778 | COG3191 | D-aminopeptidase |
| scaffold00004_gene_4157 | COG0145 | hydantoinase A |
| scaffold00001_gene_97 | COG0318 | putative long-chain fatty acid:CoA ligase |
| scaffold00001_gene_108 | COG0318 | acyl-CoA synthetase, long-chain-fatty-acid--CoA ligase |
| scaffold00001_gene_211 | COG0318 | malonyl-CoA synthase |
| scaffold00001_gene_1559 | COG0318 | acyl-CoA synthetase |
| scaffold00001_gene_1668 | COG0318 | acyl-CoA synthetase |
| scaffold00001_gene_1690 | COG0236 | putative acyl carrier protein |
| scaffold00001_gene_1691 | COG0236 | acyl carrier protein |
| scaffold00001_gene_1701 | COG0304 | 3-oxoacyl-(acyl carrier protein) synthase I |
| scaffold00001_gene_1704 | COG0304 | 3-oxoacyl-(acyl carrier protein) synthase II |
| scaffold00002_gene_1926 | COG0318 | acyl-CoA synthetase |
| scaffold00002_gene_2494 | COG0318 | putative crotonobetaine/carnitine-CoA ligase |
| scaffold00002_gene_2939 | COG0318 | fatty acid CoA ligase |
| scaffold00003_gene_3247 | COG0318 | acyl-CoA synthetase, long-chain-fatty-acid--CoA ligase |
| scaffold00003_gene_3313 | COG0318 | Putative AMP-dependent synthetase and ligase; ATP-dependent AMP-binding enzyme family |
| scaffold00003_gene_3374 | COG0318 | putative AMP-binding protein |
| scaffold00003_gene_3729 | COG0236 | acyl carrier protein; Carrier of the growing fatty acid chain in fatty acid biosynthesis (By [...] |
| scaffold00003_gene_3730 | COG0304 | 3-oxoacyl-(acyl carrier protein) synthase II |
| scaffold00004_gene_4125 | COG0318 | putative long-chain-fatty-acid--CoA ligase |
| scaffold00004_gene_4184 | COG0318 | long-chain-fatty-acid--CoA ligase |
| scaffold00004_gene_4195 | COG0318 | putative ligase |
| scaffold00006_gene_4855 | COG0318 | acyl-CoA synthetase |
| scaffold00006_gene_4887 | COG0318 | putative acetyl-CoA synthetase |
| scaffold00001_gene_363 | COG1028 | short-chain dehydrogenase/reductase SDR |
| scaffold00001_gene_364 | COG1028 | short-chain dehydrogenase/reductase SDR |
| scaffold00001_gene_406 | COG1028 | short chain dehydrogenase |
| scaffold00001_gene_432 | COG1028 | short-chain dehydrogenase |
| scaffold00001_gene_555 | COG1028 | short chain dehydrogenase |
| scaffold00001_gene_634 | COG1028 | short chain dehydrogenase |
| scaffold00001_gene_838 | COG1028 | glucose 1-dehydrogenase |
| scaffold00001_gene_930 | COG1028 | putative oxidoreductase |
| scaffold00001_gene_937 | COG1028 | short chain dehydrogenase |
| scaffold00001_gene_1054 | COG1028 | short chain dehydrogenase |
| scaffold00001_gene_1115 | COG1028 | short-chain type dehydrogenase/reductase |
| scaffold00001_gene_1210 | COG1028 | short-chain dehydrogenase/reductase SDR |
| scaffold00001_gene_1476 | COG1028 | short-chain dehydrogenase/reductase SDR |
| scaffold00001_gene_1477 | COG1028 | short-chain dehydrogenase/reductase SDR |
| scaffold00001_gene_1496 | COG1028 | 3-ketoacyl-(acyl-carrier-protein) reductase |
| scaffold00001_gene_1542 | COG1028 | short-chain dehydrogenase/reductase SDR |
| scaffold00001_gene_1556 | COG1028 | short-chain dehydrogenase |
| scaffold00001_gene_1703 | COG1028 | 3-ketoacyl-(acyl-carrier-protein) reductase |
| scaffold00002_gene_1823 | COG1028 | short chain dehydrogenase |
| scaffold00002_gene_1828 | COG1028 | probable short-chain dehydrogenase/reductase |
| scaffold00002_gene_1856 | COG1028 | short chain dehydrogenase |
| scaffold00002_gene_1857 | COG1028 | short chain dehydrogenase |
| scaffold00002_gene_1882 | COG1028 | short chain dehydrogenase |
| scaffold00002_gene_2083 | COG1028 | putative short chain dehydrogenase |
| scaffold00002_gene_2153 | COG1028 | short-chain dehydrogenase/reductase SDR |
| scaffold00002_gene_2161 | COG1028 | short chain dehydrogenase |
| scaffold00002_gene_2163 | COG1028 | short chain dehydrogenase |
| scaffold00002_gene_2246 | COG1028 | short-chain dehydrogenase/reductase SDR |
| scaffold00002_gene_2253 | COG1028 | putative short-chain dehydrogenase/reductase |
| scaffold00002_gene_2312 | COG1028 | oxidoreductase |
| scaffold00002_gene_2555 | COG1028 | short-chain dehydrogenase/reductase SDR |
| scaffold00002_gene_2561 | COG1028 | short-chain dehydrogenase/reductase SDR |
| scaffold00002_gene_2603 | COG1028 | short-chain dehydrogenase/reductase SDR |
| scaffold00002_gene_2624 | COG1028 | short-chain dehydrogenase/reductase SDR |
| scaffold00002_gene_2693 | COG1028 | short-chain dehydrogenase/reductase SDR |
| scaffold00002_gene_2770 | COG1028 | Short-chain dehydrogenase/reductase SDR |
| scaffold00002_gene_2935 | COG1028 | short-chain dehydrogenase/reductase SDR |
| scaffold00002_gene_2944 | COG1028 | short chain dehydrogenase |
| scaffold00002_gene_2954 | COG1028 | short chain dehydrogenase |
| scaffold00002_gene_3118 | COG1028 | gluconate 5-dehydrogenase |
| scaffold00003_gene_3240 | COG1028 | putative oxidoreductase |
| scaffold00003_gene_3281 | COG1028 | putative short-chain dehydrogenase |
| scaffold00003_gene_3309 | COG1028 | short-chain dehydrogenase |
| scaffold00003_gene_3370 | COG1028 | Short-chain dehydrogenase family protein |
| scaffold00003_gene_3372 | COG1028 | Short-chain dehydrogenase family protein |
| scaffold00003_gene_3530 | COG1028 | putative oxidoreductase |
| scaffold00003_gene_3559 | COG1028 | dehydrogenase/reductase oxidoreductase protein |
| scaffold00003_gene_3605 | COG1028 | 3-ketoacyl-(acyl-carrier-protein) reductase |
| scaffold00003_gene_3728 | COG1028 | 3-ketoacyl-(acyl-carrier-protein) reductase |
| scaffold00003_gene_3834 | COG1028 | 3-ketoacyl-(acyl-carrier-protein) reductase |
| scaffold00004_gene_4053 | COG1028 | short chain dehydrogenase |
| scaffold00004_gene_4066 | COG1028 | short-chain dehydrogenase/reductase SDR |
| scaffold00004_gene_4107 | COG1028 | short-chain dehydrogenase |
| scaffold00004_gene_4127 | COG1028 | short chain dehydrogenase |
| scaffold00004_gene_4188 | COG1028 | putative short chain dehydrogenase |
| scaffold00004_gene_4228 | COG1028 | short-chain dehydrogenase |
| scaffold00004_gene_4238 | COG1028 | short chain dehydrogenase |
| scaffold00004_gene_4457 | COG1028 | 3-hydroxybutyrate dehydrogenase |
| scaffold00004_gene_4487 | COG1028 | putative 3-oxoacyl-(acyl-carrier-protein) reductase |
| scaffold00006_gene_4795 | COG1028 | short chain dehydrogenase |
| scaffold00006_gene_4808 | COG1028 | putative glucose 1-dehydrogenase homolog YxnA |
| scaffold00007_gene_5040 | COG1028 | putative oxidoreductase |

**Table S7** **Potential genes related to the synthesis of biosurfactants.**

| Predicted gene | Annotation |
| --- | --- |
| scaffold00001_gene_1689 | acyltransferase |
| scaffold00002_gene_2041 | acyltransferase |
| scaffold00002_gene_2371 | acyltransferase |
| scaffold00002_gene_2426 | acyltransferase |
| scaffold00003_gene_3771 | acyltransferase |
| scaffold00004_gene_4042 | acyltransferase |
| scaffold00004_gene_4380 | acyltransferase |
| scaffold00002_gene_2469 | cell envelope biogenesis protein OmpA |
| scaffold00003_gene_3528 | Outer membrane protein OmpAb |
| scaffold00001_gene_1703 | 3-ketoacyl-ACP reductase |
| scaffold00002_gene_1823 | 3-ketoacyl-ACP reductase |
| scaffold00002_gene_2687 | 3-oxoacyl-ACP reductase |
| scaffold00003_gene_3559 | 3-oxoacyl-ACP reductase |
| scaffold00003_gene_3605 | 3-ketoacyl-ACP reductase |
| scaffold00003_gene_3728 | 3-ketoacyl-ACP reductase |
| scaffold00004_gene_4107 | short-chain dehydrogenase |
| scaffold00004_gene_4228 | 3-oxoacyl-[acyl-carrier-protein] reductase FabG |
| scaffold00004_gene_4238 | 3-oxoacyl-[acyl-carrier-protein] reductase FabG |
| scaffold00004_gene_4487 | 3-oxoacyl-ACP reductase |
| scaffold00001_gene_135 | phosphomannomutase/phosphoglucomutase |
| scaffold00001_gene_1336 | OmpH |
| scaffold00001_gene_1007 | aspartate racemase |
| scaffold00001_gene_1695 | glycosyl transferase |
| scaffold00001_gene_1694 | glycosyl transferase |
| scaffold00001_gene_1716 | N-glycosyltransferase |
| scaffold00002_gene_2923 | glycosyl transferase |
| scaffold00004_gene_4423 | glycosyl transferase family 1 |
| scaffold00008_gene_5123 | glycosyltransferase |
| scaffold00001_gene_118 | LuxR family transcriptional regulator |
| scaffold00001_gene_119 | LuxR family transcriptional regulator |
| scaffold00001_gene_1127 | LuxR family transcriptional regulator |
| scaffold00001_gene_1267 | LuxR family transcriptional regulator |
| scaffold00001_gene_1803 | LuxR family transcriptional regulator |
| scaffold00001_gene_1803 | LuxR family transcriptional regulator |
| scaffold00002_gene_2049 | LuxR family transcriptional regulator |
| scaffold00002_gene_2752 | LuxR family transcriptional regulator |
| scaffold00002_gene_2934 | LuxR family transcriptional regulator |
| scaffold00003_gene_3600 | LuxR family transcriptional regulator |
| scaffold00004_gene_4099 | LuxR family transcriptional regulator |
| scaffold00001_gene_1314 | thioesterase |
| scaffold00001_gene_1696 | thioesterase |
| scaffold00003_gene_3635 | thioesterase |
| scaffold00003_gene_3768 | thioesterase |
| scaffold00006_gene_4889 | thioesterase |
| scaffold00006_gene_4940 | thioesterase |
| scaffold00001_gene_1207 | peptide synthetase |
| scaffold00002_gene_2478 | peptide synthetase |

**Table S8 Candidate genes related to the degradation of petroleum components in strain HZ01.**

| Gene | Annotation in NCBI-nr |
| --- | --- |
| scaffold00004_gene_4047 | rubredoxin |
| scaffold00001_gene_1029 | cytochrome P450 |
| scaffold00003_gene_3377 | ferredoxin |
| scaffold00003_gene_3450 | ferredoxin |
| scaffold00003_gene_3695 | ferredoxin |
| scaffold00003_gene_3697 | ferredoxin-NADP reductase |
| scaffold00003_gene_3698 | ferredoxin |
| scaffold00006_gene_4814 | (2Fe-2S) ferredoxin |
| scaffold00006_gene_4920 | ferredoxin |
| scaffold00001_gene_356 | ferredoxin |
| scaffold00001_gene_542 | (2Fe-2S) ferredoxin |
| scaffold00001_gene_962 | ferredoxin-NADP reductase |
| scaffold00001_gene_1058 | ferredoxin |
| scaffold00002_gene_2226 | 4Fe-4S ferredoxin |
| scaffold00002_gene_2338 | ferredoxin |
| scaffold00002_gene_2350 | ferredoxin |
| scaffold00002_gene_2868 | Ferredoxin I |
| scaffold00002_gene_2869 | ferredoxin |
| scaffold00002_gene_2870 | ferredoxin |
| scaffold00003_gene_3276 | ferredoxin |
| scaffold00003_gene_3579 | FAD-dependent oxidoreductase |
| scaffold00005_gene_4564 | FAD-dependent oxidoreductase |
| scaffold00001_gene_668 | FAD-dependent oxidoreductase |
| scaffold00001_gene_830 | FAD-dependent oxidoreductase |
| scaffold00001_gene_1276 | FAD-dependent oxidoreductase |
| scaffold00001_gene_1699 | FAD-dependent oxidoreductase |
| scaffold00002_gene_2366 | FAD-dependent oxidoreductase |
| scaffold00002_gene_2482 | FAD-dependent oxidoreductase |
| scaffold00002_gene_2911 | FAD-dependent oxidoreductase |
| scaffold00002_gene_3136 | FAD-dependent oxidoreductase |
| scaffold00002_gene_3139 | FAD-dependent oxidoreductase |
| scaffold00003_gene_3344 | FAD-dependent oxidoreductase |
| scaffold00001_gene_473 | maleylacetate reductase 1 |
| scaffold00002_gene_1940 | MarR family transcriptional regulator |
| scaffold00002_gene_2150 | MarR family transcriptional regulator |
| scaffold00002_gene_2301 | MarR family transcriptional regulator |
| scaffold00002_gene_2343 | MarR family transcriptional regulator |
| scaffold00003_gene_3380 | MarR family transcriptional regulator |
| scaffold00004_gene_4079 | MarR family transcriptional regulator |
| scaffold00006_gene_4765 | MarR family transcriptional regulator |
| scaffold00007_gene_4967 | MarR family transcriptional regulator |
| scaffold00007_gene_4991 | MarR family transcriptional regulator |
| scaffold00001_gene_152 | MarR family transcriptional regulator |
| scaffold00001_gene_413 | MarR family transcriptional regulator |
| scaffold00001_gene_752 | MarR family transcriptional regulator |
| scaffold00001_gene_809 | MarR family transcriptional regulator |
| scaffold00001_gene_1216 | MarR family transcriptional regulator |
| scaffold00001_gene_1483 | MarR family transcriptional regulator |
| scaffold00002_gene_2048 | GntR family transcriptional regulator |
| scaffold00002_gene_2090 | GntR family transcriptional regulator |
| scaffold00002_gene_2201 | GntR family transcriptional regulator |
| scaffold00002_gene_2207 | GntR family transcriptional regulator |
| scaffold00002_gene_2317 | GntR family transcriptional regulator |
| scaffold00002_gene_2352 | GntR family transcriptional regulator |
| scaffold00002_gene_2373 | GntR family transcriptional regulator |
| scaffold00002_gene_2395 | GntR family transcriptional regulator |
| scaffold00002_gene_2434 | GntR family transcriptional regulator |
| scaffold00002_gene_2483 | GntR family transcriptional regulator |
| scaffold00002_gene_2502 | GntR family transcriptional regulator |
| scaffold00002_gene_2512 | GntR family transcriptional regulator |
| scaffold00002_gene_2686 | GntR family transcriptional regulator |
| scaffold00002_gene_2739 | GntR family transcriptional regulator |
| scaffold00002_gene_2745 | GntR family transcriptional regulator |
| scaffold00002_gene_2941 | GntR family transcriptional regulator |
| scaffold00002_gene_3144 | GntR family transcriptional regulator |
| scaffold00003_gene_3237 | GntR family transcriptional regulator |
| scaffold00003_gene_3803 | GntR family transcriptional regulator |
| scaffold00004_gene_3940 | GntR family transcriptional regulator |
| scaffold00004_gene_4165 | GntR family transcriptional regulator |
| scaffold00004_gene_4206 | GntR family transcriptional regulator |
| scaffold00005_gene_4701 | GntR family transcriptional regulator |
| scaffold00001_gene_98 | GntR family transcriptional regulator |
| scaffold00001_gene_177 | GntR family transcriptional regulator |
| scaffold00001_gene_208 | GntR family transcriptional regulator |
| scaffold00001_gene_368 | GntR family transcriptional regulator |
| scaffold00001_gene_387 | GntR family transcriptional regulator |
| scaffold00001_gene_462 | GntR family transcriptional regulator |
| scaffold00001_gene_697 | GntR family transcriptional regulator |
| scaffold00001_gene_724 | GntR family transcriptional regulator |
| scaffold00001_gene_783 | GntR family transcriptional regulator |
| scaffold00001_gene_1250 | GntR family transcriptional regulator |
| scaffold00001_gene_1495 | GntR family transcriptional regulator |
| scaffold00001_gene_1560 | GntR family transcriptional regulator |
| scaffold00001_gene_1587 | GntR family transcriptional regulator |
| scaffold00001_gene_1640 | GntR family transcriptional regulator |
| scaffold00001_gene_1752 | GntR family transcriptional regulator |
| scaffold00002_gene_1820 | GntR family transcriptional regulator |
| scaffold00002_gene_1850 | GntR family transcriptional regulator |
| scaffold00002_gene_2136 | cytochrome o ubiquinol oxidase subunit I |
| scaffold00002_gene_2137 | cytochrome o ubiquinol oxidase subunit III |
| scaffold00002_gene_2138 | cytochrome o ubiquinol oxidase |
| scaffold00002_gene_2181 | cytochrome o ubiquinol oxidase |
| scaffold00002_gene_2182 | cytochrome o ubiquinol oxidase subunit I |
| scaffold00002_gene_2183 | cytochrome o ubiquinol oxidase subunit III |
| scaffold00002_gene_2184 | cytochrome o ubiquinol oxidase |
| scaffold00001_gene_1559 | acyl-CoA synthetase |
| scaffold00001_gene_1668 | acyl-CoA synthetase |
| scaffold00002_gene_1926 | acyl-CoA synthetase |
| scaffold00002_gene_2767 | acyl-CoA synthetase |
| scaffold00002_gene_2779 | acyl-CoA synthetase |
| scaffold00004_gene_4184 | acyl-CoA synthetase |
| scaffold00004_gene_4190 | acyl-CoA synthetase |
| scaffold00001_gene_696 | fatty acid hydroxylase |
| scaffold00002_gene_1843 | fatty acid hydroxylase |
| scaffold00002_gene_2856 | fatty acid hydroxylase |
| scaffold00001_gene_1205 | 3-(3-hydroxyphenyl)propionate hydroxylase |
| scaffold00002_gene_1883 | aspartyl beta-hydroxylase |
| scaffold00002_gene_2205 | 2,4-dichlorophenol hydroxylase |
| scaffold00002_gene_2390 | 2-octaprenyl-3-methyl-6-methoxy-1,4-benzoquinol hydroxylase |
| scaffold00002_gene_2661 | salicylate hydroxylase |
| scaffold00003_gene_3354 | salicylate hydroxylase |
| scaffold00003_gene_3355 | salicylate hydroxylase |
| scaffold00004_gene_4067 | salicylyl-CoA 5-hydroxylase |
| scaffold00004_gene_4510 | aspartyl beta-hydroxylase |
| scaffold00005_gene_4726 | aspartyl beta-hydroxylase |
| scaffold00006_gene_4930 | prolyl 4-hydroxylase |
| scaffold00006_gene_4931 | proline hydroxylase |
| scaffold00001_gene_375 | 4-nitrocatechol monooxygenase |
| scaffold00001_gene_1112 | nitronate monooxygenase |
| scaffold00001_gene_1509 | monooxygenase |
| scaffold00002_gene_2498 | monooxygenase |
| scaffold00002_gene_2655 | monooxygenase |
| scaffold00002_gene_2989 | nitronate monooxygenase |
| scaffold00003_gene_3411 | monooxygenase |
| scaffold00006_gene_4849 | monooxygenase |
| scaffold00001_gene_175 | aromatic ring-opening dioxygenase LigB |
| scaffold00001_gene_306 | dioxygenase |
| scaffold00001_gene_312 | 4,5-dioxygenase |
| scaffold00001_gene_376 | 6-chlorohydroxyquinol-1,2-dioxygenase |
| scaffold00001_gene_378 | hydroxyquinol 1,2-dioxygenase |
| scaffold00001_gene_379 | hydroquinone dioxygenase small subunit |
| scaffold00001_gene_760 | extradiol dioxygenase |
| scaffold00001_gene_1254 | 2-nitropropane dioxygenase |
| scaffold00002_gene_1944 | 3,4-dihydroxyphenylacetate 2,3-dioxygenase |
| scaffold00002_gene_2159 | alpha-ketoglutarate-dependent dioxygenase |
| scaffold00002_gene_2204 | 2,3-dihydroxybiphenyl dioxygenase |
| scaffold00002_gene_2305 | 3-hydroxyanthranilate 3,4-dioxygenase |
| scaffold00002_gene_2308 | dioxygenase |
| scaffold00002_gene_2369 | homogentisate 1,2-dioxygenase |
| scaffold00002_gene_2644 | phytanoyl-CoA dioxygenase |
| scaffold00002_gene_2653 | gentisate 1,2-dioxygenase |
| scaffold00002_gene_2761 | gentisate 1,2-dioxygenase |
| scaffold00002_gene_2788 | 2-nitropropane dioxygenase |
| scaffold00002_gene_3151 | phytanoyl-CoA dioxygenase |
| scaffold00002_gene_3171 | taurine dioxygenase |
| scaffold00003_gene_3253 | dioxygenase |
| scaffold00003_gene_3311 | 2-nitropropane dioxygenase |
| scaffold00003_gene_3353 | naphthalene 1,2-dioxygenase |
| scaffold00003_gene_3375 | phenylpropionate dioxygenase |
| scaffold00004_gene_3903 | 4-hydroxyphenylpyruvate dioxygenase |
| scaffold00004_gene_4055 | aromatic-ring-hydroxylating dioxygenase |
| scaffold00004_gene_4128 | dioxygenase |
| scaffold00006_gene_4830 | hydroxyquinol 1,2-dioxygenase |
| scaffold00001_gene_216 | alcohol dehydrogenase |
| scaffold00001_gene_699 | alcohol dehydrogenase |
| scaffold00001_gene_709 | alcohol dehydrogenase |
| scaffold00002_gene_1821 | alcohol dehydrogenase |
| scaffold00002_gene_1903 | alcohol dehydrogenase |
| scaffold00002_gene_3211 | alcohol dehydrogenase |
| scaffold00004_gene_4287 | alcohol dehydrogenase |
| scaffold00001_gene_141 | NAD-dependent succinate-semialdehyde dehydrogenase |
| scaffold00001_gene_297 | acetaldehyde dehydrogenase |
| scaffold00001_gene_377 | betaine-aldehyde dehydrogenase |
| scaffold00001_gene_599 | aldehyde dehydrogenase |
| scaffold00001_gene_600 | aldehyde dehydrogenase |
| scaffold00001_gene_1494 | aldehyde dehydrogenase |
| scaffold00001_gene_1548 | betaine-aldehyde dehydrogenase |
| scaffold00002_gene_1946 | betaine-aldehyde dehydrogenase |
| scaffold00002_gene_2302 | betaine-aldehyde dehydrogenase |
| scaffold00002_gene_2540 | aldehyde dehydrogenase |
| scaffold00002_gene_2849 | aldehyde dehydrogenase |
| scaffold00002_gene_2850 | acetaldehyde dehydrogenase |
| scaffold00002_gene_2413 | NAD-dependent dehydratase |
| scaffold00002_gene_2699 | dihydroxy-acid dehydratase |
| scaffold00002_gene_2768 | acyl dehydratase |
| scaffold00002_gene_2774 | dehydratase |
| scaffold00001_gene_99 | 4-hydroxythreonine-4-phosphate dehydrogenase |
| scaffold00001_gene_111 | 3-hydroxyacyl-CoA dehydrogenase |
| scaffold00001_gene_115 | acyl-CoA dehydrogenase |
| scaffold00001_gene_196 | acyl-CoA dehydrogenase |
| scaffold00001_gene_197 | acyl-CoA dehydrogenase |
| scaffold00001_gene_363 | short-chain dehydrogenase |
| scaffold00001_gene_432 | 3-hydroxy-2-methylbutyryl-CoA dehydrogenase |
| scaffold00001_gene_827 | NADP-dependent oxidoreductase |
| scaffold00001_gene_1609 | 3-hydroxybutyryl-CoA dehydrogenase |
| scaffold00002_gene_2065 | formate dehydrogenase |
| scaffold00002_gene_2066 | formate dehydrogenase |
| scaffold00002_gene_2067 | formate dehydrogenase |
| scaffold00002_gene_2068 | formate dehydrogenase |
| scaffold00002_gene_2069 | formate dehydrogenase |
| scaffold00002_gene_2105 | tartrate dehydrogenase |
| scaffold00002_gene_2191 | dehydrogenase |
| scaffold00002_gene_2319 | NADH dehydrogenase |
| scaffold00007_gene_4976 | amine dehydrogenase |
| scaffold00002_gene_2377 | 2-hydroxyacid dehydrogenase |
| scaffold00002_gene_2397 | methylmalonate-semialdehyde dehydrogenase |
| scaffold00002_gene_2553 | zinc-binding dehydrogenase |
| scaffold00002_gene_2637 | FMN-dependent dehydrogenase |
| scaffold00002_gene_2696 | branched-chain alpha-keto acid dehydrogenase subunit E2 |
| scaffold00002_gene_3074 | isovaleryl-CoA dehydrogenase |
| scaffold00003_gene_3888 | prephenate dehydrogenase |
| scaffold00004_gene_4038 | shikimate dehydrogenase |
| scaffold00006_gene_4888 | Zinc-binding dehydrogenase |
| scaffold00002_gene_2801 | hydrolase |
| scaffold00001_gene_1011 | glutathione S-transferase |
| scaffold00001_gene_1673 | glutathione S-transferase |
| scaffold00002_gene_1815 | glutathione S-transferase |
| scaffold00002_gene_1993 | glutathione S-transferase |
| scaffold00002_gene_2533 | glutathione S-transferase |
| scaffold00004_gene_4470 | glutathione S-transferase |
| scaffold00006_gene_4869 | glutathione S-transferase |
| scaffold00003_gene_3832 | laccase |
| scaffold00002_gene_2864 | aldehyde dehydrogenase |
| scaffold00002_gene_2871 | aldehyde dehydrogenase |
| scaffold00002_gene_3210 | aldehyde dehydrogenase |
| scaffold00003_gene_3561 | aldehyde dehydrogenase |
| scaffold00004_gene_4511 | aldehyde dehydrogenase |
| scaffold00006_gene_4875 | aldehyde dehydrogenase |
| scaffold00007_gene_4971 | aldehyde dehydrogenase |
| scaffold00007_gene_5046 | aldehyde dehydrogenase |
| scaffold00008_gene_5118 | aldehyde dehydrogenase |

**Table S9** **Genes assigned to the “fatty acid degradation” pathway in strain HZ01.**

| Predicted gene | Entry/Gene name | Enzyme/Protein |
| --- | --- | --- |
| scaffold00001_gene_199 | K00626 E2.3.1.9, atoB | acetyl-CoA C-acetyltransferase [EC:2.3.1.9] |
| scaffold00002_gene_2940 | K00626 E2.3.1.9, atoB | acetyl-CoA C-acetyltransferase [EC:2.3.1.9] |
| scaffold00002_gene_3076 | K00626 E2.3.1.9, atoB | acetyl-CoA C-acetyltransferase [EC:2.3.1.9] |
| scaffold00006_gene_4805 | K00626 E2.3.1.9, atoB | acetyl-CoA C-acetyltransferase [EC:2.3.1.9] |
| scaffold00001_gene_110 | K00632 fadA, fadI | acetyl-CoA acyltransferase [EC:2.3.1.16] |
| scaffold00001_gene_114 | K00632 fadA, fadI | acetyl-CoA acyltransferase [EC:2.3.1.16] |
| scaffold00001_gene_443 | K00632 fadA, fadI | acetyl-CoA acyltransferase [EC:2.3.1.16] |
| scaffold00001_gene_730 | K00632 fadA, fadI | acetyl-CoA acyltransferase [EC:2.3.1.16] |
| scaffold00001_gene_113 | K01782 fadJ | 3-hydroxyacyl-CoA dehydrogenase / enoyl-CoA hydratase / 3-hydroxybutyryl-CoA epimerase [EC:1.1.1.35 4.2.1.17 5.1.2.3] |
| scaffold00001_gene_94 | K01692 paaF, echA | enoyl-CoA hydratase [EC:4.2.1.17] |
| scaffold00001_gene_201 | K01692 paaF, echA | enoyl-CoA hydratase [EC:4.2.1.17] |
| scaffold00001_gene_210 | K01692 paaF, echA | enoyl-CoA hydratase [EC:4.2.1.17] |
| scaffold00002_gene_2782 | K01692 paaF, echA | enoyl-CoA hydratase [EC:4.2.1.17] |
| scaffold00002_gene_2790 | K01692 paaF, echA | enoyl-CoA hydratase [EC:4.2.1.17] |
| scaffold00002_gene_2819 | K01692 paaF, echA | enoyl-CoA hydratase [EC:4.2.1.17] |
| scaffold00004_gene_4239 | K01692 paaF, echA | enoyl-CoA hydratase [EC:4.2.1.17] |
| scaffold00001_gene_111 | K07516 fadN | 3-hydroxyacyl-CoA dehydrogenase [EC:1.1.1.35] |
| scaffold00001_gene_731 | K07516 fadN | 3-hydroxyacyl-CoA dehydrogenase [EC:1.1.1.35] |
| scaffold00001_gene_947 | K00249 ACADM, acd | acyl-CoA dehydrogenase [EC:1.3.8.7] |
| scaffold00001_gene_1360 | K00249 ACADM, acd | acyl-CoA dehydrogenase [EC:1.3.8.7] |
| scaffold00002_gene_2552 | K00249 ACADM, acd | acyl-CoA dehydrogenase [EC:1.3.8.7] |
| scaffold00004_gene_4064 | K00249 ACADM, acd | acyl-CoA dehydrogenase [EC:1.3.8.7] |
| scaffold00004_gene_4183 | K00249 ACADM, acd | acyl-CoA dehydrogenase [EC:1.3.8.7] |
| scaffold00006_gene_4791 | K00249 ACADM, acd | acyl-CoA dehydrogenase [EC:1.3.8.7] |
| scaffold00001_gene_115 | K06445 fadE | acyl-CoA dehydrogenase [EC:1.3.99.-] |
| scaffold00002_gene_1909 | K00252 GCDH, gcdH | glutaryl-CoA dehydrogenase [EC:1.3.8.6] |
| scaffold00001_gene_108 | K01897 ACSL, fadD | long-chain acyl-CoA synthetase [EC:6.2.1.3] |
| scaffold00001_gene_551 | K01897 ACSL, fadD | long-chain acyl-CoA synthetase [EC:6.2.1.3] |
| scaffold00003_gene_3247 | K01897 ACSL, fadD | long-chain acyl-CoA synthetase [EC:6.2.1.3] |
| scaffold00004_gene_4327 | K01897 ACSL, fadD | long-chain acyl-CoA synthetase [EC:6.2.1.3] |
| scaffold00001_gene_1748 | K00529 hcaD | 3-phenylpropionate/trans-cinnamate dioxygenase ferredoxin reductase component [EC:1.18.1.3] |
| scaffold00002_gene_2882 | K00121 frmA, ADH5, adhC | S-(hydroxymethyl)glutathione dehydrogenase / alcohol dehydrogenase [EC:1.1.1.284 1.1.1.1] |
| scaffold00007_gene_4951 | K00121 frmA, ADH5, adhC | S-(hydroxymethyl)glutathione dehydrogenase / alcohol dehydrogenase [EC:1.1.1.284 1.1.1.1] |
| scaffold00001_gene_699 | K04072 adhE | acetaldehyde dehydrogenase / alcohol dehydrogenase [EC:1.2.1.10 1.1.1.1] |
| scaffold00002_gene_1903 | K13953 adhP | alcohol dehydrogenase, propanol-preferring [EC:1.1.1.1] |
| scaffold00001_gene_377 | K00128 E1.2.1.3 | aldehyde dehydrogenase (NAD+) [EC:1.2.1.3] |
| scaffold00002_gene_2864 | K00128 E1.2.1.3 | aldehyde dehydrogenase (NAD+) [EC:1.2.1.3] |
| scaffold00003_gene_3561 | K00128 E1.2.1.3 | aldehyde dehydrogenase (NAD+) [EC:1.2.1.3] |
| scaffold00006_gene_4875 | K00128 E1.2.1.3 | aldehyde dehydrogenase (NAD+) [EC:1.2.1.3] |
| scaffold00002_gene_2302 | K00149 ALDH9A1 | aldehyde dehydrogenase family 9 member A1 [EC:1.2.1.47 1.2.1.3] |

**Table S10 Genes assigned to the “methane metabolism” pathway.**

| Predicted gene | Entry/Gene name | Enzyme/Protein |
| --- | --- | --- |
| scaffold00002_gene_2882 | K00121 frmA, ADH5, adhC | S-(hydroxymethyl)glutathione dehydrogenase / alcohol dehydrogenase [EC:1.1.1.284 1.1.1.1] |
| scaffold00007_gene_4951 | K00121 frmA, ADH5, adhC | S-(hydroxymethyl)glutathione dehydrogenase / alcohol dehydrogenase [EC:1.1.1.284 1.1.1.1] |
| scaffold00002_gene_2981 | K01070 frmB, ESD, fghA | S-formylglutathione hydrolase [EC:3.1.2.12] |
| scaffold00002_gene_2067 | K00123 fdoG, fdfH | formate dehydrogenase major subunit [EC:1.2.1.2] |
| scaffold00002_gene_2229 | K00123 fdoG, fdfH | formate dehydrogenase major subunit [EC:1.2.1.2] |
| scaffold00002_gene_2066 | K00124 fdoH | formate dehydrogenase iron-sulfur subunit |
| scaffold00002_gene_2065 | K00127 fdoI | formate dehydrogenase subunit gamma |
| scaffold00002_gene_2232 | K00127 fdoI | formate dehydrogenase subunit gamma |
| scaffold00002_gene_2501 | K00600 glyA, SHMT | glycine hydroxymethyltransferase [EC:2.1.2.1] |
| scaffold00004_gene_3984 | K00600 glyA, SHMT | glycine hydroxymethyltransferase [EC:2.1.2.1] |
| scaffold00001_gene_784 | K00830 AGXT | alanine-glyoxylate transaminase / serine-glyoxylate transaminase / serine-pyruvate transaminase [EC:2.6.1.44 2.6.1.45 2.6.1.51] |
| scaffold00003_gene_3784 | K01689 ENO, eno | enolase [EC:4.2.1.11] |
| scaffold00002_gene_2706 | K01595 ppc | phosphoenolpyruvate carboxylase [EC:4.1.1.31] |
| scaffold00003_gene_3802 | K00024 mdh | malate dehydrogenase [EC:1.1.1.37] |
| scaffold00002_gene_1952 | K01624 FBA, fbaA | fructose-bisphosphate aldolase, class II [EC:4.1.2.13] |
| scaffold00005_gene_4632 | K03841 FBP, fbp | fructose-1,6-bisphosphatase I [EC:3.1.3.11] |
| scaffold00001_gene_385 | K13831 hps-phi | 3-hexulose-6-phosphate synthase / 6-phospho-3-hexuloisomerase [EC:4.1.2.43 5.3.1.27] |
| scaffold00001_gene_1164 | K13831 hps-phi | 3-hexulose-6-phosphate synthase / 6-phospho-3-hexuloisomerase [EC:4.1.2.43 5.3.1.27] |
| scaffold00002_gene_1996 | K00925 ackA | acetate kinase [EC:2.7.2.1] |
| scaffold00002_gene_1995 | K00625 E2.3.1.8, pta | phosphate acetyltransferase [EC:2.3.1.8] |
| scaffold00001_gene_1650 | K01895 ACSS, acs | acetyl-CoA synthetase [EC:6.2.1.1] |
| scaffold00003_gene_3762 | K01895 ACSS, acs | acetyl-CoA synthetase [EC:6.2.1.1] |
| scaffold00003_gene_3789 | K01895 ACSS, acs | acetyl-CoA synthetase [EC:6.2.1.1] |
| scaffold00001_gene_1256 | K01007 pps, ppsA | pyruvate, water dikinase [EC:2.7.9.2] |
| scaffold00001_gene_1328 | K01007 pps, ppsA | pyruvate, water dikinase [EC:2.7.9.2] |
| scaffold00004_gene_4211 | K01834 PGAM, gpmA | 2,3-bisphosphoglycerate-dependent phosphoglycerate mutase [EC:5.4.2.11] |
| scaffold00002_gene_1881 | K15634 gpmB | probable phosphoglycerate mutase [EC:5.4.2.12] |
| scaffold00001_gene_386 | K00058 serA, PHGDH | D-3-phosphoglycerate dehydrogenase [EC:1.1.1.95] |
| scaffold00001_gene_1165 | K00058 serA, PHGDH | D-3-phosphoglycerate dehydrogenase [EC:1.1.1.95] |
| scaffold00002_gene_2733 | K00058 serA, PHGDH | D-3-phosphoglycerate dehydrogenase [EC:1.1.1.95] |
| scaffold00002_gene_2749 | K00058 serA, PHGDH | D-3-phosphoglycerate dehydrogenase [EC:1.1.1.95] |
| scaffold00003_gene_3885 | K00831 serC, PSAT1 | phosphoserine aminotransferase [EC:2.6.1.52] |
| scaffold00001_gene_904 | K01079 serB, PSPH | phosphoserine phosphatase [EC:3.1.3.3] |
| scaffold00003_gene_3654 | K01079 serB, PSPH | phosphoserine phosphatase [EC:3.1.3.3] |
| scaffold00001_gene_423 | K05884 comC | L-2-hydroxycarboxylate dehydrogenase (NAD+) [EC:1.1.1.337] |

**Table S11 Genes assigned to “degradation of aromatic compounds”.**

| Predicted gene | Entry/Gene name | Enzyme/Protein |
| --- | --- | --- |
| scaffold00003_gene_3355 | K05549 benA-xylX | benzoate/toluate 1,2-dioxygenase subunit alpha [EC:1.14.12.10 1.14.12.-] |
| scaffold00003_gene_3376 | K05549 benA-xylX | benzoate/toluate 1,2-dioxygenase subunit alpha [EC:1.14.12.10 1.14.12.-] |
| scaffold00002_gene_2862 | K05784 benC-xylZ | benzoate/toluate 1,2-dioxygenase reductase component [EC:1.18.1.-] |
| scaffold00001_gene_376 | K03381 catA | catechol 1,2-dioxygenase [EC:1.13.11.1] |
| scaffold00001_gene_772 | K01856 catB | muconate cycloisomerase [EC:5.5.1.1] |
| scaffold00004_gene_4111 | K03464 catC | muconolactone D-isomerase [EC:5.3.3.4] |
| scaffold00004_gene_4113 | K01055 pcaD | 3-oxoadipate enol-lactonase [EC:3.1.1.24] |
| scaffold00004_gene_4173 | K01607 pcaC | 4-carboxymuconolactone decarboxylase [EC:4.1.1.44] |
| scaffold00001_gene_298 | K01666 mhpE | 4-hydroxy 2-oxovalerate aldolase [EC:4.1.3.39] |
| scaffold00001_gene_297 | K04073 mhpF | acetaldehyde dehydrogenase [EC:1.2.1.10] |
| scaffold00003_gene_3353 | K05710 hcaC | 3-phenylpropionate/trans-cinnamate dioxygenase ferredoxin component |
| scaffold00001_gene_1748 | K00529 hcaD | 3-phenylpropionate/trans-cinnamate dioxygenase ferredoxin reductase component [EC:1.18.1.3] |
| scaffold00001_gene_1550 | K16049 hsaC | 3,4-dihydroxy-9,10-secoandrosta-1,3,5(10)-triene-9,17-dione 4,5-dioxygenase [EC:1.13.11.25] |
| scaffold00001_gene_1751 | K05708 hcaE, hcaA1 | 3-phenylpropionate/trans-cinnamate dioxygenase subunit alpha [EC:1.14.12.19] |
| scaffold00001_gene_1750 | K05709 hcaF, hcaA2 | 3-phenylpropionate/trans-cinnamate dioxygenase subunit beta [EC:1.14.12.19] |
| scaffold00006_gene_4850 | K05713 mhpB | 2,3-dihydroxyphenylpropionate 1,2-dioxygenase [EC:1.13.11.16] |
| scaffold00001_gene_1205 | K05712 mhpA | 3-(3-hydroxy-phenyl)propionate hydroxylase [EC:1.14.13.127] |
| scaffold00002_gene_2366 | K05712 mhpA | 3-(3-hydroxy-phenyl)propionate hydroxylase [EC:1.14.13.127] |
| scaffold00002_gene_2911 | K05712 mhpA | 3-(3-hydroxy-phenyl)propionate hydroxylase [EC:1.14.13.127] |
| scaffold00001_gene_11 | K01053 E3.1.1.17, gnl, RGN | gluconolactonase [EC:3.1.1.17] |
| scaffold00002_gene_1944 | K00455 hpaD, hpcB | 3,4-dihydroxyphenylacetate 2,3-dioxygenase [EC:1.13.11.15] |
| scaffold00002_gene_1946 | K00151 hpaE, hpcC | 5-carboxymethyl-2-hydroxymuconic-semialdehyde dehydrogenase [EC:1.2.1.60] |
| scaffold00002_gene_1948 | K05921 hpaG | 5-oxopent-3-ene-1,2,5-tricarboxylate decarboxylase / 2-hydroxyhepta-2,4-diene-1,7-dioate isomerase [EC:4.1.1.68 5.3.3.-] |
| scaffold00002_gene_2661 | K00480 E1.14.13.1 | salicylate hydroxylase [EC:1.14.13.1] |
| scaffold00002_gene_1903 | K13953 adhP | alcohol dehydrogenase, propanol-preferring [EC:1.1.1.1] |
| scaffold00002_gene_2882 | K00121 frmA, ADH5, adhC | S-(hydroxymethyl)glutathione dehydrogenase / alcohol dehydrogenase [EC:1.1.1.284 1.1.1.1] |
| scaffold00007_gene_4951 | K00121 frmA, ADH5, adhC | S-(hydroxymethyl)glutathione dehydrogenase / alcohol dehydrogenase [EC:1.1.1.284 1.1.1.1] |
| scaffold00001_gene_699 | K04072 adhE | acetaldehyde dehydrogenase / alcohol dehydrogenase [EC:1.2.1.10 1.1.1.1] |

**Table S12 Comparisons of *Achromobacter* strains with bioremediation properties.**

| Strain | Isolated from | Pollutants degraded/removed | Primary genes/enzymes/pathways related to the degradation/removal of pollutants | Genome sequenced |
| --- | --- | --- | --- | --- |
| *Achromobacter* sp. HZ01 | crude oil-contaminated seawater | C_12_–C_27_ *n*-alkanes; anthracene; phenanthrene; pyrene | terminal oxidation pathway; catechol pathway | Yes |
| *Achromobacter* *xylosoxidans* XL | petroleum-contaminated soil | C_12_–C_23_ and C_27_–C_43_ alkanes | not determined | No |
| *Achromobacter xylosoxidans* A8 | soil contaminated with polychlorinated biphenyls | haloaromatic acid | operons *ohbRAB*, *mocpRABCD*, and *hybRABCD*; dioxygenase | Yes |
| *Achromobacter arsenitoxydans* SY8 | arsenic-contaminated soil | arsenite | arsenic resistance operons (*arsR-arsC1-arsD-arsA-arsB*, *arsR-arsO-arsC2-mfs-arsH1*, and *arsR-acr3-mfs-arsC3-arsH2*); arsenite oxidation operon (*aioX-aioS-aioR-aioA-aioB-aioC- aioD*) | Yes |
| *Achromobacter xylosoxidans* KF701 | soil | biphenyl | catechol 2,3-dioxygenase; 2-hydroxymuconic semialdehyde dehydrogenase | No |
| *Achromobacter* sp. 4(2010) | soil contaminated with diesel oil | diesel oil | monooxygenases | No |
| *Achromobacter xylosoxidans* NS12 | a mangrove sediment | *o*-nitrophenol (ONP); *p*-nitrophenol (PNP) | not determined | No |
| *Achromobacter* sp. DMS1 | sediments of long term industrially perturbed ecosystem | polycyclic aromatic hydrocarbons | FAD-dependent oxidoreductase; arsenate, nitrate,  nitrite and sulfite reductase; catechol-1,2-dioxygenase; aromatic-ring opening dioxygenase (ligB); ammonia monooxygenase; shikimate dehydrogenase; tartrate dehydrogenase; NAD(P)H dehydrogenase | Yes |
| *Achromobacter xylosoxidans* DN002 | petroleum-contaminated soil | polycyclic aromatic hydrocarbons | catechol 2,3-dioxygenase; catechol 1,2-dioxygenase; dehydrogenases; dioxygenases | No |
| *Achromobacter* sp. CH-1 | chromate slag | Cr(VI) | Cr(VI) reductase | No |
| *Achromobacter piechaudii* HLE | soil | arsenite | arsenic gene island | Yes |
| *Achromobacter xylosoxidans* subsp.  denitrificans strain EST4002 | soil | 2,4-dichlorophenoxyacetic acid | *tfd*-like genes | No |
| *Achromobacter xylosoxidans* T7 | soil | toluidine isomer; aniline; 2-aminobenzoate; phenol; cresol | catechol 2,3-dioxygenase | No |

**Table S13** **Genes assigned to the “xenobiotics biodegradation and metabolism” pathway.**

| Pathway | Predicted gene | Entry/Gene name | | Enzyme/Protein |
| --- | --- | --- | --- | --- |
| 00362 Benzoate degradation [PATH:ko00362] | | | | |
|  | scaffold00003_gene_3355 | K05549 benA-xylX | benzoate/toluate 1,2-dioxygenase subunit alpha [EC:1.14.12.10 1.14.12.-] | |
|  | scaffold00003_gene_3376 | K05549 benA-xylX | benzoate/toluate 1,2-dioxygenase subunit alpha [EC:1.14.12.10 1.14.12.-] | |
|  | scaffold00002_gene_2862 | K05784 benC-xylZ | benzoate/toluate 1,2-dioxygenase reductase component [EC:1.18.1.-] | |
|  | scaffold00001_gene_376 | K03381 catA | catechol 1,2-dioxygenase [EC:1.13.11.1] | |
|  | scaffold00001_gene_772 | K01856 catB | muconate cycloisomerase [EC:5.5.1.1] | |
|  | scaffold00004_gene_4111 | K03464 catC | muconolactone D-isomerase [EC:5.3.3.4] | |
|  | scaffold00004_gene_4113 | K01055 pcaD | 3-oxoadipate enol-lactonase [EC:3.1.1.24] | |
|  | scaffold00001_gene_110 | K00632 fadA, fadI | acetyl-CoA acyltransferase [EC:2.3.1.16] | |
|  | scaffold00001_gene_114 | K00632 fadA, fadI | acetyl-CoA acyltransferase [EC:2.3.1.16] | |
|  | scaffold00001_gene_443 | K00632 fadA, fadI | acetyl-CoA acyltransferase [EC:2.3.1.16] | |
|  | scaffold00001_gene_730 | K00632 fadA, fadI | acetyl-CoA acyltransferase [EC:2.3.1.16] | |
|  | scaffold00001_gene_298 | K01666 mhpE | 4-hydroxy 2-oxovalerate aldolase [EC:4.1.3.39] | |
|  | scaffold00001_gene_297 | K04073 mhpF | acetaldehyde dehydrogenase [EC:1.2.1.10] | |
|  | scaffold00004_gene_4173 | K01607 pcaC | 4-carboxymuconolactone decarboxylase [EC:4.1.1.44] | |
|  | scaffold00001_gene_113 | K01782 fadJ | 3-hydroxyacyl-CoA dehydrogenase / enoyl-CoA hydratase / 3-hydroxybutyryl-CoA epimerase [EC:1.1.1.35 4.2.1.17 5.1.2.3] | |
|  | scaffold00001_gene_111 | K07516 fadN | 3-hydroxyacyl-CoA dehydrogenase [EC:1.1.1.35] | |
|  | scaffold00001_gene_731 | K07516 fadN | 3-hydroxyacyl-CoA dehydrogenase [EC:1.1.1.35] | |
|  | scaffold00002_gene_1909 | K00252 GCDH, gcdH | glutaryl-CoA dehydrogenase [EC:1.3.8.6] | |
|  | scaffold00001_gene_94 | K01692 paaF, echA | enoyl-CoA hydratase [EC:4.2.1.17] | |
|  | scaffold00001_gene_201 | K01692 paaF, echA | enoyl-CoA hydratase [EC:4.2.1.17] | |
|  | scaffold00001_gene_210 | K01692 paaF, echA | enoyl-CoA hydratase [EC:4.2.1.17] | |
|  | scaffold00002_gene_2782 | K01692 paaF, echA | enoyl-CoA hydratase [EC:4.2.1.17] | |
|  | scaffold00002_gene_2790 | K01692 paaF, echA | enoyl-CoA hydratase [EC:4.2.1.17] | |
|  | scaffold00002_gene_2819 | K01692 paaF, echA | enoyl-CoA hydratase [EC:4.2.1.17] | |
|  | scaffold00004_gene_4239 | K01692 paaF, echA | enoyl-CoA hydratase [EC:4.2.1.17] | |
|  | scaffold00002_gene_2784 | K00074 paaH, hbd, fadB, mmgB | 3-hydroxybutyryl-CoA dehydrogenase [EC:1.1.1.157] | |
|  | scaffold00004_gene_4105 | K00074 paaH, hbd, fadB, mmgB | 3-hydroxybutyryl-CoA dehydrogenase [EC:1.1.1.157] | |
|  | scaffold00001_gene_199 | K00626 E2.3.1.9, atoB | acetyl-CoA C-acetyltransferase [EC:2.3.1.9] | |
|  | scaffold00002_gene_2940 | K00626 E2.3.1.9, atoB | acetyl-CoA C-acetyltransferase [EC:2.3.1.9] | |
|  | scaffold00002_gene_3076 | K00626 E2.3.1.9, atoB | acetyl-CoA C-acetyltransferase [EC:2.3.1.9] | |
|  | scaffold00006_gene_4805 | K00626 E2.3.1.9, atoB | acetyl-CoA C-acetyltransferase [EC:2.3.1.9] | |
| 00627 Aminobenzoate degradation [PATH:ko00627] | | | | |
|  | scaffold00001_gene_712 | K01426 E3.5.1.4, amiE | amidase [EC:3.5.1.4] | |
|  | scaffold00001_gene_1579 | K01426 E3.5.1.4, amiE | amidase [EC:3.5.1.4] | |
|  | scaffold00002_gene_2403 | K01426 E3.5.1.4, amiE | amidase [EC:3.5.1.4] | |
|  | scaffold00002_gene_2828 | K01426 E3.5.1.4, amiE | amidase [EC:3.5.1.4] | |
|  | scaffold00005_gene_4718 | K01426 E3.5.1.4, amiE | amidase [EC:3.5.1.4] | |
|  | scaffold00002_gene_3152 | K01512 acyP | acylphosphatase [EC:3.6.1.7] | |
|  | scaffold00004_gene_4063 | K08295 abmG | 2-aminobenzoate-CoA ligase [EC:6.2.1.32] | |
|  | scaffold00004_gene_4067 | K09461 E1.14.13.40 | anthraniloyl-CoA monooxygenase [EC:1.14.13.40] | |
|  | scaffold00004_gene_4056 | K05599 antA | anthranilate 1,2-dioxygenase (deaminating, decarboxylating) large subunit [EC:1.14.12.1] | |
|  | scaffold00002_gene_2350 | K03863 vanB | vanillate monooxygenase ferredoxin subunit | |
|  | scaffold00004_gene_4109 | K01034 atoD | acetate CoA/acetoacetate CoA-transferase alpha subunit [EC:2.8.3.8 2.8.3.9] | |
|  | scaffold00002_gene_1933 | K01035 atoA | acetate CoA/acetoacetate CoA-transferase beta subunit [EC:2.8.3.8 2.8.3.9] | |
|  | scaffold00001_gene_94 | K01692 paaF, echA | enoyl-CoA hydratase [EC:4.2.1.17] | |
|  | scaffold00001_gene_201 | K01692 paaF, echA | enoyl-CoA hydratase [EC:4.2.1.17] | |
|  | scaffold00001_gene_210 | K01692 paaF, echA | enoyl-CoA hydratase [EC:4.2.1.17] | |
|  | scaffold00002_gene_2782 | K01692 paaF, echA | enoyl-CoA hydratase [EC:4.2.1.17] | |
|  | scaffold00002_gene_2790 | K01692 paaF, echA | enoyl-CoA hydratase [EC:4.2.1.17] | |
|  | scaffold00002_gene_2819 | K01692 paaF, echA | enoyl-CoA hydratase [EC:4.2.1.17] | |
|  | scaffold00004_gene_4239 | K01692 paaF, echA | enoyl-CoA hydratase [EC:4.2.1.17] | |
|  | scaffold00002_gene_3104 | K01113 phoD | alkaline phosphatase D [EC:3.1.3.1] | |
| 00364 Fluorobenzoate degradation [PATH:ko00364] | | | | |
|  | scaffold00003_gene_3355 | K05549 benA-xylX | benzoate/toluate 1,2-dioxygenase subunit alpha [EC:1.14.12.10 1.14.12.-] | |
|  | scaffold00003_gene_3376 | K05549 benA-xylX | benzoate/toluate 1,2-dioxygenase subunit alpha [EC:1.14.12.10 1.14.12.-] | |
|  | scaffold00002_gene_2862 | K05784 benC-xylZ | benzoate/toluate 1,2-dioxygenase reductase component [EC:1.18.1.-] | |
|  | scaffold00001_gene_376 | K03381 catA | catechol 1,2-dioxygenase [EC:1.13.11.1] | |
|  | scaffold00002_gene_2056 | K01061 E3.1.1.45 | carboxymethylenebutenolidase [EC:3.1.1.45] | |
|  | scaffold00004_gene_4057 | K01061 E3.1.1.45 | carboxymethylenebutenolidase [EC:3.1.1.45] | |
|  | scaffold00005_gene_4552 | K01061 E3.1.1.45 | carboxymethylenebutenolidase [EC:3.1.1.45] | |
|  | scaffold00001_gene_772 | K01856 catB | muconate cycloisomerase [EC:5.5.1.1] | |
| 00625 Chloroalkane and chloroalkene degradation [PATH:ko00625] | | | | |
|  | scaffold00002_gene_1903 | K13953 adhP | alcohol dehydrogenase, propanol-preferring [EC:1.1.1.1] | |
|  | scaffold00002_gene_2882 | K00121 frmA, ADH5, adhC | S-(hydroxymethyl)glutathione dehydrogenase / alcohol dehydrogenase [EC:1.1.1.284 1.1.1.1] | |
|  | scaffold00007_gene_4951 | K00121 frmA, ADH5, adhC | S-(hydroxymethyl)glutathione dehydrogenase / alcohol dehydrogenase [EC:1.1.1.284 1.1.1.1] | |
|  | scaffold00001_gene_699 | K04072 adhE | acetaldehyde dehydrogenase / alcohol dehydrogenase [EC:1.2.1.10 1.1.1.1] | |
|  | scaffold00001_gene_377 | K00128 E1.2.1.3 | aldehyde dehydrogenase (NAD+) [EC:1.2.1.3] | |
|  | scaffold00002_gene_2864 | K00128 E1.2.1.3 | aldehyde dehydrogenase (NAD+) [EC:1.2.1.3] | |
|  | scaffold00003_gene_3561 | K00128 E1.2.1.3 | aldehyde dehydrogenase (NAD+) [EC:1.2.1.3] | |
|  | scaffold00006_gene_4875 | K00128 E1.2.1.3 | aldehyde dehydrogenase (NAD+) [EC:1.2.1.3] | |
|  | scaffold00004_gene_4366 | K01561 dehH | haloacetate dehalogenase [EC:3.8.1.3] | |
| 00361 Chlorocyclohexane and chlorobenzene degradation [PATH:ko00361] | | | | |
|  | scaffold00001_gene_376 | K03381 catA | catechol 1,2-dioxygenase [EC:1.13.11.1] | |
|  | scaffold00002_gene_2056 | K01061 E3.1.1.45 | carboxymethylenebutenolidase [EC:3.1.1.45] | |
|  | scaffold00004_gene_4057 | K01061 E3.1.1.45 | carboxymethylenebutenolidase [EC:3.1.1.45] | |
|  | scaffold00005_gene_4552 | K01061 E3.1.1.45 | carboxymethylenebutenolidase [EC:3.1.1.45] | |
|  | scaffold00001_gene_772 | K01856 catB | muconate cycloisomerase [EC:5.5.1.1] | |
|  | scaffold00004_gene_4366 | K01561 dehH | haloacetate dehalogenase [EC:3.8.1.3] | |
| 00623 Toluene degradation [PATH:ko00623] | | | | |
|  | scaffold00001_gene_376 | K03381 catA | catechol 1,2-dioxygenase [EC:1.13.11.1] | |
|  | scaffold00001_gene_772 | K01856 catB | muconate cycloisomerase [EC:5.5.1.1] | |
|  | scaffold00002_gene_2056 | K01061 E3.1.1.45 | carboxymethylenebutenolidase [EC:3.1.1.45] | |
|  | scaffold00004_gene_4057 | K01061 E3.1.1.45 | carboxymethylenebutenolidase [EC:3.1.1.45] | |
|  | scaffold00005_gene_4552 | K01061 E3.1.1.45 | carboxymethylenebutenolidase [EC:3.1.1.45] | |
| 00622 Xylene degradation [PATH:ko00622] | | | | |
|  | scaffold00003_gene_3355 | K05549 benA-xylX | benzoate/toluate 1,2-dioxygenase subunit alpha [EC:1.14.12.10 1.14.12.-] | |
|  | scaffold00003_gene_3376 | K05549 benA-xylX | benzoate/toluate 1,2-dioxygenase subunit alpha [EC:1.14.12.10 1.14.12.-] | |
|  | scaffold00002_gene_2862 | K05784 benC-xylZ | benzoate/toluate 1,2-dioxygenase reductase component [EC:1.18.1.-] | |
|  | scaffold00001_gene_298 | K01666 mhpE | 4-hydroxy 2-oxovalerate aldolase [EC:4.1.3.39] | |
|  | scaffold00001_gene_297 | K04073 mhpF | acetaldehyde dehydrogenase [EC:1.2.1.10] | |
| 00633 Nitrotoluene degradation [PATH:ko00633] | | | | |
|  | scaffold00003_gene_3419 | K10680 nemA | N-ethylmaleimide reductase [EC:1.-.-.-] | |
| 00642 Ethylbenzene degradation [PATH:ko00642] | | | | |
|  | scaffold00001_gene_110 | K00632 fadA, fadI | acetyl-CoA acyltransferase [EC:2.3.1.16] | |
|  | scaffold00001_gene_114 | K00632 fadA, fadI | acetyl-CoA acyltransferase [EC:2.3.1.16] | |
|  | scaffold00001_gene_443 | K00632 fadA, fadI | acetyl-CoA acyltransferase [EC:2.3.1.16] | |
|  | scaffold00001_gene_730 | K00632 fadA, fadI | acetyl-CoA acyltransferase [EC:2.3.1.16] | |
| 00643 Styrene degradation [PATH:ko00643] | | | | |
|  | scaffold00007_gene_4971 | K00146 feaB | phenylacetaldehyde dehydrogenase [EC:1.2.1.39] | |
|  | scaffold00002_gene_2369 | K00451 HGD, hmgA | homogentisate 1,2-dioxygenase [EC:1.13.11.5] | |
|  | scaffold00002_gene_2657 | K01800 maiA, GSTZ1 | maleylacetoacetate isomerase [EC:5.2.1.2] | |
|  | scaffold00002_gene_2370 | K01555 FAH, fahA | fumarylacetoacetase [EC:3.7.1.2] | |
|  | scaffold00001_gene_1122 | K16171 faaH | fumarylacetoacetate (FAA) hydrolase [EC:3.7.1.2] | |
|  | scaffold00001_gene_712 | K01426 E3.5.1.4, amiE | amidase [EC:3.5.1.4] | |
|  | scaffold00001_gene_1579 | K01426 E3.5.1.4, amiE | amidase [EC:3.5.1.4] | |
|  | scaffold00002_gene_2403 | K01426 E3.5.1.4, amiE | amidase [EC:3.5.1.4] | |
|  | scaffold00002_gene_2828 | K01426 E3.5.1.4, amiE | amidase [EC:3.5.1.4] | |
|  | scaffold00005_gene_4718 | K01426 E3.5.1.4, amiE | amidase [EC:3.5.1.4] | |
|  | scaffold00001_gene_95 | K01039 gctA | glutaconate CoA-transferase, subunit A [EC:2.8.3.12] | |
|  | scaffold00001_gene_96 | K01040 gctB | glutaconate CoA-transferase, subunit B [EC:2.8.3.12] | |
| 00791 Atrazine degradation [PATH:ko00791] | | | | |
|  | scaffold00002_gene_2092 | K01457 E3.5.1.54, atzF | allophanate hydrolase [EC:3.5.1.54] | |
|  | scaffold00002_gene_2091 | K14541 DUR1 | urea carboxylase / allophanate hydrolase [EC:6.3.4.6 3.5.1.54] | |
| 00930 Caprolactam degradation [PATH:ko00930] | | | | |
|  | scaffold00001_gene_11 | K01053 E3.1.1.17, gnl, RGN | gluconolactonase [EC:3.1.1.17] | |
|  | scaffold00002_gene_2786 | K06446 DCAA | acyl-CoA dehydrogenase [EC:1.3.99.-] | |
|  | scaffold00001_gene_94 | K01692 paaF, echA | enoyl-CoA hydratase [EC:4.2.1.17] | |
|  | scaffold00001_gene_201 | K01692 paaF, echA | enoyl-CoA hydratase [EC:4.2.1.17] | |
|  | scaffold00001_gene_210 | K01692 paaF, echA | enoyl-CoA hydratase [EC:4.2.1.17] | |
|  | scaffold00002_gene_2782 | K01692 paaF, echA | enoyl-CoA hydratase [EC:4.2.1.17] | |
|  | scaffold00002_gene_2790 | K01692 paaF, echA | enoyl-CoA hydratase [EC:4.2.1.17] | |
|  | scaffold00002_gene_2819 | K01692 paaF, echA | enoyl-CoA hydratase [EC:4.2.1.17] | |
|  | scaffold00004_gene_4239 | K01692 paaF, echA | enoyl-CoA hydratase [EC:4.2.1.17] | |
|  | scaffold00001_gene_113 | K01782 fadJ | 3-hydroxyacyl-CoA dehydrogenase / enoyl-CoA hydratase / 3-hydroxybutyryl-CoA epimerase [EC:1.1.1.35 4.2.1.17 5.1.2.3] | |
| 00621 Dioxin degradation [PATH:ko00621] | | | | |
|  | scaffold00002_gene_2661 | K00480 E1.14.13.1 | salicylate hydroxylase [EC:1.14.13.1] | |
|  | scaffold00001_gene_298 | K01666 mhpE | 4-hydroxy 2-oxovalerate aldolase [EC:4.1.3.39] | |
|  | scaffold00001_gene_297 | K04073 mhpF | acetaldehyde dehydrogenase [EC:1.2.1.10] | |
| 00626 Naphthalene degradation [PATH:ko00626] | | | | |
|  | scaffold00002_gene_2661 | K00480 E1.14.13.1 | salicylate hydroxylase [EC:1.14.13.1] | |
|  | scaffold00002_gene_1903 | K13953 adhP | alcohol dehydrogenase, propanol-preferring [EC:1.1.1.1] | |
|  | scaffold00002_gene_2882 | K00121 frmA, ADH5, adhC | S-(hydroxymethyl)glutathione dehydrogenase / alcohol dehydrogenase [EC:1.1.1.284 1.1.1.1] | |
|  | scaffold00007_gene_4951 | K00121 frmA, ADH5, adhC | S-(hydroxymethyl)glutathione dehydrogenase / alcohol dehydrogenase [EC:1.1.1.284 1.1.1.1] | |
|  | scaffold00001_gene_699 | K04072 adhE | acetaldehyde dehydrogenase / alcohol dehydrogenase [EC:1.2.1.10 1.1.1.1] | |
| 00624 Polycyclic aromatic hydrocarbon degradation [PATH:ko00624] | | | | |
|  | scaffold00002_gene_2661 | K00480 E1.14.13.1 | salicylate hydroxylase [EC:1.14.13.1] | |
| 00984 Steroid degradation [PATH:ko00984] | | | | |
|  | scaffold00001_gene_644 | K16048 hsaB | 3-hydroxy-9,10-secoandrosta-1,3,5(10)-triene-9,17-dione monooxygenase reductase component [EC:1.5.1.-] | |
|  | scaffold00001_gene_1550 | K16049 hsaC | 3,4-dihydroxy-9,10-secoandrosta-1,3,5(10)-triene-9,17-dione 4,5-dioxygenase [EC:1.13.11.25] | |
| 00980 Metabolism of xenobiotics by cytochrome P450 [PATH:ko00980] | | | | |
|  | scaffold00001_gene_735 | K00799 GST, gst | glutathione S-transferase [EC:2.5.1.18] | |
|  | scaffold00001_gene_1011 | K00799 GST, gst | glutathione S-transferase [EC:2.5.1.18] | |
|  | scaffold00001_gene_1673 | K00799 GST, gst | glutathione S-transferase [EC:2.5.1.18] | |
|  | scaffold00002_gene_1815 | K00799 GST, gst | glutathione S-transferase [EC:2.5.1.18] | |
|  | scaffold00002_gene_1993 | K00799 GST, gst | glutathione S-transferase [EC:2.5.1.18] | |
|  | scaffold00002_gene_2533 | K00799 GST, gst | glutathione S-transferase [EC:2.5.1.18] | |
|  | scaffold00004_gene_4470 | K00799 GST, gst | glutathione S-transferase [EC:2.5.1.18] | |
|  | scaffold00006_gene_4869 | K00799 GST, gst | glutathione S-transferase [EC:2.5.1.18] | |
|  | scaffold00002_gene_2882 | K00121 frmA, ADH5, adhC | S-(hydroxymethyl)glutathione dehydrogenase / alcohol dehydrogenase [EC:1.1.1.284 1.1.1.1] | |
|  | scaffold00007_gene_4951 | K00121 frmA, ADH5, adhC | S-(hydroxymethyl)glutathione dehydrogenase / alcohol dehydrogenase [EC:1.1.1.284 1.1.1.1] | |
|  | scaffold00002_gene_1903 | K13953 adhP | alcohol dehydrogenase, propanol-preferring [EC:1.1.1.1] | |
| 00982 Drug metabolism - cytochrome P450 [PATH:ko00982] | | | | |
|  | scaffold00001_gene_735 | K00799 GST, gst | glutathione S-transferase [EC:2.5.1.18] | |
|  | scaffold00001_gene_1011 | K00799 GST, gst | glutathione S-transferase [EC:2.5.1.18] | |
|  | scaffold00001_gene_1673 | K00799 GST, gst | glutathione S-transferase [EC:2.5.1.18] | |
|  | scaffold00002_gene_1815 | K00799 GST, gst | glutathione S-transferase [EC:2.5.1.18] | |
|  | scaffold00002_gene_1993 | K00799 GST, gst | glutathione S-transferase [EC:2.5.1.18] | |
|  | scaffold00002_gene_2533 | K00799 GST, gst | glutathione S-transferase [EC:2.5.1.18] | |
|  | scaffold00004_gene_4470 | K00799 GST, gst | glutathione S-transferase [EC:2.5.1.18] | |
|  | scaffold00006_gene_4869 | K00799 GST, gst | glutathione S-transferase [EC:2.5.1.18] | |
|  | scaffold00002_gene_2882 | K00121 frmA, ADH5, adhC | S-(hydroxymethyl)glutathione dehydrogenase / alcohol dehydrogenase [EC:1.1.1.284 1.1.1.1] | |
|  | scaffold00007_gene_4951 | K00121 frmA, ADH5, adhC | S-(hydroxymethyl)glutathione dehydrogenase / alcohol dehydrogenase [EC:1.1.1.284 1.1.1.1] | |
|  | scaffold00002_gene_1903 | K13953 adhP | alcohol dehydrogenase, propanol-preferring [EC:1.1.1.1] | |
| 00983 Drug metabolism - other enzymes [PATH:ko00983] | | | | |
|  | scaffold00001_gene_678 | K00088 guaB | IMP dehydrogenase [EC:1.1.1.205] | |
|  | scaffold00001_gene_679 | K01951 guaA, GMPS | GMP synthase (glutamine-hydrolysing) [EC:6.3.5.2] | |
|  | scaffold00001_gene_1324 | K01951 guaA, GMPS | GMP synthase (glutamine-hydrolysing) [EC:6.3.5.2] | |
|  | scaffold00002_gene_1829 | K00569 TPMT, tpmT | thiopurine S-methyltransferase [EC:2.1.1.67] | |

**Table S14 Genomic comparison of strain HZ01 to related species.**

| Strain | Assembly level | Size (Mb) | GC% | Gene | rRNA | tRNA | Pseudogene | Scaffold | Plasmid |
| --- | --- | --- | --- | --- | --- | --- | --- | --- | --- |
| *Achromobacter* sp. HZ01 | Scaffold | 5.53 | 68.1 | 5,162 | 4 | 54 | 31 | 12 | 0 |
| *Achromobacter* sp. RTa | Scaffold | 6.48 | 66.6 | 5,971 | 4 | 54 | 82 | 48 | 0 |
| *Achromobacter xylosoxidans* A8 | Complete genome | 7.36 | 65.79 | 6,858 | 10 | 60 | 37 | 3 | 2 |
| *Achromobacter xylosoxidans* NH44784-1996 | Chromosome | 6.92 | 67.4 | 6,408 | 5 | 54 | 94 | 1 | 0 |
| *Achromobacter denitrificans* USDA-ARS-USMARC-56712 | Complete genome | 6.23 | 67.9 | 5,638 | 10 | 57 | 79 | 1 | 0 |
| *Achromobacter arsenitoxydans* SY8 | Contig | 6.16 | 66 | 5,768 | 4 | 52 | 150 | 105 | 0 |
| *Achromobacter piechaudii* GCS2 | Scaffold | 6.18 | 64.8 | 5,651 | 4 | 53 | 92 | 17 | 0 |
| *Achromobacter insuavis* AXX-A | Scaffold | 6.86 | 67.7 | 6,213 | 4 | 55 | 230 | 13 | 0 |
| *Achromobacter insolitus* DSM23807 | Complete genome | 6.49 | 65.1 | 5,980 | 13 | 57 | 277 | 1 | 0 |
| *Achromobacter ruhlandii* SCCH3:ACH 33-1365 | Chromosome | 6.37 | 67.6 | 5,785 | 10 | 58 | 148 | 1 | 0 |
| *Achromobacter spanius* CGMCC9173 V6_1 | Contig | 6.47 | 63.9 | 5,975 | 10 | 60 | 167 | 98 | 0 |
| *Alcanivorax borkumensis* SK2 | Complete genome | 3.12 | 54.7 | 2,832 | 9 | 42 | 27 | 1 | 0 |
| *Polymorphum gilvum* SL003B-26A1^T^ | Complete genome | 4.72 | 67.12 | 4,466 | 6 | 50 | 50 | 2 | 1 |
| *Geobacillus thermodenitrificans* NG80-2 | Complete genome | 3.61 | 48.85 | 3,649 | 30 | 88 | 130 | 2 | 1 |
| *Desulfatibacillum alkenivorans* AK-01 | Complete genome | 6.52 | 54.50 | 5,442 | 6 | 56 | 36 | 1 | 0 |
| *Oleispira antarctica* RB-8 | Complete genome | 4.41 | 42.20 | 3,987 | 16 | 50 | 51 | 1 | 0 |

**Table S15 Core and pan genome analysis**

| Item | Number |
| --- | --- |
| All gene | 51,465 |
| Pan gene | 12,000 |
| Core gene | 2,643 |
| Dispensable gene | 9,357 |
| Specific gene |  |
| *Achromobacter arsenitoxydans* SY8 | 598 |
| *Achromobacter denitrificans* USDA-ARS-USMARC-56712 | 114 |
| *Achromobacter insolitus* DSM 23807 | 313 |
| *Achromobacter insuavis* AXX-A | 757 |
| *Achromobacter piechaudii* GCS2 | 348 |
| *Achromobacter ruhlandii* SCCH3:ACH 33-1365 | 217 |
| *Achromobacter spanius* CGMCC9173 V6_1 | 632 |
| *Achromobacter* sp. HZ01 | 507 |
| *Achromobacter xylosoxidans* A8 | 905 |

**Table S16** **Genes identified in the “amino acid metabolism” pathway in strain HZ01.**

| Pathway | Predicted gene | Entry/Gene name | Enzyme/Protein |
| --- | --- | --- | --- |
| 00250 Alanine, aspartate and glutamate metabolism [PATH:ko00250] | | | |
|  | scaffold00001_gene_291 | K00278 nadB | L-aspartate oxidase [EC:1.4.3.16] |
|  | scaffold00001_gene_1007 | K01779 racD | aspartate racemase [EC:5.1.1.13] |
|  | scaffold00002_gene_3198 | K01424 E3.5.1.1, ansA, ansB | L-asparaginase [EC:3.5.1.1] |
|  | scaffold00002_gene_2384 | K01953 asnB, ASNS | asparagine synthase (glutamine-hydrolysing) [EC:6.3.5.4] |
|  | scaffold00001_gene_784 | K00830 AGXT | alanine-glyoxylate transaminase / serine-glyoxylate transaminase / serine-pyruvate transaminase [EC:2.6.1.44 2.6.1.45 2.6.1.51] |
|  | scaffold00005_gene_4692 | K01940 argG, ASS1 | argininosuccinate synthase [EC:6.3.4.5] |
|  | scaffold00001_gene_1465 | K01755 argH, ASL | argininosuccinate lyase [EC:4.3.2.1] |
|  | scaffold00007_gene_5031 | K01939 purA, ADSS | adenylosuccinate synthase [EC:6.3.4.4] |
|  | scaffold00005_gene_4651 | K01756 purB, ADSL | adenylosuccinate lyase [EC:4.3.2.2] |
|  | scaffold00004_gene_4044 | K00609 pyrB, PYR2 | aspartate carbamoyltransferase catalytic subunit [EC:2.1.3.2] |
|  | scaffold00005_gene_4601 | K00609 pyrB, PYR2 | aspartate carbamoyltransferase catalytic subunit [EC:2.1.3.2] |
|  | scaffold00001_gene_142 | K00823 puuE | 4-aminobutyrate aminotransferase [EC:2.6.1.19] |
|  | scaffold00001_gene_141 | K00135 gabD | succinate-semialdehyde dehydrogenase / glutarate-semialdehyde dehydrogenase [EC:1.2.1.16 1.2.1.79 1.2.1.20] |
|  | scaffold00004_gene_4511 | K00135 gabD | succinate-semialdehyde dehydrogenase / glutarate-semialdehyde dehydrogenase [EC:1.2.1.16 1.2.1.79 1.2.1.20] |
|  | scaffold00002_gene_2983 | K00265 gltB | glutamate synthase (NADPH/NADH) large chain [EC:1.4.1.13 1.4.1.14] |
|  | scaffold00002_gene_2984 | K00266 gltD | glutamate synthase (NADPH/NADH) small chain [EC:1.4.1.13 1.4.1.14] |
|  | scaffold00001_gene_1305 | K00261 GLUD1_2, gdhA | glutamate dehydrogenase (NAD(P)+) [EC:1.4.1.3] |
|  | scaffold00004_gene_4317 | K00262 E1.4.1.4, gdhA | glutamate dehydrogenase (NADP+) [EC:1.4.1.4] |
|  | scaffold00001_gene_453 | K13821 putA | RHH-type transcriptional regulator, proline utilization regulon repressor / proline dehydrogenase / delta 1-pyrroline-5-carboxylate dehydrogenase [EC:1.5.5.2 1.2.1.88] |
|  | scaffold00001_gene_952 | K01915 glnA, GLUL | glutamine synthetase [EC:6.3.1.2] |
|  | scaffold00002_gene_3209 | K01915 glnA, GLUL | glutamine synthetase [EC:6.3.1.2] |
|  | scaffold00002_gene_1884 | K01955 carB, CPA2 | carbamoyl-phosphate synthase large subunit [EC:6.3.5.5] |
|  | scaffold00002_gene_1885 | K01956 carA, CPA1 | carbamoyl-phosphate synthase small subunit [EC:6.3.5.5] |
|  | scaffold00002_gene_2956 | K00820 glmS, GFPT | glucosamine--fructose-6-phosphate aminotransferase (isomerizing) [EC:2.6.1.16] |
|  | scaffold00001_gene_1353 | K00764 purF, PPAT | amidophosphoribosyltransferase [EC:2.4.2.14] |
| 00260 Glycine, serine and threonine metabolism [PATH:ko00260] | | | |
|  | scaffold00001_gene_80 | K00928 lysC | aspartate kinase [EC:2.7.2.4] |
|  | scaffold00001_gene_1795 | K00133 asd | aspartate-semialdehyde dehydrogenase [EC:1.2.1.11] |
|  | scaffold00005_gene_4629 | K00003 E1.1.1.3 | homoserine dehydrogenase [EC:1.1.1.3] |
|  | scaffold00007_gene_5064 | K02204 thrB2 | homoserine kinase type II [EC:2.7.1.39] |
|  | scaffold00003_gene_3742 | K01733 thrC | threonine synthase [EC:4.2.3.1] |
|  | scaffold00005_gene_4630 | K01733 thrC | threonine synthase [EC:4.2.3.1] |
|  | scaffold00003_gene_3764 | K01620 ltaE | threonine aldolase [EC:4.1.2.48] |
|  | scaffold00002_gene_2501 | K00600 glyA, SHMT | glycine hydroxymethyltransferase [EC:2.1.2.1] |
|  | scaffold00004_gene_3984 | K00600 glyA, SHMT | glycine hydroxymethyltransferase [EC:2.1.2.1] |
|  | scaffold00001_gene_904 | K01079 serB, PSPH | phosphoserine phosphatase [EC:3.1.3.3] |
|  | scaffold00003_gene_3654 | K01079 serB, PSPH | phosphoserine phosphatase [EC:3.1.3.3] |
|  | scaffold00003_gene_3885 | K00831 serC, PSAT1 | phosphoserine aminotransferase [EC:2.6.1.52] |
|  | scaffold00001_gene_386 | K00058 serA, PHGDH | D-3-phosphoglycerate dehydrogenase [EC:1.1.1.95] |
|  | scaffold00001_gene_1165 | K00058 serA, PHGDH | D-3-phosphoglycerate dehydrogenase [EC:1.1.1.95] |
|  | scaffold00002_gene_2733 | K00058 serA, PHGDH | D-3-phosphoglycerate dehydrogenase [EC:1.1.1.95] |
|  | scaffold00002_gene_2749 | K00058 serA, PHGDH | D-3-phosphoglycerate dehydrogenase [EC:1.1.1.95] |
|  | scaffold00003_gene_3294 | K00865 glxK | glycerate kinase [EC:2.7.1.31] |
|  | scaffold00004_gene_4211 | K01834 PGAM, gpmA | 2,3-bisphosphoglycerate-dependent phosphoglycerate mutase [EC:5.4.2.11] |
|  | scaffold00002_gene_1881 | K15634 gpmB | probable phosphoglycerate mutase [EC:5.4.2.12] |
|  | scaffold00001_gene_418 | K15919 HPR2 | hydroxypyruvate reductase 2 |
|  | scaffold00004_gene_3917 | K12972 ghrA | glyoxylate/hydroxypyruvate reductase A [EC:1.1.1.79 1.1.1.81] |
|  | scaffold00002_gene_2406 | K00281 GLDC, gcvP | glycine dehydrogenase [EC:1.4.4.2] |
|  | scaffold00002_gene_2408 | K00605 gcvT, AMT | aminomethyltransferase [EC:2.1.2.10] |
|  | scaffold00001_gene_893 | K00382 DLD, lpd, pdhD | dihydrolipoamide dehydrogenase [EC:1.8.1.4] |
|  | scaffold00001_gene_1420 | K00382 DLD, lpd, pdhD | dihydrolipoamide dehydrogenase [EC:1.8.1.4] |
|  | scaffold00001_gene_1471 | K00382 DLD, lpd, pdhD | dihydrolipoamide dehydrogenase [EC:1.8.1.4] |
|  | scaffold00002_gene_2697 | K00382 DLD, lpd, pdhD | dihydrolipoamide dehydrogenase [EC:1.8.1.4] |
|  | scaffold00003_gene_3822 | K00382 DLD, lpd, pdhD | dihydrolipoamide dehydrogenase [EC:1.8.1.4] |
|  | scaffold00002_gene_2407 | K02437 gcvH, GCSH | glycine cleavage system H protein |
|  | scaffold00001_gene_784 | K00830 AGXT | alanine-glyoxylate transaminase / serine-glyoxylate transaminase / serine-pyruvate transaminase [EC:2.6.1.44 2.6.1.45 2.6.1.51] |
|  | scaffold00003_gene_3536 | K17103 CHO1, pssA | CDP-diacylglycerol---serine O-phosphatidyltransferase [EC:2.7.8.8] |
|  | scaffold00001_gene_587 | K00108 betA, CHDH | choline dehydrogenase [EC:1.1.99.1] |
|  | scaffold00001_gene_788 | K00108 betA, CHDH | choline dehydrogenase [EC:1.1.99.1] |
|  | scaffold00002_gene_2540 | K00130 betB, gbsA | betaine-aldehyde dehydrogenase [EC:1.2.1.8] |
|  | scaffold00002_gene_3138 | K00302 soxA | sarcosine oxidase, subunit alpha [EC:1.5.3.1] |
|  | scaffold00002_gene_3136 | K00303 soxB | sarcosine oxidase, subunit beta [EC:1.5.3.1] |
|  | scaffold00001_gene_309 | K01758 CTH | cystathionine gamma-lyase [EC:4.4.1.1] |
|  | scaffold00005_gene_4554 | K01752 E4.3.1.17, sdaA, sdaB, tdcG | L-serine dehydratase [EC:4.3.1.17] |
|  | scaffold00001_gene_1184 | K01754 E4.3.1.19, ilvA, tdcB | threonine dehydratase [EC:4.3.1.19] |
|  | scaffold00003_gene_3404 | K01754 E4.3.1.19, ilvA, tdcB | threonine dehydratase [EC:4.3.1.19] |
|  | scaffold00003_gene_3541 | K01754 E4.3.1.19, ilvA, tdcB | threonine dehydratase [EC:4.3.1.19] |
|  | scaffold00004_gene_4267 | K01754 E4.3.1.19, ilvA, tdcB | threonine dehydratase [EC:4.3.1.19] |
|  | scaffold00003_gene_3511 | K01753 dsdA | D-serine dehydratase [EC:4.3.1.18] |
|  | scaffold00002_gene_2218 | K01695 trpA | tryptophan synthase alpha chain [EC:4.2.1.20] |
|  | scaffold00003_gene_3708 | K01695 trpA | tryptophan synthase alpha chain [EC:4.2.1.20] |
|  | scaffold00002_gene_2219 | K01696 trpB | tryptophan synthase beta chain [EC:4.2.1.20] |
|  | scaffold00003_gene_3707 | K01696 trpB | tryptophan synthase beta chain [EC:4.2.1.20] |
|  | scaffold00007_gene_4993 | K00836 E2.6.1.76, ectB | diaminobutyrate-2-oxoglutarate transaminase [EC:2.6.1.76] |
|  | scaffold00002_gene_1839 | K15784 doeB | N-alpha-acetyl-L-2,4-diaminobutyrate deacetylase [EC:3.5.1.-] |
| 00270 Cysteine and methionine metabolism [PATH:ko00270] | | | |
|  | scaffold00001_gene_138 | K00640 cysE | serine O-acetyltransferase [EC:2.3.1.30] |
|  | scaffold00003_gene_3898 | K12339 cysM | cysteine synthase B [EC:2.5.1.47] |
|  | scaffold00001_gene_309 | K01758 CTH | cystathionine gamma-lyase [EC:4.4.1.1] |
|  | scaffold00002_gene_3067 | K01760 metC | cystathionine beta-lyase [EC:4.4.1.8] |
|  | scaffold00002_gene_3201 | K01760 metC | cystathionine beta-lyase [EC:4.4.1.8] |
|  | scaffold00002_gene_2712 | K00548 metH, MTR | 5-methyltetrahydrofolate--homocysteine methyltransferase [EC:2.1.1.13] |
|  | scaffold00001_gene_645 | K00549 metE | 5-methyltetrahydropteroyltriglutamate--homocysteine methyltransferase [EC:2.1.1.14] |
|  | scaffold00005_gene_4611 | K00549 metE | 5-methyltetrahydropteroyltriglutamate--homocysteine methyltransferase [EC:2.1.1.14] |
|  | scaffold00004_gene_4378 | K00789 metK | S-adenosylmethionine synthetase [EC:2.5.1.6] |
|  | scaffold00001_gene_227 | K01611 speD, AMD1 | S-adenosylmethionine decarboxylase [EC:4.1.1.50] |
|  | scaffold00001_gene_222 | K00797 speE, SRM | spermidine synthase [EC:2.5.1.16] |
|  | scaffold00001_gene_1096 | K00797 speE, SRM | spermidine synthase [EC:2.5.1.16] |
|  | scaffold00002_gene_2894 | K00797 speE, SRM | spermidine synthase [EC:2.5.1.16] |
|  | scaffold00002_gene_2474 | K01243 mtnN, mtn, pfs | adenosylhomocysteine nucleosidase [EC:3.2.2.9] |
|  | scaffold00001_gene_533 | K00832 tyrB | aromatic-amino-acid transaminase [EC:2.6.1.57] |
|  | scaffold00004_gene_4363 | K01251 E3.3.1.1, ahcY | adenosylhomocysteinase [EC:3.3.1.1] |
|  | scaffold00003_gene_3818 | K01505 E3.5.99.7 | 1-aminocyclopropane-1-carboxylate deaminase [EC:3.5.99.7] |
|  | scaffold00001_gene_80 | K00928 lysC | aspartate kinase [EC:2.7.2.4] |
|  | scaffold00001_gene_1795 | K00133 asd | aspartate-semialdehyde dehydrogenase [EC:1.2.1.11] |
|  | scaffold00005_gene_4629 | K00003 E1.1.1.3 | homoserine dehydrogenase [EC:1.1.1.3] |
|  | scaffold00004_gene_4513 | K01739 metB | cystathionine gamma-synthase [EC:2.5.1.48] |
|  | scaffold00002_gene_2744 | K00641 metX | homoserine O-acetyltransferase [EC:2.3.1.31] |
|  | scaffold00001_gene_1223 | K01740 metY | O-acetylhomoserine (thiol)-lyase [EC:2.5.1.49] |
|  | scaffold00003_gene_3430 | K00826 E2.6.1.42, ilvE | branched-chain amino acid aminotransferase [EC:2.6.1.42] |
|  | scaffold00003_gene_3687 | K00826 E2.6.1.42, ilvE | branched-chain amino acid aminotransferase [EC:2.6.1.42] |
|  | scaffold00002_gene_2755 | K01919 gshA | glutamate--cysteine ligase [EC:6.3.2.2] |
|  | scaffold00001_gene_1769 | K01920 gshB, GSS | glutathione synthase [EC:6.3.2.3] |
|  | scaffold00005_gene_4557 | K01011 TST, MPST, sseA | thiosulfate/3-mercaptopyruvate sulfurtransferase [EC:2.8.1.1 2.8.1.2] |
|  | scaffold00003_gene_3802 | K00024 mdh | malate dehydrogenase [EC:1.1.1.37] |
|  | scaffold00005_gene_4554 | K01752 E4.3.1.17, sdaA, sdaB, tdcG | L-serine dehydratase [EC:4.3.1.17] |
|  | scaffold00001_gene_721 | K01761 E4.4.1.11 | methionine-gamma-lyase [EC:4.4.1.11] |
| 00280 Valine, leucine and isoleucine degradation [PATH:ko00280] | | | |
|  | scaffold00003_gene_3430 | K00826 E2.6.1.42, ilvE | branched-chain amino acid aminotransferase [EC:2.6.1.42] |
|  | scaffold00003_gene_3687 | K00826 E2.6.1.42, ilvE | branched-chain amino acid aminotransferase [EC:2.6.1.42] |
|  | scaffold00001_gene_1474 | K00166 BCKDHA, bkdA1 | 2-oxoisovalerate dehydrogenase E1 component alpha subunit [EC:1.2.4.4] |
|  | scaffold00001_gene_1473 | K00167 BCKDHB, bkdA2 | 2-oxoisovalerate dehydrogenase E1 component beta subunit [EC:1.2.4.4] |
|  | scaffold00001_gene_1472 | K09699 DBT, bkdB | 2-oxoisovalerate dehydrogenase E2 component (dihydrolipoyl transacylase) [EC:2.3.1.168] |
|  | scaffold00001_gene_893 | K00382 DLD, lpd, pdhD | dihydrolipoamide dehydrogenase [EC:1.8.1.4] |
|  | scaffold00001_gene_1420 | K00382 DLD, lpd, pdhD | dihydrolipoamide dehydrogenase [EC:1.8.1.4] |
|  | scaffold00001_gene_1471 | K00382 DLD, lpd, pdhD | dihydrolipoamide dehydrogenase [EC:1.8.1.4] |
|  | scaffold00002_gene_2697 | K00382 DLD, lpd, pdhD | dihydrolipoamide dehydrogenase [EC:1.8.1.4] |
|  | scaffold00003_gene_3822 | K00382 DLD, lpd, pdhD | dihydrolipoamide dehydrogenase [EC:1.8.1.4] |
|  | scaffold00001_gene_947 | K00249 ACADM, acd | acyl-CoA dehydrogenase [EC:1.3.8.7] |
|  | scaffold00001_gene_1360 | K00249 ACADM, acd | acyl-CoA dehydrogenase [EC:1.3.8.7] |
|  | scaffold00002_gene_2552 | K00249 ACADM, acd | acyl-CoA dehydrogenase [EC:1.3.8.7] |
|  | scaffold00004_gene_4064 | K00249 ACADM, acd | acyl-CoA dehydrogenase [EC:1.3.8.7] |
|  | scaffold00004_gene_4183 | K00249 ACADM, acd | acyl-CoA dehydrogenase [EC:1.3.8.7] |
|  | scaffold00006_gene_4791 | K00249 ACADM, acd | acyl-CoA dehydrogenase [EC:1.3.8.7] |
|  | scaffold00002_gene_3074 | K00253 IVD, ivd | isovaleryl-CoA dehydrogenase [EC:1.3.8.4] |
|  | scaffold00001_gene_94 | K01692 paaF, echA | enoyl-CoA hydratase [EC:4.2.1.17] |
|  | scaffold00001_gene_201 | K01692 paaF, echA | enoyl-CoA hydratase [EC:4.2.1.17] |
|  | scaffold00001_gene_210 | K01692 paaF, echA | enoyl-CoA hydratase [EC:4.2.1.17] |
|  | scaffold00002_gene_2782 | K01692 paaF, echA | enoyl-CoA hydratase [EC:4.2.1.17] |
|  | scaffold00002_gene_2790 | K01692 paaF, echA | enoyl-CoA hydratase [EC:4.2.1.17] |
|  | scaffold00002_gene_2819 | K01692 paaF, echA | enoyl-CoA hydratase [EC:4.2.1.17] |
|  | scaffold00004_gene_4239 | K01692 paaF, echA | enoyl-CoA hydratase [EC:4.2.1.17] |
|  | scaffold00001_gene_113 | K01782 fadJ | 3-hydroxyacyl-CoA dehydrogenase / enoyl-CoA hydratase / 3-hydroxybutyryl-CoA epimerase [EC:1.1.1.35 4.2.1.17 5.1.2.3] |
|  | scaffold00004_gene_4238 | K08683 HSD17B10 | 3-hydroxyacyl-CoA dehydrogenase / 3-hydroxy-2-methylbutyryl-CoA dehydrogenase [EC:1.1.1.35 1.1.1.178] |
|  | scaffold00001_gene_110 | K00632 fadA, fadI | acetyl-CoA acyltransferase [EC:2.3.1.16] |
|  | scaffold00001_gene_114 | K00632 fadA, fadI | acetyl-CoA acyltransferase [EC:2.3.1.16] |
|  | scaffold00001_gene_443 | K00632 fadA, fadI | acetyl-CoA acyltransferase [EC:2.3.1.16] |
|  | scaffold00001_gene_730 | K00632 fadA, fadI | acetyl-CoA acyltransferase [EC:2.3.1.16] |
|  | scaffold00001_gene_1315 | K00020 mmsB, HIBADH | 3-hydroxyisobutyrate dehydrogenase [EC:1.1.1.31] |
|  | scaffold00001_gene_1636 | K00020 mmsB, HIBADH | 3-hydroxyisobutyrate dehydrogenase [EC:1.1.1.31] |
|  | scaffold00004_gene_3914 | K00020 mmsB, HIBADH | 3-hydroxyisobutyrate dehydrogenase [EC:1.1.1.31] |
|  | scaffold00002_gene_2397 | K00140 mmsA, iolA, ALDH6A1 | malonate-semialdehyde dehydrogenase (acetylating) / methylmalonate-semialdehyde dehydrogenase [EC:1.2.1.18 1.2.1.27] |
|  | scaffold00003_gene_3754 | K00140 mmsA, iolA, ALDH6A1 | malonate-semialdehyde dehydrogenase (acetylating) / methylmalonate-semialdehyde dehydrogenase [EC:1.2.1.18 1.2.1.27] |
|  | scaffold00001_gene_377 | K00128 E1.2.1.3 | aldehyde dehydrogenase (NAD+) [EC:1.2.1.3] |
|  | scaffold00002_gene_2864 | K00128 E1.2.1.3 | aldehyde dehydrogenase (NAD+) [EC:1.2.1.3] |
|  | scaffold00003_gene_3561 | K00128 E1.2.1.3 | aldehyde dehydrogenase (NAD+) [EC:1.2.1.3] |
|  | scaffold00006_gene_4875 | K00128 E1.2.1.3 | aldehyde dehydrogenase (NAD+) [EC:1.2.1.3] |
|  | scaffold00002_gene_2302 | K00149 ALDH9A1 | aldehyde dehydrogenase family 9 member A1 [EC:1.2.1.47 1.2.1.3] |
|  | scaffold00001_gene_211 | K18661 matB | malonyl-CoA/methylmalonyl-CoA synthetase [EC:6.2.1.-] |
|  | scaffold00002_gene_2396 | K00822 E2.6.1.18 | beta-alanine--pyruvate transaminase [EC:2.6.1.18] |
|  | scaffold00002_gene_3078 | K01968 E6.4.1.4A | 3-methylcrotonyl-CoA carboxylase alpha subunit [EC:6.4.1.4] |
|  | scaffold00002_gene_3077 | K01969 E6.4.1.4B | 3-methylcrotonyl-CoA carboxylase beta subunit [EC:6.4.1.4] |
|  | scaffold00002_gene_2598 | K01640 E4.1.3.4, HMGCL, hmgL | hydroxymethylglutaryl-CoA lyase [EC:4.1.3.4] |
|  | scaffold00004_gene_4257 | K01640 E4.1.3.4, HMGCL, hmgL | hydroxymethylglutaryl-CoA lyase [EC:4.1.3.4] |
|  | scaffold00003_gene_3769 | K01028 E2.8.3.5A, scoA | 3-oxoacid CoA-transferase subunit A [EC:2.8.3.5] |
|  | scaffold00003_gene_3770 | K01029 E2.8.3.5B, scoB | 3-oxoacid CoA-transferase subunit B [EC:2.8.3.5] |
|  | scaffold00001_gene_199 | K00626 E2.3.1.9, atoB | acetyl-CoA C-acetyltransferase [EC:2.3.1.9] |
|  | scaffold00002_gene_2940 | K00626 E2.3.1.9, atoB | acetyl-CoA C-acetyltransferase [EC:2.3.1.9] |
|  | scaffold00002_gene_3076 | K00626 E2.3.1.9, atoB | acetyl-CoA C-acetyltransferase [EC:2.3.1.9] |
|  | scaffold00006_gene_4805 | K00626 E2.3.1.9, atoB | acetyl-CoA C-acetyltransferase [EC:2.3.1.9] |
| 00290 Valine, leucine and isoleucine biosynthesis [PATH:ko00290] | | | |
|  | scaffold00001_gene_1184 | K01754 E4.3.1.19, ilvA, tdcB | threonine dehydratase [EC:4.3.1.19] |
|  | scaffold00003_gene_3404 | K01754 E4.3.1.19, ilvA, tdcB | threonine dehydratase [EC:4.3.1.19] |
|  | scaffold00003_gene_3541 | K01754 E4.3.1.19, ilvA, tdcB | threonine dehydratase [EC:4.3.1.19] |
|  | scaffold00004_gene_4267 | K01754 E4.3.1.19, ilvA, tdcB | threonine dehydratase [EC:4.3.1.19] |
|  | scaffold00001_gene_1798 | K01703 leuC | 3-isopropylmalate/(R)-2-methylmalate dehydratase large subunit [EC:4.2.1.33 4.2.1.35] |
|  | scaffold00002_gene_3099 | K01703 leuC | 3-isopropylmalate/(R)-2-methylmalate dehydratase large subunit [EC:4.2.1.33 4.2.1.35] |
|  | scaffold00001_gene_1797 | K01704 leuD | 3-isopropylmalate/(R)-2-methylmalate dehydratase small subunit [EC:4.2.1.33 4.2.1.35] |
|  | scaffold00002_gene_3098 | K01704 leuD | 3-isopropylmalate/(R)-2-methylmalate dehydratase small subunit [EC:4.2.1.33 4.2.1.35] |
|  | scaffold00001_gene_1796 | K00052 leuB | 3-isopropylmalate dehydrogenase [EC:1.1.1.85] |
|  | scaffold00002_gene_1908 | K01652 E2.2.1.6L, ilvB, ilvG, ilvI | acetolactate synthase I/II/III large subunit [EC:2.2.1.6] |
|  | scaffold00002_gene_1924 | K01652 E2.2.1.6L, ilvB, ilvG, ilvI | acetolactate synthase I/II/III large subunit [EC:2.2.1.6] |
|  | scaffold00002_gene_2609 | K01652 E2.2.1.6L, ilvB, ilvG, ilvI | acetolactate synthase I/II/III large subunit [EC:2.2.1.6] |
|  | scaffold00002_gene_3119 | K01652 E2.2.1.6L, ilvB, ilvG, ilvI | acetolactate synthase I/II/III large subunit [EC:2.2.1.6] |
|  | scaffold00003_gene_3239 | K01652 E2.2.1.6L, ilvB, ilvG, ilvI | acetolactate synthase I/II/III large subunit [EC:2.2.1.6] |
|  | scaffold00003_gene_3533 | K01652 E2.2.1.6L, ilvB, ilvG, ilvI | acetolactate synthase I/II/III large subunit [EC:2.2.1.6] |
|  | scaffold00003_gene_3534 | K01653 E2.2.1.6S, ilvH, ilvN | acetolactate synthase I/III small subunit [EC:2.2.1.6] |
|  | scaffold00003_gene_3535 | K00053 ilvC | ketol-acid reductoisomerase [EC:1.1.1.86] |
|  | scaffold00001_gene_424 | K01687 ilvD | dihydroxy-acid dehydratase [EC:4.2.1.9] |
|  | scaffold00001_gene_1779 | K01687 ilvD | dihydroxy-acid dehydratase [EC:4.2.1.9] |
|  | scaffold00002_gene_2699 | K01687 ilvD | dihydroxy-acid dehydratase [EC:4.2.1.9] |
|  | scaffold00004_gene_3906 | K01687 ilvD | dihydroxy-acid dehydratase [EC:4.2.1.9] |
|  | scaffold00003_gene_3430 | K00826 E2.6.1.42, ilvE | branched-chain amino acid aminotransferase [EC:2.6.1.42] |
|  | scaffold00003_gene_3687 | K00826 E2.6.1.42, ilvE | branched-chain amino acid aminotransferase [EC:2.6.1.42] |
|  | scaffold00002_gene_3052 | K01649 leuA | 2-isopropylmalate synthase [EC:2.3.3.13] |
| 00300 Lysine biosynthesis [PATH:ko00300] | | | |
|  | scaffold00005_gene_4629 | K00003 E1.1.1.3 | homoserine dehydrogenase [EC:1.1.1.3] |
|  | scaffold00001_gene_80 | K00928 lysC | aspartate kinase [EC:2.7.2.4] |
|  | scaffold00001_gene_1795 | K00133 asd | aspartate-semialdehyde dehydrogenase [EC:1.2.1.11] |
|  | scaffold00001_gene_38 | K01714 dapA | 4-hydroxy-tetrahydrodipicolinate synthase [EC:4.3.3.7] |
|  | scaffold00002_gene_2046 | K01714 dapA | 4-hydroxy-tetrahydrodipicolinate synthase [EC:4.3.3.7] |
|  | scaffold00002_gene_2513 | K01714 dapA | 4-hydroxy-tetrahydrodipicolinate synthase [EC:4.3.3.7] |
|  | scaffold00003_gene_3484 | K01714 dapA | 4-hydroxy-tetrahydrodipicolinate synthase [EC:4.3.3.7] |
|  | scaffold00004_gene_4449 | K01714 dapA | 4-hydroxy-tetrahydrodipicolinate synthase [EC:4.3.3.7] |
|  | scaffold00003_gene_3459 | K00215 dapB | 4-hydroxy-tetrahydrodipicolinate reductase [EC:1.17.1.8] |
|  | scaffold00001_gene_501 | K00674 dapD | 2,3,4,5-tetrahydropyridine-2-carboxylate N-succinyltransferase [EC:2.3.1.117] |
|  | scaffold00002_gene_2803 | K00674 dapD | 2,3,4,5-tetrahydropyridine-2-carboxylate N-succinyltransferase [EC:2.3.1.117] |
|  | scaffold00002_gene_3106 | K00821 argD | acetylornithine/N-succinyldiaminopimelate aminotransferase [EC:2.6.1.11 2.6.1.17] |
|  | scaffold00001_gene_502 | K14267 dapC | N-succinyldiaminopimelate aminotransferase [EC:2.6.1.17] |
|  | scaffold00001_gene_500 | K01439 dapE | succinyl-diaminopimelate desuccinylase [EC:3.5.1.18] |
|  | scaffold00003_gene_3815 | K01439 dapE | succinyl-diaminopimelate desuccinylase [EC:3.5.1.18] |
|  | scaffold00004_gene_4381 | K01778 dapF | diaminopimelate epimerase [EC:5.1.1.7] |
|  | scaffold00001_gene_427 | K01586 lysA | diaminopimelate decarboxylase [EC:4.1.1.20] |
|  | scaffold00004_gene_4476 | K01586 lysA | diaminopimelate decarboxylase [EC:4.1.1.20] |
|  | scaffold00001_gene_23 | K01655 LYS21, LYS20 | homocitrate synthase [EC:2.3.3.14] |
|  | scaffold00004_gene_4452 | K05825 LYSN | 2-aminoadipate transaminase [EC:2.6.1.-] |
|  | scaffold00003_gene_3792 | K00290 LYS1 | saccharopine dehydrogenase (NAD+, L-lysine forming) [EC:1.5.1.7] |
|  | scaffold00003_gene_3222 | K01928 murE | UDP-N-acetylmuramoyl-L-alanyl-D-glutamate--2,6-diaminopimelate ligase [EC:6.3.2.13] |
| 00310 Lysine degradation [PATH:ko00310] | | | |
|  | scaffold00003_gene_3792 | K00290 LYS1 | saccharopine dehydrogenase (NAD+, L-lysine forming) [EC:1.5.1.7] |
|  | scaffold00003_gene_3820 | K00164 OGDH, sucA | 2-oxoglutarate dehydrogenase E1 component [EC:1.2.4.2] |
|  | scaffold00003_gene_3821 | K00658 DLST, sucB | 2-oxoglutarate dehydrogenase E2 component (dihydrolipoamide succinyltransferase) [EC:2.3.1.61] |
|  | scaffold00002_gene_1909 | K00252 GCDH, gcdH | glutaryl-CoA dehydrogenase [EC:1.3.8.6] |
|  | scaffold00001_gene_94 | K01692 paaF, echA | enoyl-CoA hydratase [EC:4.2.1.17] |
|  | scaffold00001_gene_201 | K01692 paaF, echA | enoyl-CoA hydratase [EC:4.2.1.17] |
|  | scaffold00001_gene_210 | K01692 paaF, echA | enoyl-CoA hydratase [EC:4.2.1.17] |
|  | scaffold00002_gene_2782 | K01692 paaF, echA | enoyl-CoA hydratase [EC:4.2.1.17] |
|  | scaffold00002_gene_2790 | K01692 paaF, echA | enoyl-CoA hydratase [EC:4.2.1.17] |
|  | scaffold00002_gene_2819 | K01692 paaF, echA | enoyl-CoA hydratase [EC:4.2.1.17] |
|  | scaffold00004_gene_4239 | K01692 paaF, echA | enoyl-CoA hydratase [EC:4.2.1.17] |
|  | scaffold00001_gene_113 | K01782 fadJ | 3-hydroxyacyl-CoA dehydrogenase / enoyl-CoA hydratase / 3-hydroxybutyryl-CoA epimerase [EC:1.1.1.35 4.2.1.17 5.1.2.3] |
|  | scaffold00001_gene_199 | K00626 E2.3.1.9, atoB | acetyl-CoA C-acetyltransferase [EC:2.3.1.9] |
|  | scaffold00002_gene_2940 | K00626 E2.3.1.9, atoB | acetyl-CoA C-acetyltransferase [EC:2.3.1.9] |
|  | scaffold00002_gene_3076 | K00626 E2.3.1.9, atoB | acetyl-CoA C-acetyltransferase [EC:2.3.1.9] |
|  | scaffold00006_gene_4805 | K00626 E2.3.1.9, atoB | acetyl-CoA C-acetyltransferase [EC:2.3.1.9] |
|  | scaffold00001_gene_141 | K00135 gabD | succinate-semialdehyde dehydrogenase / glutarate-semialdehyde dehydrogenase [EC:1.2.1.16 1.2.1.79 1.2.1.20] |
|  | scaffold00004_gene_4511 | K00135 gabD | succinate-semialdehyde dehydrogenase / glutarate-semialdehyde dehydrogenase [EC:1.2.1.16 1.2.1.79 1.2.1.20] |
|  | scaffold00001_gene_1188 | K03897 iucD | lysine N6-hydroxylase [EC:1.14.13.59] |
|  | scaffold00004_gene_4109 | K01034 atoD | acetate CoA/acetoacetate CoA-transferase alpha subunit [EC:2.8.3.8 2.8.3.9] |
|  | scaffold00002_gene_1933 | K01035 atoA | acetate CoA/acetoacetate CoA-transferase beta subunit [EC:2.8.3.8 2.8.3.9] |
|  | scaffold00004_gene_4408 | K00824 dat | D-alanine transaminase [EC:2.6.1.21] |
|  | scaffold00002_gene_3122 | K09186 MLL1 | histone-lysine N-methyltransferase MLL1 [EC:2.1.1.43] |
|  | scaffold00001_gene_377 | K00128 E1.2.1.3 | aldehyde dehydrogenase (NAD+) [EC:1.2.1.3] |
|  | scaffold00002_gene_2864 | K00128 E1.2.1.3 | aldehyde dehydrogenase (NAD+) [EC:1.2.1.3] |
|  | scaffold00003_gene_3561 | K00128 E1.2.1.3 | aldehyde dehydrogenase (NAD+) [EC:1.2.1.3] |
|  | scaffold00006_gene_4875 | K00128 E1.2.1.3 | aldehyde dehydrogenase (NAD+) [EC:1.2.1.3] |
|  | scaffold00002_gene_2302 | K00149 ALDH9A1 | aldehyde dehydrogenase family 9 member A1 [EC:1.2.1.47 1.2.1.3] |
| 00220 Arginine biosynthesis [PATH:ko00220] | | | |
|  | scaffold00005_gene_4698 | K00611 OTC, argF, argI | ornithine carbamoyltransferase [EC:2.1.3.3] |
|  | scaffold00005_gene_4692 | K01940 argG, ASS1 | argininosuccinate synthase [EC:6.3.4.5] |
|  | scaffold00001_gene_1465 | K01755 argH, ASL | argininosuccinate lyase [EC:4.3.2.1] |
|  | scaffold00001_gene_1020 | K01476 E3.5.3.1, rocF, arg | arginase [EC:3.5.3.1] |
|  | scaffold00002_gene_2092 | K01457 E3.5.1.54, atzF | allophanate hydrolase [EC:3.5.1.54] |
|  | scaffold00002_gene_2091 | K14541 DUR1 | urea carboxylase / allophanate hydrolase [EC:6.3.4.6 3.5.1.54] |
|  | scaffold00001_gene_952 | K01915 glnA, GLUL | glutamine synthetase [EC:6.3.1.2] |
|  | scaffold00002_gene_3209 | K01915 glnA, GLUL | glutamine synthetase [EC:6.3.1.2] |
|  | scaffold00001_gene_1305 | K00261 GLUD1_2, gdhA | glutamate dehydrogenase (NAD(P)+) [EC:1.4.1.3] |
|  | scaffold00004_gene_4317 | K00262 E1.4.1.4, gdhA | glutamate dehydrogenase (NADP+) [EC:1.4.1.4] |
|  | scaffold00005_gene_4724 | K14682 argAB | amino-acid N-acetyltransferase [EC:2.3.1.1] |
|  | scaffold00004_gene_4081 | K00620 argJ | glutamate N-acetyltransferase / amino-acid N-acetyltransferase [EC:2.3.1.35 2.3.1.1] |
|  | scaffold00004_gene_4298 | K00930 argB | acetylglutamate kinase [EC:2.7.2.8] |
|  | scaffold00001_gene_1213 | K00145 argC | N-acetyl-gamma-glutamyl-phosphate reductase [EC:1.2.1.38] |
|  | scaffold00004_gene_3991 | K00145 argC | N-acetyl-gamma-glutamyl-phosphate reductase [EC:1.2.1.38] |
|  | scaffold00005_gene_4699 | K00818 E2.6.1.11, argD | acetylornithine aminotransferase [EC:2.6.1.11] |
|  | scaffold00002_gene_3106 | K00821 argD | acetylornithine/N-succinyldiaminopimelate aminotransferase [EC:2.6.1.11 2.6.1.17] |
|  | scaffold00003_gene_3706 | K01438 argE | acetylornithine deacetylase [EC:3.5.1.16] |
| 00330 Arginine and proline metabolism [PATH:ko00330] | | | |
|  | scaffold00002_gene_3134 | K01485 codA | cytosine deaminase [EC:3.5.4.1] |
|  | scaffold00002_gene_3146 | K01473 hyuA | N-methylhydantoinase A [EC:3.5.2.14] |
|  | scaffold00002_gene_3145 | K01474 hyuB | N-methylhydantoinase B [EC:3.5.2.14] |
|  | scaffold00002_gene_2412 | K01584 adiA | arginine decarboxylase [EC:4.1.1.19] |
|  | scaffold00002_gene_2889 | K12251 aguB | N-carbamoylputrescine amidase [EC:3.5.1.53] |
|  | scaffold00001_gene_222 | K00797 speE, SRM | spermidine synthase [EC:2.5.1.16] |
|  | scaffold00001_gene_1096 | K00797 speE, SRM | spermidine synthase [EC:2.5.1.16] |
|  | scaffold00002_gene_2894 | K00797 speE, SRM | spermidine synthase [EC:2.5.1.16] |
|  | scaffold00001_gene_227 | K01611 speD, AMD1 | S-adenosylmethionine decarboxylase [EC:4.1.1.50] |
|  | scaffold00003_gene_3793 | K13747 nspC | carboxynorspermidine decarboxylase [EC:4.1.1.96] |
|  | scaffold00001_gene_377 | K00128 E1.2.1.3 | aldehyde dehydrogenase (NAD+) [EC:1.2.1.3] |
|  | scaffold00002_gene_2864 | K00128 E1.2.1.3 | aldehyde dehydrogenase (NAD+) [EC:1.2.1.3] |
|  | scaffold00003_gene_3561 | K00128 E1.2.1.3 | aldehyde dehydrogenase (NAD+) [EC:1.2.1.3] |
|  | scaffold00006_gene_4875 | K00128 E1.2.1.3 | aldehyde dehydrogenase (NAD+) [EC:1.2.1.3] |
|  | scaffold00002_gene_2302 | K00149 ALDH9A1 | aldehyde dehydrogenase family 9 member A1 [EC:1.2.1.47 1.2.1.3] |
|  | scaffold00001_gene_1488 | K00657 speG | diamine N-acetyltransferase [EC:2.3.1.57] |
|  | scaffold00001_gene_712 | K01426 E3.5.1.4, amiE | amidase [EC:3.5.1.4] |
|  | scaffold00001_gene_1579 | K01426 E3.5.1.4, amiE | amidase [EC:3.5.1.4] |
|  | scaffold00002_gene_2403 | K01426 E3.5.1.4, amiE | amidase [EC:3.5.1.4] |
|  | scaffold00002_gene_2828 | K01426 E3.5.1.4, amiE | amidase [EC:3.5.1.4] |
|  | scaffold00005_gene_4718 | K01426 E3.5.1.4, amiE | amidase [EC:3.5.1.4] |
|  | scaffold00006_gene_4880 | K09471 puuB, ordL | gamma-glutamylputrescine oxidase [EC:1.4.3.-] |
|  | scaffold00002_gene_2047 | K09472 puuC, aldH | gamma-glutamyl-gamma-aminobutyraldehyde dehydrogenase [EC:1.2.1.-] |
|  | scaffold00001_gene_1020 | K01476 E3.5.3.1, rocF, arg | arginase [EC:3.5.3.1] |
|  | scaffold00001_gene_1021 | K00819 rocD, OAT | ornithine--oxo-acid transaminase [EC:2.6.1.13] |
|  | scaffold00001_gene_996 | K00286 proC | pyrroline-5-carboxylate reductase [EC:1.5.1.2] |
|  | scaffold00002_gene_2636 | K00286 proC | pyrroline-5-carboxylate reductase [EC:1.5.1.2] |
|  | scaffold00001_gene_453 | K13821 putA | RHH-type transcriptional regulator, proline utilization regulon repressor / proline dehydrogenase / delta 1-pyrroline-5-carboxylate dehydrogenase [EC:1.5.5.2 1.2.1.88] |
|  | scaffold00002_gene_2509 | K01750 E4.3.1.12, ocd | ornithine cyclodeaminase [EC:4.3.1.12] |
|  | scaffold00002_gene_2890 | K01750 E4.3.1.12, ocd | ornithine cyclodeaminase [EC:4.3.1.12] |
|  | scaffold00004_gene_4200 | K00931 proB | glutamate 5-kinase [EC:2.7.2.11] |
|  | scaffold00006_gene_4760 | K00147 proA | glutamate-5-semialdehyde dehydrogenase [EC:1.2.1.41] |
|  | scaffold00002_gene_2526 | K01259 pip | proline iminopeptidase [EC:3.4.11.5] |
|  | scaffold00004_gene_4408 | K00824 dat | D-alanine transaminase [EC:2.6.1.21] |
| 00340 Histidine metabolism [PATH:ko00340] | | | |
|  | scaffold00002_gene_2999 | K00765 hisG | ATP phosphoribosyltransferase [EC:2.4.2.17] |
|  | scaffold00007_gene_5030 | K02502 hisZ | ATP phosphoribosyltransferase regulatory subunit |
|  | scaffold00002_gene_3007 | K01523 hisE | phosphoribosyl-ATP pyrophosphohydrolase [EC:3.6.1.31] |
|  | scaffold00002_gene_3006 | K01496 hisI | phosphoribosyl-AMP cyclohydrolase [EC:3.5.4.19] |
|  | scaffold00002_gene_3004 | K01814 hisA | phosphoribosylformimino-5-aminoimidazole carboxamide ribotide isomerase [EC:5.3.1.16] |
|  | scaffold00002_gene_3003 | K02501 hisH | glutamine amidotransferase [EC:2.4.2.-] |
|  | scaffold00002_gene_3005 | K02500 hisF | cyclase [EC:4.1.3.-] |
|  | scaffold00002_gene_3002 | K01693 hisB | imidazoleglycerol-phosphate dehydratase [EC:4.2.1.19] |
|  | scaffold00002_gene_3001 | K00817 hisC | histidinol-phosphate aminotransferase [EC:2.6.1.9] |
|  | scaffold00003_gene_3887 | K00817 hisC | histidinol-phosphate aminotransferase [EC:2.6.1.9] |
|  | scaffold00007_gene_5025 | K00817 hisC | histidinol-phosphate aminotransferase [EC:2.6.1.9] |
|  | scaffold00002_gene_3208 | K18649 IMPL2 | inositol-phosphate phosphatase / L-galactose 1-phosphate phosphatase / histidinol-phosphatase [EC:3.1.3.25 3.1.3.93 3.1.3.15] |
|  | scaffold00002_gene_3000 | K00013 hisD | histidinol dehydrogenase [EC:1.1.1.23] |
|  | scaffold00001_gene_1572 | K01745 hutH, HAL | histidine ammonia-lyase [EC:4.3.1.3] |
|  | scaffold00002_gene_2879 | K01745 hutH, HAL | histidine ammonia-lyase [EC:4.3.1.3] |
|  | scaffold00002_gene_2511 | K01712 hutU, UROC1 | urocanate hydratase [EC:4.2.1.49] |
|  | scaffold00002_gene_2878 | K01468 hutI, AMDHD1 | imidazolonepropionase [EC:3.5.2.7] |
|  | scaffold00001_gene_1306 | K01479 hutG | formiminoglutamase [EC:3.5.3.8] |
|  | scaffold00001_gene_1515 | K01479 hutG | formiminoglutamase [EC:3.5.3.8] |
|  | scaffold00001_gene_377 | K00128 E1.2.1.3 | aldehyde dehydrogenase (NAD+) [EC:1.2.1.3] |
|  | scaffold00002_gene_2864 | K00128 E1.2.1.3 | aldehyde dehydrogenase (NAD+) [EC:1.2.1.3] |
|  | scaffold00003_gene_3561 | K00128 E1.2.1.3 | aldehyde dehydrogenase (NAD+) [EC:1.2.1.3] |
|  | scaffold00006_gene_4875 | K00128 E1.2.1.3 | aldehyde dehydrogenase (NAD+) [EC:1.2.1.3] |
|  | scaffold00002_gene_2302 | K00149 ALDH9A1 | aldehyde dehydrogenase family 9 member A1 [EC:1.2.1.47 1.2.1.3] |
|  | scaffold00005_gene_4671 | K18911 egtD | L-histidine Nalpha-methyltransferase [EC:2.1.1.44] |
|  | scaffold00005_gene_4670 | K18912 egtB | gamma-glutamyl hercynylcysteine S-oxide synthase [EC:1.14.99.50] |
|  | scaffold00002_gene_2804 | K07008 egtC | gamma-glutamyl hercynylcysteine S-oxide hydrolase [EC:3.5.1.118] |
| 00350 Tyrosine metabolism [PATH:ko00350] | | | |
|  | scaffold00002_gene_3001 | K00817 hisC | histidinol-phosphate aminotransferase [EC:2.6.1.9] |
|  | scaffold00003_gene_3887 | K00817 hisC | histidinol-phosphate aminotransferase [EC:2.6.1.9] |
|  | scaffold00007_gene_5025 | K00817 hisC | histidinol-phosphate aminotransferase [EC:2.6.1.9] |
|  | scaffold00001_gene_533 | K00832 tyrB | aromatic-amino-acid transaminase [EC:2.6.1.57] |
|  | scaffold00004_gene_3903 | K00457 HPD, hppD | 4-hydroxyphenylpyruvate dioxygenase [EC:1.13.11.27] |
|  | scaffold00002_gene_2369 | K00451 HGD, hmgA | homogentisate 1,2-dioxygenase [EC:1.13.11.5] |
|  | scaffold00002_gene_2657 | K01800 maiA, GSTZ1 | maleylacetoacetate isomerase [EC:5.2.1.2] |
|  | scaffold00002_gene_2370 | K01555 FAH, fahA | fumarylacetoacetase [EC:3.7.1.2] |
|  | scaffold00001_gene_1122 | K16171 faaH | fumarylacetoacetate (FAA) hydrolase [EC:3.7.1.2] |
|  | scaffold00002_gene_2882 | K00121 frmA, ADH5, adhC | S-(hydroxymethyl)glutathione dehydrogenase / alcohol dehydrogenase [EC:1.1.1.284 1.1.1.1] |
|  | scaffold00007_gene_4951 | K00121 frmA, ADH5, adhC | S-(hydroxymethyl)glutathione dehydrogenase / alcohol dehydrogenase [EC:1.1.1.284 1.1.1.1] |
|  | scaffold00001_gene_699 | K04072 adhE | acetaldehyde dehydrogenase / alcohol dehydrogenase [EC:1.2.1.10 1.1.1.1] |
|  | scaffold00002_gene_1903 | K13953 adhP | alcohol dehydrogenase, propanol-preferring [EC:1.1.1.1] |
|  | scaffold00002_gene_1944 | K00455 hpaD, hpcB | 3,4-dihydroxyphenylacetate 2,3-dioxygenase [EC:1.13.11.15] |
|  | scaffold00002_gene_1946 | K00151 hpaE, hpcC | 5-carboxymethyl-2-hydroxymuconic-semialdehyde dehydrogenase [EC:1.2.1.60] |
|  | scaffold00002_gene_1948 | K05921 hpaG | 5-oxopent-3-ene-1,2,5-tricarboxylate decarboxylase / 2-hydroxyhepta-2,4-diene-1,7-dioate isomerase [EC:4.1.1.68 5.3.3.-] |
|  | scaffold00001_gene_636 | K02509 hpaH | 2-oxo-hept-3-ene-1,7-dioate hydratase [EC:4.2.1.-] |
|  | scaffold00002_gene_1942 | K02509 hpaH | 2-oxo-hept-3-ene-1,7-dioate hydratase [EC:4.2.1.-] |
|  | scaffold00001_gene_412 | K02510 hpaI, hpcH | 4-hydroxy-2-oxoheptanedioate aldolase [EC:4.1.2.52] |
|  | scaffold00001_gene_635 | K02510 hpaI, hpcH | 4-hydroxy-2-oxoheptanedioate aldolase [EC:4.1.2.52] |
|  | scaffold00002_gene_1941 | K02510 hpaI, hpcH | 4-hydroxy-2-oxoheptanedioate aldolase [EC:4.1.2.52] |
|  | scaffold00001_gene_141 | K00135 gabD | succinate-semialdehyde dehydrogenase / glutarate-semialdehyde dehydrogenase [EC:1.2.1.16 1.2.1.79 1.2.1.20] |
|  | scaffold00004_gene_4511 | K00135 gabD | succinate-semialdehyde dehydrogenase / glutarate-semialdehyde dehydrogenase [EC:1.2.1.16 1.2.1.79 1.2.1.20] |
|  | scaffold00002_gene_2653 | K00450 E1.13.11.4 | gentisate 1,2-dioxygenase [EC:1.13.11.4] |
|  | scaffold00008_gene_5130 | K01801 nagL | maleylpyruvate isomerase [EC:5.2.1.4] |
|  | scaffold00005_gene_4623 | K01557 FAHD1 | acylpyruvate hydrolase [EC:3.7.1.5] |
|  | scaffold00002_gene_2654 | K16165 nagK | fumarylpyruvate hydrolase [EC:3.7.1.20] |
| 00360 Phenylalanine metabolism [PATH:ko00360] | | | |
|  | scaffold00007_gene_4971 | K00146 feaB | phenylacetaldehyde dehydrogenase [EC:1.2.1.39] |
|  | scaffold00008_gene_5121 | K01912 paaK | phenylacetate-CoA ligase [EC:6.2.1.30] |
|  | scaffold00008_gene_5120 | K02614 paaI | acyl-CoA thioesterase [EC:3.1.2.-] |
|  | scaffold00008_gene_5113 | K02609 paaA | ring-1,2-phenylacetyl-CoA epoxidase subunit PaaA [EC:1.14.13.149] |
|  | scaffold00008_gene_5114 | K02610 paaB | ring-1,2-phenylacetyl-CoA epoxidase subunit PaaB |
|  | scaffold00008_gene_5115 | K02611 paaC | ring-1,2-phenylacetyl-CoA epoxidase subunit PaaC [EC:1.14.13.149] |
|  | scaffold00008_gene_5116 | K02612 paaD | ring-1,2-phenylacetyl-CoA epoxidase subunit PaaD |
|  | scaffold00008_gene_5117 | K02613 paaE | ring-1,2-phenylacetyl-CoA epoxidase subunit PaaE |
|  | scaffold00001_gene_1116 | K15866 paaG | 2-(1,2-epoxy-1,2-dihydrophenyl)acetyl-CoA isomerase [EC:5.3.3.18] |
|  | scaffold00008_gene_5119 | K15866 paaG | 2-(1,2-epoxy-1,2-dihydrophenyl)acetyl-CoA isomerase [EC:5.3.3.18] |
|  | scaffold00001_gene_94 | K01692 paaF, echA | enoyl-CoA hydratase [EC:4.2.1.17] |
|  | scaffold00001_gene_201 | K01692 paaF, echA | enoyl-CoA hydratase [EC:4.2.1.17] |
|  | scaffold00001_gene_210 | K01692 paaF, echA | enoyl-CoA hydratase [EC:4.2.1.17] |
|  | scaffold00002_gene_2782 | K01692 paaF, echA | enoyl-CoA hydratase [EC:4.2.1.17] |
|  | scaffold00002_gene_2790 | K01692 paaF, echA | enoyl-CoA hydratase [EC:4.2.1.17] |
|  | scaffold00002_gene_2819 | K01692 paaF, echA | enoyl-CoA hydratase [EC:4.2.1.17] |
|  | scaffold00004_gene_4239 | K01692 paaF, echA | enoyl-CoA hydratase [EC:4.2.1.17] |
|  | scaffold00002_gene_2784 | K00074 paaH, hbd, fadB, mmgB | 3-hydroxybutyryl-CoA dehydrogenase [EC:1.1.1.157] |
|  | scaffold00004_gene_4105 | K00074 paaH, hbd, fadB, mmgB | 3-hydroxybutyryl-CoA dehydrogenase [EC:1.1.1.157] |
|  | scaffold00002_gene_3001 | K00817 hisC | histidinol-phosphate aminotransferase [EC:2.6.1.9] |
|  | scaffold00003_gene_3887 | K00817 hisC | histidinol-phosphate aminotransferase [EC:2.6.1.9] |
|  | scaffold00007_gene_5025 | K00817 hisC | histidinol-phosphate aminotransferase [EC:2.6.1.9] |
|  | scaffold00001_gene_533 | K00832 tyrB | aromatic-amino-acid transaminase [EC:2.6.1.57] |
|  | scaffold00004_gene_3903 | K00457 HPD, hppD | 4-hydroxyphenylpyruvate dioxygenase [EC:1.13.11.27] |
|  | scaffold00001_gene_220 | K00285 dadA | D-amino-acid dehydrogenase [EC:1.4.5.1] |
|  | scaffold00001_gene_303 | K00285 dadA | D-amino-acid dehydrogenase [EC:1.4.5.1] |
|  | scaffold00001_gene_846 | K00285 dadA | D-amino-acid dehydrogenase [EC:1.4.5.1] |
|  | scaffold00001_gene_1684 | K00285 dadA | D-amino-acid dehydrogenase [EC:1.4.5.1] |
|  | scaffold00004_gene_4408 | K00824 dat | D-alanine transaminase [EC:2.6.1.21] |
|  | scaffold00001_gene_712 | K01426 E3.5.1.4, amiE | amidase [EC:3.5.1.4] |
|  | scaffold00001_gene_1579 | K01426 E3.5.1.4, amiE | amidase [EC:3.5.1.4] |
|  | scaffold00002_gene_2403 | K01426 E3.5.1.4, amiE | amidase [EC:3.5.1.4] |
|  | scaffold00002_gene_2828 | K01426 E3.5.1.4, amiE | amidase [EC:3.5.1.4] |
|  | scaffold00005_gene_4718 | K01426 E3.5.1.4, amiE | amidase [EC:3.5.1.4] |
|  | scaffold00001_gene_792 | K01451 hipO | hippurate hydrolase [EC:3.5.1.32] |
|  | scaffold00002_gene_3073 | K01451 hipO | hippurate hydrolase [EC:3.5.1.32] |
|  | scaffold00002_gene_3172 | K01451 hipO | hippurate hydrolase [EC:3.5.1.32] |
|  | scaffold00003_gene_3215 | K01451 hipO | hippurate hydrolase [EC:3.5.1.32] |
|  | scaffold00003_gene_3246 | K01451 hipO | hippurate hydrolase [EC:3.5.1.32] |
|  | scaffold00001_gene_1751 | K05708 hcaE, hcaA1 | 3-phenylpropionate/trans-cinnamate dioxygenase subunit alpha [EC:1.14.12.19] |
|  | scaffold00001_gene_1750 | K05709 hcaF, hcaA2 | 3-phenylpropionate/trans-cinnamate dioxygenase subunit beta [EC:1.14.12.19] |
|  | scaffold00003_gene_3353 | K05710 hcaC | 3-phenylpropionate/trans-cinnamate dioxygenase ferredoxin component |
|  | scaffold00001_gene_1748 | K00529 hcaD | 3-phenylpropionate/trans-cinnamate dioxygenase ferredoxin reductase component [EC:1.18.1.3] |
|  | scaffold00001_gene_1205 | K05712 mhpA | 3-(3-hydroxy-phenyl)propionate hydroxylase [EC:1.14.13.127] |
|  | scaffold00002_gene_2366 | K05712 mhpA | 3-(3-hydroxy-phenyl)propionate hydroxylase [EC:1.14.13.127] |
|  | scaffold00002_gene_2911 | K05712 mhpA | 3-(3-hydroxy-phenyl)propionate hydroxylase [EC:1.14.13.127] |
|  | scaffold00006_gene_4850 | K05713 mhpB | 2,3-dihydroxyphenylpropionate 1,2-dioxygenase [EC:1.13.11.16] |
|  | scaffold00001_gene_298 | K01666 mhpE | 4-hydroxy 2-oxovalerate aldolase [EC:4.1.3.39] |
|  | scaffold00001_gene_297 | K04073 mhpF | acetaldehyde dehydrogenase [EC:1.2.1.10] |
| 00380 Tryptophan metabolism [PATH:ko00380] | | | |
|  | scaffold00008_gene_5125 | K00453 E1.13.11.11, TDO2 | tryptophan 2,3-dioxygenase [EC:1.13.11.11] |
|  | scaffold00004_gene_4078 | K01432 AFMID | arylformamidase [EC:3.5.1.9] |
|  | scaffold00008_gene_5126 | K07130 kynB | arylformamidase [EC:3.5.1.9] |
|  | scaffold00002_gene_2122 | K01556 KYNU, kynU | kynureninase [EC:3.7.1.3] |
|  | scaffold00002_gene_2305 | K00452 HAAO | 3-hydroxyanthranilate 3,4-dioxygenase [EC:1.13.11.6] |
|  | scaffold00002_gene_2306 | K03392 ACMSD | aminocarboxymuconate-semialdehyde decarboxylase [EC:4.1.1.45] |
|  | scaffold00003_gene_3820 | K00164 OGDH, sucA | 2-oxoglutarate dehydrogenase E1 component [EC:1.2.4.2] |
|  | scaffold00002_gene_1909 | K00252 GCDH, gcdH | glutaryl-CoA dehydrogenase [EC:1.3.8.6] |
|  | scaffold00001_gene_94 | K01692 paaF, echA | enoyl-CoA hydratase [EC:4.2.1.17] |
|  | scaffold00001_gene_201 | K01692 paaF, echA | enoyl-CoA hydratase [EC:4.2.1.17] |
|  | scaffold00001_gene_210 | K01692 paaF, echA | enoyl-CoA hydratase [EC:4.2.1.17] |
|  | scaffold00002_gene_2782 | K01692 paaF, echA | enoyl-CoA hydratase [EC:4.2.1.17] |
|  | scaffold00002_gene_2790 | K01692 paaF, echA | enoyl-CoA hydratase [EC:4.2.1.17] |
|  | scaffold00002_gene_2819 | K01692 paaF, echA | enoyl-CoA hydratase [EC:4.2.1.17] |
|  | scaffold00004_gene_4239 | K01692 paaF, echA | enoyl-CoA hydratase [EC:4.2.1.17] |
|  | scaffold00001_gene_113 | K01782 fadJ | 3-hydroxyacyl-CoA dehydrogenase / enoyl-CoA hydratase / 3-hydroxybutyryl-CoA epimerase [EC:1.1.1.35 4.2.1.17 5.1.2.3] |
|  | scaffold00001_gene_199 | K00626 E2.3.1.9, atoB | acetyl-CoA C-acetyltransferase [EC:2.3.1.9] |
|  | scaffold00002_gene_2940 | K00626 E2.3.1.9, atoB | acetyl-CoA C-acetyltransferase [EC:2.3.1.9] |
|  | scaffold00002_gene_3076 | K00626 E2.3.1.9, atoB | acetyl-CoA C-acetyltransferase [EC:2.3.1.9] |
|  | scaffold00006_gene_4805 | K00626 E2.3.1.9, atoB | acetyl-CoA C-acetyltransferase [EC:2.3.1.9] |
|  | scaffold00001_gene_377 | K00128 E1.2.1.3 | aldehyde dehydrogenase (NAD+) [EC:1.2.1.3] |
|  | scaffold00002_gene_2864 | K00128 E1.2.1.3 | aldehyde dehydrogenase (NAD+) [EC:1.2.1.3] |
|  | scaffold00003_gene_3561 | K00128 E1.2.1.3 | aldehyde dehydrogenase (NAD+) [EC:1.2.1.3] |
|  | scaffold00006_gene_4875 | K00128 E1.2.1.3 | aldehyde dehydrogenase (NAD+) [EC:1.2.1.3] |
|  | scaffold00002_gene_2302 | K00149 ALDH9A1 | aldehyde dehydrogenase family 9 member A1 [EC:1.2.1.47 1.2.1.3] |
|  | scaffold00001_gene_712 | K01426 E3.5.1.4, amiE | amidase [EC:3.5.1.4] |
|  | scaffold00001_gene_1579 | K01426 E3.5.1.4, amiE | amidase [EC:3.5.1.4] |
|  | scaffold00002_gene_2403 | K01426 E3.5.1.4, amiE | amidase [EC:3.5.1.4] |
|  | scaffold00002_gene_2828 | K01426 E3.5.1.4, amiE | amidase [EC:3.5.1.4] |
|  | scaffold00005_gene_4718 | K01426 E3.5.1.4, amiE | amidase [EC:3.5.1.4] |
|  | scaffold00001_gene_1243 | K03781 katE, CAT, catB, srpA | catalase [EC:1.11.1.6] |
|  | scaffold00002_gene_3167 | K03781 katE, CAT, catB, srpA | catalase [EC:1.11.1.6] |
| 00400 Phenylalanine, tyrosine and tryptophan biosynthesis [PATH:ko00400] | | | |
|  | scaffold00001_gene_1039 | K01626 E2.5.1.54, aroF, aroG, aroH | 3-deoxy-7-phosphoheptulonate synthase [EC:2.5.1.54] |
|  | scaffold00005_gene_4637 | K01626 E2.5.1.54, aroF, aroG, aroH | 3-deoxy-7-phosphoheptulonate synthase [EC:2.5.1.54] |
|  | scaffold00004_gene_4472 | K01735 aroB | 3-dehydroquinate synthase [EC:4.2.3.4] |
|  | scaffold00004_gene_4032 | K03786 aroQ, qutE | 3-dehydroquinate dehydratase II [EC:4.2.1.10] |
|  | scaffold00002_gene_1817 | K00014 aroE | shikimate dehydrogenase [EC:1.1.1.25] |
|  | scaffold00004_gene_4038 | K00014 aroE | shikimate dehydrogenase [EC:1.1.1.25] |
|  | scaffold00004_gene_4473 | K00891 E2.7.1.71, aroK, aroL | shikimate kinase [EC:2.7.1.71] |
|  | scaffold00003_gene_3889 | K00800 aroA | 3-phosphoshikimate 1-carboxyvinyltransferase [EC:2.5.1.19] |
|  | scaffold00007_gene_5074 | K01736 aroC | chorismate synthase [EC:4.2.3.5] |
|  | scaffold00002_gene_2844 | K01657 trpE | anthranilate synthase component I [EC:4.1.3.27] |
|  | scaffold00002_gene_2845 | K01658 trpG | anthranilate synthase component II [EC:4.1.3.27] |
|  | scaffold00002_gene_2846 | K00766 trpD | anthranilate phosphoribosyltransferase [EC:2.4.2.18] |
|  | scaffold00001_gene_1791 | K01817 trpF | phosphoribosylanthranilate isomerase [EC:5.3.1.24] |
|  | scaffold00002_gene_2847 | K01609 trpC | indole-3-glycerol phosphate synthase [EC:4.1.1.48] |
|  | scaffold00002_gene_2218 | K01695 trpA | tryptophan synthase alpha chain [EC:4.2.1.20] |
|  | scaffold00003_gene_3708 | K01695 trpA | tryptophan synthase alpha chain [EC:4.2.1.20] |
|  | scaffold00002_gene_2219 | K01696 trpB | tryptophan synthase beta chain [EC:4.2.1.20] |
|  | scaffold00003_gene_3707 | K01696 trpB | tryptophan synthase beta chain [EC:4.2.1.20] |
|  | scaffold00001_gene_497 | K04092 tyrA1 | chorismate mutase [EC:5.4.99.5] |
|  | scaffold00003_gene_3886 | K14170 pheA | chorismate mutase / prephenate dehydratase [EC:5.4.99.5 4.2.1.51] |
|  | scaffold00003_gene_3888 | K04517 tyrA2 | prephenate dehydrogenase [EC:1.3.1.12] |
|  | scaffold00002_gene_3001 | K00817 hisC | histidinol-phosphate aminotransferase [EC:2.6.1.9] |
|  | scaffold00003_gene_3887 | K00817 hisC | histidinol-phosphate aminotransferase [EC:2.6.1.9] |
|  | scaffold00007_gene_5025 | K00817 hisC | histidinol-phosphate aminotransferase [EC:2.6.1.9] |
|  | scaffold00001_gene_533 | K00832 tyrB | aromatic-amino-acid transaminase [EC:2.6.1.57] |
|  |  |  |  |
| Metabolism of other amino acids | | | |
| 00410 beta-Alanine metabolism [PATH:ko00410] | | | |
|  | scaffold00007_gene_4998 | K01579 panD | aspartate 1-decarboxylase [EC:4.1.1.11] |
|  | scaffold00001_gene_142 | K00823 puuE | 4-aminobutyrate aminotransferase [EC:2.6.1.19] |
|  | scaffold00001_gene_222 | K00797 speE, SRM | spermidine synthase [EC:2.5.1.16] |
|  | scaffold00001_gene_1096 | K00797 speE, SRM | spermidine synthase [EC:2.5.1.16] |
|  | scaffold00002_gene_2894 | K00797 speE, SRM | spermidine synthase [EC:2.5.1.16] |
|  | scaffold00001_gene_377 | K00128 E1.2.1.3 | aldehyde dehydrogenase (NAD+) [EC:1.2.1.3] |
|  | scaffold00002_gene_2864 | K00128 E1.2.1.3 | aldehyde dehydrogenase (NAD+) [EC:1.2.1.3] |
|  | scaffold00003_gene_3561 | K00128 E1.2.1.3 | aldehyde dehydrogenase (NAD+) [EC:1.2.1.3] |
|  | scaffold00006_gene_4875 | K00128 E1.2.1.3 | aldehyde dehydrogenase (NAD+) [EC:1.2.1.3] |
|  | scaffold00002_gene_2302 | K00149 ALDH9A1 | aldehyde dehydrogenase family 9 member A1 [EC:1.2.1.47 1.2.1.3] |
|  | scaffold00004_gene_4100 | K01918 panC | pantoate--beta-alanine ligase [EC:6.3.2.1] |
|  | scaffold00002_gene_2396 | K00822 E2.6.1.18 | beta-alanine--pyruvate transaminase [EC:2.6.1.18] |
|  | scaffold00001_gene_94 | K01692 paaF, echA | enoyl-CoA hydratase [EC:4.2.1.17] |
|  | scaffold00001_gene_201 | K01692 paaF, echA | enoyl-CoA hydratase [EC:4.2.1.17] |
|  | scaffold00001_gene_210 | K01692 paaF, echA | enoyl-CoA hydratase [EC:4.2.1.17] |
|  | scaffold00002_gene_2782 | K01692 paaF, echA | enoyl-CoA hydratase [EC:4.2.1.17] |
|  | scaffold00002_gene_2790 | K01692 paaF, echA | enoyl-CoA hydratase [EC:4.2.1.17] |
|  | scaffold00002_gene_2819 | K01692 paaF, echA | enoyl-CoA hydratase [EC:4.2.1.17] |
|  | scaffold00004_gene_4239 | K01692 paaF, echA | enoyl-CoA hydratase [EC:4.2.1.17] |
|  | scaffold00001_gene_113 | K01782 fadJ | 3-hydroxyacyl-CoA dehydrogenase / enoyl-CoA hydratase / 3-hydroxybutyryl-CoA epimerase [EC:1.1.1.35 4.2.1.17 5.1.2.3] |
|  | scaffold00001_gene_947 | K00249 ACADM, acd | acyl-CoA dehydrogenase [EC:1.3.8.7] |
|  | scaffold00001_gene_1360 | K00249 ACADM, acd | acyl-CoA dehydrogenase [EC:1.3.8.7] |
|  | scaffold00002_gene_2552 | K00249 ACADM, acd | acyl-CoA dehydrogenase [EC:1.3.8.7] |
|  | scaffold00004_gene_4064 | K00249 ACADM, acd | acyl-CoA dehydrogenase [EC:1.3.8.7] |
|  | scaffold00004_gene_4183 | K00249 ACADM, acd | acyl-CoA dehydrogenase [EC:1.3.8.7] |
|  | scaffold00006_gene_4791 | K00249 ACADM, acd | acyl-CoA dehydrogenase [EC:1.3.8.7] |
|  | scaffold00001_gene_209 | K01578 MLYCD | malonyl-CoA decarboxylase [EC:4.1.1.9] |
|  | scaffold00002_gene_2397 | K00140 mmsA, iolA, ALDH6A1 | malonate-semialdehyde dehydrogenase (acetylating) / methylmalonate-semialdehyde dehydrogenase [EC:1.2.1.18 1.2.1.27] |
|  | scaffold00003_gene_3754 | K00140 mmsA, iolA, ALDH6A1 | malonate-semialdehyde dehydrogenase (acetylating) / methylmalonate-semialdehyde dehydrogenase [EC:1.2.1.18 1.2.1.27] |
| 00430 Taurine and hypotaurine metabolism [PATH:ko00430] | | | |
|  | scaffold00001_gene_1445 | K00681 ggt | gamma-glutamyltranspeptidase / glutathione hydrolase [EC:2.3.2.2 3.4.19.13] |
|  | scaffold00003_gene_3337 | K00681 ggt | gamma-glutamyltranspeptidase / glutathione hydrolase [EC:2.3.2.2 3.4.19.13] |
|  | scaffold00002_gene_3171 | K03119 tauD | taurine dioxygenase [EC:1.14.11.17] |
|  | scaffold00003_gene_3671 | K03119 tauD | taurine dioxygenase [EC:1.14.11.17] |
|  | scaffold00002_gene_1995 | K00625 E2.3.1.8, pta | phosphate acetyltransferase [EC:2.3.1.8] |
|  | scaffold00002_gene_1996 | K00925 ackA | acetate kinase [EC:2.7.2.1] |
| 00440 Phosphonate and phosphinate metabolism [PATH:ko00440] | | | |
|  | scaffold00006_gene_4769 | K01841 pepM | phosphoenolpyruvate phosphomutase [EC:5.4.2.9] |
|  | scaffold00005_gene_4562 | K05306 phnX | phosphonoacetaldehyde hydrolase [EC:3.11.1.1] |
|  | scaffold00002_gene_2703 | K03823 pat | phosphinothricin acetyltransferase [EC:2.3.1.183] |
|  | scaffold00003_gene_3476 | K03823 pat | phosphinothricin acetyltransferase [EC:2.3.1.183] |
|  | scaffold00002_gene_1962 | K06164 phnI | alpha-D-ribose 1-methylphosphonate 5-triphosphate synthase subunit PhnI [EC:2.7.8.37] |
|  | scaffold00002_gene_1964 | K06166 phnG | alpha-D-ribose 1-methylphosphonate 5-triphosphate synthase subunit PhnG [EC:2.7.8.37] |
|  | scaffold00002_gene_1963 | K06165 phnH | alpha-D-ribose 1-methylphosphonate 5-triphosphate synthase subunit PhnH [EC:2.7.8.37] |
|  | scaffold00002_gene_1959 | K05780 phnL | alpha-D-ribose 1-methylphosphonate 5-triphosphate synthase subunit PhnL [EC:2.7.8.37] |
|  | scaffold00002_gene_1958 | K06162 phnM | alpha-D-ribose 1-methylphosphonate 5-triphosphate diphosphatase [EC:3.6.1.63] |
|  | scaffold00002_gene_1961 | K06163 phnJ | alpha-D-ribose 1-methylphosphonate 5-phosphate C-P lyase [EC:4.7.1.1] |
| 00450 Selenocompound metabolism [PATH:ko00450] | | | |
|  | scaffold00004_gene_4513 | K01739 metB | cystathionine gamma-synthase [EC:2.5.1.48] |
|  | scaffold00002_gene_3067 | K01760 metC | cystathionine beta-lyase [EC:4.4.1.8] |
|  | scaffold00002_gene_3201 | K01760 metC | cystathionine beta-lyase [EC:4.4.1.8] |
|  | scaffold00002_gene_2712 | K00548 metH, MTR | 5-methyltetrahydrofolate--homocysteine methyltransferase [EC:2.1.1.13] |
|  | scaffold00001_gene_645 | K00549 metE | 5-methyltetrahydropteroyltriglutamate--homocysteine methyltransferase [EC:2.1.1.14] |
|  | scaffold00005_gene_4611 | K00549 metE | 5-methyltetrahydropteroyltriglutamate--homocysteine methyltransferase [EC:2.1.1.14] |
|  | scaffold00001_gene_309 | K01758 CTH | cystathionine gamma-lyase [EC:4.4.1.1] |
|  | scaffold00001_gene_721 | K01761 E4.4.1.11 | methionine-gamma-lyase [EC:4.4.1.11] |
|  | scaffold00001_gene_1598 | K11717 sufS | cysteine desulfurase / selenocysteine lyase [EC:2.8.1.7 4.4.1.16] |
|  | scaffold00002_gene_3031 | K11717 sufS | cysteine desulfurase / selenocysteine lyase [EC:2.8.1.7 4.4.1.16] |
|  | scaffold00001_gene_878 | K00384 trxB | thioredoxin reductase (NADPH) [EC:1.8.1.9] |
|  | scaffold00003_gene_3500 | K00384 trxB | thioredoxin reductase (NADPH) [EC:1.8.1.9] |
|  | scaffold00008_gene_5098 | K00956 cysN | sulfate adenylyltransferase subunit 1 [EC:2.7.7.4] |
|  | scaffold00008_gene_5097 | K00957 cysD | sulfate adenylyltransferase subunit 2 [EC:2.7.7.4] |
|  | scaffold00002_gene_2854 | K01008 selD, SEPHS | selenide, water dikinase [EC:2.7.9.3] |
|  | scaffold00002_gene_2422 | K01874 MARS, metG | methionyl-tRNA synthetase [EC:6.1.1.10] |
| 00460 Cyanoamino acid metabolism [PATH:ko00460] | | | |
|  | scaffold00001_gene_1445 | K00681 ggt | gamma-glutamyltranspeptidase / glutathione hydrolase [EC:2.3.2.2 3.4.19.13] |
|  | scaffold00003_gene_3337 | K00681 ggt | gamma-glutamyltranspeptidase / glutathione hydrolase [EC:2.3.2.2 3.4.19.13] |
|  | scaffold00003_gene_3581 | K13035 NIT4 | beta-cyano-L-alanine hydratase/nitrilase [EC:3.5.5.4 4.2.1.65] |
|  | scaffold00002_gene_3198 | K01424 E3.5.1.1, ansA, ansB | L-asparaginase [EC:3.5.1.1] |
|  | scaffold00002_gene_2501 | K00600 glyA, SHMT | glycine hydroxymethyltransferase [EC:2.1.2.1] |
|  | scaffold00004_gene_3984 | K00600 glyA, SHMT | glycine hydroxymethyltransferase [EC:2.1.2.1] |
|  | scaffold00002_gene_3137 | K10815 hcnB | hydrogen cyanide synthase HcnB [EC:1.4.99.5] |
|  | scaffold00002_gene_3139 | K10816 hcnC | hydrogen cyanide synthase HcnC [EC:1.4.99.5] |
|  | scaffold00001_gene_339 | K01455 E3.5.1.49 | formamidase [EC:3.5.1.49] |
| 00471 D-Glutamine and D-glutamate metabolism [PATH:ko00471] | | | |
|  | scaffold00002_gene_2075 | K01776 murI | glutamate racemase [EC:5.1.1.3] |
|  | scaffold00003_gene_3224 | K01925 murD | UDP-N-acetylmuramoylalanine--D-glutamate ligase [EC:6.3.2.9] |
|  | scaffold00001_gene_1305 | K00261 GLUD1_2, gdhA | glutamate dehydrogenase (NAD(P)+) [EC:1.4.1.3] |
|  | scaffold00003_gene_3227 | K01924 murC | UDP-N-acetylmuramate--alanine ligase [EC:6.3.2.8] |
| 00472 D-Arginine and D-ornithine metabolism [PATH:ko00472] | | | |
|  | scaffold00004_gene_4408 | K00824 dat | D-alanine transaminase [EC:2.6.1.21] |
|  | scaffold00003_gene_3579 | K19746 dauA | D-arginine dehydrogenase [EC:1.4.99.6] |
| 00473 D-Alanine metabolism [PATH:ko00473] | | | |
|  | scaffold00001_gene_405 | K01775 alr | alanine racemase [EC:5.1.1.1] |
|  | scaffold00003_gene_3228 | K01921 ddl | D-alanine-D-alanine ligase [EC:6.3.2.4] |
|  | scaffold00004_gene_4408 | K00824 dat | D-alanine transaminase [EC:2.6.1.21] |
|  | scaffold00004_gene_4021 | K03367 dltA | D-alanine--poly(phosphoribitol) ligase subunit 1 [EC:6.1.1.13] |
| 00480 Glutathione metabolism [PATH:ko00480] | | | |
|  | scaffold00001_gene_1445 | K00681 ggt | gamma-glutamyltranspeptidase / glutathione hydrolase [EC:2.3.2.2 3.4.19.13] |
|  | scaffold00003_gene_3337 | K00681 ggt | gamma-glutamyltranspeptidase / glutathione hydrolase [EC:2.3.2.2 3.4.19.13] |
|  | scaffold00001_gene_920 | K01469 OPLAH, OXP1, oplAH | 5-oxoprolinase (ATP-hydrolysing) [EC:3.5.2.9] |
|  | scaffold00002_gene_2755 | K01919 gshA | glutamate--cysteine ligase [EC:6.3.2.2] |
|  | scaffold00001_gene_1769 | K01920 gshB, GSS | glutathione synthase [EC:6.3.2.3] |
|  | scaffold00003_gene_3750 | K01255 CARP, pepA | leucyl aminopeptidase [EC:3.4.11.1] |
|  | scaffold00005_gene_4633 | K01256 pepN | aminopeptidase N [EC:3.4.11.2] |
|  | scaffold00001_gene_735 | K00799 GST, gst | glutathione S-transferase [EC:2.5.1.18] |
|  | scaffold00001_gene_1011 | K00799 GST, gst | glutathione S-transferase [EC:2.5.1.18] |
|  | scaffold00001_gene_1673 | K00799 GST, gst | glutathione S-transferase [EC:2.5.1.18] |
|  | scaffold00002_gene_1815 | K00799 GST, gst | glutathione S-transferase [EC:2.5.1.18] |
|  | scaffold00002_gene_1993 | K00799 GST, gst | glutathione S-transferase [EC:2.5.1.18] |
|  | scaffold00002_gene_2533 | K00799 GST, gst | glutathione S-transferase [EC:2.5.1.18] |
|  | scaffold00004_gene_4470 | K00799 GST, gst | glutathione S-transferase [EC:2.5.1.18] |
|  | scaffold00006_gene_4869 | K00799 GST, gst | glutathione S-transferase [EC:2.5.1.18] |
|  | scaffold00001_gene_1660 | K00383 GSR, gor | glutathione reductase (NADPH) [EC:1.8.1.7] |
|  | scaffold00001_gene_1185 | K00031 IDH1, IDH2, icd | isocitrate dehydrogenase [EC:1.1.1.42] |
|  | scaffold00003_gene_3482 | K00031 IDH1, IDH2, icd | isocitrate dehydrogenase [EC:1.1.1.42] |
|  | scaffold00001_gene_1296 | K00432 E1.11.1.9 | glutathione peroxidase [EC:1.11.1.9] |
|  | scaffold00001_gene_222 | K00797 speE, SRM | spermidine synthase [EC:2.5.1.16] |
|  | scaffold00001_gene_1096 | K00797 speE, SRM | spermidine synthase [EC:2.5.1.16] |
|  | scaffold00002_gene_2894 | K00797 speE, SRM | spermidine synthase [EC:2.5.1.16] |

**Table S17** **Genes assigned to the “biosynthesis of amino acids” pathway.**

| Predicted gene | Entry/Gene name | Enzyme/Protein |
| --- | --- | --- |
| scaffold00003_gene_3552 | K01803 TPI, tpiA | triosephosphate isomerase (TIM) [EC:5.3.1.1] |
| scaffold00002_gene_1999 | K00134 GAPDH, gapA | glyceraldehyde 3-phosphate dehydrogenase [EC:1.2.1.12] |
| scaffold00002_gene_1998 | K00927 PGK, pgk | phosphoglycerate kinase [EC:2.7.2.3] |
| scaffold00004_gene_4211 | K01834 PGAM, gpmA | 2,3-bisphosphoglycerate-dependent phosphoglycerate mutase [EC:5.4.2.11] |
| scaffold00002_gene_1881 | K15634 gpmB | probable phosphoglycerate mutase [EC:5.4.2.12] |
| scaffold00003_gene_3784 | K01689 ENO, eno | enolase [EC:4.2.1.11] |
| scaffold00003_gene_3295 | K00873 PK, pyk | pyruvate kinase [EC:2.7.1.40] |
| scaffold00002_gene_1952 | K01624 FBA, fbaA | fructose-bisphosphate aldolase, class II [EC:4.1.2.13] |
| scaffold00001_gene_1540 | K00615 E2.2.1.1, tktA, tktB | transketolase [EC:2.2.1.1] |
| scaffold00001_gene_1541 | K00615 E2.2.1.1, tktA, tktB | transketolase [EC:2.2.1.1] |
| scaffold00002_gene_1818 | K00615 E2.2.1.1, tktA, tktB | transketolase [EC:2.2.1.1] |
| scaffold00002_gene_1819 | K00615 E2.2.1.1, tktA, tktB | transketolase [EC:2.2.1.1] |
| scaffold00002_gene_2000 | K00615 E2.2.1.1, tktA, tktB | transketolase [EC:2.2.1.1] |
| scaffold00002_gene_1886 | K00616 E2.2.1.2, talA, talB | transaldolase [EC:2.2.1.2] |
| scaffold00002_gene_2842 | K01783 rpe, RPE | ribulose-phosphate 3-epimerase [EC:5.1.3.1] |
| scaffold00005_gene_4720 | K01807 rpiA | ribose 5-phosphate isomerase A [EC:5.3.1.6] |
| scaffold00001_gene_385 | K13831 hps-phi | 3-hexulose-6-phosphate synthase / 6-phospho-3-hexuloisomerase [EC:4.1.2.43 5.3.1.27] |
| scaffold00001_gene_1164 | K13831 hps-phi | 3-hexulose-6-phosphate synthase / 6-phospho-3-hexuloisomerase [EC:4.1.2.43 5.3.1.27] |
| scaffold00002_gene_2360 | K00948 PRPS, prsA | ribose-phosphate pyrophosphokinase [EC:2.7.6.1] |
| scaffold00001_gene_1798 | K01703 leuC | 3-isopropylmalate/(R)-2-methylmalate dehydratase large subunit [EC:4.2.1.33 4.2.1.35] |
| scaffold00002_gene_3099 | K01703 leuC | 3-isopropylmalate/(R)-2-methylmalate dehydratase large subunit [EC:4.2.1.33 4.2.1.35] |
| scaffold00001_gene_1797 | K01704 leuD | 3-isopropylmalate/(R)-2-methylmalate dehydratase small subunit [EC:4.2.1.33 4.2.1.35] |
| scaffold00002_gene_3098 | K01704 leuD | 3-isopropylmalate/(R)-2-methylmalate dehydratase small subunit [EC:4.2.1.33 4.2.1.35] |
| scaffold00001_gene_1796 | K00052 leuB | 3-isopropylmalate dehydrogenase [EC:1.1.1.85] |
| scaffold00002_gene_1908 | K01652 E2.2.1.6L, ilvB, ilvG, ilvI | acetolactate synthase I/II/III large subunit [EC:2.2.1.6] |
| scaffold00002_gene_1924 | K01652 E2.2.1.6L, ilvB, ilvG, ilvI | acetolactate synthase I/II/III large subunit [EC:2.2.1.6] |
| scaffold00002_gene_2609 | K01652 E2.2.1.6L, ilvB, ilvG, ilvI | acetolactate synthase I/II/III large subunit [EC:2.2.1.6] |
| scaffold00002_gene_3119 | K01652 E2.2.1.6L, ilvB, ilvG, ilvI | acetolactate synthase I/II/III large subunit [EC:2.2.1.6] |
| scaffold00003_gene_3239 | K01652 E2.2.1.6L, ilvB, ilvG, ilvI | acetolactate synthase I/II/III large subunit [EC:2.2.1.6] |
| scaffold00003_gene_3533 | K01652 E2.2.1.6L, ilvB, ilvG, ilvI | acetolactate synthase I/II/III large subunit [EC:2.2.1.6] |
| scaffold00003_gene_3534 | K01653 E2.2.1.6S, ilvH, ilvN | acetolactate synthase I/III small subunit [EC:2.2.1.6] |
| scaffold00003_gene_3535 | K00053 ilvC | ketol-acid reductoisomerase [EC:1.1.1.86] |
| scaffold00001_gene_424 | K01687 ilvD | dihydroxy-acid dehydratase [EC:4.2.1.9] |
| scaffold00001_gene_1779 | K01687 ilvD | dihydroxy-acid dehydratase [EC:4.2.1.9] |
| scaffold00002_gene_2699 | K01687 ilvD | dihydroxy-acid dehydratase [EC:4.2.1.9] |
| scaffold00004_gene_3906 | K01687 ilvD | dihydroxy-acid dehydratase [EC:4.2.1.9] |
| scaffold00003_gene_3430 | K00826 E2.6.1.42, ilvE | branched-chain amino acid aminotransferase [EC:2.6.1.42] |
| scaffold00003_gene_3687 | K00826 E2.6.1.42, ilvE | branched-chain amino acid aminotransferase [EC:2.6.1.42] |
| scaffold00002_gene_3052 | K01649 leuA | 2-isopropylmalate synthase [EC:2.3.3.13] |
| scaffold00001_gene_1184 | K01754 E4.3.1.19, ilvA, tdcB | threonine dehydratase [EC:4.3.1.19] |
| scaffold00003_gene_3404 | K01754 E4.3.1.19, ilvA, tdcB | threonine dehydratase [EC:4.3.1.19] |
| scaffold00003_gene_3541 | K01754 E4.3.1.19, ilvA, tdcB | threonine dehydratase [EC:4.3.1.19] |
| scaffold00004_gene_4267 | K01754 E4.3.1.19, ilvA, tdcB | threonine dehydratase [EC:4.3.1.19] |
| scaffold00005_gene_4554 | K01752 E4.3.1.17, sdaA, sdaB, tdcG | L-serine dehydratase [EC:4.3.1.17] |
| scaffold00002_gene_2501 | K00600 glyA, SHMT | glycine hydroxymethyltransferase [EC:2.1.2.1] |
| scaffold00004_gene_3984 | K00600 glyA, SHMT | glycine hydroxymethyltransferase [EC:2.1.2.1] |
| scaffold00003_gene_3764 | K01620 ltaE | threonine aldolase [EC:4.1.2.48] |
| scaffold00001_gene_386 | K00058 serA, PHGDH | D-3-phosphoglycerate dehydrogenase [EC:1.1.1.95] |
| scaffold00001_gene_1165 | K00058 serA, PHGDH | D-3-phosphoglycerate dehydrogenase [EC:1.1.1.95] |
| scaffold00002_gene_2733 | K00058 serA, PHGDH | D-3-phosphoglycerate dehydrogenase [EC:1.1.1.95] |
| scaffold00002_gene_2749 | K00058 serA, PHGDH | D-3-phosphoglycerate dehydrogenase [EC:1.1.1.95] |
| scaffold00003_gene_3885 | K00831 serC, PSAT1 | phosphoserine aminotransferase [EC:2.6.1.52] |
| scaffold00001_gene_904 | K01079 serB, PSPH | phosphoserine phosphatase [EC:3.1.3.3] |
| scaffold00003_gene_3654 | K01079 serB, PSPH | phosphoserine phosphatase [EC:3.1.3.3] |
| scaffold00001_gene_138 | K00640 cysE | serine O-acetyltransferase [EC:2.3.1.30] |
| scaffold00003_gene_3898 | K12339 cysM | cysteine synthase B [EC:2.5.1.47] |
| scaffold00001_gene_309 | K01758 CTH | cystathionine gamma-lyase [EC:4.4.1.1] |
| scaffold00001_gene_80 | K00928 lysC | aspartate kinase [EC:2.7.2.4] |
| scaffold00001_gene_1795 | K00133 asd | aspartate-semialdehyde dehydrogenase [EC:1.2.1.11] |
| scaffold00005_gene_4629 | K00003 E1.1.1.3 | homoserine dehydrogenase [EC:1.1.1.3] |
| scaffold00007_gene_5064 | K02204 thrB2 | homoserine kinase type II [EC:2.7.1.39] |
| scaffold00003_gene_3742 | K01733 thrC | threonine synthase [EC:4.2.3.1] |
| scaffold00005_gene_4630 | K01733 thrC | threonine synthase [EC:4.2.3.1] |
| scaffold00001_gene_952 | K01915 glnA, GLUL | glutamine synthetase [EC:6.3.1.2] |
| scaffold00002_gene_3209 | K01915 glnA, GLUL | glutamine synthetase [EC:6.3.1.2] |
| scaffold00002_gene_2983 | K00265 gltB | glutamate synthase (NADPH/NADH) large chain [EC:1.4.1.13 1.4.1.14] |
| scaffold00002_gene_2984 | K00266 gltD | glutamate synthase (NADPH/NADH) small chain [EC:1.4.1.13 1.4.1.14] |
| scaffold00007_gene_4993 | K00836 E2.6.1.76, ectB | diaminobutyrate-2-oxoglutarate transaminase [EC:2.6.1.76] |
| scaffold00004_gene_4513 | K01739 metB | cystathionine gamma-synthase [EC:2.5.1.48] |
| scaffold00002_gene_3067 | K01760 metC | cystathionine beta-lyase [EC:4.4.1.8] |
| scaffold00002_gene_3201 | K01760 metC | cystathionine beta-lyase [EC:4.4.1.8] |
| scaffold00002_gene_2712 | K00548 metH, MTR | 5-methyltetrahydrofolate--homocysteine methyltransferase [EC:2.1.1.13] |
| scaffold00001_gene_645 | K00549 metE | 5-methyltetrahydropteroyltriglutamate--homocysteine methyltransferase [EC:2.1.1.14] |
| scaffold00005_gene_4611 | K00549 metE | 5-methyltetrahydropteroyltriglutamate--homocysteine methyltransferase [EC:2.1.1.14] |
| scaffold00004_gene_4378 | K00789 metK | S-adenosylmethionine synthetase [EC:2.5.1.6] |
| scaffold00002_gene_2474 | K01243 mtnN, mtn, pfs | adenosylhomocysteine nucleosidase [EC:3.2.2.9] |
| scaffold00003_gene_3809 | K01647 CS, gltA | citrate synthase [EC:2.3.3.1] |
| scaffold00001_gene_176 | K01681 ACO, acnA | aconitate hydratase [EC:4.2.1.3] |
| scaffold00002_gene_2484 | K01681 ACO, acnA | aconitate hydratase [EC:4.2.1.3] |
| scaffold00001_gene_189 | K01682 acnB | aconitate hydratase 2 / 2-methylisocitrate dehydratase [EC:4.2.1.3 4.2.1.99] |
| scaffold00001_gene_1185 | K00031 IDH1, IDH2, icd | isocitrate dehydrogenase [EC:1.1.1.42] |
| scaffold00003_gene_3482 | K00031 IDH1, IDH2, icd | isocitrate dehydrogenase [EC:1.1.1.42] |
| scaffold00001_gene_23 | K01655 LYS21, LYS20 | homocitrate synthase [EC:2.3.3.14] |
| scaffold00004_gene_4452 | K05825 LYSN | 2-aminoadipate transaminase [EC:2.6.1.-] |
| scaffold00003_gene_3792 | K00290 LYS1 | saccharopine dehydrogenase (NAD+, L-lysine forming) [EC:1.5.1.7] |
| scaffold00004_gene_4081 | K00620 argJ | glutamate N-acetyltransferase / amino-acid N-acetyltransferase [EC:2.3.1.35 2.3.1.1] |
| scaffold00005_gene_4724 | K14682 argAB | amino-acid N-acetyltransferase [EC:2.3.1.1] |
| scaffold00004_gene_4298 | K00930 argB | acetylglutamate kinase [EC:2.7.2.8] |
| scaffold00001_gene_1213 | K00145 argC | N-acetyl-gamma-glutamyl-phosphate reductase [EC:1.2.1.38] |
| scaffold00004_gene_3991 | K00145 argC | N-acetyl-gamma-glutamyl-phosphate reductase [EC:1.2.1.38] |
| scaffold00005_gene_4699 | K00818 E2.6.1.11, argD | acetylornithine aminotransferase [EC:2.6.1.11] |
| scaffold00002_gene_3106 | K00821 argD | acetylornithine/N-succinyldiaminopimelate aminotransferase [EC:2.6.1.11 2.6.1.17] |
| scaffold00003_gene_3706 | K01438 argE | acetylornithine deacetylase [EC:3.5.1.16] |
| scaffold00005_gene_4698 | K00611 OTC, argF, argI | ornithine carbamoyltransferase [EC:2.1.3.3] |
| scaffold00005_gene_4692 | K01940 argG, ASS1 | argininosuccinate synthase [EC:6.3.4.5] |
| scaffold00001_gene_1465 | K01755 argH, ASL | argininosuccinate lyase [EC:4.3.2.1] |
| scaffold00001_gene_1020 | K01476 E3.5.3.1, rocF, arg | arginase [EC:3.5.3.1] |
| scaffold00004_gene_4200 | K00931 proB | glutamate 5-kinase [EC:2.7.2.11] |
| scaffold00006_gene_4760 | K00147 proA | glutamate-5-semialdehyde dehydrogenase [EC:1.2.1.41] |
| scaffold00001_gene_996 | K00286 proC | pyrroline-5-carboxylate reductase [EC:1.5.1.2] |
| scaffold00002_gene_2636 | K00286 proC | pyrroline-5-carboxylate reductase [EC:1.5.1.2] |
| scaffold00002_gene_2509 | K01750 E4.3.1.12, ocd | ornithine cyclodeaminase [EC:4.3.1.12] |
| scaffold00002_gene_2890 | K01750 E4.3.1.12, ocd | ornithine cyclodeaminase [EC:4.3.1.12] |
| scaffold00001_gene_38 | K01714 dapA | 4-hydroxy-tetrahydrodipicolinate synthase [EC:4.3.3.7] |
| scaffold00002_gene_2046 | K01714 dapA | 4-hydroxy-tetrahydrodipicolinate synthase [EC:4.3.3.7] |
| scaffold00002_gene_2513 | K01714 dapA | 4-hydroxy-tetrahydrodipicolinate synthase [EC:4.3.3.7] |
| scaffold00003_gene_3484 | K01714 dapA | 4-hydroxy-tetrahydrodipicolinate synthase [EC:4.3.3.7] |
| scaffold00004_gene_4449 | K01714 dapA | 4-hydroxy-tetrahydrodipicolinate synthase [EC:4.3.3.7] |
| scaffold00003_gene_3459 | K00215 dapB | 4-hydroxy-tetrahydrodipicolinate reductase [EC:1.17.1.8] |
| scaffold00001_gene_501 | K00674 dapD | 2,3,4,5-tetrahydropyridine-2-carboxylate N-succinyltransferase [EC:2.3.1.117] |
| scaffold00002_gene_2803 | K00674 dapD | 2,3,4,5-tetrahydropyridine-2-carboxylate N-succinyltransferase [EC:2.3.1.117] |
| scaffold00001_gene_502 | K14267 dapC | N-succinyldiaminopimelate aminotransferase [EC:2.6.1.17] |
| scaffold00001_gene_500 | K01439 dapE | succinyl-diaminopimelate desuccinylase [EC:3.5.1.18] |
| scaffold00003_gene_3815 | K01439 dapE | succinyl-diaminopimelate desuccinylase [EC:3.5.1.18] |
| scaffold00004_gene_4381 | K01778 dapF | diaminopimelate epimerase [EC:5.1.1.7] |
| scaffold00001_gene_427 | K01586 lysA | diaminopimelate decarboxylase [EC:4.1.1.20] |
| scaffold00004_gene_4476 | K01586 lysA | diaminopimelate decarboxylase [EC:4.1.1.20] |
| scaffold00002_gene_2999 | K00765 hisG | ATP phosphoribosyltransferase [EC:2.4.2.17] |
| scaffold00007_gene_5030 | K02502 hisZ | ATP phosphoribosyltransferase regulatory subunit |
| scaffold00002_gene_3007 | K01523 hisE | phosphoribosyl-ATP pyrophosphohydrolase [EC:3.6.1.31] |
| scaffold00002_gene_3006 | K01496 hisI | phosphoribosyl-AMP cyclohydrolase [EC:3.5.4.19] |
| scaffold00002_gene_3004 | K01814 hisA | phosphoribosylformimino-5-aminoimidazole carboxamide ribotide isomerase [EC:5.3.1.16] |
| scaffold00002_gene_3005 | K02500 hisF | cyclase [EC:4.1.3.-] |
| scaffold00002_gene_3003 | K02501 hisH | glutamine amidotransferase [EC:2.4.2.-] |
| scaffold00002_gene_3002 | K01693 hisB | imidazoleglycerol-phosphate dehydratase [EC:4.2.1.19] |
| scaffold00002_gene_3001 | K00817 hisC | histidinol-phosphate aminotransferase [EC:2.6.1.9] |
| scaffold00003_gene_3887 | K00817 hisC | histidinol-phosphate aminotransferase [EC:2.6.1.9] |
| scaffold00007_gene_5025 | K00817 hisC | histidinol-phosphate aminotransferase [EC:2.6.1.9] |
| scaffold00002_gene_3208 | K18649 IMPL2 | inositol-phosphate phosphatase / L-galactose 1-phosphate phosphatase / histidinol-phosphatase [EC:3.1.3.25 3.1.3.93 3.1.3.15] |
| scaffold00002_gene_3000 | K00013 hisD | histidinol dehydrogenase [EC:1.1.1.23] |
| scaffold00001_gene_1039 | K01626 E2.5.1.54, aroF, aroG, aroH | 3-deoxy-7-phosphoheptulonate synthase [EC:2.5.1.54] |
| scaffold00005_gene_4637 | K01626 E2.5.1.54, aroF, aroG, aroH | 3-deoxy-7-phosphoheptulonate synthase [EC:2.5.1.54] |
| scaffold00004_gene_4472 | K01735 aroB | 3-dehydroquinate synthase [EC:4.2.3.4] |
| scaffold00004_gene_4032 | K03786 aroQ, qutE | 3-dehydroquinate dehydratase II [EC:4.2.1.10] |
| scaffold00002_gene_1817 | K00014 aroE | shikimate dehydrogenase [EC:1.1.1.25] |
| scaffold00004_gene_4038 | K00014 aroE | shikimate dehydrogenase [EC:1.1.1.25] |
| scaffold00004_gene_4473 | K00891 E2.7.1.71, aroK, aroL | shikimate kinase [EC:2.7.1.71] |
| scaffold00003_gene_3889 | K00800 aroA | 3-phosphoshikimate 1-carboxyvinyltransferase [EC:2.5.1.19] |
| scaffold00007_gene_5074 | K01736 aroC | chorismate synthase [EC:4.2.3.5] |
| scaffold00002_gene_2844 | K01657 trpE | anthranilate synthase component I [EC:4.1.3.27] |
| scaffold00002_gene_2845 | K01658 trpG | anthranilate synthase component II [EC:4.1.3.27] |
| scaffold00002_gene_2846 | K00766 trpD | anthranilate phosphoribosyltransferase [EC:2.4.2.18] |
| scaffold00001_gene_1791 | K01817 trpF | phosphoribosylanthranilate isomerase [EC:5.3.1.24] |
| scaffold00002_gene_2847 | K01609 trpC | indole-3-glycerol phosphate synthase [EC:4.1.1.48] |
| scaffold00002_gene_2218 | K01695 trpA | tryptophan synthase alpha chain [EC:4.2.1.20] |
| scaffold00003_gene_3708 | K01695 trpA | tryptophan synthase alpha chain [EC:4.2.1.20] |
| scaffold00002_gene_2219 | K01696 trpB | tryptophan synthase beta chain [EC:4.2.1.20] |
| scaffold00003_gene_3707 | K01696 trpB | tryptophan synthase beta chain [EC:4.2.1.20] |
| scaffold00001_gene_497 | K04092 tyrA1 | chorismate mutase [EC:5.4.99.5] |
| scaffold00003_gene_3886 | K14170 pheA | chorismate mutase / prephenate dehydratase [EC:5.4.99.5 4.2.1.51] |
| scaffold00003_gene_3888 | K04517 tyrA2 | prephenate dehydrogenase [EC:1.3.1.12] |
| scaffold00001_gene_533 | K00832 tyrB | aromatic-amino-acid transaminase [EC:2.6.1.57] |

**Table S18** **Identified genes coding for amino acid transporters.**

| Predicted gene | Entry/Gene name | Enzyme/Protein |
| --- | --- | --- |
| scaffold00001_gene_922 | K10036 glnH | glutamine transport system substrate-binding protein |
| scaffold00001_gene_923 | K10037 glnP | glutamine transport system permease protein |
| scaffold00001_gene_924 | K10038 glnQ | glutamine transport system ATP-binding protein [EC:3.6.3.-] |
| scaffold00001_gene_1597 | K10001 gltI, aatJ | glutamate/aspartate transport system substrate-binding protein |
| scaffold00002_gene_3156 | K10002 gltK, aatM | glutamate/aspartate transport system permease protein |
| scaffold00002_gene_3157 | K10003 gltJ, aatQ | glutamate/aspartate transport system permease protein |
| scaffold00002_gene_3155 | K10004 gltL, aatP | glutamate/aspartate transport system ATP-binding protein [EC:3.6.3.-] |
| scaffold00002_gene_1930 | K09969 aapJ, bztA | general L-amino acid transport system substrate-binding protein |
| scaffold00002_gene_2619 | K09969 aapJ, bztA | general L-amino acid transport system substrate-binding protein |
| scaffold00002_gene_2679 | K09969 aapJ, bztA | general L-amino acid transport system substrate-binding protein |
| scaffold00004_gene_4259 | K09969 aapJ, bztA | general L-amino acid transport system substrate-binding protein |
| scaffold00002_gene_2678 | K09970 aapQ, bztB | general L-amino acid transport system permease protein |
| scaffold00002_gene_2677 | K09971 aapM, bztC | general L-amino acid transport system permease protein |
| scaffold00002_gene_2676 | K09972 aapP, bztD | general L-amino acid transport system ATP-binding protein [EC:3.6.3.-] |
| scaffold00002_gene_2126 | K02424 fliY | cystine transport system substrate-binding protein |
| scaffold00002_gene_2127 | K10009 ABC.CYST.P | cystine transport system permease protein |
| scaffold00002_gene_2128 | K10010 ABC.CYST.A | cystine transport system ATP-binding protein [EC:3.6.3.-] |
| scaffold00001_gene_987 | K01999 livK | branched-chain amino acid transport system substrate-binding protein |
| scaffold00001_gene_1002 | K01999 livK | branched-chain amino acid transport system substrate-binding protein |
| scaffold00002_gene_1824 | K01999 livK | branched-chain amino acid transport system substrate-binding protein |
| scaffold00002_gene_2825 | K01999 livK | branched-chain amino acid transport system substrate-binding protein |
| scaffold00003_gene_3680 | K01999 livK | branched-chain amino acid transport system substrate-binding protein |
| scaffold00004_gene_3998 | K01999 livK | branched-chain amino acid transport system substrate-binding protein |
| scaffold00004_gene_4077 | K01999 livK | branched-chain amino acid transport system substrate-binding protein |
| scaffold00004_gene_4322 | K01999 livK | branched-chain amino acid transport system substrate-binding protein |
| scaffold00004_gene_4331 | K01999 livK | branched-chain amino acid transport system substrate-binding protein |
| scaffold00001_gene_179 | K01997 livH | branched-chain amino acid transport system permease protein |
| scaffold00001_gene_1003 | K01997 livH | branched-chain amino acid transport system permease protein |
| scaffold00002_gene_1825 | K01997 livH | branched-chain amino acid transport system permease protein |
| scaffold00004_gene_3997 | K01997 livH | branched-chain amino acid transport system permease protein |
| scaffold00004_gene_4329 | K01997 livH | branched-chain amino acid transport system permease protein |
| scaffold00001_gene_180 | K01998 livM | branched-chain amino acid transport system permease protein |
| scaffold00001_gene_456 | K01998 livM | branched-chain amino acid transport system permease protein |
| scaffold00001_gene_548 | K01998 livM | branched-chain amino acid transport system permease protein |
| scaffold00001_gene_1004 | K01998 livM | branched-chain amino acid transport system permease protein |
| scaffold00001_gene_1644 | K01998 livM | branched-chain amino acid transport system permease protein |
| scaffold00004_gene_4330 | K01998 livM | branched-chain amino acid transport system permease protein |
| scaffold00001_gene_181 | K01995 livG | branched-chain amino acid transport system ATP-binding protein |
| scaffold00001_gene_457 | K01995 livG | branched-chain amino acid transport system ATP-binding protein |
| scaffold00001_gene_546 | K01995 livG | branched-chain amino acid transport system ATP-binding protein |
| scaffold00001_gene_1005 | K01995 livG | branched-chain amino acid transport system ATP-binding protein |
| scaffold00003_gene_3682 | K01995 livG | branched-chain amino acid transport system ATP-binding protein |
| scaffold00004_gene_3995 | K01995 livG | branched-chain amino acid transport system ATP-binding protein |
| scaffold00004_gene_4085 | K01995 livG | branched-chain amino acid transport system ATP-binding protein |
| scaffold00004_gene_4328 | K01995 livG | branched-chain amino acid transport system ATP-binding protein |
| scaffold00004_gene_4484 | K01995 livG | branched-chain amino acid transport system ATP-binding protein |
| scaffold00001_gene_458 | K01996 livF | branched-chain amino acid transport system ATP-binding protein |
| scaffold00001_gene_550 | K01996 livF | branched-chain amino acid transport system ATP-binding protein |
| scaffold00001_gene_1006 | K01996 livF | branched-chain amino acid transport system ATP-binding protein |
| scaffold00002_gene_1827 | K01996 livF | branched-chain amino acid transport system ATP-binding protein |
| scaffold00003_gene_3366 | K01996 livF | branched-chain amino acid transport system ATP-binding protein |
| scaffold00004_gene_3994 | K01996 livF | branched-chain amino acid transport system ATP-binding protein |
| scaffold00004_gene_4325 | K01996 livF | branched-chain amino acid transport system ATP-binding protein |
| scaffold00004_gene_4332 | K01996 livF | branched-chain amino acid transport system ATP-binding protein |
| scaffold00004_gene_4483 | K01996 livF | branched-chain amino acid transport system ATP-binding protein |
| scaffold00001_gene_306 | K02073 metQ | D-methionine transport system substrate-binding protein |
| scaffold00001_gene_1022 | K02073 metQ | D-methionine transport system substrate-binding protein |
| scaffold00003_gene_3253 | K02073 metQ | D-methionine transport system substrate-binding protein |
| scaffold00004_gene_4297 | K02073 metQ | D-methionine transport system substrate-binding protein |
| scaffold00001_gene_308 | K02072 metI | D-methionine transport system permease protein |
| scaffold00003_gene_3252 | K02072 metI | D-methionine transport system permease protein |
| scaffold00001_gene_307 | K02071 metN | D-methionine transport system ATP-binding protein |
| scaffold00003_gene_3251 | K02071 metN | D-methionine transport system ATP-binding protein |

**Table S19** **Genes assigned to the “nitrogen metabolism” pathway.**

| Predicted gene | Entry/Gene name | Enzyme/Protein |
| --- | --- | --- |
| scaffold00001_gene_339 | K01455 E3.5.1.49 | formamidase [EC:3.5.1.49] |
| scaffold00001_gene_1237 | K02575 NRT, narK, nrtP, nasA | MFS transporter, NNP family, nitrate/nitrite transporter |
| scaffold00001_gene_1238 | K02575 NRT, narK, nrtP, nasA | MFS transporter, NNP family, nitrate/nitrite transporter |
| scaffold00002_gene_2017 | K15576 nrtA, nasF, cynA | nitrate/nitrite transport system substrate-binding protein |
| scaffold00002_gene_2016 | K15577 nrtB, nasE, cynB | nitrate/nitrite transport system permease protein |
| scaffold00002_gene_2015 | K15578 nrtC, nasD | nitrate/nitrite transport system ATP-binding protein [EC:3.6.3.-] |
| scaffold00001_gene_1236 | K00370 narG | nitrate reductase alpha subunit [EC:1.7.99.4] |
| scaffold00001_gene_1235 | K00371 narH | nitrate reductase beta subunit [EC:1.7.99.4] |
| scaffold00001_gene_1233 | K00374 narI | nitrate reductase gamma subunit [EC:1.7.99.4] |
| scaffold00001_gene_1234 | K00373 narJ | nitrate reductase delta subunit |
| scaffold00002_gene_2013 | K00372 nasA | assimilatory nitrate reductase catalytic subunit [EC:1.7.99.4] |
| scaffold00001_gene_1614 | K02567 napA | periplasmic nitrate reductase NapA [EC:1.7.99.4] |
| scaffold00001_gene_1615 | K02568 napB | cytochrome c-type protein NapB |
| scaffold00001_gene_584 | K00362 nirB | nitrite reductase (NADH) large subunit [EC:1.7.1.15] |
| scaffold00002_gene_2014 | K00362 nirB | nitrite reductase (NADH) large subunit [EC:1.7.1.15] |
| scaffold00001_gene_583 | K00363 nirD | nitrite reductase (NADH) small subunit [EC:1.7.1.15] |
| scaffold00001_gene_1662 | K00368 nirK | nitrite reductase (NO-forming) [EC:1.7.2.1] |
| scaffold00001_gene_1665 | K04561 norB | nitric oxide reductase subunit B [EC:1.7.2.5] |
| scaffold00001_gene_68 | K00376 nosZ | nitrous-oxide reductase [EC:1.7.2.4] |
| scaffold00001_gene_1112 | K00459 ncd2, npd | nitronate monooxygenase [EC:1.13.12.16] |
| scaffold00001_gene_1254 | K00459 ncd2, npd | nitronate monooxygenase [EC:1.13.12.16] |
| scaffold00002_gene_2788 | K00459 ncd2, npd | nitronate monooxygenase [EC:1.13.12.16] |
| scaffold00002_gene_2989 | K00459 ncd2, npd | nitronate monooxygenase [EC:1.13.12.16] |
| scaffold00003_gene_3311 | K00459 ncd2, npd | nitronate monooxygenase [EC:1.13.12.16] |
| scaffold00001_gene_1305 | K00261 GLUD1_2, gdhA | glutamate dehydrogenase (NAD(P)+) [EC:1.4.1.3] |
| scaffold00004_gene_4317 | K00262 E1.4.1.4, gdhA | glutamate dehydrogenase (NADP+) [EC:1.4.1.4] |
| scaffold00001_gene_952 | K01915 glnA, GLUL | glutamine synthetase [EC:6.3.1.2] |
| scaffold00002_gene_3209 | K01915 glnA, GLUL | glutamine synthetase [EC:6.3.1.2] |
| scaffold00002_gene_2983 | K00265 gltB | glutamate synthase (NADPH/NADH) large chain [EC:1.4.1.13 1.4.1.14] |
| scaffold00002_gene_2984 | K00266 gltD | glutamate synthase (NADPH/NADH) small chain [EC:1.4.1.13 1.4.1.14] |
| scaffold00001_gene_1230 | K01673 cynT, can | carbonic anhydrase [EC:4.2.1.1] |
| scaffold00004_gene_4117 | K01673 cynT, can | carbonic anhydrase [EC:4.2.1.1] |

**Table S20** **Identified secondary metabolite clusters in strain HZ01.**

| Cluster type | Scaffold | From | To | Most similar known cluster | MIBiG BGC-ID |
| --- | --- | --- | --- | --- | --- |
| Terpene | scaffold00001 | 415030 | 436725 | —^a^ | — |
| Siderophore | scaffold00001 | 1265772 | 1277625 | Desferrioxamine_B_biosynthetic_gene_cluster (40% of genes show similarity) | BGC0000941_c1 |
| Arylpolyene | scaffold00001 | 1793826 | 1837704 | APE_Vf_biosynthetic_gene_cluster (35% of genes show similarity) | BGC0000837_c1 |
| Resorcinol | scaffold00004 | 536408 | 578330 | — | — |
| Phosphonate | scaffold00006 | 1 | 38834 | — | — |
| Ectoine | scaffold00007 | 52088 | 63122 | Ectoine_biosynthetic_gene_cluster (75% of genes show similarity) | BGC0000856_c1 |
| T1pks | scaffold00001 | 1014634 | 1062265 | Capsular_polysaccharide_biosynthetic_gene_cluster (26% of genes show similarity) | BGC0000735_c1 |

^a^No record.

**Table S21** **Genes related to the biosynthesis of other secondary metabolites.**

| Pathway | Predicted gene | Entry/Gene name | Enzyme/Protein |
| --- | --- | --- | --- |
| 01051 Biosynthesis of ansamycins [PATH:ko01051] | | | |
|  | scaffold00001_gene_1540 | K00615 E2.2.1.1, tktA, tktB | transketolase [EC:2.2.1.1] |
|  | scaffold00001_gene_1541 | K00615 E2.2.1.1, tktA, tktB | transketolase [EC:2.2.1.1] |
|  | scaffold00002_gene_1818 | K00615 E2.2.1.1, tktA, tktB | transketolase [EC:2.2.1.1] |
|  | scaffold00002_gene_1819 | K00615 E2.2.1.1, tktA, tktB | transketolase [EC:2.2.1.1] |
|  | scaffold00002_gene_2000 | K00615 E2.2.1.1, tktA, tktB | transketolase [EC:2.2.1.1] |
| 00253 Tetracycline biosynthesis [PATH:ko00253] | | | |
|  | scaffold00001_gene_78 | K01962 accA | acetyl-CoA carboxylase carboxyl transferase subunit alpha [EC:6.4.1.2] |
|  | scaffold00004_gene_4031 | K02160 accB, bccP | acetyl-CoA carboxylase biotin carboxyl carrier protein |
|  | scaffold00004_gene_4030 | K01961 accC | acetyl-CoA carboxylase, biotin carboxylase subunit [EC:6.4.1.2 6.3.4.14] |
|  | scaffold00003_gene_3709 | K01963 accD | acetyl-CoA carboxylase carboxyl transferase subunit beta [EC:6.4.1.2] |
| 01055 Biosynthesis of vancomycin group antibiotics [PATH:ko01055] | | | |
|  | scaffold00004_gene_4397 | K01710 E4.2.1.46, rfbB, rffG | dTDP-glucose 4,6-dehydratase [EC:4.2.1.46] |
| 00950 Isoquinoline alkaloid biosynthesis [PATH:ko00950] | | | |
|  | scaffold00001_gene_533 | K00832 tyrB | aromatic-amino-acid transaminase [EC:2.6.1.57] |
| 00960 Tropane, piperidine and pyridine alkaloid biosynthesis [PATH:ko00960] | | | |
|  | scaffold00002_gene_3001 | K00817 hisC | histidinol-phosphate aminotransferase [EC:2.6.1.9] |
|  | scaffold00003_gene_3887 | K00817 hisC | histidinol-phosphate aminotransferase [EC:2.6.1.9] |
|  | scaffold00007_gene_5025 | K00817 hisC | histidinol-phosphate aminotransferase [EC:2.6.1.9] |
|  | scaffold00001_gene_533 | K00832 tyrB | aromatic-amino-acid transaminase [EC:2.6.1.57] |
| 00965 Betalain biosynthesis [PATH:ko00965] | | | |
|  | scaffold00001_gene_175 | K15777 DOPA | 4,5-DOPA dioxygenase extradiol [EC:1.13.11.-] |
| 00311 Penicillin and cephalosporin biosynthesis [PATH:ko00311] | | | |
|  | scaffold00001_gene_921 | K19200 IAL | isopenicillin-N N-acyltransferase like protein |
|  | scaffold00001_gene_1174 | K01434 E3.5.1.11 | penicillin amidase [EC:3.5.1.11] |
| Genes related to the biosynthesis of Bacilysin | | | |
|  | scaffold00002_gene_2392 | K00560 thyA, TYMS | thymidylate synthase [EC:2.1.1.45] |
|  | scaffold00001_gene_663 | K15580 oppA, mppA | oligopeptide transport system substrate-binding protein |
|  | scaffold00002_gene_2796 | K15580 oppA, mppA | oligopeptide transport system substrate-binding protein |
|  | scaffold00004_gene_4177 | K07691 comA | two-component system, NarL family, competent response regulator ComA |
| 00332 Carbapenem biosynthesis [PATH:ko00332] | | | |
|  | scaffold00004_gene_4200 | K00931 proB | glutamate 5-kinase [EC:2.7.2.11] |
|  | scaffold00006_gene_4760 | K00147 proA | glutamate-5-semialdehyde dehydrogenase [EC:1.2.1.41] |
| 00261 Monobactam biosynthesis [PATH:ko00261] | | | |
|  | scaffold00008_gene_5098 | K00956 cysN | sulfate adenylyltransferase subunit 1 [EC:2.7.7.4] |
|  | scaffold00008_gene_5097 | K00957 cysD | sulfate adenylyltransferase subunit 2 [EC:2.7.7.4] |
|  | scaffold00001_gene_80 | K00928 lysC | aspartate kinase [EC:2.7.2.4] |
|  | scaffold00001_gene_1795 | K00133 asd | aspartate-semialdehyde dehydrogenase [EC:1.2.1.11] |
|  | scaffold00001_gene_38 | K01714 dapA | 4-hydroxy-tetrahydrodipicolinate synthase [EC:4.3.3.7] |
|  | scaffold00002_gene_2046 | K01714 dapA | 4-hydroxy-tetrahydrodipicolinate synthase [EC:4.3.3.7] |
|  | scaffold00002_gene_2513 | K01714 dapA | 4-hydroxy-tetrahydrodipicolinate synthase [EC:4.3.3.7] |
|  | scaffold00003_gene_3484 | K01714 dapA | 4-hydroxy-tetrahydrodipicolinate synthase [EC:4.3.3.7] |
|  | scaffold00004_gene_4449 | K01714 dapA | 4-hydroxy-tetrahydrodipicolinate synthase [EC:4.3.3.7] |
|  | scaffold00003_gene_3459 | K00215 dapB | 4-hydroxy-tetrahydrodipicolinate reductase [EC:1.17.1.8] |
| 00521 Streptomycin biosynthesis [PATH:ko00521] | | | |
|  | scaffold00001_gene_135 | K15778 pmm-pgm | phosphomannomutase / phosphoglucomutase [EC:5.4.2.8 5.4.2.2] |
|  | scaffold00002_gene_2379 | K15778 pmm-pgm | phosphomannomutase / phosphoglucomutase [EC:5.4.2.8 5.4.2.2] |
|  | scaffold00001_gene_62 | K01092 E3.1.3.25, IMPA, suhB | myo-inositol-1(or 4)-monophosphatase [EC:3.1.3.25] |
|  | scaffold00003_gene_3248 | K01092 E3.1.3.25, IMPA, suhB | myo-inositol-1(or 4)-monophosphatase [EC:3.1.3.25] |
|  | scaffold00004_gene_4399 | K00973 E2.7.7.24, rfbA, rffH | glucose-1-phosphate thymidylyltransferase [EC:2.7.7.24] |
|  | scaffold00004_gene_4397 | K01710 E4.2.1.46, rfbB, rffG | dTDP-glucose 4,6-dehydratase [EC:4.2.1.46] |
|  | scaffold00004_gene_4400 | K01790 rfbC, rmlC | dTDP-4-dehydrorhamnose 3,5-epimerase [EC:5.1.3.13] |
|  | scaffold00004_gene_4398 | K00067 rfbD, rmlD | dTDP-4-dehydrorhamnose reductase [EC:1.1.1.133] |
| 00401 Novobiocin biosynthesis [PATH:ko00401] | | | |
|  | scaffold00003_gene_3888 | K04517 tyrA2 | prephenate dehydrogenase [EC:1.3.1.12] |
|  | scaffold00002_gene_3001 | K00817 hisC | histidinol-phosphate aminotransferase [EC:2.6.1.9] |
|  | scaffold00003_gene_3887 | K00817 hisC | histidinol-phosphate aminotransferase [EC:2.6.1.9] |
|  | scaffold00007_gene_5025 | K00817 hisC | histidinol-phosphate aminotransferase [EC:2.6.1.9] |
|  | scaffold00001_gene_533 | K00832 tyrB | aromatic-amino-acid transaminase [EC:2.6.1.57] |

**Table S22** **Antibiotic resistance assay by using the Kirby-Bauer disk diffusion method.**

| Antibiotic | Disk content (μg/scrip) | Strain HZ01 | |  | *E. coli* ATCC 25922 | |
| --- | --- | --- | --- | --- | --- | --- |
|  |  | Diameter of inhibition zones (mean ± SD, mm) | Susceptibility |  | Diameter of inhibition zones (mean ± SD, mm) | Reference diameter (mm) |
| Ampicillin | 10 | 16.1 ± 1.7 | I |  | 16.4 ± 1.2 | 16–22 |
| Cefotaxime | 30 | 11.1 ± 1.3 | R |  | 33.6 ± 1.3 | 29–35 |
| Azithromycin | 15 | 20.8 ± 1.2 | S |  | 24.5 ± 0.8 | —^a^ |
| Tetracycline | 30 | 21.8 ± 0.7 | S |  | 23.7 ± 1.6 | 18–25 |
| Chloramphenicol | 30 | 22.4 ± 0.7 | S |  | 26.8 ± 1.3 | 21–27 |
| Kanamycin | 30 | 11.3 ± 2.1 | R |  | 18.1 ± 1.3 | 17–25 |
| Bacitracin | 0.04 U | 6.9 ± 0.1 | R |  | 12.6 ± 0.5 | — |
| Gentamicin | 10 | 11.9 ± 0.8 | R |  | 21.8 ± 0.7 | 19–26 |
| Nalidixic acid | 30 | 16.5 ± 1.4 | I |  | 28.2 ± 0.3 | 22–28 |
| Imipenem | 10 | 11.5 ± 0.7 | R |  | 16.9 ± 1.4 | 26–32 |

^a^No record. I, intermediate; R, resistant; S, susceptible.

**Table S23 Candidate genes for drug resistance.**

| Predicted gene | Annotation |
| --- | --- |
| scaffold00004_gene_3960 | multidrug transporter AcrB |
| scaffold00003_gene_3614 | multidrug transporter AcrB |
| scaffold00003_gene_3871 | cation transporter |
| scaffold00002_gene_2718 | outer membrane protein |
| scaffold00001_gene_235 | multidrug efflux RND transporter permease subunit |
| scaffold00003_gene_3620 | acriflavine resistance protein B |
| scaffold00003_gene_3624 | multidrug transporter |
| scaffold00003_gene_3626 | multidrug transporter |
| scaffold00007_gene_4997 | multidrug transporter |
| scaffold00001_gene_1718 | fosmidomycin resistance protein |
| scaffold00004_gene_4094 | membrane fusion protein, multidrug efflux system |
| scaffold00004_gene_4093 | multidrug efflux pump |
| scaffold00004_gene_4092 | multidrug efflux pump |
| scaffold00003_gene_3498 | quaternary ammonium compound-resistance protein QacE |
| scaffold00004_gene_3961 | MexH family multidrug efflux RND transporter periplasmic adaptor subunit |
| scaffold00006_gene_4817 | MexE family multidrug efflux RND transporter periplasmic adaptor subunit |
| scaffold00001_gene_234 | MexX family efflux pump subunit |
| scaffold00003_gene_3619 | MexH family multidrug efflux RND transporter periplasmic adaptor subunit |
| scaffold00002_gene_2647 | MexE family multidrug efflux RND transporter periplasmic adaptor subunit |
| scaffold00001_gene_1571 | MBL fold hydrolase |
| scaffold00001_gene_575 | metallo-beta-lactamase domain protein |
| scaffold00002_gene_2595 | MBL fold metallo-hydrolase |
| scaffold00001_gene_410 | MBL fold hydrolase |
| scaffold00002_gene_2365 | MBL fold metallo-hydrolase |
| scaffold00001_gene_1123 | beta-lactamase |
| scaffold00004_gene_4339 | beta-lactamase |
| scaffold00001_gene_1429 | adeC/adeK/oprM family multidrug efflux complex outer membrane factor |
| scaffold00003_gene_3621 | RND transporter |
| scaffold00002_gene_2695 | bleomycin resistance protein |
| scaffold00002_gene_2648 | multidrug efflux RND transporter permease subunit |

**Table S24** **Antibiotic resistance profile annotation of query sequences (above cutoff value).**

| Query accession | Best hit accession^a^ | Resistance type | Description | Resistance | E-Value |
| --- | --- | --- | --- | --- | --- |
| scaffold00001_gene_72 | CAG76487 | baca | Undecaprenyl pyrophosphate phosphatase, which consists in the sequestration of Undecaprenyl pyrophosphate. | bacitracin | 1e-44 |
| scaffold00002_gene_2648 | CAJ47930 | ceob | Resistance-nodulation-cell division transporter system. Multidrug resistance efflux pump. | chloramphenicol | 0.0 |

^a^NCBI protein accession.

**Table S25** **Detailed information of antibiotic resistance profile annotation (below cutoff hits).**

| Query accession | Best hit accession^a^ | Best hit type | BLAST statistics |
| --- | --- | --- | --- |
| scaffold00003_gene_3625 | YP_442966 | Type: amra  Require: amrb; opra  Description: Resistance-nodulation-cell division transporter system. Multidrug resistance efflux pump.  Resistance: acriflavine; aminoglycoside; macrolide | Score: 206  E-Value: 1e-54  Query Length: 184  Hit Length: 399  HSP Length: 182  Percent Identity: 56.04%  Cutoff Percent Identity: 80% |
| scaffold00001_gene_471 | YP_001453760 | Type: macb  Require: maca; tolc  Description: Resistance-nodulation-cell division transporter system. Multidrug resistance efflux pump. Macrolide-specific efflux system.  Resistance: macrolide | Score: 149  E-Value: 2e-37  Query Length: 256  Hit Length: 648  HSP Length: 238  Percent Identity: 40.76%  Cutoff Percent Identity: 80% |
| scaffold00002_gene_2647 | ZP_01563885 | –^b^ | Score: 360  E-Value: 1e-100  Query Length: 399  Hit Length: 521  HSP Length: 345  Percent Identity: 53.62%  Cutoff Percent Identity: % |
| scaffold00006_gene_4819 | ZP_00967074 | – | Score: 412  E-Value: 1e-116  Query Length: 507  Hit Length: 485  HSP Length: 461  Percent Identity: 49.46%  Cutoff Percent Identity: % |
| scaffold00001_gene_386 | AAY52004 | Type: vanha  Require: vanra; vansa; vana; vanya; vanxa  Description: VanA type vancomycin resistance operon genes, which can synthesize peptidoglycan with modified C-terminal D-Ala-D-Ala to D-alanine--D-lactate.  Resistance: teicoplanin; vancomycin | Score: 46  E-Value: 6e-06  Query Length: 354  Hit Length: 322  HSP Length: 40  Percent Identity: 52.50%  Cutoff Percent Identity: 80% |
| scaffold00003_gene_3620 | YP_001346277 | Type: mexi  Require: opmd; mexh  Description: Resistance-nodulation-cell division transporter system. Multidrug resistance efflux pump.  Resistance: – | Score: 1251  E-Value: 0.0  Query Length: 1029  Hit Length: 1029  HSP Length: 975  Percent Identity: 65.33%  Cutoff Percent Identity: 80% |
| scaffold00001_gene_1581 | AAA73392 | Type: aph6ic  Description: Aminoglycoside O-phosphotransferase, which modifies aminoglycosides by phosphorylation.  Resistance: streptomycin | Score: 264  E-Value: 6e-72  Query Length: 269  Hit Length: 266  HSP Length: 241  Percent Identity: 53.94%  Cutoff Percent Identity: 80% |
| scaffold00001_gene_561 | YP_001438541 | Type: macb  Require: maca; tolc  Description: Resistance-nodulation-cell division transporter system. Multidrug resistance efflux pump. Macrolide-specific efflux system.  Resistance: macrolide | Score: 173  E-Value: 1e-44  Query Length: 233  Hit Length: 647  HSP Length: 223  Percent Identity: 42.60%  Cutoff Percent Identity: 80% |
| scaffold00003_gene_3624 | ZP_00975709 | – | Score: 1313  E-Value: 0.0  Query Length: 1045  Hit Length: 1045  HSP Length: 1033  Percent Identity: 66.12%  Cutoff Percent Identity: % |
| scaffold00001_gene_1429 | YP_001345922 | Type: oprm  Require: mexy; mexb; mexw; mexa; mexv; mexk; mexj; mexx  Description: Resistance-nodulation-cell division transporter system. Multidrug resistance efflux pump.  Resistance: aminoglycoside; beta_lactam; fluoroquinolone; tetracycline; tigecycline | Score: 457  E-Value: 1e-130  Query Length: 487  Hit Length: 485  HSP Length: 461  Percent Identity: 53.80%  Cutoff Percent Identity: 80% |
| scaffold00001_gene_234 | YP_001438873 | Type: acra  Require: acrb; tolc  Description: Resistance-nodulation-cell division transporter system. Multidrug resistance efflux pump.  Resistance: acriflavin; aminoglycoside; beta_lactam; glycylcycline; macrolide | Score: 299  E-Value: 3e-82  Query Length: 418  Hit Length: 401  HSP Length: 359  Percent Identity: 45.96%  Cutoff Percent Identity: 80% |
| scaffold00003_gene_3736 | CAA37477 | Type: otra  Description: Ribosomal protection protein, which protects ribosome from the translation inhibition of tetracycline.  Resistance: tetracycline | Score: 107  E-Value: 4e-24  Query Length: 597  Hit Length: 663  HSP Length: 164  Percent Identity: 40.85%  Cutoff Percent Identity: 80% |
| scaffold00003_gene_3350 | YP_001453760 | Type: macb  Require: tolc; maca  Description: Resistance-nodulation-cell division transporter system. Multidrug resistance efflux pump. Macrolide-specific efflux system.  Resistance: macrolide | Score: 621  E-Value: 1e-179  Query Length: 652  Hit Length: 648  HSP Length: 649  Percent Identity: 51.31%  Cutoff Percent Identity: 80% |
| scaffold00002_gene_2615 | BAE06288 | Type: aad9ib  Description: Aminoglycoside O-nucleotidylyltransferase, which modifies aminoglycosides by adenylylation.  Resistance: spectomycin; streptomycin | Score: 197  E-Value: 1e-51  Query Length: 257  Hit Length: 260  HSP Length: 251  Percent Identity: 41.43%  Cutoff Percent Identity: 80% |
| scaffold00001_gene_1430 | AAQ92181 | Type: mexb  Require: oprm; mexa  Description: Resistance-nodulation-cell division transporter system. Multidrug resistance efflux pump.  Resistance: aminoglycoside; beta_lactam; fluoroquinolone; tetracycline; tigecycline | Score: 1444  E-Value: 0.0  Query Length: 1053  Hit Length: 1048  HSP Length: 1046  Percent Identity: 70.75%  Cutoff Percent Identity: 80% |
| scaffold00004_gene_4272 | YP_001176128 | Type: macb  Require: tolc; maca  Description: Resistance-nodulation-cell division transporter system. Multidrug resistance efflux pump. Macrolide-specific efflux system.  Resistance: macrolide | Score: 55  E-Value: 2e-08  Query Length: 564  Hit Length: 646  HSP Length: 72  Percent Identity: 43.06%  Cutoff Percent Identity: 80% |
| scaffold00001_gene_235 | YP_348488 | Type: mexd  Require: oprj; mexc  Description: Resistance-nodulation-cell division transporter system. Multidrug resistance efflux pump.  Resistance: erythromycin; fluoroquinolone; glycylcycline; roxithromycin | Score: 1051  E-Value: 0.0  Query Length: 1071  Hit Length: 1043  HSP Length: 1035  Percent Identity: 53.04%  Cutoff Percent Identity: 80% |
| scaffold00001_gene_511 | P08457 | Type: sta  Description: Streptothricin acetyltransferase (STAT).  Resistance: streptothricin | Score: 59  E-Value: 2e-10  Query Length: 161  Hit Length: 189  HSP Length: 95  Percent Identity: 42.11%  Cutoff Percent Identity: 80% |
| scaffold00005_gene_4542 | CAA37477 | Type: otra  Description: Ribosomal protection protein, which protects ribosome from the translation inhibition of tetracycline.  Resistance: tetracycline | Score: 116  E-Value: 5e-27  Query Length: 607  Hit Length: 663  HSP Length: 142  Percent Identity: 44.37%  Cutoff Percent Identity: 80% |
| scaffold00006_gene_4818 | AAY92510 | Type: mexf  Require: mexe; oprn  Description: Resistance-nodulation-cell division transporter system. Multidrug resistance efflux pump.  Resistance: chloramphenicol; fluoroquinolone | Score: 1199  E-Value: 0.0  Query Length: 1056  Hit Length: 1059  HSP Length: 1039  Percent Identity: 58.71%  Cutoff Percent Identity: 80% |
| scaffold00003_gene_3293 | CAE12904 | Type: ksga  Description: Specifically dimethylates two adjacent adenosines in the loop of a conserved hairpin near the 3'-end of 16S rRNA in the 30S particle. Its inactivation leads to kasugamycin resistance.  Resistance: kasugamycin | Score: 236  E-Value: 2e-63  Query Length: 263  Hit Length: 272  HSP Length: 266  Percent Identity: 48.87%  Cutoff Percent Identity: 80% |
| scaffold00003_gene_3592 | P25256 | Type: tlrc  Description: ABC transporter system, Macrolide-Lincosamide-Streptogramin B efflux pump.  Resistance: lincosamide; macrolide; streptogramin_b | Score: 53  E-Value: 3e-08  Query Length: 231  Hit Length: 548  HSP Length: 55  Percent Identity: 49.09%  Cutoff Percent Identity: 80% |
| scaffold00001_gene_236 | ZP_00967074 | – | Score: 395  E-Value: 1e-111  Query Length: 494  Hit Length: 485  HSP Length: 484  Percent Identity: 47.52%  Cutoff Percent Identity: % |
| scaffold00007_gene_4997 | CAA05588 | Type: ykkc  Require: ykkd  Description: Small Multidrug Resistance (SMR) protein family. Multidrug resistance efflux pump, which consists of two proteins.  Resistance: na_antimicrobials | Score: 87  E-Value: 4e-19  Query Length: 105  Hit Length: 112  HSP Length: 102  Percent Identity: 42.16%  Cutoff Percent Identity: 80% |
| scaffold00004_gene_4474 | ZP_00155442 | Type: pbp1a  Description: The enzyme has a penicillin-insensitive transglycosylase N-terminal domain (formation of linear glycan strands) and a penicillin-sensitive transpeptidase C-terminal domain (cross-linking of the peptide subunits)  Resistance: penicillin | Score: 424  E-Value: 1e-119  Query Length: 815  Hit Length: 864  HSP Length: 586  Percent Identity: 40.78%  Cutoff Percent Identity: 80% |
| scaffold00003_gene_3623 | YP_442964 | Type: opra  Require: amrb; amra  Description: Resistance-nodulation-cell division transporter system. Multidrug resistance efflux pump.  Resistance: acriflavine; aminoglycoside; macrolide | Score: 389  E-Value: 1e-109  Query Length: 460  Hit Length: 544  HSP Length: 408  Percent Identity: 52.45%  Cutoff Percent Identity: 80% |
| scaffold00003_gene_3221 | AAP46601 | Type: pbp2b  Description: The enzyme has a penicillin-insensitive transglycosylase N-terminal domain (formation of linear glycan strands) and a penicillin-sensitive transpeptidase C-terminal domain (cross-linking of the peptide subunits)  Resistance: penicillin | Score: 461  E-Value: 1e-131  Query Length: 576  Hit Length: 582  HSP Length: 541  Percent Identity: 42.88%  Cutoff Percent Identity: 80% |
| scaffold00001_gene_1431 | EAZ60964 | Type: mexa  Require: mexb; oprm  Description: Resistance-nodulation-cell division transporter system. Multidrug resistance efflux pump.  Resistance: aminoglycoside; beta_lactam; fluoroquinolone; tetracycline; tigecycline | Score: 377  E-Value: 1e-105  Query Length: 392  Hit Length: 383  HSP Length: 350  Percent Identity: 59.14%  Cutoff Percent Identity: 80% |
| scaffold00002_gene_2394 | CAL48457 | Type: dfra26  Description: Group A drug-insensitive dihydrofolate reductase, which can not be inhibited by trimethoprim.  Resistance: trimethoprim | Score: 146  E-Value: 1e-36  Query Length: 162  Hit Length: 183  HSP Length: 166  Percent Identity: 48.19%  Cutoff Percent Identity: 95% |
| scaffold00001_gene_1718 | YP_001477378 | Type: rosa  Require: rosb  Description: Efflux pump/potassium antiporter system. RosA: Major facilitator superfamily transporter. RosB: Potassium antiporter.  Resistance: fosmidomycin | Score: 410  E-Value: 1e-115  Query Length: 412  Hit Length: 406  HSP Length: 368  Percent Identity: 57.61%  Cutoff Percent Identity: 80% |
| scaffold00002_gene_2649 | YP_838976 | Type: opcm  Require: ceob; ceoa  Description: Resistance-nodulation-cell division transporter system. Multidrug resistance efflux pump.  Resistance: chloramphenicol | Score: 456  E-Value: 1e-129  Query Length: 492  Hit Length: 513  HSP Length: 471  Percent Identity: 52.44%  Cutoff Percent Identity: 80% |

^a^NCBI protein accession. ^b^No annotation.

**Table S26 Genes assigned to the “two****-component system” pathway.**

| Predicted gene | Entry/Gene name | Enzyme/Protein |
| --- | --- | --- |
| scaffold00002_gene_2726 | K07636 phoR | two-component system, OmpR family, phosphate regulon sensor histidine kinase PhoR [EC:2.7.13.3] |
| scaffold00002_gene_2725 | K07657 phoB | two-component system, OmpR family, phosphate regulon response regulator PhoB |
| scaffold00002_gene_3104 | K01113 phoD | alkaline phosphatase D [EC:3.1.3.1] |
| scaffold00002_gene_1864 | K02040 pstS | phosphate transport system substrate-binding protein |
| scaffold00003_gene_3402 | K07638 envZ | two-component system, OmpR family, osmolarity sensor histidine kinase EnvZ [EC:2.7.13.3] |
| scaffold00003_gene_3660 | K07638 envZ | two-component system, OmpR family, osmolarity sensor histidine kinase EnvZ [EC:2.7.13.3] |
| scaffold00003_gene_3401 | K07659 ompR | two-component system, OmpR family, phosphate regulon response regulator OmpR |
| scaffold00003_gene_3661 | K07659 ompR | two-component system, OmpR family, phosphate regulon response regulator OmpR |
| scaffold00001_gene_564 | K07662 cpxR | two-component system, OmpR family, response regulator CpxR |
| scaffold00003_gene_3735 | K04771 degP, htrA | serine protease Do [EC:3.4.21.107] |
| scaffold00008_gene_5131 | K04771 degP, htrA | serine protease Do [EC:3.4.21.107] |
| scaffold00004_gene_4094 | K07799 mdtA | membrane fusion protein, multidrug efflux system |
| scaffold00004_gene_4093 | K07788 mdtB | multidrug efflux pump |
| scaffold00004_gene_4092 | K07789 mdtC | multidrug efflux pump |
| scaffold00005_gene_4690 | K07643 basS | two-component system, OmpR family, sensor histidine kinase BasS [EC:2.7.13.3] |
| scaffold00002_gene_2728 | K07644 cusS, copS, silS | two-component system, OmpR family, heavy metal sensor histidine kinase CusS [EC:2.7.13.3] |
| scaffold00003_gene_3454 | K07644 cusS, copS, silS | two-component system, OmpR family, heavy metal sensor histidine kinase CusS [EC:2.7.13.3] |
| scaffold00002_gene_2727 | K07665 cusR, copR, silR | two-component system, OmpR family, copper resistance phosphate regulon response regulator CusR |
| scaffold00003_gene_3453 | K07665 cusR, copR, silR | two-component system, OmpR family, copper resistance phosphate regulon response regulator CusR |
| scaffold00001_gene_236 | K07796 cusC, silC | outer membrane protein, Cu(I)/Ag(I) efflux system |
| scaffold00003_gene_3623 | K07796 cusC, silC | outer membrane protein, Cu(I)/Ag(I) efflux system |
| scaffold00008_gene_5132 | K07645 qseC | two-component system, OmpR family, sensor histidine kinase QseC [EC:2.7.13.3] |
| scaffold00005_gene_4689 | K07666 qseB | two-component system, OmpR family, response regulator QseB |
| scaffold00008_gene_5133 | K07666 qseB | two-component system, OmpR family, response regulator QseB |
| scaffold00001_gene_1416 | K02403 flhD | flagellar transcriptional activator FlhD |
| scaffold00001_gene_1415 | K02402 flhC | flagellar transcriptional activator FlhC |
| scaffold00001_gene_1418 | K02406 fliC | flagellin |
| scaffold00001_gene_1417 | K02405 fliA | RNA polymerase sigma factor for flagellar operon FliA |
| scaffold00001_gene_768 | K02556 motA | chemotaxis protein MotA |
| scaffold00001_gene_1414 | K02556 motA | chemotaxis protein MotA |
| scaffold00003_gene_3488 | K07646 kdpD | two-component system, OmpR family, sensor histidine kinase KdpD [EC:2.7.13.3] |
| scaffold00003_gene_3487 | K07667 kdpE | two-component system, OmpR family, KDP operon response regulator KdpE |
| scaffold00003_gene_3491 | K01546 kdpA | K+-transporting ATPase ATPase A chain [EC:3.6.3.12] |
| scaffold00003_gene_3490 | K01547 kdpB | K+-transporting ATPase ATPase B chain [EC:3.6.3.12] |
| scaffold00003_gene_3489 | K01548 kdpC | K+-transporting ATPase ATPase C chain [EC:3.6.3.12] |
| scaffold00002_gene_3186 | K07649 tctE | two-component system, OmpR family, sensor histidine kinase TctE [EC:2.7.13.3] |
| scaffold00005_gene_4606 | K07649 tctE | two-component system, OmpR family, sensor histidine kinase TctE [EC:2.7.13.3] |
| scaffold00003_gene_3603 | K07774 tctD | two-component system, OmpR family, response regulator TctD |
| scaffold00004_gene_3921 | K07774 tctD | two-component system, OmpR family, response regulator TctD |
| scaffold00005_gene_4607 | K07774 tctD | two-component system, OmpR family, response regulator TctD |
| scaffold00002_gene_3189 | K07793 tctA | putative tricarboxylic transport membrane protein |
| scaffold00002_gene_3187 | K07795 tctC | putative tricarboxylic transport membrane protein |
| scaffold00001_gene_1257 | K07651 resE | two-component system, OmpR family, sensor histidine kinase ResE [EC:2.7.13.3] |
| scaffold00002_gene_2967 | K02259 COX15 | cytochrome c oxidase assembly protein subunit 15 |
| scaffold00002_gene_3162 | K02313 dnaA | chromosomal replication initiator protein |
| scaffold00004_gene_4021 | K03367 dltA | D-alanine--poly(phosphoribitol) ligase subunit 1 [EC:6.1.1.13] |
| scaffold00004_gene_4022 | K03739 dltB | membrane protein involved in D-alanine export |
| scaffold00001_gene_989 | K18072 parS | two-component system, OmpR family, sensor kinase ParS [EC:2.7.13.3] |
| scaffold00001_gene_988 | K18073 parR | two-component system, OmpR family, response regulator ParR |
| scaffold00003_gene_3624 | K18095 mexY, amrB | multidrug efflux pump |
| scaffold00001_gene_1263 | K19611 fepA, pfeA, iroN, pirA | ferric enterobactin receptor |
| scaffold00001_gene_1036 | K01644 citE | citrate lyase subunit beta / citryl-CoA lyase [EC:4.1.3.34] |
| scaffold00004_gene_4358 | K01644 citE | citrate lyase subunit beta / citryl-CoA lyase [EC:4.1.3.34] |
| scaffold00001_gene_1228 | K07673 narX | two-component system, NarL family, nitrate/nitrite sensor histidine kinase NarX [EC:2.7.13.3] |
| scaffold00001_gene_1227 | K07684 narL | two-component system, NarL family, nitrate/nitrite response regulator NarL |
| scaffold00001_gene_1236 | K00370 narG | nitrate reductase alpha subunit [EC:1.7.99.4] |
| scaffold00001_gene_1235 | K00371 narH | nitrate reductase beta subunit [EC:1.7.99.4] |
| scaffold00001_gene_1234 | K00373 narJ | nitrate reductase delta subunit |
| scaffold00001_gene_1233 | K00374 narI | nitrate reductase gamma subunit [EC:1.7.99.4] |
| scaffold00001_gene_1267 | K07686 uhpA | two-component system, NarL family, uhpT operon response regulator UhpA |
| scaffold00001_gene_8 | K07677 rcsC | two-component system, NarL family, capsular synthesis sensor histidine kinase RcsC [EC:2.7.13.3] |
| scaffold00001_gene_1772 | K07687 rcsB | two-component system, NarL family, captular synthesis response regulator RcsB |
| scaffold00004_gene_4177 | K07691 comA | two-component system, NarL family, competent response regulator ComA |
| scaffold00001_gene_5 | K07679 evgS, bvgS | two-component system, NarL family, sensor histidine kinase EvgS [EC:2.7.13.3] |
| scaffold00001_gene_7 | K07690 evgA, bvgA | two-component system, NarL family, response regulator EvgA |
| scaffold00001_gene_1266 | K07777 degS | two-component system, NarL family, sensor histidine kinase DegS [EC:2.7.13.3] |
| scaffold00004_gene_4176 | K07778 desK | two-component system, NarL family, sensor histidine kinase DesK [EC:2.7.13.3] |
| scaffold00001_gene_1347 | K00990 glnD | [protein-PII] uridylyltransferase [EC:2.7.7.59] |
| scaffold00001_gene_1462 | K04751 glnB | nitrogen regulatory protein P-II 1 |
| scaffold00001_gene_953 | K07708 glnL, ntrB | two-component system, NtrC family, nitrogen regulation sensor histidine kinase GlnL [EC:2.7.13.3] |
| scaffold00001_gene_954 | K07712 glnG, ntrC | two-component system, NtrC family, nitrogen regulation response regulator GlnG |
| scaffold00001_gene_952 | K01915 glnA, GLUL | glutamine synthetase [EC:6.3.1.2] |
| scaffold00002_gene_3209 | K01915 glnA, GLUL | glutamine synthetase [EC:6.3.1.2] |
| scaffold00001_gene_1049 | K07713 zraR, hydG | two-component system, NtrC family, response regulator HydG |
| scaffold00004_gene_4109 | K01034 atoD | acetate CoA/acetoacetate CoA-transferase alpha subunit [EC:2.8.3.8 2.8.3.9] |
| scaffold00002_gene_1933 | K01035 atoA | acetate CoA/acetoacetate CoA-transferase beta subunit [EC:2.8.3.8 2.8.3.9] |
| scaffold00004_gene_4106 | K02106 atoE | short-chain fatty acids transporter |
| scaffold00001_gene_199 | K00626 E2.3.1.9, atoB | acetyl-CoA C-acetyltransferase [EC:2.3.1.9] |
| scaffold00002_gene_2940 | K00626 E2.3.1.9, atoB | acetyl-CoA C-acetyltransferase [EC:2.3.1.9] |
| scaffold00002_gene_3076 | K00626 E2.3.1.9, atoB | acetyl-CoA C-acetyltransferase [EC:2.3.1.9] |
| scaffold00006_gene_4805 | K00626 E2.3.1.9, atoB | acetyl-CoA C-acetyltransferase [EC:2.3.1.9] |
| scaffold00002_gene_1877 | K08478 pgtC | phosphoglycerate transport regulatory protein PgtC |
| scaffold00007_gene_4948 | K10125 dctB | two-component system, NtrC family, C4-dicarboxylate transport sensor histidine kinase DctB [EC:2.7.13.3] |
| scaffold00007_gene_4947 | K10126 dctD | two-component system, NtrC family, C4-dicarboxylate transport response regulator DctD |
| scaffold00007_gene_4949 | K11103 dctA | aerobic C4-dicarboxylate transport protein |
| scaffold00001_gene_1047 | K03092 SIG54, rpoN | RNA polymerase sigma-54 factor |
| scaffold00002_gene_3049 | K03092 SIG54, rpoN | RNA polymerase sigma-54 factor |
| scaffold00001_gene_1408 | K00575 cheR | chemotaxis protein methyltransferase CheR [EC:2.1.1.80] |
| scaffold00001_gene_441 | K03406 mcp | methyl-accepting chemotaxis protein |
| scaffold00001_gene_1384 | K03406 mcp | methyl-accepting chemotaxis protein |
| scaffold00001_gene_1386 | K03406 mcp | methyl-accepting chemotaxis protein |
| scaffold00001_gene_1387 | K03406 mcp | methyl-accepting chemotaxis protein |
| scaffold00002_gene_2249 | K03406 mcp | methyl-accepting chemotaxis protein |
| scaffold00001_gene_1409 | K05875 tar | methyl-accepting chemotaxis protein II, aspartate sensor receptor |
| scaffold00001_gene_1383 | K03776 aer | aerotaxis receptor |
| scaffold00001_gene_1410 | K03408 cheW | purine-binding chemotaxis protein CheW |
| scaffold00001_gene_1411 | K03407 cheA | two-component system, chemotaxis family, sensor kinase CheA [EC:2.7.13.3] |
| scaffold00001_gene_1406 | K03413 cheY | two-component system, chemotaxis family, response regulator CheY |
| scaffold00001_gene_1407 | K03412 cheB | two-component system, chemotaxis family, response regulator CheB [EC:3.1.1.61] |
| scaffold00001_gene_715 | K13486 wspC | chemotaxis protein methyltransferase WspC |
| scaffold00001_gene_713 | K13487 wspA | methyl-accepting chemotaxis protein WspA |
| scaffold00001_gene_714 | K13488 wspB | chemotaxis-related protein WspB |
| scaffold00001_gene_716 | K13489 wspD | chemotaxis-related protein WspD |
| scaffold00001_gene_717 | K13490 wspE | two-component system, chemotaxis family, sensor histidine kinase and response regulator WspE |
| scaffold00001_gene_718 | K13491 wspF | two-component system, chemotaxis family, response regulator WspF [EC:3.1.1.61] |
| scaffold00001_gene_719 | K11444 wspR | two-component system, chemotaxis family, response regulator WspR [EC:2.7.7.65] |
| scaffold00002_gene_1853 | K11688 dctP | C4-dicarboxylate-binding protein DctP |
| scaffold00005_gene_4704 | K11690 dctM | C4-dicarboxylate transporter, DctM subunit |
| scaffold00001_gene_1626 | K08357 ttrA | tetrathionate reductase subunit A |
| scaffold00001_gene_1628 | K08358 ttrB | tetrathionate reductase subunit B |
| scaffold00001_gene_1423 | K14986 fixL | two-component system, LuxR family, sensor kinase FixL [EC:2.7.13.3] |
| scaffold00001_gene_1424 | K14987 fixJ | two-component system, LuxR family, response regulator FixJ |
| scaffold00002_gene_3155 | K10004 gltL, aatP | glutamate/aspartate transport system ATP-binding protein [EC:3.6.3.-] |
| scaffold00002_gene_3156 | K10002 gltK, aatM | glutamate/aspartate transport system permease protein |
| scaffold00002_gene_3157 | K10003 gltJ, aatQ | glutamate/aspartate transport system permease protein |
| scaffold00001_gene_1597 | K10001 gltI, aatJ | glutamate/aspartate transport system substrate-binding protein |
| scaffold00002_gene_3185 | K15859 SKN7 | osomolarity two-component system, response regulator SKN7 |
| scaffold00002_gene_2133 | K15011 regB, regS, actS | two-component system, sensor histidine kinase RegB [EC:2.7.13.3] |
| scaffold00002_gene_2134 | K15012 regA, regR, actR | two-component system, response regulator RegA |
| scaffold00002_gene_3016 | K00411 UQCRFS1, RIP1, petA | ubiquinol-cytochrome c reductase iron-sulfur subunit [EC:1.10.2.2] |
| scaffold00002_gene_3017 | K00412 CYTB, petB | ubiquinol-cytochrome c reductase cytochrome b subunit |
| scaffold00002_gene_3018 | K00413 CYC1, CYT1, petC | ubiquinol-cytochrome c reductase cytochrome c1 subunit |
| scaffold00001_gene_257 | K00404 ccoN | cytochrome c oxidase cbb3-type subunit I [EC:1.9.3.1] |
| scaffold00001_gene_258 | K00405 ccoO | cytochrome c oxidase cbb3-type subunit II |
| scaffold00001_gene_260 | K00406 ccoP | cytochrome c oxidase cbb3-type subunit III |
| scaffold00002_gene_2431 | K00425 cydA | cytochrome d ubiquinol oxidase subunit I [EC:1.10.3.-] |
| scaffold00004_gene_4276 | K00425 cydA | cytochrome d ubiquinol oxidase subunit I [EC:1.10.3.-] |
| scaffold00002_gene_2432 | K00426 cydB | cytochrome d ubiquinol oxidase subunit II [EC:1.10.3.-] |
| scaffold00004_gene_4275 | K00426 cydB | cytochrome d ubiquinol oxidase subunit II [EC:1.10.3.-] |
| scaffold00002_gene_2718 | K12340 tolC | outer membrane protein |

**Table S27** **Genes assigned to “cell motility”.**

| Pathway | Predicted gene | Entry/Gene name | Enzyme/Protein |
| --- | --- | --- | --- |
| 02030 Bacterial chemotaxis [PATH:ko02030] | | | |
|  | scaffold00001_gene_441 | K03406 mcp | methyl-accepting chemotaxis protein |
|  | scaffold00001_gene_1384 | K03406 mcp | methyl-accepting chemotaxis protein |
|  | scaffold00001_gene_1386 | K03406 mcp | methyl-accepting chemotaxis protein |
|  | scaffold00001_gene_1387 | K03406 mcp | methyl-accepting chemotaxis protein |
|  | scaffold00002_gene_2249 | K03406 mcp | methyl-accepting chemotaxis protein |
|  | scaffold00001_gene_1409 | K05875 tar | methyl-accepting chemotaxis protein II, aspartate sensor receptor |
|  | scaffold00001_gene_1383 | K03776 aer | aerotaxis receptor |
|  | scaffold00001_gene_942 | K10439 rbsB | ribose transport system substrate-binding protein |
|  | scaffold00001_gene_1411 | K03407 cheA | two-component system, chemotaxis family, sensor kinase CheA [EC:2.7.13.3] |
|  | scaffold00001_gene_1410 | K03408 cheW | purine-binding chemotaxis protein CheW |
|  | scaffold00001_gene_1406 | K03413 cheY | two-component system, chemotaxis family, response regulator CheY |
|  | scaffold00001_gene_1405 | K03414 cheZ | chemotaxis protein CheZ |
|  | scaffold00001_gene_1407 | K03412 cheB | two-component system, chemotaxis family, response regulator CheB [EC:3.1.1.61] |
|  | scaffold00002_gene_2682 | K03411 cheD | chemotaxis protein CheD [EC:3.5.1.44] |
|  | scaffold00001_gene_1408 | K00575 cheR | chemotaxis protein methyltransferase CheR [EC:2.1.1.80] |
|  | scaffold00001_gene_1371 | K02410 fliG | flagellar motor switch protein FliG |
|  | scaffold00001_gene_1377 | K02416 fliM | flagellar motor switch protein FliM |
|  | scaffold00001_gene_1378 | K02417 fliNY, fliN | flagellar motor switch protein FliN/FliY |
|  | scaffold00001_gene_768 | K02556 motA | chemotaxis protein MotA |
|  | scaffold00001_gene_1414 | K02556 motA | chemotaxis protein MotA |
|  | scaffold00001_gene_767 | K02557 motB | chemotaxis protein MotB |
|  | scaffold00001_gene_1413 | K02557 motB | chemotaxis protein MotB |
| 02040 Flagellar assembly [PATH:ko02040] | | | |
|  | scaffold00001_gene_1403 | K02400 flhA | flagellar biosynthesis protein FlhA |
|  | scaffold00001_gene_1404 | K02401 flhB | flagellar biosynthetic protein FlhB |
|  | scaffold00001_gene_1382 | K02421 fliR | flagellar biosynthetic protein FliR |
|  | scaffold00001_gene_1381 | K02420 fliQ | flagellar biosynthetic protein FliQ |
|  | scaffold00001_gene_1380 | K02419 fliP | flagellar biosynthetic protein FliP |
|  | scaffold00001_gene_1378 | K02417 fliNY, fliN | flagellar motor switch protein FliN/FliY |
|  | scaffold00001_gene_1377 | K02416 fliM | flagellar motor switch protein FliM |
|  | scaffold00001_gene_767 | K02557 motB | chemotaxis protein MotB |
|  | scaffold00001_gene_1413 | K02557 motB | chemotaxis protein MotB |
|  | scaffold00001_gene_768 | K02556 motA | chemotaxis protein MotA |
|  | scaffold00001_gene_1414 | K02556 motA | chemotaxis protein MotA |
|  | scaffold00001_gene_1415 | K02402 flhC | flagellar transcriptional activator FlhC |
|  | scaffold00001_gene_1416 | K02403 flhD | flagellar transcriptional activator FlhD |
|  | scaffold00001_gene_1395 | K02390 flgE | flagellar hook protein FlgE |
|  | scaffold00001_gene_1396 | K02389 flgD | flagellar basal-body rod modification protein FlgD |
|  | scaffold00001_gene_1374 | K02413 fliJ | flagellar FliJ protein |
|  | scaffold00001_gene_1373 | K02412 fliI | flagellum-specific ATP synthase [EC:3.6.3.14] |
|  | scaffold00001_gene_1372 | K02411 fliH | flagellar assembly protein FliH |
|  | scaffold00001_gene_1371 | K02410 fliG | flagellar motor switch protein FliG |
|  | scaffold00001_gene_1370 | K02409 fliF | flagellar M-ring protein FliF |
|  | scaffold00001_gene_1369 | K02408 fliE | flagellar hook-basal body complex protein FliE |
|  | scaffold00001_gene_1397 | K02388 flgC | flagellar basal-body rod protein FlgC |
|  | scaffold00001_gene_1398 | K02387 flgB | flagellar basal-body rod protein FlgB |
|  | scaffold00001_gene_1399 | K02386 flgA | flagella basal body P-ring formation protein FlgA |
|  | scaffold00001_gene_1394 | K02391 flgF | flagellar basal-body rod protein FlgF |
|  | scaffold00001_gene_1393 | K02392 flgG | flagellar basal-body rod protein FlgG |
|  | scaffold00001_gene_1392 | K02393 flgH | flagellar L-ring protein precursor FlgH |
|  | scaffold00001_gene_1391 | K02394 flgI | flagellar P-ring protein precursor FlgI |
|  | scaffold00001_gene_1389 | K02396 flgK | flagellar hook-associated protein 1 FlgK |
|  | scaffold00001_gene_1388 | K02397 flgL | flagellar hook-associated protein 3 FlgL |
|  | scaffold00001_gene_1418 | K02406 fliC | flagellin |
|  | scaffold00001_gene_1363 | K02407 fliD | flagellar hook-associated protein 2 |
|  | scaffold00001_gene_1364 | K02422 fliS | flagellar protein FliS |

**Table S28** **Genes assigned to “membrane transport”.**

| Pathway | Predicted gene | Entry/Gene name | Enzyme/Protein |
| --- | --- | --- | --- |
| 02010 ABC transporters [PATH:ko02010] | | | |
|  | scaffold00008_gene_5092 | K02048 cysP, sbp | sulfate transport system substrate-binding protein |
|  | scaffold00008_gene_5093 | K02046 cysU | sulfate transport system permease protein |
|  | scaffold00008_gene_5094 | K02047 cysW | sulfate transport system permease protein |
|  | scaffold00008_gene_5095 | K02045 cysA | sulfate transport system ATP-binding protein [EC:3.6.3.25] |
|  | scaffold00002_gene_2017 | K15576 nrtA, nasF, cynA | nitrate/nitrite transport system substrate-binding protein |
|  | scaffold00002_gene_2016 | K15577 nrtB, nasE, cynB | nitrate/nitrite transport system permease protein |
|  | scaffold00002_gene_2015 | K15578 nrtC, nasD | nitrate/nitrite transport system ATP-binding protein [EC:3.6.3.-] |
|  | scaffold00001_gene_1272 | K15551 tauA | taurine transport system substrate-binding protein |
|  | scaffold00001_gene_1274 | K15552 tauC | taurine transport system permease protein |
|  | scaffold00001_gene_1273 | K10831 tauB | taurine transport system ATP-binding protein [EC:3.6.3.36] |
|  | scaffold00006_gene_4885 | K15554 ssuC | sulfonate transport system permease protein |
|  | scaffold00001_gene_228 | K02020 modA | molybdate transport system substrate-binding protein |
|  | scaffold00001_gene_229 | K02018 modB | molybdate transport system permease protein |
|  | scaffold00001_gene_230 | K02017 modC | molybdate transport system ATP-binding protein [EC:3.6.3.29] |
|  | scaffold00001_gene_900 | K02012 afuA, fbpA | iron(III) transport system substrate-binding protein |
|  | scaffold00001_gene_961 | K02012 afuA, fbpA | iron(III) transport system substrate-binding protein |
|  | scaffold00002_gene_3207 | K02012 afuA, fbpA | iron(III) transport system substrate-binding protein |
|  | scaffold00005_gene_4563 | K02012 afuA, fbpA | iron(III) transport system substrate-binding protein |
|  | scaffold00001_gene_901 | K02011 afuB, fbpB | iron(III) transport system permease protein |
|  | scaffold00001_gene_960 | K02011 afuB, fbpB | iron(III) transport system permease protein |
|  | scaffold00001_gene_959 | K02010 afuC, fbpC | iron(III) transport system ATP-binding protein [EC:3.6.3.30] |
|  | scaffold00001_gene_902 | K02062 thiQ | thiamine transport system ATP-binding protein |
|  | scaffold00004_gene_4150 | K11069 potD | spermidine/putrescine transport system substrate-binding protein |
|  | scaffold00002_gene_3140 | K11070 potC | spermidine/putrescine transport system permease protein |
|  | scaffold00004_gene_4156 | K11070 potC | spermidine/putrescine transport system permease protein |
|  | scaffold00002_gene_3141 | K11071 potB | spermidine/putrescine transport system permease protein |
|  | scaffold00004_gene_4154 | K11071 potB | spermidine/putrescine transport system permease protein |
|  | scaffold00001_gene_1481 | K11072 potA | spermidine/putrescine transport system ATP-binding protein [EC:3.6.3.31] |
|  | scaffold00002_gene_1879 | K11072 potA | spermidine/putrescine transport system ATP-binding protein [EC:3.6.3.31] |
|  | scaffold00002_gene_2640 | K11072 potA | spermidine/putrescine transport system ATP-binding protein [EC:3.6.3.31] |
|  | scaffold00002_gene_3142 | K11072 potA | spermidine/putrescine transport system ATP-binding protein [EC:3.6.3.31] |
|  | scaffold00002_gene_3206 | K11072 potA | spermidine/putrescine transport system ATP-binding protein [EC:3.6.3.31] |
|  | scaffold00005_gene_4710 | K11073 potF | putrescine transport system substrate-binding protein |
|  | scaffold00005_gene_4713 | K11074 potI | putrescine transport system permease protein |
|  | scaffold00005_gene_4712 | K11075 potH | putrescine transport system permease protein |
|  | scaffold00005_gene_4711 | K11076 potG | putrescine transport system ATP-binding protein |
|  | scaffold00003_gene_3688 | K02002 proX | glycine betaine/proline transport system substrate-binding protein |
|  | scaffold00006_gene_4778 | K02002 proX | glycine betaine/proline transport system substrate-binding protein |
|  | scaffold00006_gene_4779 | K02001 proW | glycine betaine/proline transport system permease protein |
|  | scaffold00006_gene_4780 | K02000 proV | glycine betaine/proline transport system ATP-binding protein [EC:3.6.3.32] |
|  | scaffold00001_gene_14 | K10112 msmX, msmK, malK, sugC, ggtA, msiK | multiple sugar transport system ATP-binding protein |
|  | scaffold00001_gene_992 | K10112 msmX, msmK, malK, sugC, ggtA, msiK | multiple sugar transport system ATP-binding protein |
|  | scaffold00002_gene_2283 | K10112 msmX, msmK, malK, sugC, ggtA, msiK | multiple sugar transport system ATP-binding protein |
|  | scaffold00002_gene_3056 | K10112 msmX, msmK, malK, sugC, ggtA, msiK | multiple sugar transport system ATP-binding protein |
|  | scaffold00001_gene_15 | K10227 smoE, mtlE | sorbitol/mannitol transport system substrate-binding protein |
|  | scaffold00002_gene_2285 | K10228 smoF, mtlF | sorbitol/mannitol transport system permease protein |
|  | scaffold00002_gene_2994 | K07323 mlaC | phospholipid transport system substrate-binding protein |
|  | scaffold00002_gene_2992 | K02067 mlaD, linM | phospholipid/cholesterol/gamma-HCH transport system substrate-binding protein |
|  | scaffold00004_gene_4412 | K02067 mlaD, linM | phospholipid/cholesterol/gamma-HCH transport system substrate-binding protein |
|  | scaffold00002_gene_2991 | K02066 mlaE, linK | phospholipid/cholesterol/gamma-HCH transport system permease protein |
|  | scaffold00004_gene_4414 | K02066 mlaE, linK | phospholipid/cholesterol/gamma-HCH transport system permease protein |
|  | scaffold00002_gene_2990 | K02065 mlaF, linL, mkl | phospholipid/cholesterol/gamma-HCH transport system ATP-binding protein |
|  | scaffold00004_gene_4413 | K02065 mlaF, linL, mkl | phospholipid/cholesterol/gamma-HCH transport system ATP-binding protein |
|  | scaffold00001_gene_942 | K10439 rbsB | ribose transport system substrate-binding protein |
|  | scaffold00001_gene_940 | K10440 rbsC | ribose transport system permease protein |
|  | scaffold00005_gene_4592 | K10537 araF | L-arabinose transport system substrate-binding protein |
|  | scaffold00005_gene_4590 | K10538 araH | L-arabinose transport system permease protein |
|  | scaffold00005_gene_4591 | K10539 araG | L-arabinose transport system ATP-binding protein [EC:3.6.3.17] |
|  | scaffold00001_gene_837 | K17321 glpV | glycerol transport system substrate-binding protein |
|  | scaffold00001_gene_834 | K17322 K17322, glpP | glycerol transport system permease protein |
|  | scaffold00001_gene_835 | K17323 glpQ | glycerol transport system permease protein |
|  | scaffold00001_gene_832 | K17324 glpS | glycerol transport system ATP-binding protein |
|  | scaffold00001_gene_833 | K17325 K17325, glpT | glycerol transport system ATP-binding protein |
|  | scaffold00001_gene_995 | K05813 ugpB | sn-glycerol 3-phosphate transport system substrate-binding protein |
|  | scaffold00001_gene_994 | K05814 ugpA | sn-glycerol 3-phosphate transport system permease protein |
|  | scaffold00001_gene_993 | K05815 ugpE | sn-glycerol 3-phosphate transport system permease protein |
|  | scaffold00002_gene_1864 | K02040 pstS | phosphate transport system substrate-binding protein |
|  | scaffold00002_gene_1863 | K02037 pstC | phosphate transport system permease protein |
|  | scaffold00002_gene_1862 | K02038 pstA | phosphate transport system permease protein |
|  | scaffold00002_gene_1861 | K02036 pstB | phosphate transport system ATP-binding protein [EC:3.6.3.27] |
|  | scaffold00002_gene_1975 | K02044 phnD | phosphonate transport system substrate-binding protein |
|  | scaffold00002_gene_2329 | K02044 phnD | phosphonate transport system substrate-binding protein |
|  | scaffold00002_gene_1974 | K02042 phnE | phosphonate transport system permease protein |
|  | scaffold00002_gene_2332 | K02042 phnE | phosphonate transport system permease protein |
|  | scaffold00002_gene_1976 | K02041 phnC | phosphonate transport system ATP-binding protein [EC:3.6.3.28] |
|  | scaffold00002_gene_2330 | K02041 phnC | phosphonate transport system ATP-binding protein [EC:3.6.3.28] |
|  | scaffold00001_gene_922 | K10036 glnH | glutamine transport system substrate-binding protein |
|  | scaffold00001_gene_923 | K10037 glnP | glutamine transport system permease protein |
|  | scaffold00001_gene_924 | K10038 glnQ | glutamine transport system ATP-binding protein [EC:3.6.3.-] |
|  | scaffold00001_gene_1596 | K10040 ABC.GLN1.P | putative glutamine transport system permease protein |
|  | scaffold00001_gene_1597 | K10001 gltI, aatJ | glutamate/aspartate transport system substrate-binding protein |
|  | scaffold00002_gene_3156 | K10002 gltK, aatM | glutamate/aspartate transport system permease protein |
|  | scaffold00002_gene_3157 | K10003 gltJ, aatQ | glutamate/aspartate transport system permease protein |
|  | scaffold00002_gene_3155 | K10004 gltL, aatP | glutamate/aspartate transport system ATP-binding protein [EC:3.6.3.-] |
|  | scaffold00002_gene_1930 | K09969 aapJ, bztA | general L-amino acid transport system substrate-binding protein |
|  | scaffold00002_gene_2619 | K09969 aapJ, bztA | general L-amino acid transport system substrate-binding protein |
|  | scaffold00002_gene_2679 | K09969 aapJ, bztA | general L-amino acid transport system substrate-binding protein |
|  | scaffold00004_gene_4259 | K09969 aapJ, bztA | general L-amino acid transport system substrate-binding protein |
|  | scaffold00002_gene_2678 | K09970 aapQ, bztB | general L-amino acid transport system permease protein |
|  | scaffold00002_gene_2677 | K09971 aapM, bztC | general L-amino acid transport system permease protein |
|  | scaffold00002_gene_2676 | K09972 aapP, bztD | general L-amino acid transport system ATP-binding protein [EC:3.6.3.-] |
|  | scaffold00002_gene_2126 | K02424 fliY | cystine transport system substrate-binding protein |
|  | scaffold00002_gene_2127 | K10009 ABC.CYST.P | cystine transport system permease protein |
|  | scaffold00002_gene_2128 | K10010 ABC.CYST.A | cystine transport system ATP-binding protein [EC:3.6.3.-] |
|  | scaffold00001_gene_987 | K01999 livK | branched-chain amino acid transport system substrate-binding protein |
|  | scaffold00001_gene_1002 | K01999 livK | branched-chain amino acid transport system substrate-binding protein |
|  | scaffold00002_gene_1824 | K01999 livK | branched-chain amino acid transport system substrate-binding protein |
|  | scaffold00002_gene_2825 | K01999 livK | branched-chain amino acid transport system substrate-binding protein |
|  | scaffold00003_gene_3680 | K01999 livK | branched-chain amino acid transport system substrate-binding protein |
|  | scaffold00004_gene_3998 | K01999 livK | branched-chain amino acid transport system substrate-binding protein |
|  | scaffold00004_gene_4077 | K01999 livK | branched-chain amino acid transport system substrate-binding protein |
|  | scaffold00004_gene_4322 | K01999 livK | branched-chain amino acid transport system substrate-binding protein |
|  | scaffold00004_gene_4331 | K01999 livK | branched-chain amino acid transport system substrate-binding protein |
|  | scaffold00001_gene_179 | K01997 livH | branched-chain amino acid transport system permease protein |
|  | scaffold00001_gene_1003 | K01997 livH | branched-chain amino acid transport system permease protein |
|  | scaffold00002_gene_1825 | K01997 livH | branched-chain amino acid transport system permease protein |
|  | scaffold00004_gene_3997 | K01997 livH | branched-chain amino acid transport system permease protein |
|  | scaffold00004_gene_4329 | K01997 livH | branched-chain amino acid transport system permease protein |
|  | scaffold00001_gene_180 | K01998 livM | branched-chain amino acid transport system permease protein |
|  | scaffold00001_gene_456 | K01998 livM | branched-chain amino acid transport system permease protein |
|  | scaffold00001_gene_548 | K01998 livM | branched-chain amino acid transport system permease protein |
|  | scaffold00001_gene_1004 | K01998 livM | branched-chain amino acid transport system permease protein |
|  | scaffold00001_gene_1644 | K01998 livM | branched-chain amino acid transport system permease protein |
|  | scaffold00004_gene_4330 | K01998 livM | branched-chain amino acid transport system permease protein |
|  | scaffold00001_gene_181 | K01995 livG | branched-chain amino acid transport system ATP-binding protein |
|  | scaffold00001_gene_457 | K01995 livG | branched-chain amino acid transport system ATP-binding protein |
|  | scaffold00001_gene_546 | K01995 livG | branched-chain amino acid transport system ATP-binding protein |
|  | scaffold00001_gene_1005 | K01995 livG | branched-chain amino acid transport system ATP-binding protein |
|  | scaffold00003_gene_3682 | K01995 livG | branched-chain amino acid transport system ATP-binding protein |
|  | scaffold00004_gene_3995 | K01995 livG | branched-chain amino acid transport system ATP-binding protein |
|  | scaffold00004_gene_4085 | K01995 livG | branched-chain amino acid transport system ATP-binding protein |
|  | scaffold00004_gene_4328 | K01995 livG | branched-chain amino acid transport system ATP-binding protein |
|  | scaffold00004_gene_4484 | K01995 livG | branched-chain amino acid transport system ATP-binding protein |
|  | scaffold00001_gene_458 | K01996 livF | branched-chain amino acid transport system ATP-binding protein |
|  | scaffold00001_gene_550 | K01996 livF | branched-chain amino acid transport system ATP-binding protein |
|  | scaffold00001_gene_1006 | K01996 livF | branched-chain amino acid transport system ATP-binding protein |
|  | scaffold00002_gene_1827 | K01996 livF | branched-chain amino acid transport system ATP-binding protein |
|  | scaffold00003_gene_3366 | K01996 livF | branched-chain amino acid transport system ATP-binding protein |
|  | scaffold00004_gene_3994 | K01996 livF | branched-chain amino acid transport system ATP-binding protein |
|  | scaffold00004_gene_4325 | K01996 livF | branched-chain amino acid transport system ATP-binding protein |
|  | scaffold00004_gene_4332 | K01996 livF | branched-chain amino acid transport system ATP-binding protein |
|  | scaffold00004_gene_4483 | K01996 livF | branched-chain amino acid transport system ATP-binding protein |
|  | scaffold00002_gene_2093 | K11959 urtA | urea transport system substrate-binding protein |
|  | scaffold00002_gene_2833 | K11959 urtA | urea transport system substrate-binding protein |
|  | scaffold00002_gene_2094 | K11960 urtB | urea transport system permease protein |
|  | scaffold00002_gene_2095 | K11961 urtC | urea transport system permease protein |
|  | scaffold00002_gene_2831 | K11961 urtC | urea transport system permease protein |
|  | scaffold00002_gene_2096 | K11962 urtD | urea transport system ATP-binding protein |
|  | scaffold00002_gene_2830 | K11962 urtD | urea transport system ATP-binding protein |
|  | scaffold00002_gene_2097 | K11963 urtE | urea transport system ATP-binding protein |
|  | scaffold00001_gene_306 | K02073 metQ | D-methionine transport system substrate-binding protein |
|  | scaffold00001_gene_1022 | K02073 metQ | D-methionine transport system substrate-binding protein |
|  | scaffold00003_gene_3253 | K02073 metQ | D-methionine transport system substrate-binding protein |
|  | scaffold00004_gene_4297 | K02073 metQ | D-methionine transport system substrate-binding protein |
|  | scaffold00001_gene_308 | K02072 metI | D-methionine transport system permease protein |
|  | scaffold00003_gene_3252 | K02072 metI | D-methionine transport system permease protein |
|  | scaffold00001_gene_307 | K02071 metN | D-methionine transport system ATP-binding protein |
|  | scaffold00003_gene_3251 | K02071 metN | D-methionine transport system ATP-binding protein |
|  | scaffold00001_gene_663 | K15580 oppA, mppA | oligopeptide transport system substrate-binding protein |
|  | scaffold00002_gene_2796 | K15580 oppA, mppA | oligopeptide transport system substrate-binding protein |
|  | scaffold00002_gene_3038 | K15582 oppC | oligopeptide transport system permease protein |
|  | scaffold00001_gene_665 | K15583 oppD | oligopeptide transport system ATP-binding protein |
|  | scaffold00001_gene_1533 | K15583 oppD | oligopeptide transport system ATP-binding protein |
|  | scaffold00002_gene_2799 | K15583 oppD | oligopeptide transport system ATP-binding protein |
|  | scaffold00002_gene_3174 | K15583 oppD | oligopeptide transport system ATP-binding protein |
|  | scaffold00003_gene_3574 | K15583 oppD | oligopeptide transport system ATP-binding protein |
|  | scaffold00003_gene_3506 | K10823 oppF | oligopeptide transport system ATP-binding protein |
|  | scaffold00001_gene_1443 | K12369 dppB | dipeptide transport system permease protein |
|  | scaffold00001_gene_1535 | K12369 dppB | dipeptide transport system permease protein |
|  | scaffold00002_gene_3037 | K12369 dppB | dipeptide transport system permease protein |
|  | scaffold00001_gene_1442 | K12370 dppC | dipeptide transport system permease protein |
|  | scaffold00001_gene_779 | K12371 dppD | dipeptide transport system ATP-binding protein |
|  | scaffold00003_gene_3385 | K12372 dppF | dipeptide transport system ATP-binding protein |
|  | scaffold00001_gene_332 | K15585 nikB | nickel transport system permease protein |
|  | scaffold00003_gene_3775 | K13889 gsiB | glutathione transport system substrate-binding protein |
|  | scaffold00003_gene_3776 | K13890 gsiC | glutathione transport system permease protein |
|  | scaffold00003_gene_3777 | K13891 gsiD | glutathione transport system permease protein |
|  | scaffold00001_gene_1441 | K13892 gsiA | glutathione transport system ATP-binding protein |
|  | scaffold00003_gene_3774 | K13892 gsiA | glutathione transport system ATP-binding protein |
|  | scaffold00001_gene_151 | K13896 yejF | microcin C transport system ATP-binding protein |
|  | scaffold00002_gene_2402 | K13896 yejF | microcin C transport system ATP-binding protein |
|  | scaffold00001_gene_566 | K02016 ABC.FEV.S | iron complex transport system substrate-binding protein |
|  | scaffold00001_gene_1196 | K02016 ABC.FEV.S | iron complex transport system substrate-binding protein |
|  | scaffold00002_gene_2112 | K02016 ABC.FEV.S | iron complex transport system substrate-binding protein |
|  | scaffold00002_gene_2269 | K02016 ABC.FEV.S | iron complex transport system substrate-binding protein |
|  | scaffold00001_gene_567 | K02015 ABC.FEV.P | iron complex transport system permease protein |
|  | scaffold00001_gene_568 | K02015 ABC.FEV.P | iron complex transport system permease protein |
|  | scaffold00001_gene_1197 | K02015 ABC.FEV.P | iron complex transport system permease protein |
|  | scaffold00001_gene_1198 | K02015 ABC.FEV.P | iron complex transport system permease protein |
|  | scaffold00002_gene_2111 | K02015 ABC.FEV.P | iron complex transport system permease protein |
|  | scaffold00002_gene_2272 | K02015 ABC.FEV.P | iron complex transport system permease protein |
|  | scaffold00004_gene_3931 | K02015 ABC.FEV.P | iron complex transport system permease protein |
|  | scaffold00001_gene_569 | K02013 ABC.FEV.A | iron complex transport system ATP-binding protein [EC:3.6.3.34] |
|  | scaffold00001_gene_1199 | K02013 ABC.FEV.A | iron complex transport system ATP-binding protein [EC:3.6.3.34] |
|  | scaffold00002_gene_2110 | K02013 ABC.FEV.A | iron complex transport system ATP-binding protein [EC:3.6.3.34] |
|  | scaffold00002_gene_2270 | K02013 ABC.FEV.A | iron complex transport system ATP-binding protein [EC:3.6.3.34] |
|  | scaffold00004_gene_3932 | K02013 ABC.FEV.A | iron complex transport system ATP-binding protein [EC:3.6.3.34] |
|  | scaffold00002_gene_2714 | K06858 ABC.VB12.S1, btuF | vitamin B12 transport system substrate-binding protein |
|  | scaffold00003_gene_3677 | K16786 ecfA1 | energy-coupling factor transport system ATP-binding protein [EC:3.6.3.-] |
|  | scaffold00001_gene_974 | K10107 ABC-2.CPSE.P1 | capsular polysaccharide transport system permease protein |
|  | scaffold00002_gene_2919 | K10107 ABC-2.CPSE.P1 | capsular polysaccharide transport system permease protein |
|  | scaffold00004_gene_4426 | K09690 ABC-2.LPSE.P | lipopolysaccharide transport system permease protein |
|  | scaffold00004_gene_4425 | K09691 ABC-2.LPSE.A | lipopolysaccharide transport system ATP-binding protein |
|  | scaffold00001_gene_560 | K09808 lolC_E | lipoprotein-releasing system permease protein |
|  | scaffold00001_gene_561 | K09810 lolD | lipoprotein-releasing system ATP-binding protein [EC:3.6.3.-] |
|  | scaffold00001_gene_1619 | K02195 ccmC | heme exporter protein C |
|  | scaffold00001_gene_1618 | K02194 ccmB | heme exporter protein B |
|  | scaffold00001_gene_1617 | K02193 ccmA | heme exporter protein A [EC:3.6.3.41] |
|  | scaffold00003_gene_3751 | K07091 lptF | lipopolysaccharide export system permease protein |
|  | scaffold00004_gene_4040 | K11720 lptG | lipopolysaccharide export system permease protein |
|  | scaffold00004_gene_4283 | K06861 lptB | lipopolysaccharide export system ATP-binding protein [EC:3.6.3.-] |
|  | scaffold00001_gene_65 | K19341 nosY | Cu-processing system permease protein |
|  | scaffold00001_gene_66 | K19340 nosF | Cu-processing system ATP-binding protein |
|  | scaffold00002_gene_2681 | K09811 ftsX | cell division transport system permease protein |
|  | scaffold00002_gene_2680 | K09812 ftsE | cell division transport system ATP-binding protein |
|  | scaffold00001_gene_1521 | K05658 ABCB1 | ATP-binding cassette, subfamily B (MDR/TAP), member 1 [EC:3.6.3.44] |
|  | scaffold00005_gene_4745 | K11085 msbA | ATP-binding cassette, subfamily B, bacterial MsbA [EC:3.6.3.-] |
|  | scaffold00002_gene_2274 | K14698 irtA | ATP-binding cassette, subfamily B, bacterial IrtA [EC:3.6.3.-] |
|  | scaffold00002_gene_2273 | K14699 irtB | ATP-binding cassette, subfamily B, bacterial IrtB [EC:3.6.3.-] |
|  | scaffold00001_gene_1801 | K12541 lapB | ATP-binding cassette, subfamily C, bacterial LapB |
|  | scaffold00004_gene_4272 | K16012 cydC | ATP-binding cassette, subfamily C, bacterial CydC |
|  | scaffold00004_gene_4273 | K16013 cydD | ATP-binding cassette, subfamily C, bacterial CydD |
|  | scaffold00003_gene_3350 | K05685 macB | macrolide transport system ATP-binding/permease protein [EC:3.6.3.-] |
|  | scaffold00001_gene_318 | K02471 yddA | putative ATP-binding cassette transporter |
| 02060 Phosphotransferase system (PTS) [PATH:ko02060] | | | |
|  | scaffold00001_gene_1766 | K08483 PTS-EI.PTSI, ptsI | phosphotransferase system, enzyme I, PtsI [EC:2.7.3.9] |
|  | scaffold00001_gene_1768 | K02821 PTS-Ula-EIIA, ulaC, sgaA | PTS system, ascorbate-specific IIA component [EC:2.7.1.69] |
|  | scaffold00004_gene_4285 | K02806 PTS-Ntr-EIIA, ptsN | PTS system, nitrogen regulatory IIA component [EC:2.7.1.69] |
| 03070 Bacterial secretion system [PATH:ko03070] | | | |
|  | scaffold00002_gene_2718 | K12340 tolC | outer membrane protein |
|  | scaffold00001_gene_1460 | K02453 gspD | general secretion pathway protein D |
|  | scaffold00001_gene_1461 | K02455 gspF | general secretion pathway protein F |
|  | scaffold00001_gene_1451 | K02456 gspG | general secretion pathway protein G |
|  | scaffold00001_gene_1454 | K02459 gspJ | general secretion pathway protein J |
|  | scaffold00001_gene_1455 | K02460 gspK | general secretion pathway protein K |
|  | scaffold00001_gene_1459 | K02454 gspE | general secretion pathway protein E |
|  | scaffold00002_gene_2028 | K03072 secD | preprotein translocase subunit SecD |
|  | scaffold00002_gene_2029 | K03074 secF | preprotein translocase subunit SecF |
|  | scaffold00004_gene_4499 | K03076 secY | preprotein translocase subunit SecY |
|  | scaffold00002_gene_2027 | K03210 yajC | preprotein translocase subunit YajC |
|  | scaffold00002_gene_3166 | K03217 yidC, spoIIIJ, OXA1 | YidC/Oxa1 family membrane protein insertase |
|  | scaffold00003_gene_3235 | K03070 secA | preprotein translocase subunit SecA |
|  | scaffold00002_gene_2335 | K03110 ftsY | fused signal recognition particle receptor |
|  | scaffold00004_gene_4214 | K03071 secB | preprotein translocase subunit SecB |
|  | scaffold00002_gene_2529 | K03106 SRP54, ffh | signal recognition particle subunit SRP54 [EC:3.6.5.4] |
|  | scaffold00002_gene_3010 | K03117 tatB | sec-independent protein translocase protein TatB |
|  | scaffold00002_gene_3011 | K03118 tatC | sec-independent protein translocase protein TatC |
|  | scaffold00002_gene_2443 | K11904 vgrG | type VI secretion system secreted protein VgrG |
|  | scaffold00002_gene_2448 | K11903 K11903, hcp | type VI secretion system secreted protein Hcp |
|  | scaffold00002_gene_2467 | K11891 impL, vasK, icmF | type VI secretion system protein ImpL |
|  | scaffold00002_gene_2454 | K11892 impK, ompA, vasF, dotU | type VI secretion system protein ImpK |
|  | scaffold00002_gene_2444 | K11907 vasG, clpV | type VI secretion system protein VasG |

**References**

Amin, S., Shah, B., Jain, K., Patel, A., Patel, N., Joshi, C.G., et al. (2015). Draft genome sequence of *Achromobacter* sp. strain DMS1, capable of degrading polyaromatic hydrocarbons isolated from the industrially perturbed environment of Amlakhadi Canal, India. *Genome Announc* 3, e01264-01215. doi: 10.1128/genomeA.01264-15

Chai, L., Wang, Y., Yang, Z., Wang, Q., and Wang, H. (2010). Detoxification of chromium-containing slag by *Achromobacter* sp. CH-1 and selective recovery of chromium. *T Nonferr Metal Soc* 20, 1500–1504. doi: 10.1016/s1003-6326(09)60328-9

Deng, M.C., Li, J., Liang, F.R., Yi, M., Xu, X.M., Yuan, J.P., et al. (2014). Isolation and characterization of a novel hydrocarbon-degrading bacterium *Achromobacter* sp. HZ01 from the crude oil-contaminated seawater at the Daya Bay, southern China. *Mar. Pollut. Bull.* 83, 79–86. doi: 10.1016/j.marpolbul.2014.04.018

Hinteregger, C., and Streichsbier, F. (2001). Isolation and characterization of *Achromobacter xylosoxidans* T7 capable of degrading toluidine isomers. *J. Basic Microbiol.* 41, 159–170.

Kaczorek, E., Salek, K., Guzik, U., Dudzinska-Bajorek, B., and Olszanowski, A. (2013). The impact of long-term contact of *Achromobacter* sp. 4(2010) with diesel oil - Changes in biodegradation, surface properties and hexadecane monooxygenase activity. *Int. Biodeterior. Biodegradation* 78, 7–16. doi: 10.1016/j.ibiod.2012.12.003

Kang, E., Oh, J.M., Lee, J., Kim, Y.C., Min, K.H., Min, K.R., et al. (1998). Genetic structure of the *bphG* gene encoding 2-hydroxymuconic semialdehyde dehydrogenase of *Achromobacter xylosoxidans* KF701. *Biochem. Biophys. Res. Commun.* 246, 20–25. doi: 10.1006/bbrc.1998.8556

Li, X., Hu, Y., Gong, J., Lin, Y., Johnstone, L., Rensing, C., et al. (2012). Genome sequence of the highly efficient arsenite-oxidizing bacterium *Achromobacter arsenitoxydans* SY8. *J. Bacteriol.* 194, 1243–1244. doi: 10.1128/jb.06667-11

Ma, Y.L., Lu, W., Wan, L.L., and Luo, N. (2015). Elucidation of fluoranthene degradative characteristics in a newly isolated *Achromobacter xylosoxidans* DN002. *Appl. Biochem. Biotechnol.* 175, 1294–1305. doi: 10.1007/s12010-014-1347-7

Moon, J., Kang, E., Min, K.R., Kim, C.K., Min, K.H., Lee, K.S., et al. (1997). Characterization of the gene encoding catechol 2,3-dioxygenase from *Achromobacter xylosoxidans* KF701. *Biochem. Biophys. Res. Commun.* 238, 430–435. doi: 10.1006/bbrc.1997.7312

Si, M., Zhao, Y., and Su, T. (2011). Isolation and identification of a high-efficiency alkane-degrading *Achromobacter insolitus* XL strain and its degradation characteristics. *Journal of Soil Science* 42, 562–567.

Strnad, H., Ridl, J., Paces, J., Kolar, M., Vlcek, C., and Paces, V. (2011). Complete genome sequence of the haloaromatic acid-degrading bacterium *Achromobacter xylosoxidans* A8. *J. Bacteriol.* 193, 791–792. doi: 10.1128/jb.01299-10

Trimble, W.L., Phung le, T., Meyer, F., Silver, S., and Gilbert, J.A. (2012). Draft genome sequence of *Achromobacter piechaudii* strain HLE. *J. Bacteriol.* 194, 6355. doi: 10.1128/jb.01660-12

Vedler, E., Koiv, V., and Heinaru, A. (2000). Analysis of the 2,4-dichlorophenoxyacetic acid-degradative plasmid pEST4011 of *Achromobacter xylosoxidans* subsp. *denitrificans* strain EST4002. *Gene* 255, 281–288.

Wan, N., Gu, J., Huang, J., and Gao, C. (2007). Isolation of *Achromobacter xylosoxidans* NS12 and degradation of nitrophenols. *Clinese Journal of Environmental Science* 28, 422–426.
