# Supplementary material for: Genome Sequencing Reveals the Potential of Achromobacter sp. HZ01 for Bioremediation
Source: Front Microbiol. 2017 Aug 9;8:1507. doi: 10.3389/fmicb.2017.01507 (PMC5552670; doi:10.3389/fmicb.2017.01507)
Supplement: Supplementary file 2 [file Presentation_1.pdf]

Supplementary Results and Discussion

Genome Sequencing Reveals the Potential of *Achromobacter* sp. HZ01 for Bioremediation

Yue-Hui Hong[1], Cong-Cong Ye[1], Qian-Zhi Zhou[1], Xiao-Ying Wu[2], Jian-Ping Yuan[1], Juan Peng[1], Hailin Deng[1*] and Jiang-Hai Wang[1*]

[1] Guangdong Provincial Key Laboratory of Marine Resources and Coastal Engineering/South China Sea Bioresource Exploitation and Utilization Collaborative Innovation Center, School of Marine Sciences, Sun Yat-Sen University, Guangzhou, People's Republic of China

[2] State Key Laboratory of Conservation and Utilization of Subtropical Agro-Bioresources, College of Natural Resources and Environment, South China Agricultural University, Guangzhou, People's Republic of China

[*]Correspondence:
Jiang-Hai Wang
wangjhai@mail.sysu.edu.cn
Hailin Deng
denghlin3@mail.sysu.edu.cn

Results

Comparative Genomics

Among the compared genomes, *Achromobacter* sp. HZ01 contains a smaller genome size, less genes and less pseudogenes than other *Achromobacter* strains (Supplementary Table S14). Nevertheless, the GC content (68.10%) in the genome of strain HZ01 is the highest, which is consistent with the previous prediction that containing a high GC content may be a special feature of strain HZ01 (Hong et al., 2016). Among the compared strains, only *Achromobacter xylosoxidans* A8, *Polymorphum gilvum* SL003B-26A1^T and *Geobacillus thermodenitrificans* NG80-2 contain plasmids (Supplementary Table S14).

Taxonomic distribution analysis revealed that 82% of the predicted genes in strain HZ01 were assigned to the genus *Achromobacter* (Supplementary Figure S9). The analysis of genome synteny showed that the genome of strain HZ01 was mostly co-linear with that of *Achromobacter xylosoxidans* A8 and *Achromobacter xylosoxidans* NH44784-1996, respectively (Supplementary Figure S10). Strain A8, isolated from polychlorinated biphenyl-contaminated soil, can use 2,5-dichlorobenzoate and 2-chlorobenzoate as carbon and energy sources (Pavlu et al., 1999), while strain HZ01 is capable of utilizing C_{12}–C_{27} *n*-alkanes and polycyclic aromatic hydrocarbons (PAHs) (Deng et al., 2014). Strain NH44784-1996 is an opportunistic pathogen with resistance to a broad range of antibiotics (Jakobsen et al., 2013). Strain HZ01 also harbors a pathogenic potential with antibiotic resistance (see below).

A phylogenetic tree based on the 16S rRNA genes (including all *Achromobacter* species) showed that strain HZ01 was clustered with *Achromobacter* sp. H2 (Supplementary Figure S11A). The result was similar to that previously described (Deng et al., 2014). It also showed that strain HZ01 had a far evolutionary distance with some hydrocarbon-degrading bacteria. Core and pan genome analysis showed that a total of 12,000 pan genes and 2,643 core genes were obtained from nine *Achromobacter* strains (Supplementary Table S15). Dilution curves of the pan and core genes showed that the number of genes increased and declined in the pan and core genome with each consecutive addition of an *Achromobacter* genome (Supplementary Figure S12). A phylogenetic tree was constructed according to the core genes and showed that strain HZ01 was clustered with *Achromobacter xylosoxidans* A8 and *Achromobacter insolitus* DSM 23807 (Supplementary Figure S11B).

Two-tailed Fisher exact test showed that the gene abundances of five COG categories in strain HZ01, such as “lipid transport and metabolism” (275 genes) and “secondary metabolites biosynthesis, transport, and catabolism” (197 genes), were higher than the levels recorded in the IMG database (Supplementary Table S3). The gene abundances of seven COG categories, such as “carbohydrate transport and metabolism” (274 genes), were lower than that of other genomes in the IMG database. A large number of fatty acids are produced during petroleum degradation (Rojo, 2009). Thus, the genes related to “lipid transport and metabolism” are important for the further degradation of hydrocarbons in strain HZ01. The high abundance of genes related to “secondary metabolites biosynthesis, transport, and catabolism” and low abundance of genes in “carbohydrate transport and metabolism” are consistent with the general features of hydrocarbon-degrading bacteria (Nie et al., 2012).

The differences in gene abundance of COG categories between strain HZ01 and four other bacteria were also evaluated using the two-tailed Fisher exact test. Compared to strains A8, NH44784-1996, SK2, and SL003B-26A1^T, strain HZ01 showed overabundance in “transcription,” “energy production and conversion,” “amino acid transport and metabolism,” “general function prediction only,” and “function unknown” (Supplementary Figure S13). The gene abundance of “cell wall/membrane/envelope biogenesis” in strain HZ01 was also higher than that in hydrocarbon-degrading strains SK2 and SL003B-26A1^T (Supplementary

Figure S13B). Comparisons of COG catalogues showed that strain HZ01 shared 1,683 identical catalogues with strains A8 and NH44784-1996 (Supplementary Figure S14A), suggesting that these three strains (all belonging to the genus *Achromobacter*) may have abundant identical functions. Besides, strain HZ01 shared 952 identical catalogues with strains SK2 and SL003B-26A1^T (Supplementary Figure S14B); contained 38 special COG catalogues which were not included in strain A8 or NH44784-1996 (Supplementary Figure S14A); and contained 330 special COG catalogues which were not found in strain SK2 or SL003B-26A1^T (Supplementary Figure S14B).

Amino Acid Metabolism

KAAS annotation revealed that "amino acid metabolism" was the most abundant pathway consisting of 443 genes (Supplementary Table S16). More specifically, a total of 152 genes were assigned to "biosynthesis of amino acids" (Supplementary Table S17), and strain HZ01 contained 54 tRNAs for carrying and transferring 19 amino acids. The gene coding for tRNA-Ile was not included in the genome. Hydrocarbon-degrading *Polymorphum gilvum* SL003B-26A1^T contained 50 tRNAs for transferring all 20 amino acids, while *Celeribacter indicus* P73^T lacked the tRNA transferring Tyr (Cao et al., 2015). Many genes related to "metabolism of other amino acids" were also identified in strain HZ01, such as genes involved in the metabolism of beta-alanine, taurine, D-glutamate, D-ornithine, and D-alanine (Supplementary Table S16). In addition, genes coding for amino acid transporters were identified, such as (i) *glnH*, *glnP*, and *glnQ* for glutamine transport; (ii) *gltI*, *gltK*, *gltJ*, and *gltL* for glutamate/aspartate transport; (iii) *aapJ*, *aapQ*, *aapM*, and *aapP* for general L-amino acid transport; and (iv) *metQ*, *metI*, and *metN* for D-methionine transport (Supplementary Table S18).

Nitrogen Metabolism

Nitrogen utilization is one of the most important limiting factors for hydrocarbon degradation in oil-degrading bacteria (Mohn and Stewart, 2000). In this study, a total of 32 genes were assigned to the "nitrogen metabolism" pathway (Supplementary Table S19). Strain HZ01 contains *narX*, *narL*, and the genes coding for nitrate reductase (*narG*, *narH*, *narJ*, and *narI*), whereas *narQ* and *narP* are not present in its genome (Figure 6 and Supplementary Figure S15). The sensor-response regulator pairs NarX-NarL and NarQ-NarP control the transcriptional response to nitrate and nitrite (Noriega et al., 2010). The NarQ regulates the phosphorylation of NarL and NarP with similar phosphotransferase activities, while the NarX exhibits a marked preference for the NarL phosphorylation. Either the NarQ or NarX is sufficient to activate the NarL-dependent transcription of the *narGHJI* operon. The identified genes for nitrate metabolism in strain HZ01 is consistent with its ability to use NH_4NO_3 as a nitrogen source. In addition to the *narGHJI* operon, genes involved in the downstream steps of denitrification were also identified in the genome, including *napA* (gene_1614), *napB* (gene_1615), *nirK* (gene_1662), *norB* (gene_1665), and *nosZ* (gene_68).

Glutamine synthetase (GS), encoded by *glnA* (GSI; gene_952 and gene_3209), *glnII* (GSII) or *glnT* (GSIII), is essential for nitrogen assimilation (Bravo and Mora, 1988). The synthesis and activity of GSI (GlnA) are regulated by the Ntr system which is present in strain HZ01 (Figure 6). Under the condition of low nitrogen availability, GlnB (gene_1462) is uridylylated by GlnD (gene_1347) and thus become inactive. Subsequently, NtrB (gene_953) catalyzes the phosphorylation of NtrC (gene_954), leading to GlnA production and nitrogen assimilation (Schluter et al., 2000). These genes are clearly beneficial for the nitrogen metabolism in strain HZ01, which may indirectly contribute to its performance on hydrocarbon degradation (Gao et al., 2014).

Biosynthesis of Other Secondary Metabolites

Seven gene clusters related to secondary metabolites were predicted using the antiSMASH software (Supplementary Figure S16 and Supplementary Table S20), including "terpene," "phosphonate," "siderophore," "t1pks," "arylpolyene," "resorcinol," and "ectoine."

Terpenes, consisting of repeated isoprene (C_5H_8) units, are widely used, e.g., as mouthwashes, inhalations, gargles, insecticides, and flavoring agents (Aqil et al., 2007). They are also an effective and safe class of penetration enhancers and are classified as generally regarded as safe (GRAS) by the Food and Drug Administration.

Phosphorus is needed for growth in all organisms. Most Gram-negative bacteria utilize phosphonate as phosphorus source under phosphate-limiting conditions (Kamat et al., 2011). A number of genes, including one gene cluster for phosphonate transport and metabolism, were identified in strain HZ01, such as *phnD* (gene_1975 and gene_2329), *phnE* (gene_1974 and gene_2332), and *phnC* (gene_1976 and gene_2330) for phosphonate transport, and *phnF* (gene_1966) for regulation of phosphonate metabolism. Operon *phnGHIJKLM* (corresponding to gene_1964, gene_1963, gene_1962, gene_1961, gene_1960, gene_1959, and gene_1958, respectively) has been demonstrated to encode proteins for the conversion of phosphonate to phosphate (Metcalf and Wanner, 1993). These genes are beneficial for the utilization of phosphorus element in strain HZ01.

Iron is an essential element in most organisms and is involved in diverse metabolic and informational cellular pathways (Miethke and Marahiel, 2007). Many enzymes acting in metabolism harbor iron-containing cofactors. Marine microorganisms produce siderophores to cope with the extremely low availability of dissolved iron in marine environments (Schneiker et al., 2006). One gene cluster for siderophore production is contained in strain HZ01 (Supplementary Figure S16 and Supplementary Table S20), which facilitates its iron uptake under iron-limiting conditions.

Type-I polyketide synthases (t1pks) catalyze the production of polyketides that are found across bacteria, fungi, and plants (Bhetariya et al., 2016). The polyketides are a structurally diverse and industrially well-exploited group of secondary metabolites with promising medicinal importance due to their antibacterial, antiviral, antitumor, anti-pathogenic, antituberculous, and immunosuppressive effects (Manivasagan et al., 2013).

Aryl polyenes, an abundant class of lipids with an aryl head group conjugated to a polyene tail, are widespread in Gram-negative bacteria (Schoner et al., 2016). The aryl polyenes are structurally similar to carotenoids and protect bacteria from exogenous oxidative stress that will otherwise cause damage to cellular components (Cimermancic et al., 2014).

Resorcinol is found in diverse environments since it is widely used in tire and rubber processing, antiseptic and disinfectant preparations, and adhesive production (Lynch et al., 2002). In addition to chemical synthesis, resorcinol is produced in nature as secondary products. It had been reported that resorcinol was subjected to catabolism via three metabolic pathways in bacteria (Li et al., 2012). In *Azotobacter vinelandii*, resorcinol was converted into pyrogallol, which was further processed by pyrogallol 1,2-dioxygenase. Some bacteria converted resorcinol into hydroxyquinol which was subsequently degraded by hydroxyquinol 1,2-dioxygenase (*ortho*-cleavage) or 2,3,5-trihydroxytoluene 1,2-dioxygenase (*meta*-cleavage). All these pathways were initiated by hydroxylation under the action of resorcinol hydroxylase (Huang et al., 2006). One gene cluster was contained in strain HZ01 for resorcinol metabolism (Supplementary Figure S16 and Supplementary Table S20). TetR family transcriptional regulator (gene_4231, gene_4458, gene_4461, and gene_4464), hydroxyquinol 1,2-dioxygenase (gene_378 and gene_4830) and maleylacetate reductase (gene_473) were identified, whereas resorcinol hydroxylase was not found.

Ectoine is a pyrimidine derivative produced by the intramolecular dehydration of N-acetylated diaminobutyrate (Kuhlmann and Bremer, 2002). This highly water-soluble

molecule does not carry a net charge at physiological pH and can be accumulated in the cell to a high concentration without affecting vital physiological functions. In addition, ectoine has a stabilizing influence on the native structure of DNA, proteins, and cell membranes. It helps the cell resist various harsh conditions, such as high osmosis, high temperature, radiation, and desiccation. Strain HZ01 exhibits a favorable adaptation to a wide range of salinity, as it can degrade approximately 55% of diesel oil (2%, w/v) in a minimal salt medium (MSM) containing 5% (w/v) NaCl (Deng et al., 2014). One mechanism for its osmoregulation may be related to the synthesis of compatible solutes (e.g. ectoine) as osmoprotectants. Indeed, one gene cluster for the biosynthesis of ectoine was identified in strain HZ01, which might contribute to its resistance to consistently high salinity in marine environments.

Additionally, numerous genes related to the biosynthesis of other secondary metabolites are also contained in strain HZ01, mainly including the genes involved in the production of ansamycins, tetracycline, vancomycin, isoquinoline alkaloid, pyridine alkaloid, penicillin, cephalosporin, streptomycin, and novobiocin, respectively (Supplementary Table S21). The presence of abundant genes for biosynthesis of secondary metabolites suggests that marine bacterium strain HZ01 exhibits a biotechnological potential for producing valuable natural compounds.

Antibiotic Resistance

In this study, the antibiotic susceptibility of strain HZ01 was investigated using *E. coli* ATCC 25922 for quality control. As shown in Supplementary Table S22, almost all the diameters of inhibition zones from *E. coli* ATCC 25922 were within the reference values. Strain HZ01 was susceptible to azithromycin, tetracycline, and chloramphenicol, respectively. It had an intermediate susceptibility to ampicillin and nalidixic acid, respectively. Additionally, this bacterium was resistant to cefotaxime, kanamycin, bacitracin, gentamicin, and imipenem, respectively (Supplementary Table S22 and Supplementary Figure S17). Mechanisms associated with antibiotic resistance in bacteria include: (i) elimination of the antibiotics by efflux pumps; (ii) catabolism or inactivation of the drugs by enzymes; (iii) modifications to the antibiotic targets; (iv) elimination or reduction of porins through which the antibiotics enter the cell; and (v) activation of alternative pathways to bypass the drug action (Liu and Pop, 2009). Strain HZ01 harbors candidate genes for drug resistance (Supplementary Tables S23, S24 and S25), such as genes coding for metallo-β-lactamase (gene_410, gene_575, gene_1571, and gene_2595) associated with the resistance to carbapenems (including imipenem) (Bush and Jacoby, 2010). AcrAB-TolC, belonging to the resistance-nodulation-division (RND) family and consisting of AcrA, AcrB, and TolC, is a representative multidrug resistance efflux pump that can eliminate diverse antibiotics from the cell, such as ampicillin, tetracycline, chloramphenicol, and nalidixic acid (Nikaido, 1996). Genes coding for AcrB (gene_3614, gene_3871, and gene_3960) and TolC (gene_2718) were included in strain HZ01, whereas the gene encoding AcrA was not identified. AcrA, a periplasmic protein connecting with AcrB and TolC, is essential for the pump complex to function, as it can activate the efflux pump protein AcrB (Elkins and Nikaido, 2003). Lacking the AcrA protein may be one of the reasons explaining why strain HZ01 is susceptible to tetracycline and chloramphenicol and has an intermediate susceptibility to ampicillin and nalidixic acid, respectively (Supplementary Table S22). In addition, *tet* genes, generally associated with tetracycline resistance (Levy et al., 1999), are not present in the genome, which may be another reason for the susceptibility of strain HZ01 to tetracycline. Bacterial resistance to aminoglycosides is related to the following mechanisms: (i) reduction in the uptake and accumulation of drugs; (ii) modifications to the ribosome binding sites; and (iii) inactivation of the antibiotics by enzymes. The enzymatic inactivation is the primary

mechanism for the aminoglycoside resistance, involving three categories of modifying enzymes, i.e., aminoglycoside phosphotransferase (gene_9 and gene_3290), nucleotidyltransferase (gene_3841), and acetyltransferase (gene_1655, gene_2156, gene_3476, gene_3953, gene_4656, and gene_4753) (Chow, 2000). The existence of these enzymes in strain HZ01 is likely associated with its resistance to kanamycin and gentamicin. Beta-lactamase (gene_1123 and gene_4339), which is the most important enzyme related to cephalosporin resistance (Livermore, 1995), is contained in strain HZ01, accounting for its resistance to cefotaxime.

Annotation by the ARDB revealed that among the 5,162 query sequences, 2 and 31 sequences had similarity above and below the cutoff value, respectively (Supplementary Tables S24 and S25). The results also showed that strain HZ01 might be resistant to bacitracin and chloramphenicol, of which the former prediction was consistent with the results of antibiotic resistance assay, while the latter was not (Supplementary Table S22). Bacterial resistance to chloramphenicol is associated with several mechanisms, including (i) inactivation of the antibiotic by acetylation using chloramphenicol acetyltransferases, (ii) inactivation by 3-*O*-phosphotransferase, (iii) permeability change of the membrane, (iv) mutation of target sites, and (v) drug elimination by efflux systems, of which enzymatic inactivation is the primary mechanism (Schwarz et al., 2004). Although strain HZ01 contains multidrug resistance efflux pumps (Supplementary Tables S23 and S24), neither the chloramphenicol acetyltransferase nor the 3-*O*-phosphotransferase, essential for the enzymatic inactivation of chloramphenicol, is included in the genome. It may be drawn a conclusion that the existence of efflux pumps is not sufficient for strain HZ01 to become resistant to chloramphenicol.

Overall, strain HZ01 may be regarded as a multidrug-resistant (MDR) bacterium because it is at least resistant to four categories of antibiotics (Supplementary Table S22), including cephalosporins (cefotaxime), aminoglycosides (kanamycin and gentamicin), polypeptides (bacitracin), and carbapenems (imipenem).

Two-component Systems and Cell Motility

Two-component systems play a key role in bacterial responses to a variety of environmental changes, regulating most physiological processes, such as protein synthesis, chemotaxis, osmoregulation, sporulation, biosynthesis of secondary metabolites, biofilm formation, and quorum sensing (Hsing et al., 1998). A total of 130 genes in strain HZ01 were assigned to "two-component system" (Supplementary Table S26). For instance, the Kdp system, including KdpD, KdpE, KdpA, KdpB, and KdpC, is included in strain HZ01, of which KdpD and KdpE belong to the two-component system. The Kdp system plays an important role in maintaining the stability of potassium concentration in the cell (Voelkner et al., 1993). Regulatory system CusR (gene_2727 and gene_3453) and CusS (gene_2728 and gene_3454) are involved in the regulation of metal responsive genes (Munson et al., 2000). The expression of outer membrane protein CusC (gene_236), regulated by the *cusRS*, is induced by high concentrations of copper ions to maintain the intracellular copper levels within a safe range. Strain HZ01 also contains the Wsp chemosensory system (Figure 6), which is composed of WspA (gene_713), WspB (gene_714), WspD (gene_716), WspE (gene_717), WspC (gene_715), WspR (gene_719), and WspF (gene_718). The Wsp chemosensory system plays an important role in the formation of cyclic diguanylate (c-di-GMP) which is an intracellular signaling molecule (Hickman et al., 2005). It has been demonstrated that the intracellular levels of c-di-GMP control the expression of genes related to exopolysaccharide (EPS) production, and that increased levels of c-di-GMP result in enhanced biofilm formation.

DesKR is a cold stress-related two-component system. Under low-temperature

conditions, DesK (gene_4176) changes its conformation through autophosphorylation and subsequently activates DesR to induce the expression of *des*, leading to the changes of membrane fluidity (Aguilar et al., 2001). The expression of porins OmpC and OmpF, both related to osmoregulation, is under the regulation of the EnvZ/OmpR two-component system (Hsing and Silhavy, 1997). Under the condition of high osmotic pressure, EnvZ (gene_3402 and gene_3660) activates OmpR (gene_3401 and gene_3661) through phosphorylation. Then, the activated OmpR regulates the transcription of *ompC* and *ompF*.

Regulatory system RegB/RegA is widely distributed among Proteobacteria. Upon sensing a redox signal, the histidine kinase RegB (gene_2133) phosphorylates itself on a conserved histidine residue and subsequently transfers the phosphate to RegA (gene_2134) (Elsen et al., 2004). RegA controls a variety of energy-generating and energy-utilizing cellular processes, such as nitrogen fixation, denitrification, photosynthesis, aerobic and anaerobic respiration, hydrogen oxidation, carbon fixation, aerotaxis, and electron transport. Among these biological processes, the electron transfer system and aerobic respiration are contained in strain HZ01 (Supplementary Figure S18).

The EvgS/EvgA two-component system, related to drug resistance and regulation of virulence factors, is included in strain HZ01. EvgS (gene_5) senses environmental signals and modulates transcriptional regulator EvgA (gene_7) via phosphorylation. Thereafter, EvgA stimulates the transcription of *emrKY* that encodes multidrug efflux pumps, finally leading to antibiotic resistance (Kato et al., 2000). Genes *qseB* (gene_4689 and gene_5133) and *qseC* (gene_5132) encode response regulator and sensor kinase, respectively. The QseBC is one component of the quorum-sensing regulatory cascade and activates the transcription of *flhDC* (gene_1416 and gene_1415), which regulates the expression of flagella and motility genes (Sperandio et al., 2002). Other two-component systems were shown in Supplementary Table S26, such as (i) *comA* (gene_4177), associated with the competence development in bacteria (Weinrauch et al., 1989), and (ii) PhoR/PhoB (gene_2726 and gene_2725) related to the regulation of phosphate assimilation (von Kruger et al., 1999). In summary, the identified two-component systems provide strain HZ01 with a genetic basis for responding to rapidly changing marine environments.

Chemotaxis occurs when microorganisms move along chemical gradients to seek an advantageous environment or to escape from adverse conditions (Hazelbauer, 2012). Genome annotation revealed that a total of 55 genes were assigned to “cell motility,” including 22 genes involved in chemotaxis and 33 genes related to flagellar assembly (Supplementary Table S27). For instance, several chemotaxis family proteins were identified, such as CheW, CheA, CheR, CheY, and CheB. Strain HZ01 contains MCP for alkane chemotaxis, as mentioned above. It also harbors chemotaxis proteins (MotA and MotB) and abundant flagellar biosynthesis proteins for flagellar assembly (Supplementary Figure S19), which is in accord with the fact that strain HZ01 is a motile bacterium (Deng et al., 2014). In chemotaxis, MCP binds chemoattractants and signals CheA to phosphorylate CheY (Guvener and Harwood, 2007). Then, the phosphorylated CheY affects swimming behaviours and chemotactic responses. CheA also regulates the phosphorylation of CheB. The phosphorylated CheB modifies the methylation state of MCP, thereby affecting the intensity of the signalling pathway. The flagellar assembly in motile strain HZ01 is clearly beneficial for cell motility and helps this bacterium move to a relatively better niche to pursue nutrients and avoid environmental damages in the marine sites.

Membrane Transport

Effective uptake of major nutrient elements, such as N, P, and S, is crucial for bacteria to survive in the oligotrophic marine environments. Membrane transport clearly plays a key role in this process. In this study, genome annotation revealed that a total of 240 genes were

assigned to “membrane transport” (Supplementary Table S28). “ABC transporters” was the most abundant subcategories among the annotated metabolic pathways. Strain HZ01 contains *nrtA* (gene_2017), *nrtB* (gene_2016), and *nrtC* (gene_2015) for nitrate/nitrite transport. The intact operon *urtABCDE* coding for an ABC-type urea permease was identified, which was consistent with the fact that strain HZ01 was also capable of utilizing urea other than nitrate as a nitrogen source (Deng et al., 2014).

Generally, bacteria can utilize three classes of phosphorus sources, including inorganic phosphates (Pi), organophosphates, and phosphonates (Pn). Pst system is a typical ATP-binding cassette (ABC) transporter composed of *phoU* (gene_4721), *pstS* (gene_1864), *pstC* (gene_1863), *pstA* (gene_1862), and *pstB* (gene_1861) (da Costa Vasconcelos et al., 2016). This system is contained in strain HZ01 for Pi uptake. As mentioned above, *phnD*, *phnE*, and *phnC* are included in the genome for phosphonate transport. The organophosphates are transported in the form of sn-glycerol 3-phosphate by the UgpBAEC system, which is composed of *ugpB* (gene_995), *ugpA* (gene_994), *ugpE* (gene_993), and *ugpC* (gene_2283 and gene_3056) (Metcalf et al., 1990).

Strain HZ01 contains a sulfate transport system consisting of *cysP* (gene_5092), *cysU* (gene_5093), *cysW* (gene_5094), and *cysA* (gene_5095). Several proteins for the transport of inorganic ions were also identified, such as cation:proton antiporter (gene_854), putative calcium/sodium:proton antiporter (gene_3414), sodium:proton antiporter (gene_4345), Na^+/Pi cotransporter (gene_4553), potassium transporter Trk (gene_4221), cobalt-zinc-cadmium resistance protein (gene_3873 and gene_4935), and nickel/cobalt efflux system RcnA (gene_1120) (Figure 6).

Strain HZ01 contains several transporters for scavenging trace elements, such as Mo and Fe (Supplementary Table S28). For molybdate transport, ModA (gene_228), ModB (gene_229), and ModC (gene_230) were identified, whereas ModF was not included in the genome. Strain HZ01 contains an intact transport system for Fe^{3+} uptake, consisting of AfuA (gene_900, gene_961, gene_3207, and gene_4563), AfuB (gene_901 and gene_960), and AfuC (gene_959). Besides, its genome harbors an iron complex transport system composed of FhuD, FhuB, and FhuC (Supplementary Table S28).

In addition to possessing the transporters for nutrient uptake, strain HZ01 also contains secretion systems for substance export. Genome annotation revealed that 23 genes were enriched in three bacterial secretion systems (Supplementary Table S28), including Types I, II and VI secretion systems. Only one gene (gene_2718) was assigned to the Type I secretion system (Supplementary Figure S20). The Type II secretion system widely exists in Gram-negative bacteria and mediates the extracellular transport of various proteins, such as proteases, biofilm matrix proteins, chitinases, pectinases, cellulases, phosphoesterases, and toxins (Rule et al., 2016). The Type VI secretion system is a single-step secretion system widely distributed amongst diverse Gram-negative bacteria and is dedicated to the injection of effector proteins into target cells (Journet and Cascales, 2016). This secretion system is currently believed to involve in pathogenesis as well as interbacterial competition. Additionally, strain HZ01 contains virulence factors, such as colicin V synthesis protein (gene_1354), cell envelope protein (gene_2626), PgaB (gene_1715), PgaD (gene_1717), type VI secretion protein ImpA (gene_2470), and uncharacterized protein ImpD (gene_2448). The existence of pathogenic factors and Types II and VI secretion systems in strain HZ01 suggests that this Gram-negative bacterium exhibits a pathogenic potential.

Discussion

Oceans represent an exhaustive and huge source of natural compounds. Diverse marine bacteria usually possess distinct metabolic pathways and physiological functions to thrive in their habitats and produce novel compounds that are rarely present in the terrestrial microbes.

It had been reported that marine strain HZ01 might be a novel bacterium belonging to the genus *Achromobacter* (Deng et al., 2014). This strain contains numerous genes for biosynthesis of secondary metabolites, such as biosurfactants, polyketides, and antibiotics. Additionally, a total of 562 and 469 genes were annotated as "general function prediction only" and "function unknown," respectively (Supplementary Table S3). Thus, strain HZ01 exhibits a biotechnological potential for producing novel natural products of commercial or medicinal importance.

To the best of our knowledge, genome analysis revealing the pathogenic and drug-resistant aspects of hydrocarbon-degrading bacteria is currently scarce. Certain strains of *Achromobacter xylosoxidans*, such as *Achromobacter xylosoxidans* NH44784-1996 (Jakobsen et al., 2013), were resistant to a broad range of drugs. The genome of strain HZ01 showed a high similarity to that of *Achromobacter xylosoxidans* NH44784-1996 (Supplementary Figure S10). Therefore, we analyzed the antibiotic resistance and pathogenic factors in strain HZ01. The results suggest that strain HZ01 harbors a pathogenic potential and may be regarded as a multidrug-resistant bacterium. These data provide us with a new insight into the physiological characters of marine oil-degrading bacteria, which contributes to the future genetic manipulations of the original strains for various applications. Although the pathogenicity is a notorious feature and may cause negative impacts, some pathogenic bacteria, such as *Shewanella* strains and *Pseudomonas aeruginosa*, have been used directly or indirectly in several fields, such as bioremediation and biosurfactant production (Hau and Gralnick, 2007; Cha et al., 2008). To purposefully pursue the merits of strain HZ01, gene engineering technique may be an appropriate method.

References

Aguilar, P.S., Hernandez-Arriaga, A.M., Cybulski, L.E., Erazo, A.C., and de Mendoza, D. (2001). Molecular basis of thermosensing: a two-component signal transduction thermometer in *Bacillus subtilis*. *EMBO J.* 20, 1681–1691. doi: 10.1093/emboj/20.7.1681

Aqil, M., Ahad, A., Sultana, Y., and Ali, A. (2007). Status of terpenes as skin penetration enhancers. *Drug Discov. Today* 12, 1061–1067. doi: 10.1016/j.drudis.2007.09.001

Bhetariya, P.J., Prajapati, M., Bhaduri, A., Mandal, R.S., Varma, A., Madan, T., et al. (2016). Phylogenetic and structural analysis of polyketide synthases in *Aspergilli*. *Evol. Bioinform. Online* 12, 109–119. doi: 10.4137/ebo.s32694

Bravo, A., and Mora, J. (1988). Ammonium assimilation in *Rhizobium phaseoli* by the glutamine synthetase-glutamate synthase pathway. *J. Bacteriol.* 170, 980–984. doi: 10.1128/jb.170.2.980-984.1988

Bush, K., and Jacoby, G.A. (2010). Updated functional classification of beta-lactamases. *Antimicrob. Agents Chemother.* 54, 969–976. doi: 10.1128/aac.01009-09

Cao, J., Lai, Q., Yuan, J., and Shao, Z. (2015). Genomic and metabolic analysis of fluoranthene degradation pathway in *Celeribacter indicus* P73^T. *Sci. Rep.* 5:7741. doi: 10.1038/srep07741

Cha, M., Lee, N., Kim, M., Kim, M., and Lee, S. (2008). Heterologous production of *Pseudomonas aeruginosa* EMS1 biosurfactant in *Pseudomonas putida*. *Bioresour. Technol.* 99, 2192–2199. doi: 10.1016/j.biortech.2007.05.035

Chow, J.W. (2000). Aminoglycoside resistance in enterococci. *Clin. Infect. Dis.* 31, 586–589. doi: 10.1086/313949

Cimermancic, P., Medema, M.H., Claesen, J., Kurita, K., Wieland Brown, L.C., Mavrommatis, K., et al. (2014). Insights into secondary metabolism from a global

analysis of prokaryotic biosynthetic gene clusters. *Cell* 158, 412–421. doi: 10.1016/j.cell.2014.06.034

da Costa Vasconcelos, F.N., Padilla, G., and Spira, B. (2016). *Chromobacterium violaceum* adaptation to low-phosphate conditions. *Arch. Microbiol.* 198, 269–277. doi: 10.1007/s00203-016-1188-6

Deng, M.C., Li, J., Liang, F.R., Yi, M., Xu, X.M., Yuan, J.P., et al. (2014). Isolation and characterization of a novel hydrocarbon-degrading bacterium *Achromobacter* sp. HZ01 from the crude oil-contaminated seawater at the Daya Bay, southern China. *Mar. Pollut. Bull.* 83, 79–86. doi: 10.1016/j.marpolbul.2014.04.018

Elkins, C.A., and Nikaido, H. (2003). Chimeric analysis of AcrA function reveals the importance of its C-terminal domain in its interaction with the AcrB multidrug efflux pump. *J. Bacteriol.* 185, 5349–5356. doi: 10.1128/JB.185.18.5349-5356.2003

Elsen, S., Swem, L.R., Swem, D.L., and Bauer, C.E. (2004). RegB/RegA, a highly conserved redox-responding global two-component regulatory system. *Microbiol. Mol. Biol. Rev.* 68, 263–279. doi: 10.1128/mmbr.68.2.263-279.2004

Gao, Y.C., Guo, S.H., Wang, J.N., Li, D., Wang, H., and Zeng, D.H. (2014). Effects of different remediation treatments on crude oil contaminated saline soil. *Chemosphere* 117, 486–493. doi: 10.1016/j.chemosphere.2014.08.070

Guvener, Z.T., and Harwood, C.S. (2007). Subcellular location characteristics of the *Pseudomonas aeruginosa* GGDEF protein, WspR, indicate that it produces cyclic-di-GMP in response to growth on surfaces. *Mol. Microbiol.* 66, 1459–1473. doi: 10.1111/j.1365-2958.2007.06008.x

Hau, H.H., and Gralnick, J.A. (2007). Ecology and biotechnology of the genus *Shewanella*. *Annu. Rev. Microbiol.* 61, 237–258. doi: 10.1146/annurev.micro.61.080706.093257

Hazelbauer, G.L. (2012). Bacterial chemotaxis: the early years of molecular studies. *Annu. Rev. Microbiol.* 66, 285–303. doi: 10.1146/annurev-micro-092611-150120

Hickman, J.W., Tifrea, D.F., and Harwood, C.S. (2005). A chemosensory system that regulates biofilm formation through modulation of cyclic diguanylate levels. *Proc. Natl. Acad. Sci. U.S.A.* 102, 14422–14427. doi: 10.1073/pnas.0507170102

Hong, Y.H., Deng, M.C., Xu, X.M., Wu, C.F., Xiao, X., Zhu, Q., et al. (2016). Characterization of the transcriptome of *Achromobacter* sp. HZ01 with the outstanding hydrocarbon-degrading ability. *Gene* 584, 185–194. doi: 10.1016/j.gene.2016.02.032

Hsing, W., Russo, F.D., Bernd, K.K., and Silhavy, T.J. (1998). Mutations that alter the kinase and phosphatase activities of the two-component sensor EnvZ. *J. Bacteriol.* 180, 4538–4546.

Hsing, W., and Silhavy, T.J. (1997). Function of conserved histidine-243 in phosphatase activity of EnvZ, the sensor for porin osmoregulation in *Escherichia coli*. *J. Bacteriol.* 179, 3729–3735. doi: 10.1128/jb.179.11.3729-3735.1997

Huang, Y., Zhao, K.X., Shen, X.H., Chaudhry, M.T., Jiang, C.Y., and Liu, S.J. (2006). Genetic characterization of the resorcinol catabolic pathway in *Corynebacterium glutamicum*. *Appl. Environ. Microbiol.* 72, 7238–7245. doi: 10.1128/aem.01494-06

Jakobsen, T.H., Hansen, M.A., Jensen, P.O., Hansen, L., Riber, L., Cockburn, A., et al. (2013). Complete genome sequence of the cystic fibrosis pathogen *Achromobacter xylosoxidans* NH44784-1996 complies with important pathogenic phenotypes. *PLoS ONE* 8:e68484. doi: 10.1371/journal.pone.0068484

Journet, L., and Cascales, E. (2016). The Type VI secretion system in *Escherichia coli* and related species. *EcoSal Plus* 7. doi: 10.1128/ecosalplus.ESP-0009-2015

Kamat, S.S., Williams, H.J., and Raushel, F.M. (2011). Intermediates in the transformation of phosphonates to phosphate by bacteria. *Nature* 480, 570–573. doi: 10.1038/nature10622

Kato, A., Ohnishi, H., Yamamoto, K., Furuta, E., Tanabe, H., and Utsumi, R. (2000).

Transcription of *emrKY* is regulated by the EvgA-EvgS two-component system in *Escherichia coli* K-12. *Biosci. Biotechnol. Biochem.* 64, 1203–1209. doi: 10.1271/bbb.64.1203

Kuhlmann, A.U., and Bremer, E. (2002). Osmotically regulated synthesis of the compatible solute ectoine in *Bacillus pasteurii* and related *Bacillus* spp. *Appl. Environ. Microbiol.* 68, 772–783. doi: 10.1128/AEM.68.2.772-783.2002

Levy, S.B., McMurry, L.M., Barbosa, T.M., Burdett, V., Courvalin, P., Hillen, W., et al. (1999). Nomenclature for new tetracycline resistance determinants. *Antimicrob. Agents Chemother.* 43, 1523–1524.

Li, T., Zhao, K., Huang, Y., Li, D., Jiang, C.Y., Zhou, N., et al. (2012). The TetR-type transcriptional repressor RolR from *Corynebacterium glutamicum* regulates resorcinol catabolism by binding to a unique operator, *rolO*. *Appl. Environ. Microbiol.* 78, 6009–6016. doi: 10.1128/aem.01304-12

Liu, B., and Pop, M. (2009). ARDB—Antibiotic Resistance Genes Database. *Nucleic Acids Res.* 37, D443–D447. doi: 10.1093/nar/gkn656

Livermore, D.M. (1995). Beta-Lactamases in laboratory and clinical resistance. *Clin. Microbiol. Rev.* 8, 557–584.

Lynch, B.S., Delzell, E.S., and Bechtel, D.H. (2002). Toxicology review and risk assessment of resorcinol: thyroid effects. *Regul. Toxicol. Pharmacol.* 36, 198–210. doi: 10.1006/rtph.2002.1585

Manivasagan, P., Venkatesan, J., Sivakumar, K., and Kim, S.K. (2013). Marine actinobacterial metabolites: current status and future perspectives. *Microbiol. Res.* 168, 311–332. doi: 10.1016/j.micres.2013.02.002

Metcalf, W.W., Steed, P.M., and Wanner, B.L. (1990). Identification of phosphate starvation-inducible genes in *Escherichia coli* K-12 by DNA sequence analysis of *psi*::*lacZ*(Mu d1) transcriptional fusions. *J. Bacteriol.* 172, 3191–3200. doi: 10.1128/jb.172.6.3191-3200.1990

Metcalf, W.W., and Wanner, B.L. (1993). Evidence for a fourteen-gene, *phnC* to *phnP* locus for phosphonate metabolism in *Escherichia coli*. *Gene* 129, 27–32. doi: 10.1016/0378-1119(93)90692-V

Miethke, M., and Marahiel, M.A. (2007). Siderophore-based iron acquisition and pathogen control. *Microbiol. Mol. Biol. Rev.* 71, 413–451. doi: 10.1128/mmbr.00012-07

Mohn, W.W., and Stewart, G.R. (2000). Limiting factors for hydrocarbon biodegradation at low temperature in Arctic soils. *Soil Biol. Biochem.* 32, 1161–1172. doi: 10.1016/s0038-0717(00)00032-8

Munson, G.P., Lam, D.L., Outten, F.W., and O'Halloran, T.V. (2000). Identification of a copper-responsive two-component system on the chromosome of *Escherichia coli* K-12. *J. Bacteriol.* 182, 5864–5871. doi: 10.1128/JB.182.20.5864-5871.2000

Nie, Y., Tang, Y.Q., Li, Y., Chi, C.Q., Cai, M., and Wu, X.L. (2012). The genome sequence of *Polymorphum gilvum* SL003B-26A1(T) reveals its genetic basis for crude oil degradation and adaptation to the saline soil. *PLoS ONE* 7, e31261. doi: 10.1371/journal.pone.0031261

Nikaido, H. (1996). Multidrug efflux pumps of Gram-negative bacteria. *J. Bacteriol.* 178, 5853–5859. doi: 10.1128/jb.178.20.5853-5859.1996

Noriega, C.E., Lin, H.Y., Chen, L.L., Williams, S.B., and Stewart, V. (2010). Asymmetric cross-regulation between the nitrate-responsive NarX-NarL and NarQ-NarP two-component regulatory systems from *Escherichia coli* K-12. *Mol. Microbiol.* 75, 394–412. doi: 10.1111/j.1365-2958.2009.06987.x

Pavlu, L., Vosahlova, J., Klierova, H., Prouza, M., Demnerova, K., and Brenner, V. (1999). Characterization of chlorobenzoate degraders isolated from polychlorinated

biphenyl-contaminated soil and sediment in the Czech Republic. *J. Appl. Microbiol.* 87, 381–386. doi: 10.1046/j.1365-2672.1999.00830.x

Rojo, F. (2009). Degradation of alkanes by bacteria. *Environ. Microbiol.* 11, 2477–2490. doi: 10.1111/j.1462-2920.2009.01948.x

Rule, C.S., Patrick, M., Camberg, J.L., Maricic, N., Hol, W.G., and Sandkvist, M. (2016). Zinc coordination is essential for the function and activity of the type II secretion ATPase EpsE. *Microbiologyopen* 5, 870–882. doi: 10.1002/mbo3.376

Schluter, A., Nohlen, M., Kramer, M., Defez, R., and Priefer, U.B. (2000). The *Rhizobium leguminosarum* bv. viciae *glnD* gene, encoding a uridylyltransferase/uridylyl-removing enzyme, is expressed in the root nodule but is not essential for nitrogen fixation. *Microbiology* 146 (Pt 11), 2987–2996. doi: 10.1099/00221287-146-11-2987

Schneiker, S., Martins dos Santos, V.A., Bartels, D., Bekel, T., Brecht, M., Buhrmester, J., et al. (2006). Genome sequence of the ubiquitous hydrocarbon-degrading marine bacterium *Alcanivorax borkumensis*. *Nat. Biotechnol.* 24, 997–1004. doi: 10.1038/nbt1232

Schoner, T.A., Gassel, S., Osawa, A., Tobias, N.J., Okuno, Y., Sakakibara, Y., et al. (2016). Aryl polyenes, a highly abundant class of bacterial natural products, are functionally related to antioxidative carotenoids. *ChemBioChem* 17, 247–253. doi: 10.1002/cbic.201500474

Schwarz, S., Kehrenberg, C., Doublet, B., and Cloeckaert, A. (2004). Molecular basis of bacterial resistance to chloramphenicol and florfenicol. *FEMS Microbiol. Rev.* 28, 519–542. doi: 10.1016/j.femsre.2004.04.001

Sperandio, V., Torres, A.G., and Kaper, J.B. (2002). Quorum sensing *Escherichia coli* regulators B and C (QseBC): a novel two-component regulatory system involved in the regulation of flagella and motility by quorum sensing in *E. coli*. *Mol. Microbiol.* 43, 809–821. doi: 10.1046/j.1365-2958.2002.02803.x

Voelkner, P., Puppe, W., and Altendorf, K. (1993). Characterization of the KdpD protein, the sensor kinase of the K^+-translocating Kdp system of *Escherichia coli*. *Eur. J. Biochem.* 217, 1019–1026. doi: 10.1111/j.1432-1033.1993.tb18333.x

von Kruger, W.M., Humphreys, S., and Ketley, J.M. (1999). A role for the PhoBR regulatory system homologue in the *Vibrio cholerae* phosphate-limitation response and intestinal colonization. *Microbiology* 145 (Pt 9), 2463–2475. doi: 10.1099/00221287-145-9-2463

Weinrauch, Y., Guillen, N., and Dubnau, D.A. (1989). Sequence and transcription mapping of *Bacillus subtilis* competence genes *comB* and *comA*, one of which is related to a family of bacterial regulatory determinants. *J. Bacteriol.* 171, 5362–5375. doi: 10.1128/jb.171.10.5362-5375.1989
