# Supplementary material for: Genome Sequencing Reveals the Potential of Achromobacter sp. HZ01 for Bioremediation
Source: Front Microbiol. 2017 Aug 9;8:1507. doi: 10.3389/fmicb.2017.01507 (PMC5552670; doi:10.3389/fmicb.2017.01507)
Supplement: Supplementary file 3 [file Image_1.PDF]

Supplementary Figures

Genome Sequencing Reveals the Potential of *Achromobacter* sp. HZ01 for Bioremediation

Yue-Hui Hong[1], Cong-Cong Ye[1], Qian-Zhi Zhou[1], Xiao-Ying Wu[2], Jian-Ping Yuan[1], Juan Peng[1], Hailin Deng[1,*], Jiang-Hai Wang[1,*]

[1] Guangdong Provincial Key Laboratory of Marine Resources and Coastal Engineering/South China Sea Bioresource Exploitation and Utilization Collaborative Innovation Center, School of Marine Sciences, Sun Yat-Sen University, Guangzhou, People's Republic of China

[2] State Key Laboratory of Conservation and Utilization of Subtropical Agro-Bioresources, College of Natural Resources and Environment, South China Agricultural University, Guangzhou, People's Republic of China

***Correspondence:**
Jiang-Hai Wang
wangjhai@mail.sysu.edu.cn
Hailin Deng
denghlin3@mail.sysu.edu.cn

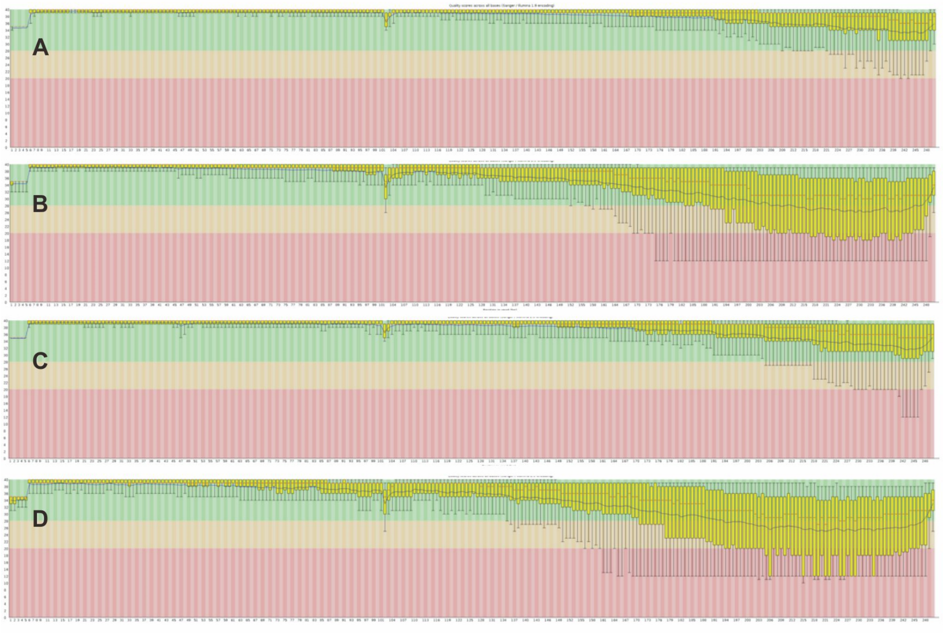

Figure S1. FastQC report (per base sequence quality). The sequences subjected to quality assessment are from the 500 bp-insert-library (**A** and **B**) and 800 bp-insert-library (**C** and **D**), respectively. The report indicates that the sequence quality meets the standard of genome analysis.

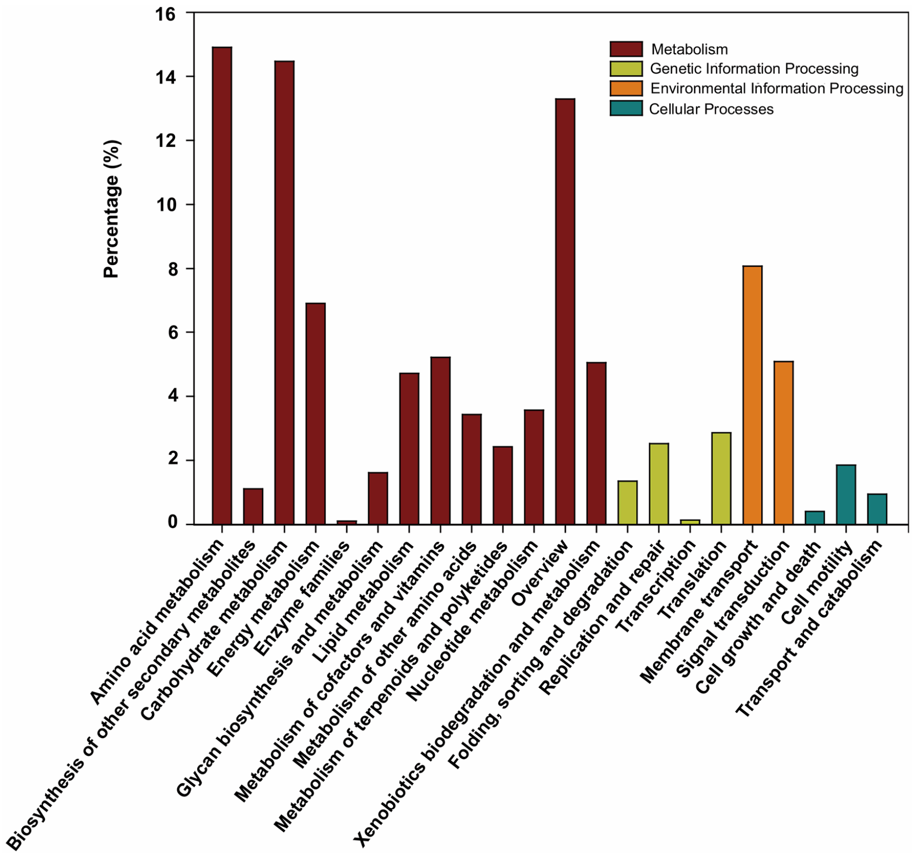

Figure S2. Secondary classification of the annotated KEGG pathways in strain HZ01. The annotation is conducted using the KEGG Automatic Annotation Server (KAAS). A total of 2,972 genes are assigned to pathway categories.

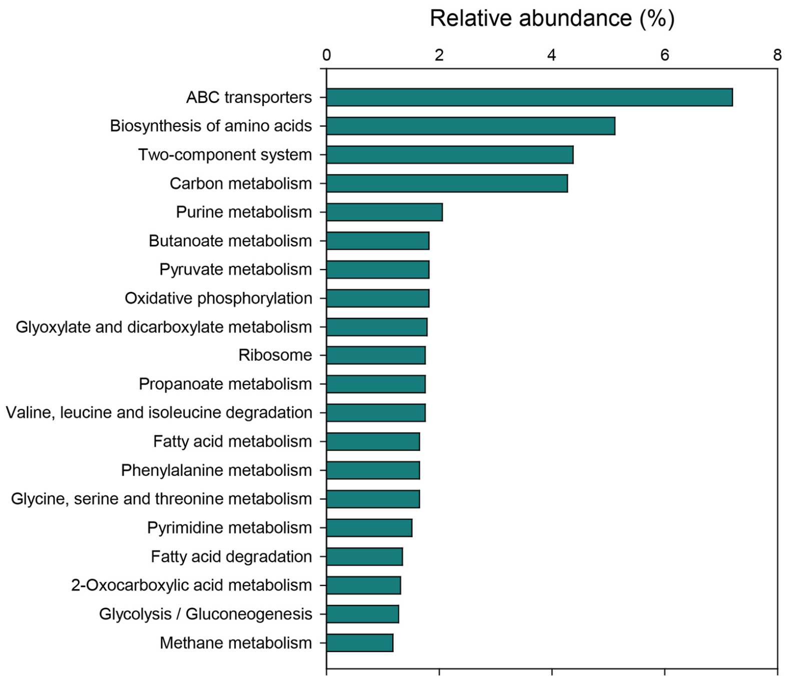

Figure S3. Tertiary classification of the annotated KEGG pathways in strain HZ01. The annotation is performed using the KAAS. A total of 2,972 genes are assigned to pathway categories. The top 20 pathways are shown in the figure.

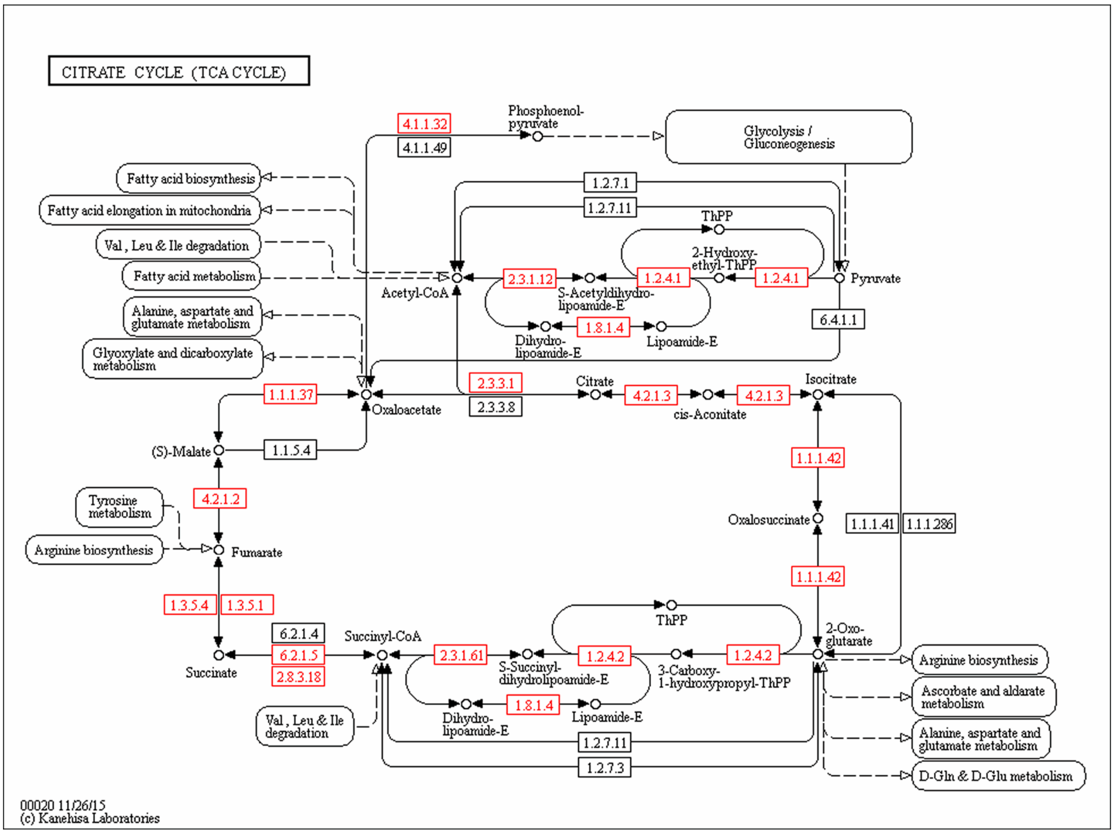

Figure S4. Genes identified in the citrate cycle (TCA cycle) pathway. The classification numbers of enzymes are presented in the boxes. Red boxes indicate the identified proteins in strain HZ01. Genes corresponding to the identified proteins are shown in Table S4.

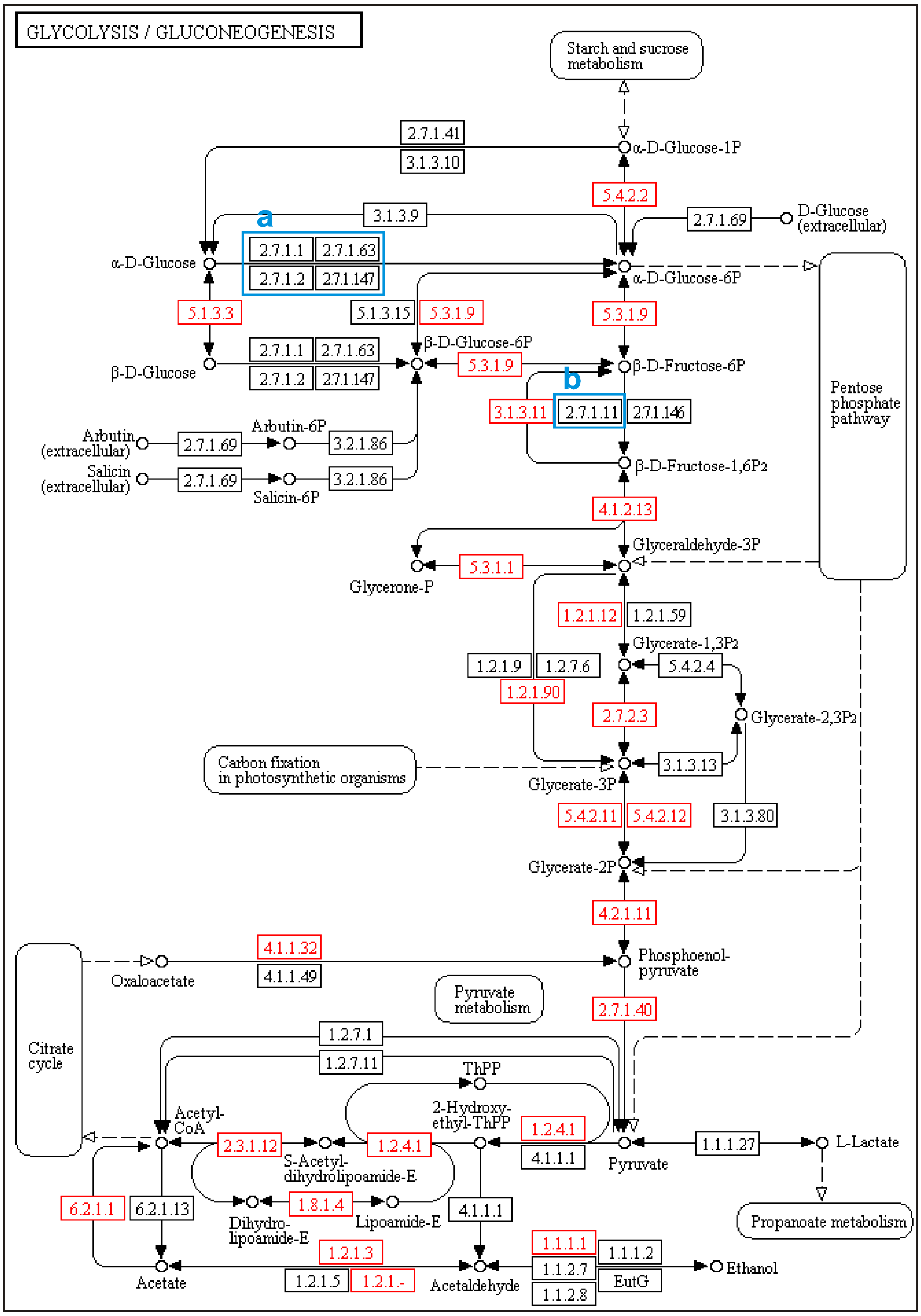

Figure S5. Genes identified in the "glycolysis/gluconeogenesis" pathway in strain HZ01. The classification numbers of enzymes are presented in the boxes. Red boxes indicate the identified enzymes in strain HZ01. a: hexokinase and its homologs; b: 6-phosphofructokinase-1. Genes corresponding to the identified enzymes are shown in Table S4.

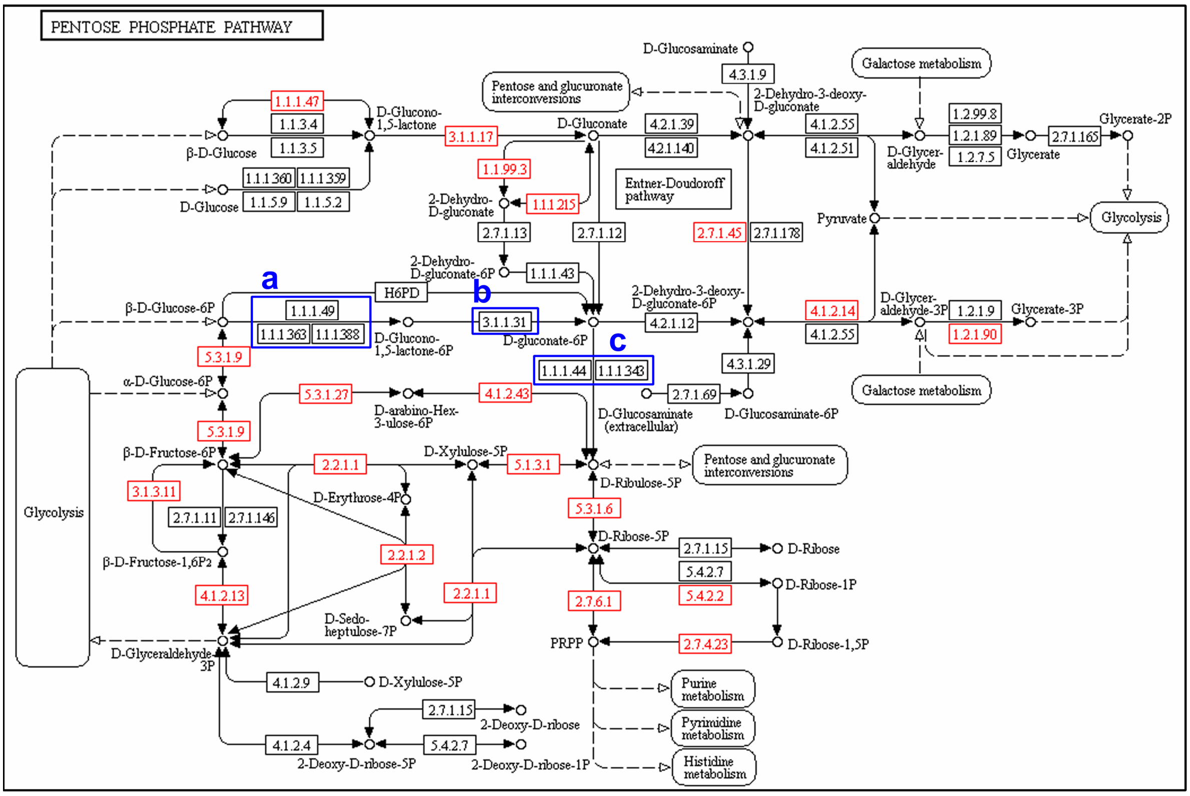

Figure S6. Genes identified in the "pentose phosphate pathway" in strain HZ01. The classification numbers of enzymes are presented in the boxes. Red boxes indicate the identified enzymes in strain HZ01. a： glucose-6-phosphate dehydrogenase； b： 6-phosphogluconolactonase；c： phosphogluconate dehydrogenase. Genes corresponding to the identified enzymes are shown in Table S4.

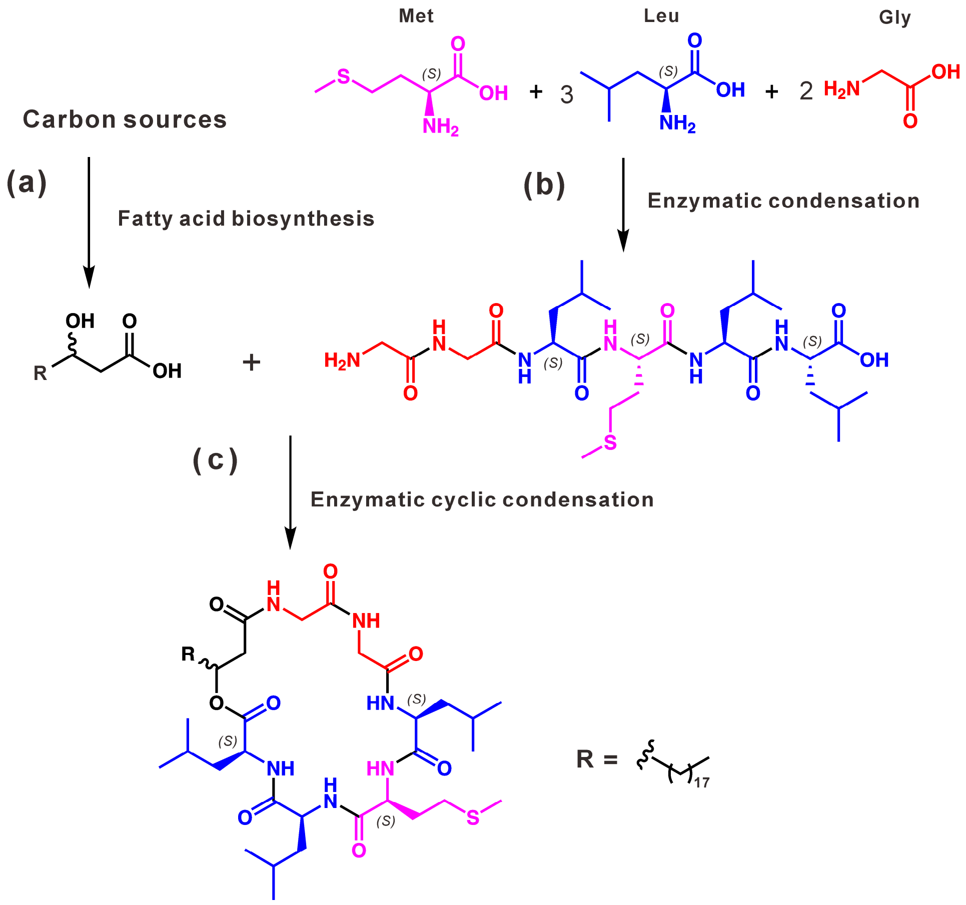

Figure S7. A putative biosynthetic pathway of the cyclic lipopeptide produced by strain HZ01. This pathway includes three major processes. (a) A 3-hydroxy-heneicosanoic acid is produced from fatty acid biosynthesis. (b) One methionine (Met), three leucines (Leu) and two glycines (Gly) are subjected to condensation to produce a hexapeptide. (c) The 3-hydroxy-heneicosanoic acid may undergo an enzymatic condensation process, being incorporated at the C- and N-terminus of the hexapeptide to produce a cyclic lipopeptide.

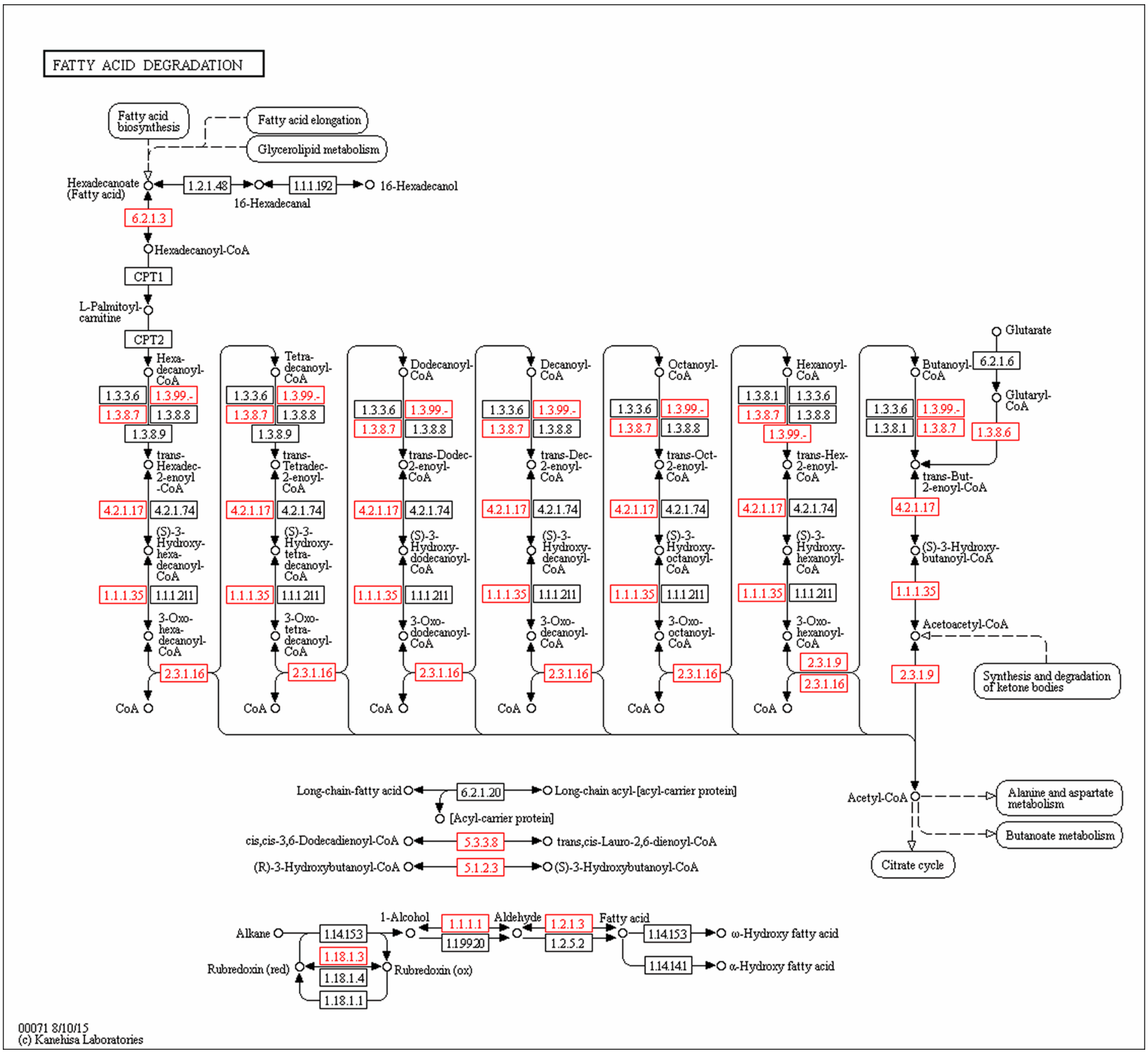

Figure S8. Genes assigned to the "fatty acid degradation" pathway. The classification numbers of enzymes are presented in the boxes. Red boxes indicate the identified enzymes in strain HZ01. Genes corresponding to the identified enzymes are shown in Table S9.

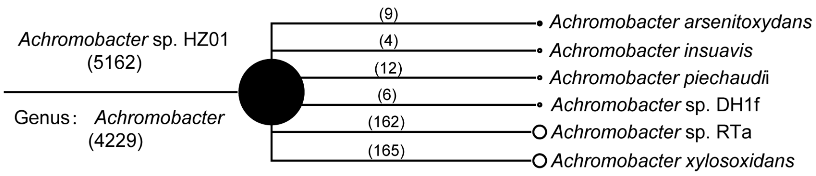

Figure S9. Genes assigned to the genus *Achromobacter* using similarity alignment against the NCBI-nr database. The numbers of genes are shown in the parentheses. A total of 5,162 genes are predicted in the genome of strain HZ01, of which 4,229 genes are assigned to the genus *Achromobacter*. Only several *Achromobacter* strains are shown in the figure.

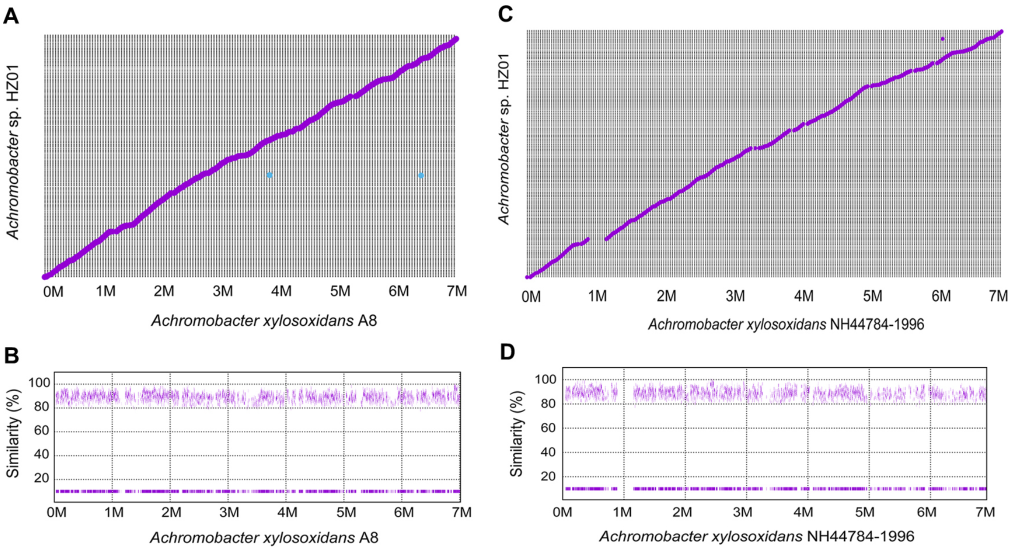

Figure S10. Genome synteny analysis. The results show that the genome of strain HZ01 is mostly co-linear with that of *Achromobacter xylosoxidans* A8 (**A** and **B**) and *Achromobacter xylosoxidans* NH44784-1996 (**C** and **D**), respectively. Consistent matches are slanted from bottom left to upper right and are shown in purple (**A** and **C**). (**B** and **D**) The scatter diagrams indicate the genome similarity at all the nucleotide positions; the discontinuous lines in purple represent the genome coverage of strain HZ01 against the reference genomes.

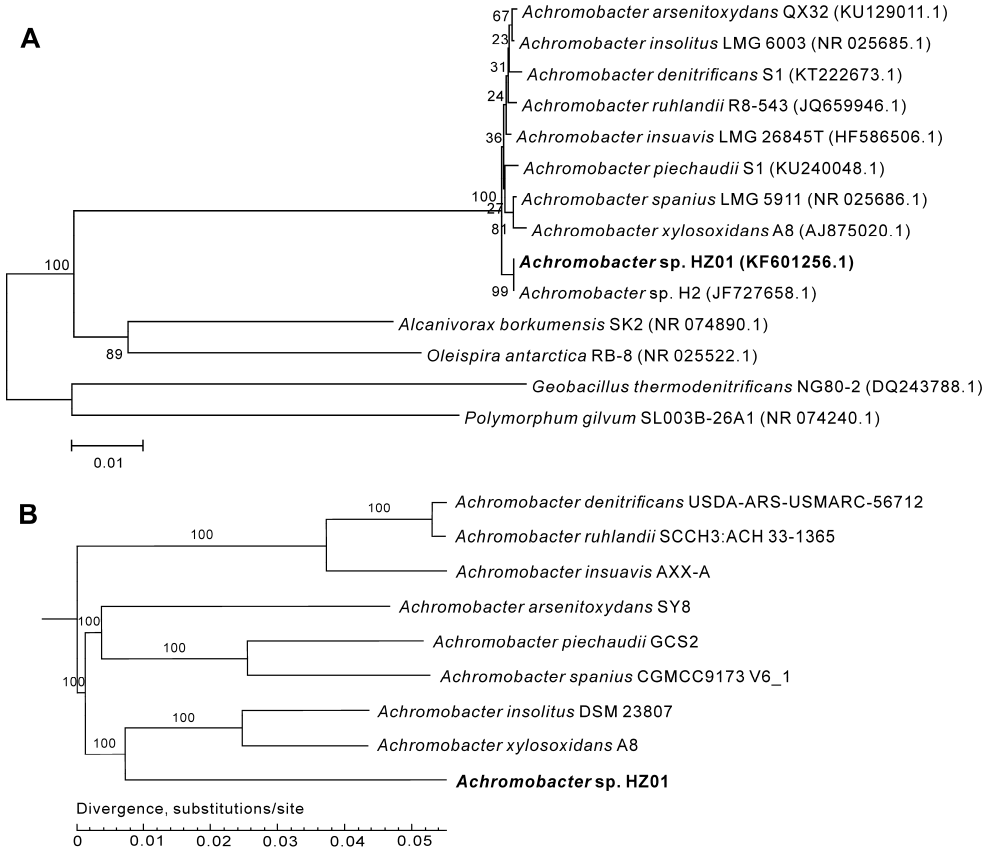

Figure S11. Phylogenetic analysis of *Achromobacter* sp. HZ01 and other strains. (**A**) The phylogenetic tree was constructed based on the 16S rDNA sequences. The sequence accession numbers were shown in the parentheses. (**B**) The phylogenetic tree was constructed according to the core genes of nine *Achromobacter* strains.

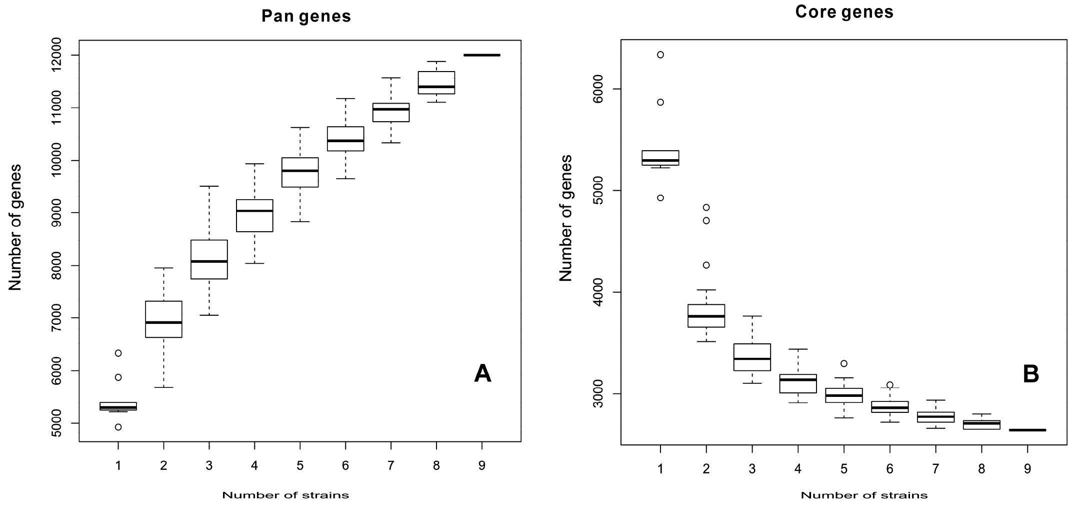

Figure S12. Dilution curves of the pan (A) and core genes (B). A total of 12,000 pan genes and 2,643 core genes were obtained from nine *Achromobacter* strains.

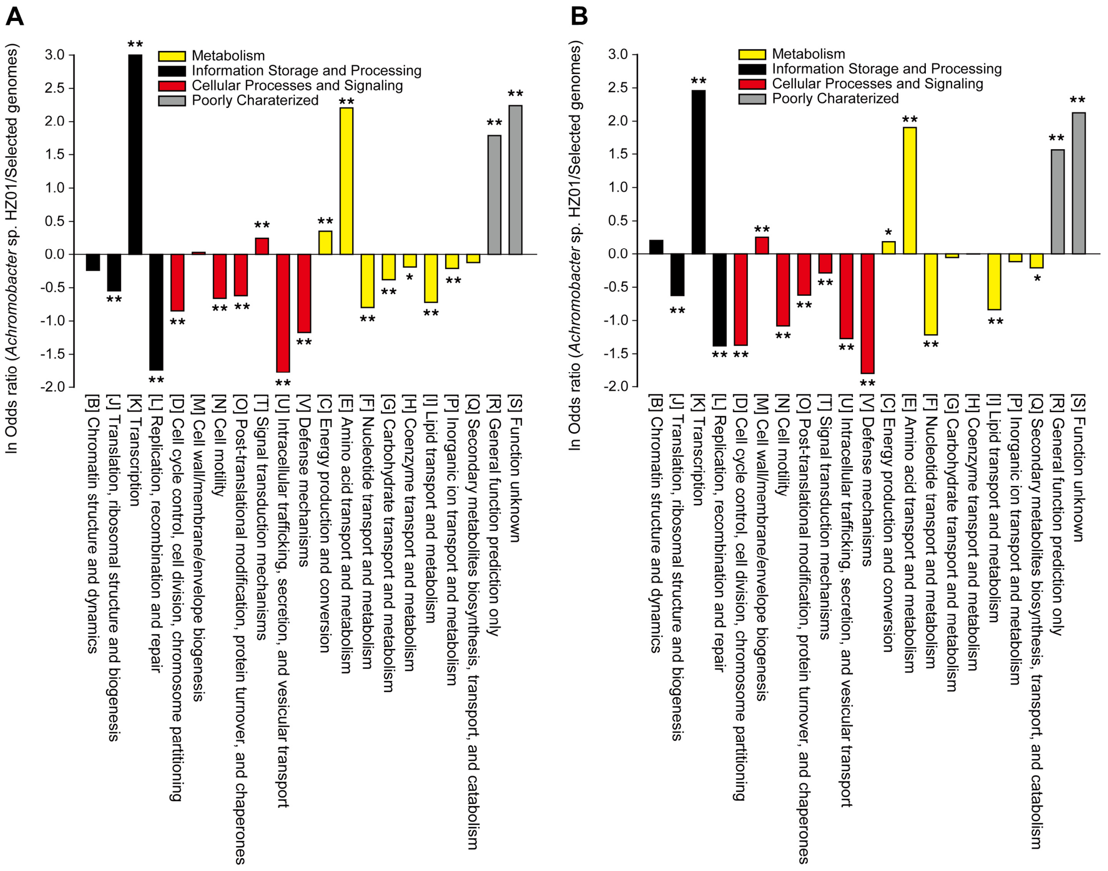

Figure S13. Differences in the gene abundance of COG categories between strain HZ01 and other strains. (**A**) The selected genomes for a comparative study are from *Achromobacter xylosoxidans* A8 and *Achromobacter xylosoxidans* NH44784-1996. (**B**) The selected genomes for comparisons are from *Alcanivorax borkumensis* SK2 and *Polymorphum gilvum* SL003B-26A1^{T}. Asterisks indicate significant deviations determined by two-tailed Fisher exact test.

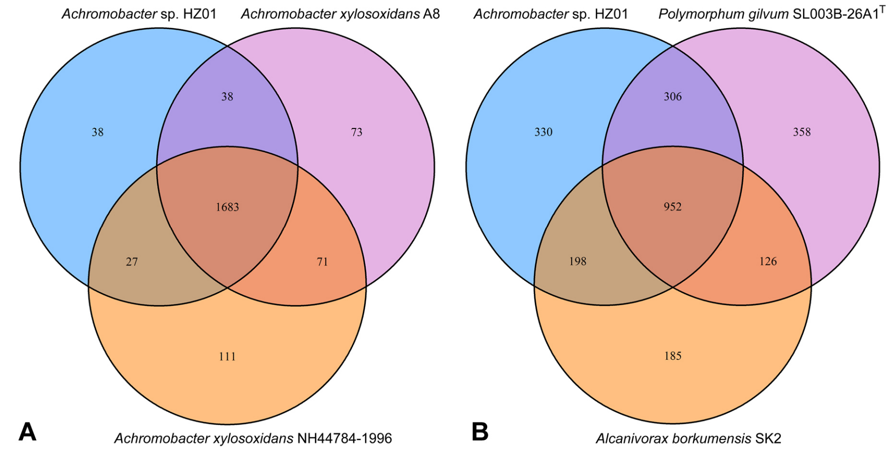

Figure S14. Comparisons of COG catalogues. The Arabic numerals in the circles indicate the numbers of COG catalogues. Each COG catalogue represents an indicated function. The numbers within the overlap sections indicate the identical catalogues shared between strains.

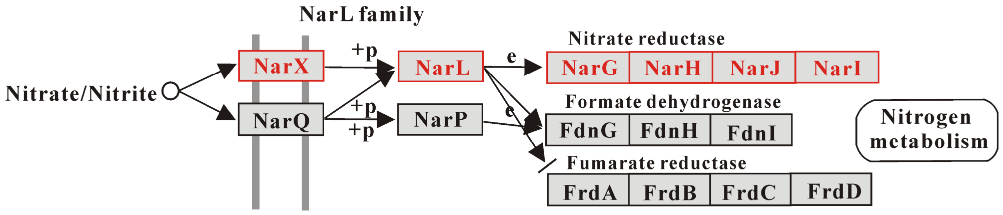

Figure S15. Identified NarX-NarL regulatory system related to nitrogen metabolism. Red boxes indicate the identified proteins in strain HZ01. NarX (gene_1228) and NarL (gene_1227) belong to the NarL family two-component system. NarG (gene_1236), NarH (gene_1235), NarJ (gene_1234), and NarI (gene_1233) are the subunits of nitrate reductase. +p, phosphorylation; e, expression.

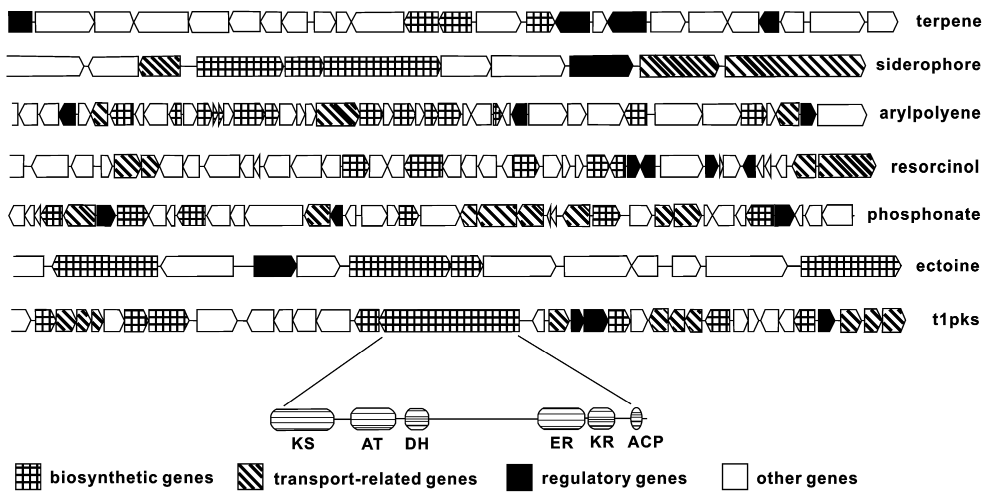

Figure S16. Predicted gene clusters in *Achromobacter* sp. HZ01. The gene cluster prediction is performed by using the antiSMASH. Abbreviations: KS, ketosynthase; AT, acyltransferase; DH, dehydratase; ER, enoylreductase; KR, ketoreductase; ACP, acyl carrier protein. Some more information on the gene clusters is shown in Table S20.

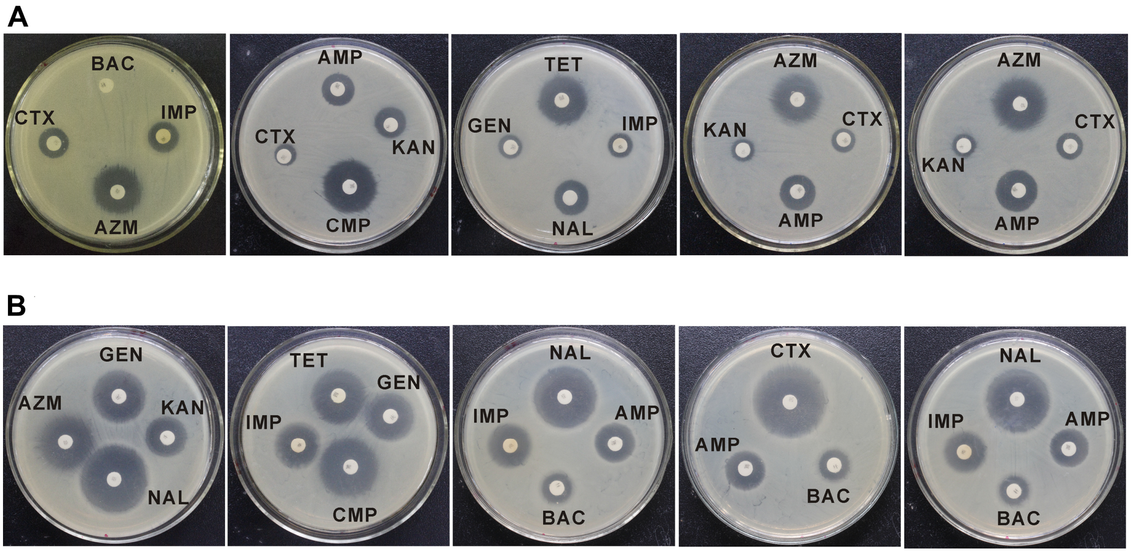

Figure S17. Inhibition zones of the antibiotic resistance assay. The antibiotic susceptibility of strain HZ01 (**A**) is investigated using *E. coli* ATCC 25922 (**B**) for a quality control. Abbreviations: AMP, ampicillin; AZM, azithromycin; BAC, bacitracin; CMP, chloramphenicol; CTX, cefotaxime; GEN, gentamicin; IMP, imipenem; KAN, kanamycin; NAL, nalidixic acid; TET, tetracycline.

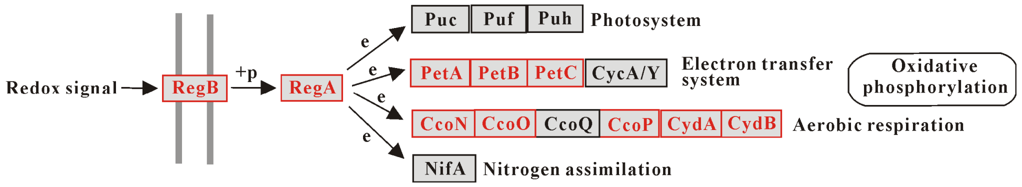

Figure S18. Identified regulatory system RegB/RegA in strain HZ01. Red boxes indicate the identified proteins.

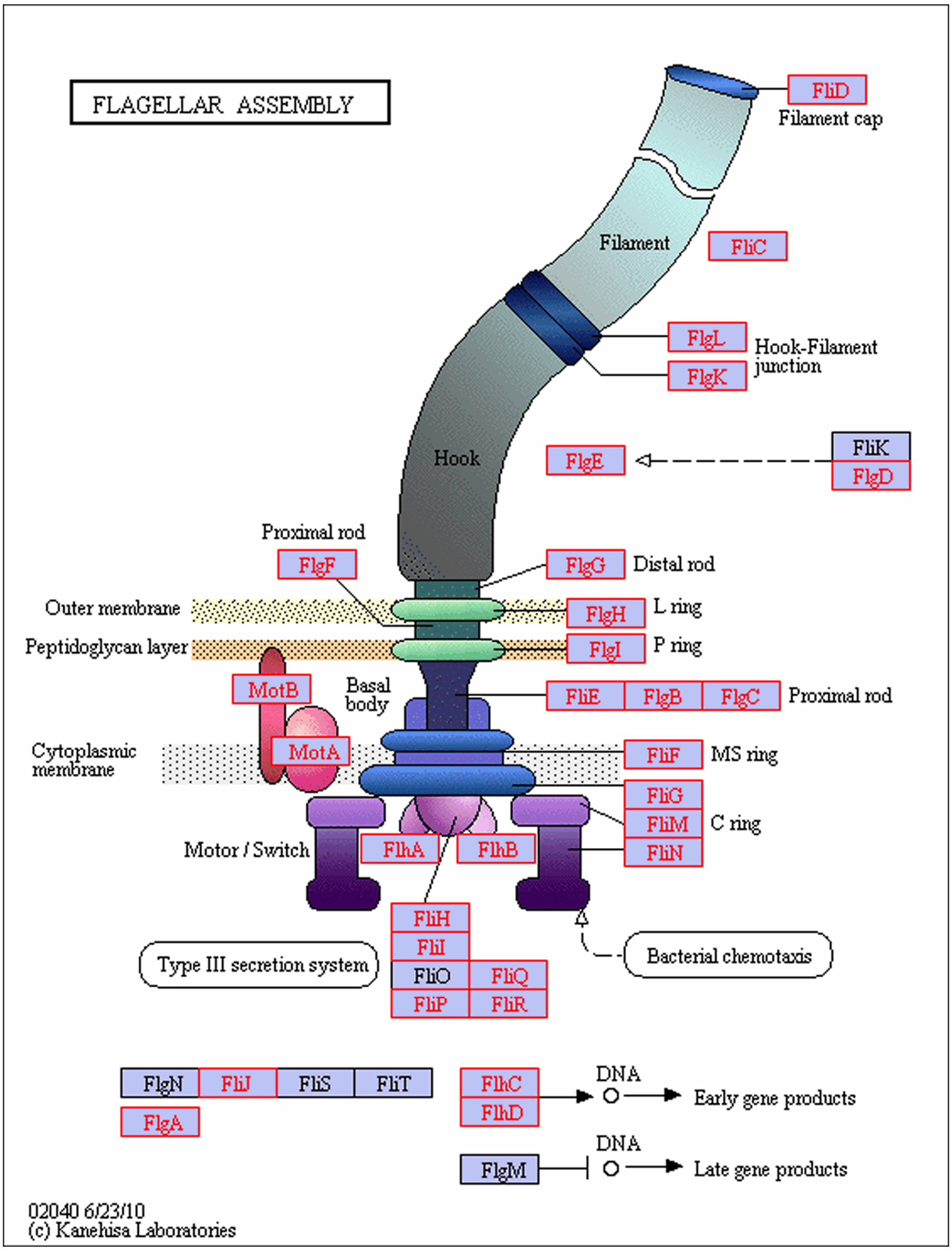

Figure S19. Genes assigned to "flagellar assembly". The protein names are presented in the boxes. Red boxes indicate the identified proteins in strain HZ01. Genes corresponding to the identified proteins are shown in Table S27.

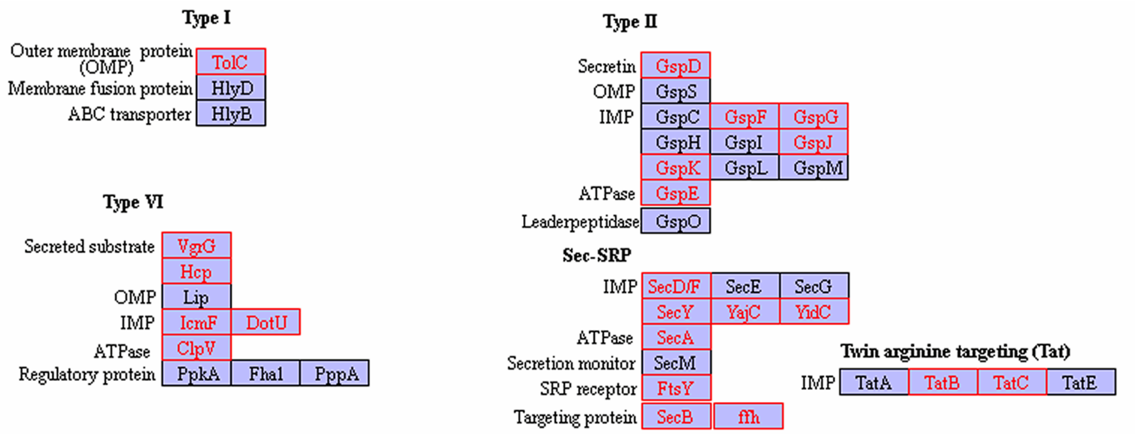

Figure S20. Identified bacterial secretion systems in strain HZ01. The protein names are presented in the boxes. Red boxes indicate the identified proteins in strain HZ01. Genes corresponding to the identified proteins are shown in Table S28.
